# Transcriptome analysis of the response of Burmese python to digestion

**DOI:** 10.1093/gigascience/gix057

**Published:** 2017-07-13

**Authors:** Jinjie Duan, Kristian Wejse Sanggaard, Leif Schauser, Sanne Enok Lauridsen, Jan J. Enghild, Mikkel Heide Schierup, Tobias Wang

**Affiliations:** 1 Bioinformatics Research Center, Aarhus University, C.F. Moellers Alle 8, Aarhus C, Denmark; 2 Department of Molecular Biology and Genetics, Aarhus University, Gustav Wieds Vej 10C, Aarhus C, Denmark; 3 Interdisciplinary Nanoscience Center (iNANO), Aarhus University, Gustav Wieds Vej 14, Aarhus C, Denmark; 4 QIAGEN Aarhus, Silkeborgvej 2, Aarhus C, Denmark; 5 Department of Bioscience, Aarhus University, Ny Munkegade 116, Aarhus C, Denmark

**Keywords:** Burmese python, transcriptome, tissue expression, digestion, pathway, proteome

## Abstract

Exceptional and extreme feeding behaviour makes the Burmese python (*Python bivittatus*) an interesting model to study physiological remodelling and metabolic adaptation in response to refeeding after prolonged starvation. In this study, we used transcriptome sequencing of 5 visceral organs during fasting as well as 24 hours and 48 hours after ingestion of a large meal to unravel the postprandial changes in Burmese pythons. We first used the pooled data to perform a *de novo* assembly of the transcriptome and supplemented this with a proteomic survey of enzymes in the plasma and gastric fluid. We constructed a high-quality transcriptome with 34 423 transcripts, of which 19 713 (57%) were annotated. Among highly expressed genes (fragments per kilo base per million sequenced reads > 100 in 1 tissue), we found that the transition from fasting to digestion was associated with differential expression of 43 genes in the heart, 206 genes in the liver, 114 genes in the stomach, 89 genes in the pancreas, and 158 genes in the intestine. We interrogated the function of these genes to test previous hypotheses on the response to feeding. We also used the transcriptome to identify 314 secreted proteins in the gastric fluid of the python. Digestion was associated with an upregulation of genes related to metabolic processes, and translational changes therefore appear to support the postprandial rise in metabolism. We identify stomach-related proteins from a digesting individual and demonstrate that the sensitivity of modern liquid chromatography/tandem mass spectrometry equipment allows the identification of gastric juice proteins that are present during digestion.

## Background

All animals exhibit dynamic changes in the size and functional capacities of bodily organs and tissues to match energetic maintenance costs to prevailing physiological demands [1]. This phenotypic flexibility is particularly pronounced in the digestive organs in animals that naturally experience prolonged periods of fasting but are capable of ingesting large prey items at irregular intervals. The Burmese python is an iconic example of this extreme phenotype [1]. Many species of pythons easily endure months of fasting, while remaining capable of subduing and ingesting very large meals. In Burmese pythons, digestion is attended by a large and rapid rise in mass and/or functional capacity of the intestine, stomach, liver, heart, and kidneys [2–4] in combination with a stimulation of secretory processes and an activation of enzymes and transporter proteins. These physiological responses are associated with a many-fold rise in aerobic metabolism. Hence, the Burmese python is an excellent model to study the mechanisms underlying extreme metabolic transitions and physiological remodelling in response to altered demand [1, 3, 5–10].

The postprandial changes in the morphology and physiology of the intestine, heart, and other organs have been described in some detail in pythons [1, 5, 8, 9, 11], but only a few studies [12–14] have addressed the underlying transcriptional changes of this interesting biological response. Transcriptome sequencing technology now allows comprehensive surveys [15, 16], prompting our use of transcriptome sequencing of the heart, liver, stomach, pancreas, and intestine in snakes that had fasted for 1 month, at 24 and 48 hours into the postprandial period. These organs were chosen because a number of earlier studies have revealed their profound phenotypic changes during the postprandial period [1–4, 17], and they are therefore likely to exhibit large changes in gene expression. Differential gene expression in some of these organs has previously been reported [12–14], but we provide new data on 48 hours into the digestive period and the first descriptions of gene expression in the stomach and the pancreas. As the Burmese python reference genome assembly [12] is relatively fragmented (contig size N50 ∼10 kb), we found it impractical to use re-sequencing approaches and opted instead to use our high-coverage data to build a *de novo* transcriptome assembly to identify differentially expressed genes (DEGs). To identify the enzymes involved in the digestion process, we isolated the digestive fluid and characterized the protein composition using a proteomics-based approached. This also allowed us to identify the major hydrolytic enzymes used to digest the large and un-masticated meals.

## Analyses

### Data summary

**Figure 1:**
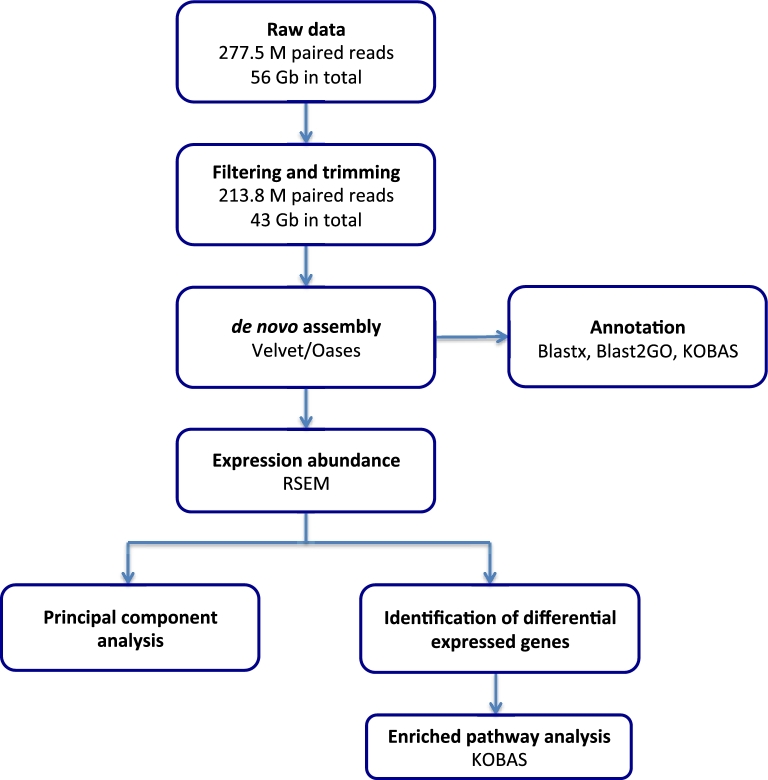
The workflow of Python RNA-Seq data analysis. The diagram shows the main steps and bioinformatics tools used in the study.

A total of 277 485 924 raw paired reads (2*101 bp, insert size 180 bp) were obtained from Illumina Hi-Seq 2000 sequencing of 15 non-normalized cDNA libraries derived from 5 tissues (heart, liver, stomach, pancreas, and intestine) at 3 time points (fasted for 1 month, 24 hours, and 48 hours postprandial) and 10 DSN-normalized cDNA libraries (see the Methods section) (Supplementary Table S1). After removal of low-quality reads (see the Methods section), 213 806 111 (77%) high-quality paired reads were retained. These reads contained a total of 43 146 073 200 bp nucleotides with a mean Phred quality higher than 37 (Q37). To develop a comprehensive transcriptomics resource for the Burmese python (Fig. 1), we pooled these high-quality reads from 25 libraries for subsequent *de novo* assembly.

### *De novo* transcriptome assembly and evaluation

As short k-mers have a higher propensity to generate misassembled transcripts when using a de Bruijn graph–based *de novo* assembler, such as Velvet [18], we conservatively chose an assembly generated using long k-mers for subsequent analysis, at the cost of some sensitivity regarding assembled isoforms. Thus, balancing key metrics (Supplementary Table S2), we used an assembly based on the longest k-mer (= 95) (Table 1) as it had the fewest scaffolds/transcripts (34 423) but represented a very large proportion (74%) of all reads. The scaffold N50 of this assembly was 1673 bp.

**Table 1:** Summary of transcriptome assembly of Burmese python.

Parameter	*De novo* assembly
Total transcripts	34 423
Annotated transcripts with nr NCBI	19 713
Annotated transcripts with GO term	16 992
Minimum transcript size (nt)	100
Medium transcript size (nt)	605
Mean transcript size (nt)	1034
Largest transcript (nt)	26 010
N50	6240
N50 size (nt)	1673
Total assembled bases (Mb)	35.6

To evaluate the accuracy of the transcriptome assembly, we compared it with the Burmese python reference genome (GenBank assembly accession: GCA_000186305.2) and corresponding gene set in the National Center for Biotechnology Information (NCBI) database using rnaQUAST v. 1.4.0 [19]. The transcriptome assembly had 34 423 transcripts in total; 34 040 (98%) of these transcripts had at least 1 significant alignment to the reference genome, and 31 102 (91%) out of 34 040 were uniquely aligned (Supplementary Table S3). The average aligned fraction (i.e., total number of aligned bases in the transcript divided by the total transcript length) was 0.975 (Supplementary Table S3). The high concordance between the *de novo* transcript assembly and the genome reference strengthened our confidence in using the *de novo* assembly as our reference and showed that the individual fragments were accurate although the reference genome assembly was fragmented. By aligning assembled sequences back to the reference genome, we reviewed the chimeric assembled sequences that had discordant best-scored alignment (partial alignments that are either mapped to different strands/different chromosomes/in reverse order/too distant) and found 1974 (5.7%) misassembled (chimeric) transcripts (Supplementary Table S3). Considering that some of these sequences could potentially be correct, we included all sequences in our subsequent analysis, but also provided chimeric sequences in a supplementary FASTA file. The comparison of assembled sequences and reference gene sequences (Supplementary Table S3) showed that 26 320 (77.3%) assembled transcripts cover at least 1 isoform from the reference gene set, and the mean fraction of matched transcript is 67.8%, suggesting that there is a good concordance but also some differences, which can be due to errors in either the reference genome assembly/annotation or our assembly. In addition, we assessed the completeness of our transcriptome assembly with the Benchmarking Universal Single-Copy Orthologs (BUSCO) strategy. Results comprised 55.2% (1428 out of 2586) complete BUSCOs, 19.8% (512) fragmented BUSCOs, and 25% (646) missing BUSCOs. These results are consistent with a survey [20] of assessment completeness of 28 transcriptomes from 18 vertebrates. In this survey, most of the transcriptomes from species with close phylogenetic relationships to snakes contain less than 50% complete BUSCOs and more than 40% missing BUSCOs. Therefore, we conclude that the quality of our transcriptome assembly was acceptable.

### Transcriptome annotation

A total of 19 713 transcripts (57% of 34 423) were annotated using transfer of blastx hit annotation against the non-redundant (nr) NCBI peptide database [21]. To assign proper annotation for each transcript, we chose the first best hit that was not represented in uninformative descriptions (Supplementary Table S4). The most closely related species with an annotated genome, *Anolis carolinensis*, was able to annotate 10 704 transcripts (54% of all annotated transcripts). Burmese Python and *Anolis carolinensis* both belong to the reptilian order Squamata and diverged from each other approximately 160 million years ago [22].

Blast2GO was used to annotate these 19 713 transcripts [23], of which 16 992 could be assigned to 1 or more gene ontology (GO) terms and their putative functional roles. The distributions of the most frequently identified GO term categories for biological processes, molecular function, and cellular component are shown in Fig. S1. Moreover, we used the functionality of InterPro [24] annotations in Blast2GO to retrieve domain/motif information for our transcripts, and 21 023 transcripts were annotated by the InterPro database.

### Gene expression analysis and principal component analysis

For comparisons between genes, expression profiles were obtained by mapping high-quality reads to the reference transcriptome, and the expression level was given by fragments per kilo base per million sequenced reads (FPKM) [25]. For the study of expression profiles, we chose to investigate 1862 highly expressed genes (FPKM ≥ 100 in at least 1 of 15 tissues) as it is known that for highly expressed genes, the biological variation among biological replicates in the same tissue at the same stage is lower than for genes showing low expression levels [26]. The majority (∼64%) of these 1862 genes were expressed in all tissues, and only ∼18% were expressed solely in 1 tissue (Supplementary Fig. S2). The liver had the highest number of uniquely expressed genes, which may reflect its particular role in metabolism and excretion of waste products.

**Figure 2:**
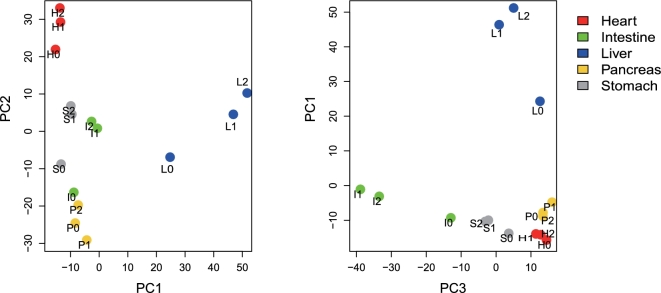
PCA plots of FPKM of 1862 genes. PC: principal component. PC1 represents 25%, PC2 represents 18%, and PC3 represents 16% of total variation in the data. The name of the label consists of 2 parts: 1 capital letter plus 1 number. The letters H, S, I, L, and P represent heart, stomach, intestine, liver, and pancreas, respectively. Numbers 0, 1, and 2 represent fasting for 1 month, 24 hours/1day after feeding, and 48 hours/2 days after feeding, respectively.

We used principal component analysis (PCA) to reveal overall differences in gene expression patterns among tissues and time points within the digestive period. The first 3 principal components (PCs) accounted for ∼58% of the variation (Supplementary Fig. S3). Despite the large overlap in expressed genes (Supplementary Fig. S2), the different tissues exhibited distinct transcriptional signatures, shown by the PCA in Fig. 2, showing a tendency for 24 hours to represent an intermediate position between fasting and 48 hours. Liver, intestine, and stomach displayed greater shifts in the PCA plots compared to the heart and pancreas, and the largest changes occurred between fasting and 24 hours in the stomach and intestine. This fits well with the expectation that the stomach and intestine respond early in digestion [3]. The dramatic changes in gene expression in the liver are also consistent with previous observations in pythons [12].

### Pattern of transcriptional responses to feeding

**Figure 3:**
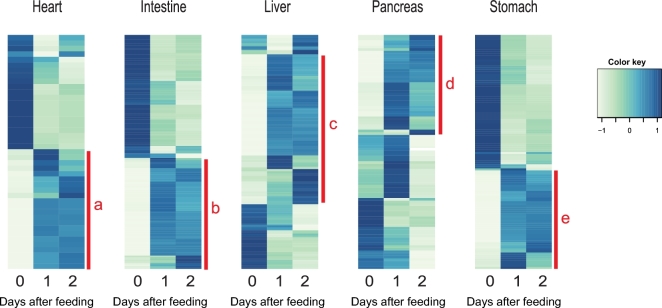
Heat maps from hierarchical clustering of DEGs in each tissue. Heat maps showing the hierarchically clustered Spearman correlation matrix resulting from comparing the normalized FPKM value for each pair of genes. Heat map columns represent samples, and rows correspond to genes. Expression values (FPKM) are log_2_-transformed and then median-centred by gene. Relative levels of gene expression are represented by colours. Pale colour is low expression, and darker blue is high expression. Five sub-clusters, labelled a–e, are shown with full annotation in Figs 4–8.

**Figure 4:**
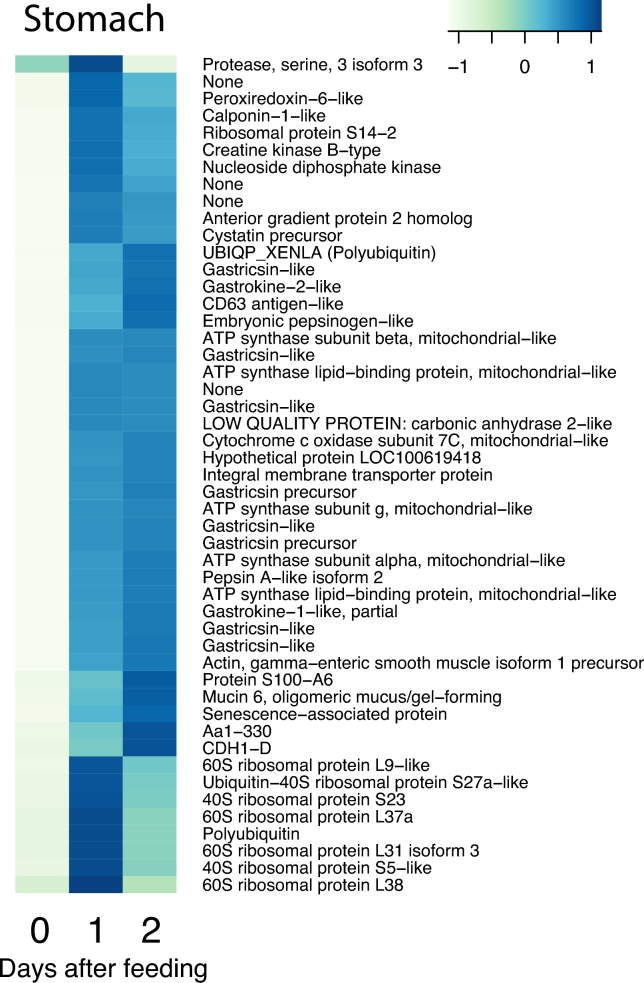
The cluster of upregulated genes with NCBI nr annotation in the stomach. The figure represents cluster e in Fig. 3. Heat map columns represent samples, and rows correspond to genes. Expression values (FPKM) are log_2_-transformed and then median-centred by gene. Relative levels of gene expression are represented by colours. Pale colour is low expression, and darker blue is high expression.

**Figure 5:**
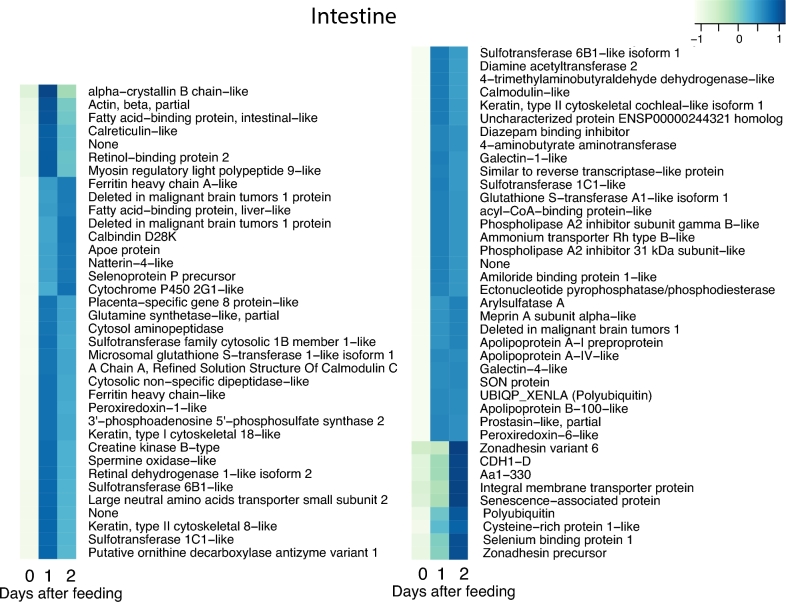
The cluster of upregulated genes with NCBI nr annotation in the intestine. The figure represents cluster b in Fig. 3. Heat map columns represent samples, and rows correspond to genes. Expression values (FPKM) are log_2_-transformed and then median-centred by gene. Relative levels of gene expression are represented by colours. Pale colour is low expression, and darker blue is high expression.

**Figure 6:**
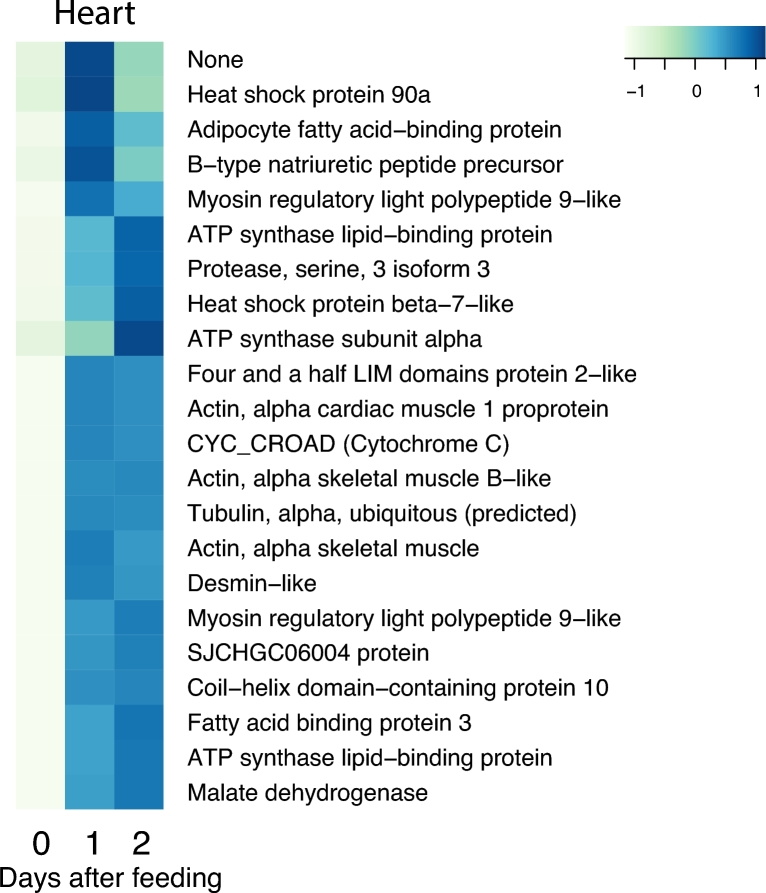
The cluster of upregulated genes with NCBI nr annotation in the heart. The figure represents cluster a in Fig. 3. Heat map columns represent samples, and rows correspond to genes. Expression values (FPKM) are log_2_-transformed and then median-centred by gene. Relative levels of gene expression are represented by colours. Pale colour is low expression, and darker blue is high expression.

**Figure 7:**
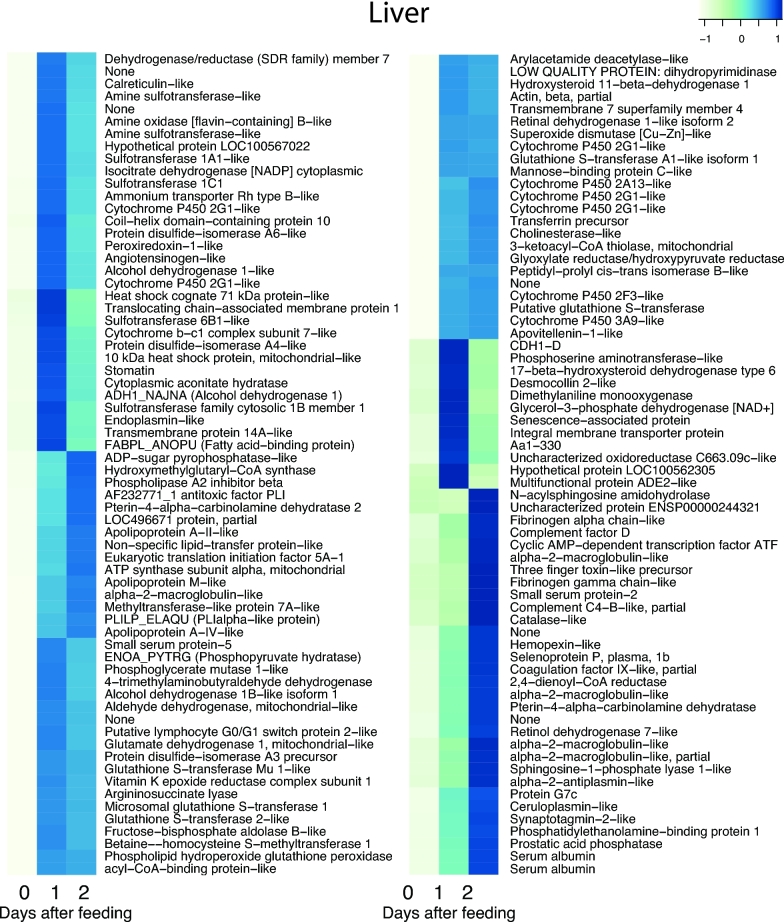
The cluster of upregulated genes with NCBI nr annotation in the liver. The figure represents cluster c in Fig. 3. Heat map columns represent samples, and rows correspond to genes. Expression values (FPKM) are log_2_-transformed and then median-centred by gene. Relative levels of gene expression are represented by colours. Pale colour is low expression, and darker blue is high expression.

**Figure 8:**
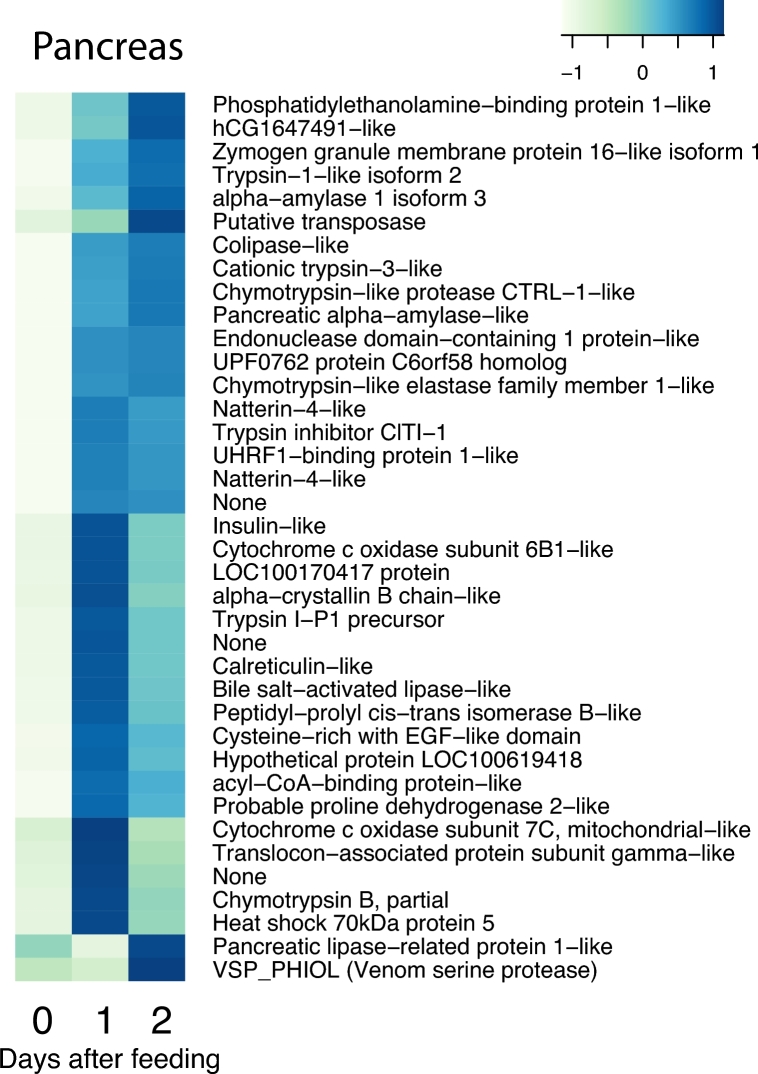
The cluster of upregulated genes with NCBI nr annotation in the pancreas. The figure represents cluster d in Fig. 3. Heat map columns represent samples, and rows correspond to genes. Expression values (FPKM) are log_2_-transformed and then median-centred by gene. Relative levels of gene expression are represented by colours. Pale colour is low expression, and darker blue is high expression.

The postprandial response involves thousands of genes and large changes in gene expression. To restrict the analysis of these numerous genes, we used a conservative approach where we selected genes that are both highly and differentially expressed with 2 strict thresholds (see the Methods section). Application of these 2 thresholds yielded 43 genes for the heart, 206 genes for the liver, 114 genes for the stomach, 89 genes for the pancreas, and 158 genes for the intestine, respectively, that were differentially expressed in response to digestion (Fig. 3). To illustrate this in greater detail, we enlarged the 5 sub-clusters with the most prominent increase in expression. These sub-clusters, labelled a–e in Fig. 3, are shown with full annotation in Figs 4–8. To unravel the functional implications of these responses, we searched for genes encoding for proteins involved in processes of tissue re-organization, cellular metabolism, and digestion within these sub-clusters for each organ.

### GO enrichment analysis and coloured KEGG pathway maps

To gain broader biological insight, in functional annotation analysis we applied a looser threshold set (Table 2) to define DEGs as both maximum FPKM (of 3 time points) over 10 and fold change (FC) over 4 (along with digestion) and highly expressed genes as both maximum FPKM over 200 and FC below 4. The summary of number of genes differentially expressed during digestion in each tissue is illustrated in Table 3. In each organ, most genes (>76%) have low expression (max FPKM < 10). Around 1% of the genes are highly expressed (max FPKM ≥ 200). The number of upregulated genes is approximately 3% in each organ, except for the heart, where only 0.57% of the genes were upregulated in response to feeding. This suggests that during digestion, the digestive organs, like the liver, stomach, intestine, and pancreas show more pronounced post-feeding response than the heart. To dissect the functions of DEGs, we performed GO enrichment analysis with upregulated genes and highly expressed genes, respectively, for each organ (Supplementary Figs S4–S8). As an example, the GO terms most significantly associated with upregulated genes in the stomach were “mitochondrial respiratory chain complex 1,” “endoplasmic reticulum membrane,” and “cytosol” (Supplementary Fig. S4A).

**Table 2:** Colour coding of genes in KEGG pathway maps.

Expression level	Fold change level	Expression trend (fasting -> 24h -> 48h)	Color code
max FPKM over 10	FC over 4	Upregulated	Red
		Downregulated	Blue
		Up- then downregulated	Yellow
		Down- then upregulated	Brown
	FC below 4	Highly expressed (max FPKM over 200)	Purple
		Moderately expressed (max FPKM below 200)	Pink
max FPKM below 10	–	Lowly expressed	Dark grey

Three criteria are used to classify and colour genes. First, (i) whether the maximum FPKM of the gene among fasting, 24 hours, and 48 hours is over 10, then (ii) whether the gene is differentially expressed in at least 1 of the pairwise comparisons among fasting, 24 hours, and 48 hours with FC over 4. Finally, (iii) for those genes expressed, but not differentially expressed, whether it is highly expressed with maximum FPKM among 3 time points over 200. The term expression trend indicates the trend of gene expression across fasting, 24 hours, and 48 hours; e.g., the trend up means the gene is upregulated from either fasting to 24 hours, fasting to 48 hours, or 24 hours to 48 hours. The trend up- then downregulated means the gene is first upregulated from fasting to 24 hours, then downregulated from 24 hours to 48 hours.

**Table 3:** The number of DEGs across fasting, 24 hours, and 48 hours in each tissue.

Expression trend (fasting -> 24h -> 48h)	Stomach	Intestine	Pancreas	Liver	Heart
Upregulated	932 (2.9%)	1131 (3.5%)	859 (2.6%)	1047 (3.2%)	184 (0.6%)
Up- then downregulated	28 (0.1%)	31 (0.1%)	150 (0.5%)	61 (0.2%)	6 (0.0%)
Downregulated	869 (2.7%)	625 (1.9%)	567 (1.7%)	618 (1.9%)	168 (0.5%)
Down- then upregulated	36 (0.1%)	45 (0.1%)	127 (0.4%)	90 (0.3%)	16 (0.1%)
Highly expressed	199 (0.6%)	211 (0.7%)	225 (0.7%)	354 (1.1%)	232 (0.7%)
Moderately expressed	5541 (17.0%)	5582 (17.2%)	4933 (15.2%)	5385 (16.5%)	6044 (18.6%)
Lowly expressed	24 926 (76.6%)	24 906 (76.5%)	25 670 (78.9%)	24 976 (76.8%)	25 881 (79.5%)
Total	32 531 (100%)	32 531 (100%)	32 531 (100%)	32 531 (100%)	32 531 (100%)

The expression trend is consistent with the definition in Table 2.

To specifically identify the pathways associated with DEGs and highly expressed genes, we mapped genes to Kyoto Encyclopedia of Genes and Genomes (KEGG) [27, 28] human pathway maps and coloured the mapped entries with trends of gene expression during digestion (Table 2). We identified the upregulated genes and highly expressed genes, respectively, involved in 3 selected pathways (glycolysis/gluconeogenesis, citrate cycle [tricarboxylic acid (TCA) cycle], and oxidative phosphorylation) for each tissue (Supplementary Table S5), and we performed the same identification for 2 main pathway categories in the KEGG pathway database (1.3 lipid metabolism and 1.5 amino acid metabolism) (Supplementary Table S6). The glycolysis/gluconeogenesis pathway, glyceraldehyde-3 phosphate dehydrogenase, showed high expression in all organs.

### Identification of the python gastric juice proteome

**Figure 9:**
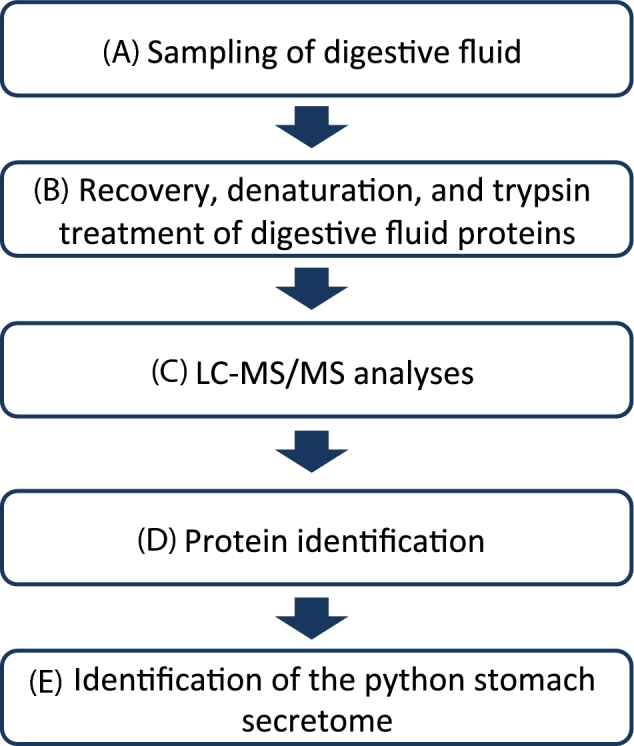
The workflow used to identify the python's stomach secretome during digestion. (**A**) Initially pythons were fed with mice or a peptide mixture, and later the gastric juice samples were obtained and mice debris was removed. (**B**) The proteins were precipitated, denatured, and digested with trypsin. (**C**) The resulting tryptic peptides were analysed by LC-MS/MS analyses, and the data were merged into a single file. (**D**) The file was used to interrogate the in-house generated python protein sequence database (based on the transcriptomic data), and python proteins were identified. (**E**) The data were filtered to remove mouse proteins and plasma proteins. Subsequently, the annotation of the remaining proteins was reassessed and the secretome identified.

We identified the secretome of the python stomach during digestion (Fig. 9). The resulting mass spectrometry data (containing 122 538 MS/MS spectra) was used to interrogate our python transcriptome database, which included transcriptome from stimulated stomach tissue. In total, 549 python proteins were identified using this approach. Afterwards, all identifications based on a single tryptic peptide were removed, reducing the number of identified python proteins to 314 (Supplementary Table S7).

Five classical types of pepsinogens exist, namely pepsinogen A, B, and F, progastricsin (or pepsinogen C), and prochymosin [29]. Of these, our analyses (Supplementary Table S8) show that pythons primarily rely on progastricsin for proteolytic digestion as the 5 most abundant proteases identified in the gastric juice are annotated as progastricsin-like. We aligned the 6 gastricsin-like transcript sequences using webPRANK [30] on amino acid level and calculated the pairwise distance between sequences using Tajima-Nei model (Supplementary Table S9) in MEGA7 [31]. The mean pairwise distance of 1.16 suggests considerable differences in their sequences, which indicate the presence of numerous different proteins with similar functions. This annotation is based on accession XP_003220378.1 and XP_003220378.1 from *Anolis carolinensis.* Alignment of the python sequences with the 2 anole sequences, as well as with the well-characterized human gastricsin variant, shows that both the active site residues, as well as cysteine bridges, are conserved. It demonstrates the similarity between these enzymes and suggests that the identified python sequences indeed represent catalytically active proteolytic enzymes (Supplementary Fig. S9). The last identified pepsinogen-like python sequence (m.31615_Py95) was annotated based on the predicted embryonic pepsinogen-like sequence (XP_003220239.1), also from *Anolis carolinensis.* Here, the annotation originates from an embryonic pepsinogen identified in chicken [32]. This protease was identified in the python's gastric juice with a lower emPAI value than the gastricsin sequences, indicating a lower concentration of this enzyme (Supplementary Table S8), although the transcript displays the highest concentration of the analysed pepsinogens in the postprandial period (Supplementary Table S9). As the name indicates, it is exclusively expressed during the embryonic period in chickens [32, 33], and phylogenetic analysis of the sequence suggests that its closest homolog, among the classical pepsinogens, is prochymosin [32]. Prochymosin also displays a temporal expression pattern and is, in mammals, mainly expressed in newborn species. However, the identified python embryonic-chicken-pepsinogen homolog does not display a similar development-related temporal expression pattern and is, as shown, produced among adult specimens during digestion. However, this does not exclude the protease also being expressed during the python's embryonic phase.

### Identification of prey proteins and the python plasma proteome

Many of the obtained MS/MS spectra were expected to correspond to abundant mice proteins, such as collagen. To facilitate the downstream analyses of python proteins, we produced a list of background proteins related to the prey. Hence, cross-examination of the mass spectrometry data with the 16 693 mouse protein sequences in the Swiss-Prot database was performed, resulting in the identification of 212 mouse proteins, after removing hits based on single peptides (Supplementary Table S10). To produce a list of identified python proteins most likely present in the digestive fluid samples due to blood contamination during collection, we characterized the python plasma proteome. The most abundant plasma proteins are produced by the liver. Consequently, our python transcriptome sequence database, which encompasses liver transcriptomes, is expected to contain the protein sequences of the python plasma proteins. Thus, our python plasma liquid chromatography/tandem mass spectrometry (LC-MS/MS) data were used to interrogate our python sequence database. Aan overview of the most abundant python plasma proteins is provided in Supplementary Table S11. In total, 64 plasma proteins were identified with a minimum of 2 tryptic peptides. We observed a limited correlation of *R*^2^ = 0.13 fitted with a linear model (Supplementary Table S11) between these abundant (based on emPAI) plasma protein expressions and corresponding mRNA expression levels (based on FPKM value at 1 day post-feeding in the liver). One protein that stands out is the anti-haemorrhagic factor cHLP-B (m.27_Py95), which appeared in high concentrations in the plasma of these snakes. This is a protease inhibitor of the haemorrhagic-causing metalloproteinases present in snake venom, and these inhibitors have previously been purified from the serum of venomous snakes [34, 35] and have been proposed to inhibit deleterious actions of venom enzymes in non-venomous snakes [36]. It is, however, also possible that it is an ancestral gene with a function not related to venom production.

### Identification of the python stomach secretome

To identify the python stomach secretome, the list of python proteins identified in the digestive fluid (Supplementary Table S7) was analysed further. We assumed no overlap between abundant plasma proteins and proteins secreted by the stomach. Thus, plasma proteins, identified in the gastric juice, were assumed to be contaminations from blood, and therefore the 64 identified plasma proteins were, when present, removed from the list. Subsequently, python proteins that most likely were identified based on prey protein homology (e.g., mouse collagens and keratins, as well as conserved intracellular household proteins) were removed. These 2 steps reduced the list of proteins identified in the stomach samples from 314 to 114 proteins (Supplementary Table S12). It cannot be excluded that a few proteins belonging to the python stomach secretome also were removed.

To identify the secretome, the 114 identified proteins were manually analysed as described in the Methods section (Supplementary Table S12). In addition to household proteins, the identified intracellular proteins also included intracellular stomach-specific proteins (e.g., the stomach-specific calpain 9 cysteine protease) [37], underlining the specificity of the proteomics analysis. In total, 37 proteins constituted the putative python stomach secretome (Supplementary Table S8). These could be divided into 18 gastric mucosal-related proteins (e.g., mucin homologous and gastrokine), 7 proteolytic enzymes (mainly pepsin homologous), 4 other hydrolytic enzymes (e.g., phospholipases), and 8 other proteins (e.g., gastric intrinsic factor) (Supplementary Table S8). Here, we identify stomach-related proteins from a digesting individual and thereby demonstrate that the sensitivity of modern LC-MS/MS equipment allows the identification of gastric juice proteins that are present during digestion.

## Discussion

As a primary motivation, we wished to describe the temporal changes in gene expression in the visceral organs of Burmese pythons during the transition from fasting to digestion and identify key regulatory genes and pathways responsible for the pronounced tissue restructuring, increased metabolism, and increased functional capacity during the postprandial period. We achieved these goals by identifying the biochemical and physiological roles of highly expressed genes with increased expression during digestion and by using KEGG analysis of the specific pathways underlying physiological responses known to be stimulated by digestion. We also present GO enrichment analyses of both upregulated genes and highly expressed genes in all organs (Supplementary Figs S4–S8), showing that “biological process” is the most common enriched category.

The influence of digestion on gene expression profiles in the heart, liver, kidney, and small intestine has been studied previously in pythons [12–14]. These earlier studies reported thousands of genes being either up- or downregulated within the first day of digestion [12–14], and we confirm these substantial changes in gene expression at 24 hours and 48 hours. However, we merely identified hundreds of genes, probably because we selected a more stringent threshold for defining differential expression. Given the differences in the selection thresholds and analysis strategies for differential expression and differences in the times of sampling, it is difficult to make a direct comparison between our study and that of Castoe et al. (2013) [12]. Nevertheless, for the heart, liver, and small intestine, both studies have determined a number of upregulated genes at 24 hours, where we identified 15, 93, and 61 upregulated genes, respectively. Comparing upregulated genes between 2 studies (see the Supplementary Material for detailed methods and results), we found that in the liver more than half of the 93 upregulated genes identified in our study were also identified as upregulated genes by Castoe et al. (2013) [12]. However, there was less overlap for the heart and small intestine. These differences may be due to the use of different quantification methods for gene expression in the 2 studies, but they may also be a result of the limited biological replicates in our study. Nevertheless, genes identified as being upregulated in both studies can be referred to with high confidence.

### Physiological interpretation of the upregulated genes in the stomach

The considerable changes in gene expression in the stomach were reflected in a pronounced rise in expression of ribosomal 40S and 60S proteins (Fig. 4), which is likely to have attended the rise in protein synthesis required for the marked transition from a quiescent fasting state to the activated digestive state. This is also supported by the presence of ribosomal functions in the enriched GO analysis of the highly expressed genes in the stomach (Supplementary Fig. S4B). During fasting, gastric acid secretion, and presumably also the secretion of digestive enzymes and lysozymes, is halted, such that the gastric fluid has a neutral pH, whilst ingestion of prey is followed by an immediate activation of gastric acid secretion [38, 39]. The stimulation of the secretory actions of the stomach is attended by an increased mass of the stomach, where particularly the mucosa expands within the first 24 hours [40].

**Figure 10:**
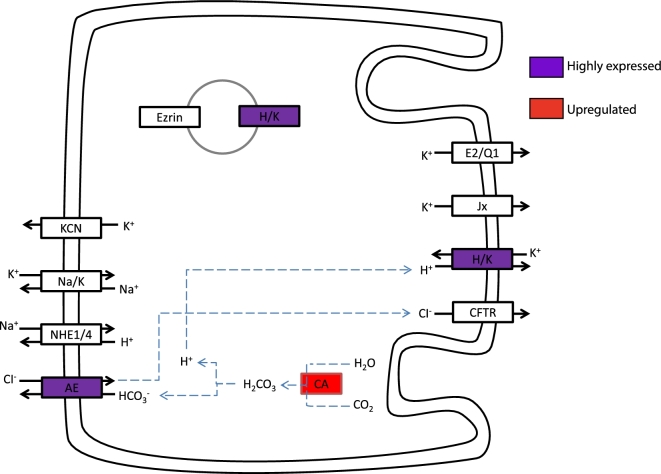
Cartoon depiction of coloured KEGG pathway of gastric acid secretion in the stomach. The entry in red represents upregulation during digestion; the entry in purple represents high expression. H/K is H+/K+-exchanging ATPase alpha polypeptide. CA is carbonic anhydrase. AE is solute carrier family 26 (anion exchange transporter).

The KEGG analysis, however, shows that the genes encoding for the gastric H, K ATPase, the active and ATP-consuming ion transporter responsible for gastric acid secretion, are highly expressed in fasting animals and are not additionally elevated in the postprandial period (Fig. 10). This strongly indicates that the enzymatic machinery for gastric acid secretion is maintained during fasting, a trait that may enable fast activation of acid secretion, at modest energetic expenditure, to kill bacteria and match gastric pH to the optimum value for pepsin. This interpretation is consistent with a number of recent studies indicating a rather modest contribution of gastric acid secretion to the specific dynamic action (SDA) response in pythons [41, 42], but we also did observe a high prevalence of ATP synthase subunits (Fig. 4) amongst the highly upregulated genes, which does indicate a rise in aerobic metabolism (see also Supplementary Fig. S4). Furthermore, the upregulation of the gene encoding for creatine kinase (Fig. 4) indicates that increased capacity for aerobic respiration required costs of acid secretion and the stimulation of the accompanying gastric functions. It has been proposed that gastric processes account for more than half of the rise in total metabolism during digestion [38], and aerobic metabolism of isolated gastric strips *in vitro* increased during digestion [43]. However, while metabolism of the stomach certainly must increase during the postprandial period, more recent studies indicate a considerably smaller contribution of gastric acid secretion to the total SDA response, meaning that gastric acid comprises considerably less than 50% of the SDA [41, 42, 44].

Our KEGG analysis also showed a large rise in the expression of the gene encoding for carbonic anhydrase (Fig. 10), the enzyme that hydrates CO_2_ and provides protons for gastric acid secretion. Gastric acid secretion, therefore, does not appear to undergo transcriptional regulation, but it is likely to involve translocation of existing H, K ATPases in vesicles from intracellular vacuoles to the apical membrane of the oxyntopeptic cells that are responsible for both gastric acid secretion and the release of pepsinogen in reptiles [45]. An activation of the processes involved in vesicle transport is further supported by increased transcription of the gene encoding for CD63 (Fig. 4), which belongs to the tetraspanin family and mediates signal transduction events.

In contrast to acid secretion, expression of several genes encoding digestive enzymes (embryonic pepsinogen-like, gastricsin precursor, and gastricsin-like peptides) (Fig. 4) were upregulated, which is consistent with *de novo* synthesis of the enzymes responsible for gastric protein degradation. Also, there was good overlap between the upregulation of the relevant genes encoding the proteins identified in the stomach secretome, such as gastrokines, pepsin homologs, phospholipases, and gastric intrinsic factor (Supplementary Table S8). In this context, it is also interesting that mucin 6 (Fig. 4), the gene coding for the large glycoprotein (gastric mucin) that protects the gastric mucosa from the acidic and proteolytically active chyme in the stomach lumen, was upregulated. Thus, as gastric acid secretion is activated, probably in response to increased levels of the gastrin as well as luminal factors, there is an accompanying activation of the protective mucus layer that prevents auto-digestion of the gastric mucosa. It is also noteworthy that the genes for both gastrokine 1 and 2 were upregulated during digestion (Fig. 4). Gastrokines are constitutively produced proteins in the gastric mucosa in mammals and chickens, and while their physiological function remains somewhat elusive, they appear to be upregulated during mucosal remodelling in response to inflammation (e.g., in connection with ulcers) and often downregulated in cancers. Thus, it is likely that the gastrokines are involved in regulating the restructuring of the mucosa during digestion in pythons.

In addition to analysing the gene expression profiles of the stomach, we also used a proteomics approach, assisted by our python transcriptome sequence database, to identify the hydrolytic enzymes in the gastric juice secreted during digestion. We identified python proteins on a complex background of highly abundant mice proteins. Thus, the digestive enzymes secreted by the pancreas are probably functionally similar to known hydrolytic enzymes from other species.

We hypothesized that relatively aggressive proteolytic digestive enzymes in the gastric juice facilitate digestion of large and un-masticated whole prey items [8]. In our analysis, 6 out of the 7 identified proteolytic enzymes were pepsinogen homologs (Peptidase subfamily A1A), and these were also the most abundant hydrolytic enzymes in the gastric juice according to the emPAI values (Supplementary Table S8). It is likely that other pepsinogen isoforms exist in the gastric juice as our approach predominantly targeted the most abundant proteolytic enzymes. The importance of the proteomics-identified pepsinogens was also substantiated by the transcriptomics data (Supplementary Table S9). Here, we found that the 6 different pepsinogens were upregulated between 2.2- and 22.2-fold from the fasting animals to 48 hours after ingestion of mice. On average, the pepsinogen transcripts were upregulated 10.7-fold. It supports that these proteases play a substantial role in the aggressive digestion process performed by the python.

Our proteomic analysis also suggested the identification of the pepsinogens as the major digestive proteolytic enzymes, similar to all other vertebrate species. Thus, our results indicate that the pepsinogen is not unique (with respect to protease class) and that hitherto uncharacterized proteases do not facilitate the aggressive digestion process. Instead, pepsins, homologous to pepsins among other species, digest the intact swallowed prey. As in other vertebrates, pythons have a low gastric pH during digestion [38, 42], and it is likely that these pepsins variants are among the most effective and aggressive pepsins identified so far; our sequence information facilitate future cloning, expression, and characterization of these potentially industrial relevant enzymes.

### Physiological interpretation of the upregulated genes in the intestine

The small intestine of pythons undergoes a remarkable and fast expansion during digestion, where both wet and dry mass more than double within the first 24 hours. The expansion stems primarily from increased mucosal mass, achieved by swelling of the individual enterocytes [46], while the smooth muscle in the gut wall is much less responsive [47]. Earlier studies on gene expression profiles during digestion in the python intestine revealed massive upregulation of more than 1 thousand genes, commencing within the first 6 hours after ingestion [12, 13]. Importantly, this previous study [13] identified a number of genes that are likely to be involved in the restructuring of the microvilli, cell division, and apoptosis, as well as brush-border transporter proteins. In line with these earlier findings, our GO enrichment analysis also highlights functions pertaining to mitotic cell division, which supports a contribution to growth by hyperplasia faster cell turnover (Supplementary Fig. S5). The expansion of the individual enterocytes is accompanied by pronounced elongation of the microvilli [48], and the resulting rise in surface area of the intestinal lining is accompanied by a 10-fold increase in intestinal transport capacity for amino acids and other nutrients [1, 4, 49].

Earlier studies provided strong evidence for an upregulation of genes coding for nutrient transporter proteins, such as D-glucose, L-proline, and L-leucine [13]. In this context, it is noteworthy that there were no nutrient transporters amongst the highly expressed and upregulated genes in the intestine (Fig. 5), but our KEGG analysis nevertheless showed increased expression of the serosal L-type amino acid transporter. Clearly, it would be worthwhile to quantitatively analyse the extent to which *de novo* synthesis of the various nutrient transporters, particularly those for amino acids, is increased during digestion and how much such synthesis contributes to absorptive capacity. It would seem adaptive if many of the transporters merely have to be activated, either by insertion within the luminal membrane or exposed as the enterocytes expand: an energetically cheap manner of matching intestinal performance to the sudden appearance of nutrients in the intestine after a meal. The GO enrichment analysis also pointed to an enrichment of various metabolic processes during digestion, particularly for the upregulated genes (Supplementary Fig. S5). It is noteworthy that the expression of genes coding for glutathione S-transferase, peroxiredoxin, and selenoprotein increased during digestion (Fig. 5). These 3 proteins are involved in cellular defence, particularly as antioxidants are a likely protection from the reactive oxygen species that result from increased aerobic metabolism.

There is consensus that the anatomical and structural responses underlying this phenotypic flexibility of intestinal function occur at modest energetic expenditure [17, 38, 50], but our expression profile does show increased expression of the gene coding for cytochrome P450, pointing to increased aerobic and mitochondrial metabolism. An increased expression of the genes involved in oxidative phosphorylation was also reported in earlier studies on pythons [12, 13]. This rise in metabolism may be driven primarily by the massive rise in secondary active transport to absorb the amino acids and smaller peptides rather than the structural changes [50]. Nevertheless, the structural changes may be reflected in increased expression of galectin 1 (Fig. 5), which mediates numerous functions including cell–cell interactions, cell–matrix adhesion, and transmembrane signalling.

Figure 5 reveals the importance of lipid absorption and its subsequent transport by the cardiovascular and lymph systems, and it is also possible that several of the expressed proteins play a role in the incorporation of lipid droplets within the enterocytes. Thus, the presence of numerous apolipoproteins, and their precursor apoe protein, amongst the list of highly expressed and highly expressed genes (Fig. 5) is probably required to transport the absorbed lipids in plasma and lymph, but the apolipoproteins could also act as enzyme cofactors, receptor ligands, and lipid transfer carriers in the regulation of lipoprotein metabolism and cellular uptake. The presence of diazepam-binding inhibitor (Fig. 5), a protein involved in lipid metabolism and under hormonal regulation mostly within nervous tissue, is also likely to reflect the increased lipid absorption and metabolism in the postprandial period, and there was also a rise in phospholipases (Fig. 5) that are likely to be involved in lipid degradation. Also, the capacity for protein metabolism clearly increased in the intestine during digestion (seen in, e.g., meprin A and endopeptidase that cleave peptides, as well as 4-aminobutyrate aminotransferase, 4-trimethylaminobutyraldehyde dehydrogenase, and diamine acetyltransferase), and there was a rise in the ammonium transporter protein Rh (Fig. 5). Finally, a number of proteins involved in calcium uptake and metabolism, such as calbindin and calmodulin (Fig. 5), could be important to handle the break-down of the bone in a normal rodent, and it was recently shown that the enterocytes of pythons already contain small particles of bone at 24 hours after ingestion [48].

### Physiological interpretation of the upregulated genes in the heart

The large metabolic response to digestion is accompanied by a doubling of heart rate and stroke volume of the heart, such that cardiac output remains elevated for many days during digestion [51, 52]. This cardiovascular response plays a pivotal role in securing adequate oxygen delivery to the various organs and serves to ensure an appropriate convective transport of the nutrients taken up by the intestine. The tachycardia is mediated by a release of vagal tone and the presence of a non-adrenergic, non-cholinergic factor that stimulates the heart and that has been speculated to be released from the gastrointestinal organs during digestion [53, 54]. The increased heart rate and the rise in the volume of blood pumped with each beat must be supported by increased myocardial metabolism, and we observed an upregulation of malate dehydrogenase, cytochromes, and ATPase-linked enzymes (Fig. 6) that are likely to be related to an increased oxidative phosphorylation within the individual myocytes (see also the prevalence of enriched GO terms associated with aerobic metabolism in Supplementary Fig. S8). Previous gene expression studies on the python heart also yielded evidence for its increased oxidative capacity in the postprandial period [55] and that cytochrome oxidase activity is almost doubled during digestion [56]. We confirm that transcription for heat shock proteins may be increased [55], possibly to protect against oxidative damage as result of the increased metabolism. As in earlier studies [55], our observation of increased ATP synthase lipid−binding protein and fatty acid binding protein 3 (Fig. 6) provide evidence for increased fatty acid metabolism, which may reflect the substantial rise in circulating fatty acids in the plasma.

It was originally suggested that the postprandial rise in stroke volume could be ascribed to an impressive and swift growth of the heart [10], possibly triggered by lipid signalling [55]. However, a number of recent studies, primarily from our laboratory, have shown that increased cardiac mass is not an obligatory postprandial response amongst pythons [56–58] and that stroke volume may be increased in response to increased venous return rather than cardiac hypertrophy [56]. It is nevertheless noteworthy that our study and the previous studies show a clear increase in the expression of contractile proteins (e.g., myosin and actin) as well as tubulin (Fig. 6), which may reflect increased protein turnover in response to increased myocardial workload rather than cell proliferation or hypertrophy. The enriched GO analyses also point to major changes in the extracellular space, as well as both elastin and collagen, which may indicate some level of cardiac reorganization at the cellular or subcellular level that may alter compliance of the myocardial wall and influence cardiac filling (Supplementary Fig. S8). It is noteworthy that the increased expression of brain natriuretic peptide (BNP) may serve a signalling function as described in response to the cardiac hypertrophy that attends hypertension.

### Physiological interpretation of the genes in the liver

The liver exhibited a diverse expression profile in response to digestion that is likely to reflect its many metabolic functions in connection with metabolism, synthesis, and detoxification during the postprandial period. This pattern is also evident from the many metabolic functions identified in the enriched GO analysis (Supplementary Fig. S7). There were marked upregulations of the P450 system (Fig. 7), which fits well with a rise in synthesis and breakdown of hormones and signalling molecules, cholesterol synthesis in response to lipid absorption, and possibly also an increased metabolism of potentially toxic compounds in the prey. A rise in cholesterol metabolism was supported by increased expression apolipoproteins (Fig. 7). The hepatic involvement in lipid metabolism was also supported by the increased expression of genes for Alpha-2-macroglobulin and serum albumin (Fig. 7). The increased expression of albumin obviously also corresponds nicely with the proteomic analysis of plasma proteins, and it is likely that the postprandial rise in plasma albumin serves a functional role in the lipid transport between the intestine and the liver, as well as other metabolically active organs.

It is also noteworthy that a number of genes associated with the protection of oxidative stress, such as catalase, heat shock protein, and glutathionine transferase, were markedly upregulated (Fig. 7). It was recently argued that snakes digesting large meals experience oxidative damage due to reactive oxygen metabolites requiring increased antioxidant responses to protect cellular functions [59].

### Physiological interpretation of the genes in the pancreas

We sampled the entire pancreas for our analysis of gene expression, and our data therefore reflect both endocrine and exocrine pancreatic functions. We found ample evidence for upregulated expression of genes associated with the digestive functions, such as lipases, trypsin, chymotrypsin, and elastase, as well as other enzymes for the digestion of protein and lipid (Fig. 8). This general upregulation of secretory processes is likely to explain the prevalence of processes associated with protein synthesis in the enriched GO analysis (Supplementary Fig. S6). There was even an increased expression of amylase (Fig. 8), which breaks down polysaccharides. In connection with this latter function, the increased expression of insulin (Fig. 8) from the endocrine pancreas is likely to reflect increased cellular signalling for the postprandial uptake of both glucose and amino acids. As in the other organs, we found increased expression of cytochrome oxidase (Fig. 8) to be indicative of increased metabolism during digestion, and the rise in heat shock protein expression may reflect a response to formation of reactive oxygen species as metabolism is stimulated by increased secretion of the pancreas.

## Conclusions

Our study confirms that the extensive physiological and anatomical reorganization of the visceral organs of pythons during the postprandial period is driven by differential expression of hundreds or even thousands of genes. Many of the upregulated functions pertain to energy production to support the rise in aerobic metabolism associated with the digestion and absorption of large meals. In terms of the gastrointestinal organs, the gene expression profiles also support the view that many of the digestive functions, such as gastric acid secretion and nutrient absorption, can be stimulated with little change to gene expression, indicating that the proteins involved in these processes merely need to be activated during the postprandial period, and thus avoiding the energy and time-consuming processes associated with *de novo* synthesis. This digestive strategy may, at least in part, explain how intermittent feeders, such as snakes, retain the capacity for rapid and reliable upregulation of the digestive processes immediately after prey ingestion.

## Methods

### Stimulation of the postprandial response, collection of tissue biopsies, and purification of RNA for mRNA-Seq analyses

Six *Python bivittatus* (tiger python/Burmese python) with body masses ranging from 180 to 700 g (average 373 g) were obtained from a commercial supplier and housed *in vivaria* with a heating system providing temperatures of 25–32°C. The animals were fed rodents once a week, and fresh water was always available. The animals appeared healthy, and all experiments were performed according to Danish Federal Regulations. All 6 individuals were fasted for 1 month and divided in 3 groups. Four animals were fed a rodent meal of 25% of their body weight and euthanized with an intra-peritoneal injection of pentobarbital (50 mg kg^−1^; Mebumal) at 24 hours (*n* = 2) or 48 hours after feeding (*n* = 2). The remaining 2 snakes served as fasted controls. During deep anaesthesia, 2 biopsies were obtained from each snake from each of the following tissues: heart (ventricles), liver, stomach, intestine, and pancreas. In regard to the stomach tissue samples, 1 sample was obtained from the proximal part of the stomach, and 1 sample was obtained from the distal part. In total, 60 biopsies were collected. The samples were taken from the same part of the different tissues in all individuals. After sampling, the biopsies were weighed and immediately snap-frozen in liquid nitrogen; stomach and intestinal tissues were rinsed in sterile saline solution before weighting to avoid contamination with rodent tissue from the ingested meal. Subsequently, all 60 biopsies were homogenized in liquid nitrogen, and the 4 biological replicates (2 biopsies from each individual) were pooled in a 1:1 manner based on mass. This resulted in 15 samples (5 tissues × 3 time points). From these samples, total RNA was purified using the Nucleospin RNA II kit (Machery-Nagel GmbH & Co. Dueren, North Rhine-Westphalia, Germany), as recommended by the manufacturer. The RNA concentration and quality were assessed by Nanodrop ND 1000 Spectrophotometer (Thermo Scientific) analyses, agarose gel-electrophoreses, and Agilent BioAnalyzer (Agilent) analyses.

### Library production and sequencing

Poly-A transcripts were enriched and the transcripts broken in the presence of Zn^2+^. Subsequently, double-stranded cDNA was synthesized using random primers and RNase H. After end repair and purification, the fragments were ligated with bar-coded paired-end adapters, and fragments with insert sizes of approximately 150–250 bp were isolated from an agarose gel. The 15 samples were derived from 5 tissues (heart, liver, stomach, pancreas, and intestine) at the 3 time points (fasted for 1 month, 24 hours, and 48 hours post-feeding) and were amplified by polymerase chain reaction (PCR) to generate DNA colony template libraries, and the libraries were then purified. In addition, to sample as broadly from each transcriptome as possible, we also produced normalized libraries for each tissue in order to capture the reads from lowly expressed, tissue-specific genes. Here, part of the samples, which originated from the same tissue, was pooled before the PCR analyses; i.e., in total 5 pooled samples were generated. These 5 samples were split in 2, and after PCR amplification and library purification, they were normalized using 2 different normalization protocols; i.e., in total, 10 normalized libraries were prepared. The library quality of all 25 samples was then assessed by a titration run (1 × 50 bp) on an Illumina HiSeq 2000 instrument. Finally, the sequencing was performed on the same instrument using paired reads (2 × 101 bp). One channel was used for the 15 non-normalized libraries, and 1 channel was used for the 10 normalized libraries.

### Data pre-processing and *de novo* transcriptome assembly

To reduce the amount of erroneous data, the raw paired reads were processed by (i) removing reads that contained the sequencing adaptor, (ii) removing reads that contained ambiguous characters (Ns), and (iii) trimming bases that had low average quality (Q < 20) within a sliding window length of 10.

To develop a comprehensive transcriptomics resource for the Burmese python, all high-quality reads from 25 libraries were pooled together for *de novo* assembly. To determine the optimal assembly, *de novo* assembly was performed using Velvet (v. 1.2.03; Velvet, RRID:SCR_010755) [18] and Oases (v. 0.2.06; Oases, RRID:SCR_011896) [60] with different k-mer parameters. The performance of these assemblies was assessed according to number of transcripts, total length of transcripts, N50 length, mean length, proportion of mapped reads, and number of transcripts whose length was larger than N50 (Supplementary Table S2).

### Assessment of the transcriptome assembly

The transcriptome assembly was evaluated by rnaQUAST 1.4.0, with default parameters supplying the reference genome sequences and genome annotation of the Burmese python (GenBank assembly accession: GCA_000186305.2).

BUSCO_v2 (BUSCO, RRID:SCR_015008) [20] was used to test the completeness of transcriptome assembly with dependencies NCBI BLAST+ 2.4.0 [61] and HMMER 3.1b2 (Hmmer, RRID:SCR_005305) [62]. The vertebrata lineage set was used and accessed on 28 November 2016.

### Transcriptome annotation

To assess the identity of the most closely related gene in other organisms, the assembled transcripts were compared with the sequences in the NCBI non-redundant protein database using blastx (BLASTX, RRID:SCR_001653) [63] with an e-value cut-off of 0.01. The nr annotation term of each transcript was assigned with the first best hit, which was not represented in uninformative description (e.g., “hypothetical protein,” “novel protein,” “unnamed protein product,” “predicted protein,” or “uncharacterized protein”) (Supplementary Table S4). To assign functional annotations of transcripts, Blast2GO was used (e-value threshold = 0.01) to return GO annotation, enzyme code annotation with KEGG maps, and InterPro annotation.

### Estimation of gene expression values

For each of 15 non-normalized libraries, the paired-end reads were first mapped back to the assembled transcriptome using Bowtie2 (Bowtie, RRID:SCR_005476) [64] with default parameters; the raw counts then were calculated based on the alignment results using RSEM (v. 1.1.20) [65] for each transcript. To quantify the gene expression level, for genes with alternative splicing transcripts, the longest transcript was selected to represent the gene, and a gene's abundance estimate was the sum of its transcripts’ abundance estimates. Finally, the raw expression counts were normalized into FPKM with custom Perl scripts.

### PCA

To facilitate graphical interpretation of tissue relatedness, R function prcomp was used to perform PCA with genes where the maximum FPKM of 15 samples was greater than 100.

### Identification of DEGs and clustering analysis

For each tissue, DEGs were selected with 2 thresholds: (i) FPKM is greater than or equal to 400 in at least 1 time point and (ii) FC is greater than or equal to 2 in at least 1 pairwise comparison among 3 time points. FPKM values of DEGs were log2-transformed and median-centred, then hierarchical clustering was performed using R command hclust with method = “average” and distance = “Spearman correlation.” Results were displayed using R command heatmap.2.

### Coloured KEGG Pathway and GO enrichment analysis

For each tissue, all assembled genes were mapped to KEGG human pathway maps using KOBAS 2.0 [66] with an e-value of 1e-50. Then genes were coloured to represent FPKM value and trend of differential expression value (Table 2).

Blast2GO was used to implement GO enrichment analysis (Fisher's exact test) with a threshold false discovery rate of 0.001. The reference set is the whole transcripts with GO slim annotation. For each organ, the selected test set is either upregulated or highly expressed genes, defined in Table 2. Finally, we performed Blast2GO to reduce to most specific GO terms.

### Isolation of samples for proteomics analyses

Two Burmese pythons (weighing 400 and 800 g, respectively) were fed a rodent meal corresponding to approximately 25% of their body mass. Approximately 24 hours into the postprandial period, the animals were euthanized with an overdose of pentobarbital (100 mg kg^−1^, i.m.). Immediately afterwards, an incision was made to expose the stomach, which was then ligated at the lower oesophagus and the pylorus, before the intact stomach was excised by a cleavage just below the 2 sutures, resulting in the stomach being released from the rest of the animal. All undigested mouse remains were manually removed by forceps, and 25 ml/kg tris-buffered saline (TBS) was injected into the stomach. The stomach was then ligated at the opened end, rinsed by gently shaking the tissue, and finally the digestive fluid-containing solution was collected and stored on ice. To ensure collection of all gastric fluid, the stomach was rinsed an additional 2 to 3 times with 12 ml/kg TBS. Subsequently, the samples were filtered and centrifuged, and the supernatant stored at –80°C. We also obtained 2 samples of gastric juice from a third individual (200 g) that had been fed 4 g peptone (Sigma Aldrich), suspended in water. Peptone is a mixture of small peptides and amino acids, and the solution was injected directly into the stomach; after 3 hours, the snake was euthanized by an overdose of pentobarbital. The stomach was removed and rinsed with TBS, and a single sample was collected and stored, as described above. We analysed 2 samples from each of the 3 individuals, resulting in a total of 6 digestive fluid samples being analysed by MS/MS. In addition, we obtained a single plasma sample from each snake by direct cardiac puncture, followed by centrifugation and storage for later analysis.

### Sample preparation for mass spectrometry analyses

The proteins in the 6 obtained python digestive fluid samples were recovered by trichloroacetic acid precipitation. The resulting pellets were resuspended in 8 M Urea, 5 mM Dithiothreitol (DTT), and 0.1 M ammonium bicarbonate pH 8.0 and incubated for 30 minutes at room temperature in order to denature and reduce the proteins. Subsequently, the proteins were alkylated by the addition of iodoacetamide to a final concentration of 25 mM. The samples were incubated for an additional 20 minutes at room temperature and then diluted 5 times with a 50-mM ammonium bicarbonate, pH 8.0 buffer before the addition of approximately 2 μg sequencing grade modified trypsin (Promega) per 50-μg protein in the sample. Subsequently, the samples were incubated at 37°C for approximately 16 hours. The proteins in the plasma sample were denatured, reduced, alkylated, and digested with trypsin, as described for the digestive fluid samples. Finally, the resulting peptides in all samples were micropurified and stored at –20ºC until the LC-MS/MS analyses.

### Liquid chromatography/tandem mass spectrometry analyses

LC-MS/MS analyses were performed on a nanoflow HPLC system (Thermo Scientific, EASY-nLC II) connected to a mass spectrometer (TripleTOF 5600, AB Sciex) equipped with an electrospray ionization source (NanoSpray III, AB Sciex) and operated under Analyst TF 1.6 control. The samples were dissolved in 0.1% formic acid, injected, trapped, and desalted isocratically on a precolumn, whereupon the peptides were eluted and separated on an analytical column (16 cm × 75 μm, i.d.) packed in-house with ReproSil-Pur C18-AQ 3 μm resin (Dr. Marisch GmbH). The peptides were eluted at a flow rate of 250 nL/min using a 50-minute gradient from 5% to 35% phase B (0.1% formic acid and 90% acetonitrile). An information-dependent acquisition method was employed, allowing up to 25 MS/MS spectra per cycle of 2.8 seconds.

### Protein identification and filtering of data

The 6 collected MS files related to digested fluid were converted to mascot generic format (MGF) using the AB SCIEX MS Data Converter beta 1.3 (AB SCIEX) and the “proteinpilot MGF” parameter. Subsequently, the files were merged to a single MGF file using Mascot daemon. The resulting file (encompassing 122538 MS/MS queries) was used to interrogate the 16693 *Mus musculus* sequences in the Swiss-Prot database (v.2015_10) and the generated python database (encompassing 21131 protein sequences) respectively using Mascot 2.5.0 (Matrix Science) [67]. Trypsin, with up to 1 missed cleavage allowed, was selected as enzyme; carbamidomethyl was employed as fixed modification, and oxidation of methionine and proline was selected as variable modification. The instrument setting was specified as ESI-QUAD-TOF, the mass accuracy rates of the precursor and product ions were 15 ppm and 0.2 da, respectively, and the significance threshold (*P*) was set to 0.01, with an expected cut-off at 0.005. The data obtained by the LC-MS/MS analysis of the python plasma proteome were analysed as described for digestive fluid samples, except that the *Mus musculus* sequences were not interrogated. This dataset contains 9224 MS/MS queries. All obtained results were subsequently parsed using MS Data Miner v. 1.3.0 [68], and protein hits were only accepted if they were identified based on 2 unique peptides. Semi-quantitative proteomics data were obtained using the emPAI-values given by the Mascot 2.5.0 software after analysis of the MS/MS data [69].

To identify the proteins secreted into the python stomach, the identified python plasma and the mouse protein homologs were removed from the list of identified python digestive fluid proteins. With regard to the removal of prey protein homologs, the overall mouse protein names were used to search the list of python proteins (e.g., “collagen” was used as search term, not “collagen alpha-1(I) chain”) and to identify python proteins that were identified based on homology with mice. These proteins were removed from the list of stomach-secreted python proteins. For each identified protein remaining on the list, we reassessed the annotation of the python sequence; i.e., sequence comparisons were performed using blastp v. 2.2.30, and in addition, the UniProt and NCBI protein databases, as well as PubMed and SignalP 4.1, were interrogated to identify the functional properties and cellular locations of the identified proteins. Plasma proteins, remaining collagen homologues, intracellular proteins, and membrane proteins were discarded from the list of identified python stomach secretome proteins.

The mass spectrometry proteomics data have been deposited in the ProteomeXchange Consortium via the PRIDE [70] partner repository, with the dataset identifier PXD006665.

## Availability of data and materials

The raw RNA-Seq sequencing data that support the findings of this study have been deposited in the NCBI BioProject database (accession No. PRJNA343735; https://www.ncbi.nlm.nih.gov/bioproject/PRJNA343735).

The mass spectrometry proteomics data have been deposited to the ProteomeXchange Consortium via the PRIDE [70] partner repository with the dataset identifier PXD006665.

Supporting data are also available from the *GigaScience* repository, *Giga*DB [71].

## Abbreviations

BUSCO: Benchmarking Universal Single-Copy Orthologs; DEG: differentially expressed genes; FC: fold change; FPKM: fragments per kilo base per million sequenced reads; GO: gene ontology; LC-MS/MS: liquid chromatography/tandem mass spectrometry; MGF: mascot generic format; PCA: principal component analysis; PCR: polymerase chain reaction; SDA: specific dynamic action; TBS: tris-buffered saline.

## Competing interests

The authors declare that they have no competing interests.

## Funding

This work was supported by a grant (Novenia) from the Danish Research Council for Strategic Research (grant identification number: 09-067076).

## Author contributions

J.D., K.W.S., T.W., and M.H.S. designed the study. J.D. performed the transcriptome data analysis with input from L.S. and was a major contributor in writing the manuscript. S.E.L. performed RNA-Seq lab experiments. K.W.S. and J.E. performed the proteomics experiment and data analysis. W.T. interpreted the transcriptome data regarding digestion. All authors read and approved the final manuscript.

## Supplementary Material

GIGA-D-16-00152_Original-Submission.pdf

GIGA-D-16-00152_Revision-1.pdf

GIGA-D-16-00152_Revision-2.pdf

Response-to-Reviewer-Comments_Original-Submission.pdf

Response-to-Reviewer-Comments_Revision-1.pdf

Reviewer-1-Report-(Original-Submission).pdf

Reviewer-1-Report-(Revision-1).pdf

Reviewer-1_Revision-1-(Attachment).pdf

Reviewer-2-Report-(Original-Submission).pdf

Reviewer-2-Report-(Revision-1).pdf

Supplement Materials
