# Supplementary material for: Transcriptome analysis of the response of Burmese python to digestion
Source: Gigascience. 2017 Jul 13;6(8):1–18. doi: 10.1093/gigascience/gix057 (PMC5597892; doi:10.1093/gigascience/gix057)
Supplement: GIGA-D-16-00152_Original-Submission.pdf [file gix057_GIGA-D-16-00152_Original-Submission.pdf]

Manuscript
Click here to download Manuscript Burmese python_withBUSCO.docx

Transcriptome Analysis of the Response of Burmese Python to Digestion

Jinjie Duan[1], Kristian Wejse Sanggaard[2,3], Leif Schauser[5], Sanne Enok Lauridsen[4], Jan J. Enghild[2,3], Mikkel Heide Schierup[1,4] and Tobias Wang[4]

1. Bioinformatics Research Center, Aarhus University, Denmark
2. Department of Molecular Biology and Genetics, Aarhus University, Denmark
3. Interdisciplinary Nanoscience Center (iNANO), Aarhus University, Denmark
4. Department of Bioscience, Aarhus University, Denmark
5. QIAGEN Aarhus, Silkeborgvej 2, 8000 Aarhus C, Denmark

Corresponding authors: Jinjie Duan, Mikkel Heide Schierup & Tobias Wang

Email address:
Jinjie Duan: jjduan@birc.au.dk
Kristian Wejse Sanggaard: kristian@wejsesanggaard.dk
Leif Schauser: Leif.Schauser@qiagen.com
Sanne Enok Lauridsen: sanne.lauridsen@inano.au.dk
Jan J. Enghild: jje@mbg.au.dk
Mikkel Heide Schierup: mheide@birc.au.dk
Tobias Wang: tobias.wang@bios.au.dk

Abstract

Background:

The exceptional and extreme feeding behaviour makes the Burmese python a unique and interesting model to study physiological remodelling and metabolic adaptation in response to feeding after prolonged starvation. With outset in specific hypotheses based on in vivo physiological responses, we use transcriptome sequencing of five visceral organs and three digestive stages to unravel the patterns of changes in the gene expression of Burmese python upon ingestion of a large meal. We first used the combined data to perform a *de novo* assembly of the transcriptome. We supplemented with a proteomic survey of enzymes in the gastric juice, stomach secretome and plasma during digestion assisted by our transcriptome sequence database.

Results:

We constructed a high quality transcriptome with 34,423 transcripts of which 19,713 (57%) were annotated. Among highly expressed genes (FPKM>100 in one tissue) we found differential expression for 43 genes in heart, 206 genes in liver, 114 genes in stomach, 89 genes in pancreas and 158 genes in intestine. We interrogated the function of these genes in order to test previous hypothesis on the response to feeding. We also used the transcriptome to identify 314 secreted proteins in the python gastric juice.

Conclusions:

We provide comprehensive transcriptome data of multiple organs and various digestive time of Burmese python and address specific hypothesis on certain pathways known to related digestion process. We also identify, for the first time, stomach-related proteins from a

digesting individual and thereby demonstrate that the sensitivity of modern LC-MS/MS equipment allows the identification of gastric juice proteins that are present during digestion thereby providing novel insight into the digestion mechanism.

Keywords:

Burmese Python, transcriptome, tissue expression, digestion, pathway, proteome

Background

All animals exhibit dynamic changes in the size and functional capacities of bodily organs and tissues to match energetic maintenance costs to the prevailing physiological demands [1]. This phenotypic flexibility is particularly pronounced for the digestive organs in animals that naturally experience prolonged periods of fasting and ingest large prey items at irregular intervals. The Burmese python is one such model system for studying extreme phenotypes [1]. Many species of pythons easily endure months of fasting and subdue and ingest very large meals. In Burmese pythons, digestion is attended by a large and rapid increase in the mass and/or functional capacities of the intestine, stomach, heart and kidneys [2-4] in combination with a stimulation of secretory processes and an activation of enzymes and transporter proteins. These physiological responses of organ functions are associated with a many-fold rise in aerobic metabolism. Hence, the Burmese python is an excellent model to study the mechanisms underlying extreme metabolic transitions and physiological remodelling in response to altered demand [1, 3, 5-10].

The postprandial changes in the morphology and physiology of the intestine, heart and other organs have been described in some detail in pythons [1, 5, 8, 9, 11], but only a few studies [12-14] have addressed the underlying transcriptional changes of this interesting biological response. Transcriptome sequencing technology now allows comprehensive surveys [15, 16], and we therefore decided to use transcriptome sequencing of heart, liver, stomach, pancreas and intestine in snakes that had fasted for one month and at 24 and 48h into the postptrandial period. As the Burmese python reference genome assembly [12] currently is relatively fragmented (contig size N50 ~10kb), we found it impractical to use re-sequencing approaches and opted instead to use our high coverage data to build a *de novo* transcriptome assembly to identify differentially expressed genes (DEGs). To identify the enzymes involved in the

digestion process, we initiated digestion, then isolated the digestive fluid and characterized the protein composition using a proteomics-based approached. This also allowed us to identify the major hydrolytic enzymes used to digest the large and un-masticated meals.

Analyses

Data summary

277,485,924 raw paired reads (2*101 bp, insert size 180 bp) were obtained from Illumina Hi-Seq 2000 sequencing of 15 non-normalized cDNA libraries derived from 5 tissues (heart, liver, stomach, pancreas and intestine) at 3 time points (fasted for 1 month, 24h and 48h post-feeding) and 10 DSN-normalized cDNA libraries (see methods) (Supplementary Table S1). After removal of low-quality reads (See methods), 213,806,111 (77%) high-quality paired reads were retained. These reads contained a total 43,146,073,200 bp nucleotides with a mean Phred quality score of >= 37 (Q37). To develop a comprehensive transcriptomics resource for the Burmese python (Fig. 1), we pooled these high quality reads from 25 libraries for subsequent *de novo* assembly.

de novo transcriptome assembly and gene annotation

As short k-mers have a higher propensity to generate misassembled transcripts when using a de Bruijn graph-based *de novo* assembler, such as Velvet [17], we conservatively chose an assembly generated using long k-mers for subsequent analysis, at the cost of some sensitivity regarding assembled isoforms. Thus, balancing key metrics (Supplementary Table S2), we used an assembly based on the longest k-mer = 95 (Table 1), as it had the fewest scaffolds/transcripts (34,423), but represented a very large proportion (74%) of all reads. The scaffold N50 of this assembly was 1,673 bp. To evaluate our assembly of the transcriptome, we mapped and aligned the scaffolds against the reference genome of Burmese python [12]. We found that 99.7% of transcripts mapped back to the genome assembly and that 86.2% transcripts had both a coverage percentage and an identity percentage of alignment greater than 90% (Supplementary Fig. S1). The high concordance between the *de novo* transcript

assembly and genome reference strengthened our confidence in using *de novo* assembly as our reference, and shows that the individual fragments were accurate although the reference genome assembly is fragmented. We also assess the completeness of our transcriptome assembly with the Benchmarking Universal Single-Copy Orthologs (BUSCO) strategy. Results showed 55.2% (1428 out of 2586) complete BUSCOs, 19.8% (512) fragmented BUSCOs and 25% (646) missing BUSCOs. These results are consistent with the survey [18] of assessment completeness of 28 transcriptomes from 18 vertebrates. In this survey, most of transcriptomes from species with close phylogenetic relationship to snake contain less than 50% complete BUSCOs and more than 40% missing BUSCOs. Therefore we conclude the quality of our transcriptome assembly was well acceptable.

19,713 transcripts (57% of 34,423) were annotated using transfer of blastx hit annotation against the non-redundant (nr) NCBI peptide database [19]. To assign proper annotation for each transcript, we chose the first best hit that was not represented in uninformative descriptions (Supplementary Table S3). The most closely related species with an annotated genome, *Anolis carolinensis* was able to annotate 10,704 transcripts (54% of all annotated transcripts). Burmese Python and *A. carolinensis* both belong to the reptilian Squamata order, and are separated by approximately 120 million years of evolution [20].

Blast2GO [21] then annotated these 19,713 transcripts, and 16,992 of them could be assigned by one or more GO terms and functional roles were described. The distributions of the most frequently identified GO terms categories for biological process (BP), molecular function (MF) and cellular component (CC) are shown in Fig. S2. Moreover, we used the functionality of InterPro [22] annotations in Blast2GO to retrieve domain/motif information for our transcripts, and 21,023 transcripts were annotated by the InterPro database.

The Burmese python has the highest number of albumin isoforms reported

We observed 12 genes (Supplementary Table S4) annotated with ‘serum albumin [Trimeresurus flavoviridis]’ and multiple sequence alignment of these 12 genes (Supplementary Fig. S3) suggests they are dissimilar. 6 out of 12 genes were also identified at the protein-level by a proteomics analysis (LC-MS/MS) of python plasma (Supplementary Table S5). It illustrates that the python has at least 6 copies of albumin, whereas cobra, anole, chicken have 2-3 copies and human has 4 copies on chromosome 4.

Gene expression analysis and principal component analysis

For comparisons between genes, expression profiles were obtained by mapping high quality reads to the reference transcriptome and the expression level was given by fragments per kilobase per million sequenced reads (FPKM) [23]. For the study of expression profiles, we chose to investigate 1862 highly expressed genes (FPKM >= 100 in at least one tissue of 15), as it is known that for highly expressed genes, the biological variation among biological replicates in the same tissue at the same stage is lower than for genes showing low expression levels [24]. The majority (~64%) of these 1862 genes were expressed in all tissues, and only ~18% were expressed solely in one tissue (Supplementary Fig. S4). The liver had the highest number of uniquely expressed genes, which may reflect its particular role in metabolism and excretion of waste products.

We used principal component analysis (PCA) to reveal overall differences in gene expression patterns among tissues and time points within the digestive period. The first three principal components (PCs) accounted for ~58% of the variation (Supplementary Fig. S5). Despite the large overlap in expressed genes (Supplementary Fig. S4), the different tissues exhibited distinct transcriptional signatures shown by the PCA in Figure 2, showing a tendency for 24h to represent an intermediate position between fasting and 48h. Liver, intestine and stomach displayed greater shifts in the PCA plots compared to heart and

pancreas, and the largest changes occurred between fasting and 24h in the stomach and intestine. This fits well with the expectation that the stomach and intestine respond early in digestion [3] and the observation that the liver exhibit a more dramatic change in gene expression is consistent with the previous study [12] on pythons.

Pattern of transcriptional responses to feeding

The responses to feeding involve thousands of genes and large changes in gene expression. To restrict the analysis of these many genes, we used a conservative approach where we selected genes that are both highly and differentially expressed with two strict thresholds (see methods). Application of these two thresholds yielded 43 genes for heart, 206 genes for liver, 114 genes for stomach, 89 genes for pancreas and 158 genes for intestine, respectively, that were significantly differentially expressed in response to digestion (Fig. 3). To illustrate in greater detail, we enlarged the five sub-clusters with the most prominent increase in expression. These sub-clusters, labelled a - e in Figure 3, are shown with full annotation in Figures 4-8. To unravel the functional implications of these responses, we searched for genes encoding for proteins involved in processes of tissue re-organisation, cellular metabolism and digestion within these sub-clusters for each organ.

GO enrichment analysis and colored KEGG pathway maps

To get a broader biological insight, compared to the strict threshold set used in the above clustering analysis, we applied a looser threshold set (Table 2) of defining DEG and highly expressed genes for functional annotation analysis. The summary of number of DEGs during digestion in each tissue is illustrated in Table 3. In each organ, most of genes (> 76%) have low expression (max FPKM < 10). Around 1% of the genes are highly expressed (max FPKM >= 200). The number of upregulated genes is approximately 3% in each organ, except

for the heart where only 0.57% of the genes were upregulated in response to feeding. This suggests that during digestion, the digestive organs, like liver, stomach, intestine and pancreas show more pronounced post feeding response than the heart. To dissect the functions of DEGs, we performed GO enrichment analysis with upregulated genes and highly expressed genes respectively for each organ (Figs. 9-13). As an example, the most significantly associated GO term to upregulated genes in stomach was “mitochondrial respiratory chain complex 1”, “endoplasmic reticulum membrane” and “cytosol” (Fig. 9a).

To specifically identify the pathways associated to DEGs and highly expressed genes, we mapped genes to KEGG [25, 26] human pathway maps and coloured the mapped entries with trend of gene expression during digestion (Table 2). We identified upregulated genes and highly expressed genes, respectively, involved in three selected pathways (glycolysis/gluconeogenesis, citrate cycle (TCA cycle), and oxidative phosphorylation) for each tissue (Table 4), and we performed the same identification for two main pathway categories in the KEGG pathway database (1.3 lipid metabolism and 1.5 amino acid metabolism; Table 5). The glycolysis/gluconeogenesis pathway, glyceraldehyde-3 phosphate dehydrogenase showed high expression in all organs.

Identification of the python gastric juice proteome

We identified the secretome of the python stomach during digestion (Fig. 14). The resulting mass spectrometry data (containing 122538 MS/MS spectra) was used to interrogate our python transcriptome database, which includes transcriptome from stimulated stomach tissue. In total, 549 python proteins were identified using this approach. Afterwards, all identifications based on a single tryptic peptide were removed reducing the number of identified python proteins to 314 (Supplementary Table S6).

Five classical types of pepsinogens exist, namely pepsinogen A, B, and F, progastricsin (or pepsinogen C), and prochymosin [27]. Of these, our analyses (Supplementary table S7 and S8) show that pythons primarily rely on progastricsin for proteolytic digestion, as the five most abundant proteases identified in the gastric juice are annotated as progastricsin-like. Alignment of the sequences of the various transcripts for gastricsin-like proteins shows considerable differences in sequence, which indicate the presence of numerous different proteins with similar functions. This annotation is based on accession XP_003220378.1 and XP_003220378.1 from *Anolis Carolinensis.* Alignment of the python sequences with the two anole sequences, as well as with the well-characterized human gastricsin variant, shows that both the active site residues, as well as cysteine bridges, are conserved. It demonstrates the similarity between these enzymes and suggests that the identified python sequences indeed represent catalytical active proteolytic enzymes (Fig. 15). The last identified pepsinogen-like python sequence (m.31615_Py95) was annotated based on the predicted embryonic pepsinogen-like sequence (XP_003220239.1), also from *Anolis Carolinensis.* Here, the annotation originates from an embryonic pepsinogen identified in chicken [28]. This protease was identified in the python's gastric juice with a lower emPAI value than the gastricsin sequences indicating a lower concentration of this enzyme (Supplementary table S7), although the transcript displays the highest concentration of the analyzed pepsinogens in the post-prandial period (Supplementary table S8). As the name indicate it is exclusively expressed during the embryonic period [28, 29], and phylogenic analysis of the sequence suggest that its closest homolog, among the classical pepsinogens, is prochymosin [28]. Also prochymosin, displays a temporal expression pattern and is, in mammals, mainly expressed in newborn species. However, the identified python snake embryonic chicken pepsinogen homolog does not display a similar development-related temporal expression pattern and is, as shown, used among adult species for digestion.

However, it does not exclude that the protease is expressed during the python's embryonic phase.

Identification of prey proteins and the python plasma proteome

Many of the obtained MS/MS spectra were expected to correspond to abundant mice proteins, such as collagen. To facilitate the downstream analyses of the python proteins, we produced a list of background proteins related to the prey. Hence, interrogation of the mass spectrometry data against the 16693 mouse protein sequences in the Swiss-Prot database resulted in the identification of 212 mouse proteins, after removing hits based on single peptides (Supplementary table S9). To produce a list of identified python proteins, most likely present in the digestive fluid samples due to blood contaminations during collection, we characterized the python plasma proteome. The most abundant plasma proteins are produced by the liver. Consequently, our python transcriptome sequence database, which encompasses liver transcriptomes, is expected to contain the protein sequences of the python plasma proteins. Thus, our python plasma LC-MS/MS data was used to interrogate our python sequence database. It provided an overview of the most abundant python plasma proteins (Supplementary table S10). In total, 64 plasma proteins were identified with minimum 2 tryptic peptides. The result supports the liver transcriptome data, since the abundant (based on emPAI) plasma proteins correlate with the transcripts that are detected at high concentration in the liver tissue. The overall protein composition is similar to the composition in humans with albumin, fibrinogen, alpha-2-macroglobulin, immunoglobulins, complement factors and apolipoproteins being the dominating proteins. One protein that stands out is the anti-haemorrhagic factor cHLP-B (m.27_Py95), which apparently is present in high concentration in the plasma of these snakes. This is a protease inhibitor of the haemorrhagic -causing metalloproteinases present in snake venom and these inhibitors have

previously been purified from serum of venomous snakes and thoroughly characterized [30, 31]. Our data supports older studies that identify these inhibitors of the deleterious action of venom enzymes in non-venomous snakes [32].

Identification of the python stomach secretome

To identify the python stomach secretome, the list of python proteins, identified in the digestive fluid (Supplementary table S6) was analyzed further. We assumed no overlap between abundant plasma proteins and proteins secreted by the stomach. Thus, plasma proteins, identified in the gastric juice, were assumed to be contaminations from blood and therefore the 64 identified plasma proteins were, when present, removed from the list. Subsequently, python proteins that most likely were identified based on prey proteins homology (*e.g.* python collagens and keratins, as well as conserved intracellular household proteins) were removed. These two steps reduced the list of proteins identified in the stomach samples from 314 to 114 proteins (Supplementary table S11). It cannot be excluded that a few proteins belonging to the python stomach secretome also were removed.

To identify the secretome, the 114 identified proteins were manually analyzed as described in the method section (Supplementary table S11). In addition to household proteins, the identified intracellular proteins also included intracellular stomach-specific proteins (*e.g.* the stomach specific calpain 9 cysteine protease [33]), underlining the specificity of the proteomics analysis. In total, 37 proteins constituted the putative python stomach secretome (Supplementary table S7). These could be divided into 18 gastric mucosal-related proteins (e.g. mucin homologous and gastrokine), 7 proteolytic enzymes (mainly pepsin homologous), 4 other hydrolytic enzymes (e.g. phospholipases), and 8 other proteins (e.g. gastric intrinsic factor) (Supplementary table S7). Previous gastric juice proteomics analyses were performed on samples obtained from fasting humans, most likely to avoid the complex prey-protein

background. In our study, we identify, for the first time, stomach-related proteins from a digesting individual and thereby demonstrate that the sensitivity of modern LC-MS/MS equipment allows the identification of gastric juice proteins that are present during digestion.

Discussion

A primary motivation for our description of the temporal changes in gene expression profiles as the visceral organs of Burmese pythons made the transition from fasting to digestion was to identify key regulatory genes and pathways responsible for the pronounced tissue restructuring as well as the increased functional capacity during the postprandial period. An equally important motivation was to address specific hypothesis on the upregulation of certain pathways known to be involved in the secretion of digestive juices and enzymes as well as the absorption of the nutrients as digestion proceed. We achieved these goals by identifying the biochemical and physiological roles of the highly expressed genes with significantly increased expression during digestion and by using KEGG analysis of specific pathways underlying physiological responses known to be stimulated by digestion. We also present GO enrichment analyses of both up-regulated genes and highly expressed genes in all organs (Figs. 9-13), showing that "biological process" is the most common enriched category.

Physiological interpretation of the upregulated genes in the stomach

The considerable changes in gene expression in the stomach were reflected in a pronounced rise in expression of ribosomal 40S and 60S proteins (Fig. 4) that is likely to have attended a rise in protein synthesis required for the marked transition from a quiescent fasting state to the activated digestive state. This is also supported by the presence of ribosomal functions in the enriched GO analysis of the stomach of the highly expressed genes (Fig. 9B). During fasting, gastric acid secretion and presumably also the secretion of digestive enzymes and lysozymes, is halted, such that the gastric juice has a neutral pH, whilst ingestion of prey is followed by an immediate activation of gastric acid secretion [34, 35]. The stimulation of the secretory actions of the stomach is attended by an increased mass of the stomach, where

particularly the mucosa expands already within the first 24h [36].

The KEGG analysis, however, shows that the genes encoding for the gastric H,K ATPase, the active and ATP consuming ion-transporter responsible for gastric acid secretion, are highly expressed in fasting animals, and not additionally elevated in the postprandial period (Fig. 16). This strongly indicates that the enzymatic machinery for gastric acid secretion is maintained during fasting, a trait that may enable fast activation of acid secretion, at modest energetic expenditure, to kill bacteria and match gastric pH to the optimum value for pepsin. This interpretation is consistent with a number of recent studies indicating a rather modest contribution of gastric acid secretion to the specific dynamic action (SDA) response in pythons [37, 38], but we also did observe a high prevalence of ATP synthase subunits (Fig. 4) amongst the highly upregulated genes, which does indicate a rise in aerobic metabolism (see also Fig. 9). Furthermore, the upregulation of the gene encoding for creatine kinase (Fig. 4) indicate increased capacity for aerobic respiration required costs of acid secretion and the stimulation of the accompanying gastric functions. It has been proposed that gastric processes account for more than half of the rise in total metabolism during digestion [34], and aerobic metabolism of isolated gastric strips *in vitro* increased during digestion [39]. However, while metabolism of the stomach certainly must increase during the postprandial period, more recent studies indicate a considerably smaller contribution of gastric acid secretion to the total SDA response is considerable lower than 50% [37, 38, 40].

Our KEGG analysis also showed a large rise in expression of the gene encoding for carbonic anhydrase (Fig. 16), the enzyme that hydrates CO_2 and provide protons for gastric acid secretion. Gastric acid secretion, therefore, does not appear to under transcriptional regulation, but is likely to involve translocation of existing H,K ATPases in vesicles from intracellular vacuoles to the apical membrane of the oxyntopeptic cells that are responsible

for both gastric acid secreting as well as the release of pepsinogen in reptiles [41]. An activation of the processes involved in vesicle transport is further supported by increased transcription of the gene encoding for CD63 (Fig. 4), which belongs to the tetraspanin family and mediate signal transduction events.

In contrast to acid secretion, expression of several genes encoding for digestive enzymes (embryonic pepsinogen-like, gastricsin precursor and gastricsin-like) (Fig. 4) were upregulated, which is consistent with *de novo* synthesis of the enzymes responsible gastric protein degradation. Also there was good overlap between the upregulation of the relevant genes encoding for the proteins identified in the stomach secretome, such as gastrokines, pepsin homologous, phospholipases and gastric intrinsic factor (Supplementary table S7). In this context, it is also interesting that mucin 6 (Fig. 4), the gene coding for the large glycoprotein (gastric mucin) that protects the gastric mucosa from the acidic and proteolytically active chyme in the stomach lumen was upregulated. Thus, as gastric acid secretion is activated, probably in response to increased levels of the gastrin as well as luminal factors, there is an accompanying activation of the protective mucus layer that prevents auto-digestion of the gastric mucosa. It is also noteworthy that the genes for both gastrokine 1 and 2 were upregulated during digestion (Fig. 4). Gastrokines are constitutively produced proteins in the gastric mucosa in mammals and chickens, and while the physiological function remain somewhat elusive, they appear to upregulated during mucosal remodeling in response to inflammation (*e.g.* in connection with ulcers) and often downregulated in cancers. Thus, it is likely that the gastrokines are involved in regulating the restructuring of the mucosa during digestion in pythons.

In addition to analyzing the gene expression profiles of the stomach, we also used a proteomics approach, assisted by our python transcriptome sequence database, to identify the

hydrolytic enzymes in the gastric juice secreted during digestion. We identified python proteins on a complex background of highly abundant mice proteins. Python's digested food is, when it enters the duodenom, overall similar to digested food in e.g. humans. Thus, the digestive enzymes secreted by the pancreas are probably functional similar to known hydrolytic enzymes from other species. Consequently, the enzymes that facilitate the extreme digestion process and allow for have to be present in the stomach's digestive fluid.

We hypothesized that relative aggressive proteolytic digestive enzymes in the gastric juice facilitate digestion of large and un-masticated whole prey items [8]. In our analysis, six out of the seven identified proteolytic enzymes were pepsinogens homologous (Peptidase subfamily A1A), and these were also the most abundant hydrolytic enzymes in the gastric juice according to the emPAI values (Supplementary table S7). Most likely other pepsinogen isoforms exist in the gastric juice, as our approach predominantly target the most abundant proteolytic enzymes. The importance of the proteomics-identified pepsinogens was also substantiated by the transcriptomics data (Supplementary table S8). Here, we found that the six different pepsinogens were upregulated between 2.2 and 22.2 fold from the fasting animals to 48 hours after ingestion of mice. In average the pepsinogen transcripts were upregulated 10.7 fold. It supports that these proteases play a substantial role in the aggressive digestion process performed by the python.

Our proteomic analysis also suggested the identification of the pepsinogens as the major digestive proteolytic enzymes is similar to all other vertebrate species. Thus, our results indicate that it is not unique (with respect to protease class) and hitherto uncharacterized proteases that facilitate the aggressive digestion process. Instead, pepsins, homologous to pepsins among other species, digest the intact swallowed prey. The general condition in the stomach during digestion (e.g. pH) is also similar to other species. Thus, it is

likely that these pepsins variants are among the most effective and aggressive pepsins identified so far and the provided sequence information facilitate future cloning, expression, and characterization of these potential industrial relevant enzymes.

Physiological interpretation of the upregulated genes in the intestine

The small intestine of pythons undergoes a remarkable and fast expansion during digestion where both wet and dry mass more than doubles within the first 24 hours. The expansion stems primarily from increased mucosal mass, achieved by swelling of the individual enterocytes [42], while the smooth muscle in the gut wall is much less responsive [43]. Nevertheless, the GO enrichment analysis also highlights functions pertaining to mitotic cell division, which may indicate a contribution to growth by hyperplasia faster cell turnover (Fig. 10). The expansion of the individual enterocytes is accompanied by pronounced elongation of the microvilli [44] and the resulting rise in surface area of the intestinal lining is accompanied by an ten-fold increase in intestinal transport capacity for amino acids and other nutrients [1, 4, 45]. It remains, however, unknown to what extent the increased capacity for nutrient uptake is also driven by increased synthesis of nutrient transporters. In this context, it is noteworthy that there were no nutrient transporters amongst the highly expressed and upregulated genes in the intestine (Fig. 5), but our KEGG analysis nevertheless showed increased expression of the serosal L-type amino acid transporter. Clearly, it would be worthwhile to quantitatively analyze the extent to which *de novo* synthesis of the various nutrient transporters, particularly those for amino acids, is increased during digestion and how much such synthesis contribute to absorptive capacity. It would seem adaptive if many of the transporters merely have to be activated, either by insertion within the luminal membrane or exposed as the enterocytes expand, to allow for an energetically cheap manner of matching intestinal performance to the sudden appearance of nutrients in the intestine after

a meal. The GO enrichment analysis also pointed to an enrichment of various metabolic processes during digestion, particularly for the upregulated genes (Fig. 10). It is noteworthy that the expression of genes for glutathione S-transferase, peroxiredoxin and selenoprotein increased during digestion (Fig. 5). These three proteins are involved in cellular defence, particularly as antioxidants as a likely protection of reactive oxygen species resulting from increased aerobic metabolism.

There is consensus that the anatomical and structural responses underlying this phenotypic flexibility of intestinal function occur at modest energetic expenditure [34, 46, 47], but our expression profile does show increased expression of the gene coding for Cytochrome P450 pointing to increased aerobic and mitochondrial metabolism. This rise in metabolism may be driven primarily by the massive rise in secondary active transport to absorb the amino acids and smaller peptides rather than the structural changes [46]. Nevertheless, the structural changes may be reflected in increased expression of galectin 1 (Fig. 5), which mediate numerous function including cell–cell interactions, cell–matrix adhesion and transmembrane signaling.

Fig. 5 reveals the importance of lipid absorption and the subsequent transport by the cardiovascular and lymph systems, and it is also possible that several of the expressed proteins play a role in the incorporation of lipid droplets within the enterocytes. Thus, the presence of numerous apolipoproteins, and their precursor apoe protein, amongst the list of highly expressed and highly expressed genes (Fig. 5) are probably needed to transport the absorbed lipids in plasma and lymph, but the apolipoproteins could also act enzyme cofactors, receptor ligands, and lipid transfer carriers in the regulation of lipoprotein metabolism and cellular uptake. Diazepam-binding inhibitor (Fig. 5), a protein involved in lipid metabolism and under hormonal regulation mostly within nervous tissue, is also likely

to reflect the increased lipid absorption and metabolism in the postprandial period, and there was also a rise phospholipases (Fig. 5) that are likely to be involved in lipid degradation. Also, the capacity for protein metabolism clearly increased in the intestine during digestion (meprin A and endopeptidase that cleaves peptides, as well as 4-aminobutyrate aminotransferase, 4-trimethylaminobutyraldehyde dehydrogenase and diamine acetyltransferase) and there was a rise in the ammonium transporter protein Rh (Fig. 5). Finally, a number of proteins involved in calcium uptake and metabolism, such as calbindin and calmodulin (Fig. 5), could be important to handle the break-down of the bone in a normal rodent, and it was recently shown the enterocytes of pythons contain small particles of bone already 24 hours after ingestion [44].

Physiological interpretation of the upregulated genes in the heart

The large metabolic response to digestion is tailored by a doubling of heart rate and stroke of the heart such that cardiac output remains elevated for many days during digestion [48, 49]. This cardiovascular response plays a pivotal role in securing adequate oxygen delivery to the various organs and serves to ensure an appropriate convective transport of the nutrients taken up by the intestine. The tachycardia is mediated by a release of vagal tone and the presence of a non-adrenergic-non-cholinergic stimulation of the heart, which has been speculated to be released from the gastrointestinal organs during digestion [50, 51]. The increased heart rate, and the rise in the amount of blood pumped with each beat, must be supported by increased metabolism of the myocardium and we observed a significant upregulation of malate dehydrogenase, cytochromes and ATPase linked enzymes (Fig. 6) that are likely to be related to an increased oxidative phosphorylation within the individual myocytes (see also the prevalence of enriched GO terms associated with aerobic metabolism in Fig. 13). Previous gene expression studies on the python heart also yielded evidence for increased oxidative

capacity in postprandial period [52] and cytochrome oxidase activity is almost doubled during digestion [53], and we confirm that transcription for heat shock proteins may be increased [52], possibly to protect against oxidative damage as result of the increased metabolism. As in earlier studies [52], our observation of increased ATP synthase lipid–binding protein and fatty acid binding protein 3 (Fig. 6) provide evidence for increased fatty acid metabolism, which may reflect the substantial rise in circulating fatty acids in the plasma.

It was originally suggested that the postprandial rise in stroke volume could be ascribed to an impressive and swift growth of the heart [10], possibly triggered lipid-signalling [52]. However, a number of recent studies, primarily from our laboratory, have shown that increased cardiac mass is not an obligatory postprandial response amongst pythons [53-55], and that stroke volume may be increased in response to increased venous return rather than cardiac hypertrophy [53]. It is nevertheless, noteworthy that our and the previous studies show a clear increase in the expression of contractile proteins (e.g. myosin and actin) as well as tubulin (Fig. 6), which may reflect increased protein-turnover in response to increased myocardial workload rather than cell proliferation or hypertrophy. The enriched GO analyses also point to major changes in the extracellular space as well as both elastin and collagen, which may indicate some level of cardiac reorganisation at the cellular or subcellular level that may alter compliance of the myocardial wall and influence cardiac filling (Fig. 13). It is noteworthy that the increased expression of BNP may serve a signalling function as described in response to the cardiac hypertrophy that attends hypertension.

Physiological interpretation of the genes in the liver

The liver exhibited a diverse expression profile in response to digestion that is likely to reflect its many metabolic functions in connection with metabolism, synthesis and

detoxification during the postprandial period. This patterns is also evident from the many metabolic functions identified in the enriched GO analysis (Fig. 12). There were marked upregulations of the P450 system (Fig. 7), which stems well with a rise in synthesis and breakdown of hormones and signaling molecules, cholesterol synthesis in response to lipid absorption and possibly also an increased metabolism of potentially toxic compounds in the prey. A rise in cholesterol metabolism was supported by increased expression apolipoproteins (Fig. 7). The hepatic involvement in lipid metabolism was also supported by the increased expression of genes for Alpha-2-macroglobulin and serum albumin (Fig. 7). The increased expression of albumin obviously also fits nicely with the proteomic analysis of plasma proteins and it is likely that the postprandial rise in plasma albumin serves a functional role in the lipid transport between the intestine and the liver as well as other metabolically active organs

It is also noteworthy that a number of genes associated with the protection of oxidative stress, such as catalase, heat shock protein and glutathionine transferase were markedly upregulated (Fig. 7). It was recently argued that snakes digesting large meals experience oxidative damage due to reactive oxygen metabolites requiring increased antioxidant responses to protect cellular functions [56].

Physiological interpretation of the genes in the pancreas

We sampled the entire pancreas for our analysis of gene expression and our data therefore reflect both endocrine and exocrine pancreatic functions. The vast majority of the upregulated genes concerned the exocrine pancreas, and we found ample evidence for upregulated expression of genes associated with the digestive functions, such as lipases, trypsin, chymotrypsin and elastase and other enzymes for digestion of protein and lipid (Fig. 8). This general upregulation of secretory processes is likely to explain the prevalence of processes

associuated with protein synthesis in the enriched GO analysis (Fig. 11). There was even an increased expression of amylase (Fig. 8) that breaks down polysaccharides. In connection with this latter function, the increased expression of insulin (Fig. 8) from the endocrine pancreas is likely to reflect increased cellular signaling for postprandial uptake of both glucose and amino acids. As in the other organs, we found increased expression of cytochrome oxidase (Fig. 8) indicative of increased metabolism during digestion, and the rise in heat shock protein expression may reflect a response to formation of reactive oxygen-species as metabolism is stimulated by increased secretion of the pancreas.

Conclusions

Our study shows that the substantial physiological and anatomical reorganisation of the visceral organs during the postprandial period is driven by massive changes in gene expression profiles involving differential expression of hundreds or thousands of genes. Many of the upregulated functions pertain to energy production to support the rise in metabolism associated with digestion and absorption of the large meals. In terms of the gastrointestinal organs, the gene expression profiles also supports the view that many of the digestive functions, such as gastric acid secretion and nutrient absorption, can be stimulated with little gene expression indicating that the proteins involved in these processes are merely need to be activated during the postprandial period, and thus avoiding the energy and time-consuming processes associated with *de novo* synthesis. This digestive strategy may, at least in part, explain how intermittent feeders, such as snakes, retain the capacity for fast and reliable upregulation of the digestive processes immediately after ingestion.

Methods

Stimulation of the postprandial response, collection of tissue biopsies and purification of RNA for mRNA-seq analyses

Six *Python molurus* (Tiger Python/Burmese Python) with a body mass ranging from 180 to 700 g (average 373 g) were obtained from a commercial supplier and housed in vivaria with a heating system providing temperatures of 25-32 °C. The animals were fed rodents once a week and fresh water was always available. The animals appeared healthy and all experiments were performed according to Danish Federal Regulations. All six individuals were fasted for one month and divided in three groups. Four animals were fed a rodent meal of 25 % of body weight and euthanized with an intra-peritoneal injection of pentobarbital (50 mg kg^{-1}; Mebumal) at 24h (N = 2) or 48h after feeding (N = 2). The remaining two snakes served as fasted controls. During deep anaesthesia, two biopsies were obtained from each snake from each of the following tissues: The heart (ventricles), liver, stomach, intestine, and pancreas. In regard to the stomach tissue samples, one sample was obtained from the proximal part of the stomach and one sample was obtained from the distal part. In total, 60 biopsies were collected. The samples were taken from the same part of the different tissues in all individuals. After sampling, the biopsies were weighted and immediately snap frozen in liquid nitrogen; stomach and intestinal tissues were rinsed in sterile saline solution before weighting to avoid contamination with rodent tissue from the ingested meal. Subsequently, all 60 biopsies were homogenized in liquid nitrogen and the four biological replicates (2 biosies from each individual) were pooled in a 1:1 manner based on mass. It resulted in 15 samples (5 tissues X 3 time points). From these samples, total RNA was purified using the Nucleospin RNA II kit (Machery-Nagel GmbH & Co.), as recommended by the manufacturer. The RNA concentration and quality were assessed by Nanodrop ND 1000

Spectrophotometer (Thermo Scientific) analyses, agarose gel-electrophoreses, and Agilent BioAnalyzer (Agilent) analyses.

Library production and sequencing

Poly-A transcripts were enriched and the transcripts broken in the presence of Zn^{2+}. Subsequently, double-stranded cDNA was synthesised using random primers and RNase H. After end repair and purification, the fragments were ligated with bar-coded paired-end adapters, and fragments with insert sizes of approximately 150-250 bp were isolated from an agarose gel. Each of the 15 samples derived from 5 tissues (heart, liver, stomach, pancreas and intestine) at the 3 time points (fasted for 1 month, 24h and 48h post-feeding) were amplified by PCR to generate DNA colonies template libraries and the libraries were then purified. In addition, a part of the samples, which originating from the same tissue, were pooled before the PCR analyses, i.e. in total five pooled samples were generated. These five samples were split in two and after PCR amplification and library purification they were normalized using two different normalization protocols, i.e. in total 10 normalized libraries were prepared. Library quality of all 25 samples was then assessed by a titration-run (1 x 50 bp) on an Illumina HiSeq 2000 instrument. Finally the sequencing was performed on the same instrument using paired-reads (2 × 101 bp). One channel was used for the 15 non-normalized libraries and one channel was used for the 10 normalized libraries.

Data pre-processing and *de novo* transcriptome assembly

To reduce the amount of erroneous data, the raw paired reads were processed by i) removing reads that contained the sequencing adaptor, ii) removing reads that contained ambiguous characters (Ns), and iii) trimming bases that had the low average quality (Q<20) within a sliding window of length 10.

To develop a comprehensive transcriptomics resource for the Burmese python, all high-quality reads from 25 libraries were pooled together for *de novo* assembly. To determine the optimal assembly, *de novo* assembly was performed using Velvet（version 1.2.03) [17] and Oases (version 0.2.06) [57] with different k-mer parameters. The performance of these assemblies were assessed according to number of transcripts, total length of transcripts, N50 length, mean length, proportion of mapped reads and number of transcripts which length is larger than N50 (Supplementary Table S2).

Annotation of the transcriptome and alignment with the genome sequence

To assess the identity of the most closely related gene in other organisms, the assembled transcripts were compared with the sequences in the National Center for Biotechnology Information (NCBI) non-redundant protein (nr) database using blastx [58] with an e-value cut-off of 0.01. The nr annotation term of each transcript was assigned with the first best hit, which was not represented in uninformative description (e.g., 'hypothetical protein', 'novel protein', 'unnamed protein product', 'predicted protein' or 'Uncharacterized protein') (Supplementary Table S3). To assign functional annotations of transcripts, Blast2GO was used (e-value threshold = 0.01) to return GO annotation, Enzyme code annotation with KEGG maps and InterPro annotation.

Gmap [59] was used to map and align our assembled transcripts to python reference genome with parameters 'intronlength = 30000'.

Assessment of the completeness of transcriptome assembly

BUSCO_v2 [18] was used to test the completeness of transcriptome assembly with dependencies NCBI BLAST+ 2.4.0 [60] and HMMER 3.1b2 [61]. The vertebrata lineage set was used and accessed on 28 Nov 2016.

Mutiple sequence alignment of albumin-like genes

T-coffee (version 11.00) [62] was used with default parameters for multiple sequence alignment of albumin-like genes.

Estimation of gene expression values

For each 15 non-normalized libraries, the paired-end reads were firstly mapped back to assembled transcriptome using Bowtie2 [63]with default parameters, the raw counts then were calculated based on the alignment results using RSEM (version 1.1.20) [64] for each transcript. To quantify the gene expression level, for genes with alternative splicing transcripts, the longest transcript was selected to represent the gene, and a gene’s abundance estimate was the sum of its transcripts’ abundance estimates. Finally the raw expression counts were normalized into FPKM with custom Perl scripts.

PCA

To facilitate graphical interpretation of tissue relatedness, R function prcomp was used to perform PCA with genes which the maximum FPKM of 15 samples was greater than 100.

Identification of DEGs and clustering analysis

For each tissue, DEGs were selected with two thresholds, 1) FPKM is greater than or equal to 400 in at least one time point and 2) fold change (FC) is greater than or equal to 2 in at least one pairwise comparison among three time points. FPKM values of DEGs were log2-transformed and median-centered, then hierarchical clustering was performed using R command hclust with method = ‘average’ and distance = ‘Spearman correlation’ and results were displayed using R command heatmap.2.

Colored KEGG Pathway and GO enrichment analysis

For each tissue, all assembled genes were mapped to KEGG human pathway maps using KOBAS 2.0 [65] with e-value 1e-50. Then genes were colored by representing FPKM value and trend of differential expression value (Table 2).

Blast2GO was used to implement GO enrichment analysis (Fisher's exact test) with threshold of FDR 0.001. The reference set is the whole transcripts with GO slim annotation. For each organ, the selected test set is either upregulated or highly expressed genes defined in Table 2. Finally, we performed Blast2GO to reduce to most specific GO terms.

Isolation of samples for proteomics analyses

Two Burmese python at 400 g and 800 g were fed a rodent meal corresponding to approximately 25% of body weight. Approximately 24 h into the postprandial period the animals were euthanized with an overdose of pentobarbital. Immediately afterwards, an incision was made to expose the stomach, which was then ligated at the lower oesophagus and the pylorus, before the intact stomach was excised by a cleavage just below the two sutures resulting in the stomach being released from the rest of the animal. All undigested mouse remains were manually removed by forceps and 25 ml/kg tris-buffered saline (TBS) was injected into the stomach. The stomach was then ligated at the opened end, rinsed by gently shaking the tissue, and finally the digestive fluid-containing solution was collected and stored on ice. To ensure collection of all gastric fluid, the stomach was rinsed an additional 2-3 times with 12 ml/kg TBS. Subsequently, the samples were filtered and centrifuged, and the supernatant stored at -80 °C. The last digestive fluid sample was obtained from a 200 g Burmese python, fed 4 g peptone (Sigma Aldrich), a mixture of small peptides and amino acids, suspended in water. The peptone was injected directly into the stomach and after 3

hours the snake was euthanized by an overdose of pentobarbital. The stomach was removed, rinsed with TBS, and a single sample collected and stored, as described above. In addition, to the six digestive fluid samples a single plasma sample was also obtained.

Sample preparation for mass spectrometry analyses

The proteins in the six obtained python digestive fluid samples were recovered by trichloroacetic acid precipitation. The resulting pellets were resuspended in 8 M Urea, 5 mM DTT, 0.1 M ammonium bicarbonate pH 8.0 and incubated for 30 minutes at room temperature in order to denature and reduce the proteins. Subsequently, the proteins were alkylated by the addition of iodoacetamide to a final concentration of 25 mM. The samples were incubated for additional 20 minutes at room temperature and then diluted 5 times with a 50 mM ammonium bicarbonate, pH 8.0 buffer before the addition of approximately 2 µg sequencing grade modified trypsin (Promega) per 50 µg protein in the sample. Subsequently, the samples were incubated at 37 °C for approximately 16 h. The proteins in the plasma sample were denatured, reduced, alcylated, and digested with trypsin, as described for the digestive fluid samples. Finally, the resulting peptides in all samples were micropurified and stored at -20 C until the LC-MS/MS analyses.

Liquid chromatography-tandem mass spectrometry analyses

Nano-liquid chromatography-tandem mass spectrometry (LC–MS/MS) analyses were performed on a nanoflow HPLC system (Thermo Scientific, EASY-nLC II) connected to a mass spectrometer (TripleTOF 5600, AB Sciex) equipped with an electrospray ionization source (NanoSpray III, AB Sciex) and operated under Analyst TF 1.6 control. The samples were dissolved in 0.1% formic acid, injected, trapped and desalted isocratically on a precolumn whereupon the peptides were eluted and separated on an analytical column (16 cm

× 75 µm i.d.) packed in-house with ReproSil-Pur C18-AQ 3 µm resin (Dr. Marisch GmbH). The peptides were eluted at a flow rate of 250 nL/min using a 50 min gradient from 5 % to 35 % phase B (0.1 % formic acid and 90 % acetonitrile). An information dependent acquisition method was employed allowing up to 25 MS/MS spectra per cycle of 2.8 s.

Protein identification and filtering of data

The six collected MS files, related to digested fluid, were converted to Mascot generic format (MGF) using the AB SCIEX MS Data Converter beta 1.3 (AB SCIEX) and the "proteinpilot MGF" parameters. Subsequently, the files were merged to a single MGF-file using Mascot daemon. The resulting file (encompassing 122538 MS/MS queries) was used to interrogate the 16693 *Mus musculus* sequences in the Swiss-Prot database (version 2014_10) and the generated python database encompassing 21131 protein sequences using Mascot 2.5.0 (Matrix Science)[66]. Trypsin, with up to one missed cleavage allowed, was selected as enzyme; carbamidomethyl was employed as fixed modification, and oxidation of methionine and proline was selected as variable modifications. The instrument setting was specified as ESI-QUAD-TOF, the mass accuracy of the precursor and product ions was 15 ppm and 0.2 da respectively, and the significance threshold (p) was set to 0.01 and an expect cut-off at 0.005. The data obtained by the LC-MS/MS-analysis of the python plasma proteome was analyzed as described for the digestive fluid samples, except that the *Mus musculus* sequences were not interrogated. This dataset contains 9224 MS/MS queries. All obtained results were subsequently parsed using MS Data Miner v. 1.3.0 [67], and protein hits were only accepted if they were identified based on two unique peptides.

To identify the proteins secreted into the python stomach, identified python plasma proteins, as well as the mouse protein homologs were removed from the list of identified python digestive fluid proteins. With regard to the removal of prey protein homologs, the

overall mouse protein names were used to search the list of python proteins (e.g. “collagen” was used as search term, not “collagen alpha-1(I) chain”) and to identify python proteins that were identified based on homology with mouse. These proteins were removed from the list of stomach-secreted python proteins. For each identified protein remaining on the list, we reassessed the annotation of the python sequence, i.a. sequence comparisons were performed using blastp version 2.2.30, and in addition, UniProt and NCBI protein databases, as well as PubMed and SignalP 4.1, were interrogated to identify functional properties and cellular location of the identified proteins. Plasma proteins, remaining collagen homologous, intracellular proteins, and membrane proteins were discarded from the list of identified python stomach secretome proteins.

List of abbreviations

DEG differentially expressed genes

FC fold change

FPKM fragments per kilobase per million sequenced reads

PCA principal component analysis

Declarations

Ethics approval and consent to participate

Not applicable

Consent for publication

Not applicable

Availability of data and material

The raw RNA-Seq sequencing data that support the findings of this study have been deposited in the NCBI BioProject database (accession no. PRJNA343735). https://www.ncbi.nlm.nih.gov/bioproject/PRJNA343735

Competing interests

The authors declare that they have no competing interests.

Funding

This work was supported by a grant (Novenia) from the Danish Research Council for Strategic Research (grant identification number: 09-067076).

Authors' contributions

JD, KWS, TW and MHS designed the study. JD performed the transcriptome data analysis with input from LS and was a major contributor in writing the manuscript. SEL performed RNA-Seq lab experiment. KWS and JE performed the proteomics experiment and data analysis. WT interpreted the transcriptome data regarding digestion. All authors read and approved the final manuscript.

Acknowledgements

We thank Tania A. Nielsen (Aarhus, Denmark) for valuable assistance with RNA purification and Fasteris SA (Switzerland) for library preparation and Illumina sequencing.

Figure and table legends

Fig. 1. The workflow of Python RNA-Seq data analysis. The diagram shows the main steps and bioinformatics tools used in the study.

Fig. 2. PCA plots of FPKM of 1862 genes. PC, principal component. PC1 represents 25%, PC2 represents 18% and PC3 represents 16% of total variation in the data. The name of the label consist two part, one capital letter plus one number. Letter H, S, I, L, P represent heart, stomach, intestine, liver and pancreas respectively. Number 0, 1, 2 represent fasting for one month, 24h/1d after feeding and 48h/2d after feeding respectively.

Fig. 3. Heat maps from hierarchical clustering of DEGs in each tissue. Heat maps showing the hierarchically clustered Spearman correlation matrix resulting from comparing the normalized FPKM value for each pair of genes. Heat map columns represent samples and rows correspond to genes. Expression values (FPKM) are log_2-transformed and then median-centered by gene. Relative levels of gene expression are represented by colors. Pale color is low expression and darker blue is high expression. Five sub-clusters labelled a to e are shown with full annotation in Fig. 4-8.

Fig. 4. The cluster of upregulated genes with NCBI nr annotation in stomach. It shows the cluster e in Fig. 3. Heat map columns represent samples and rows correspond to genes. Expression values (FPKM) are log_2-transformed and then median-centered by gene. Relative levels of gene expression are represented by colors. Pale color is low expression and darker blue is high expression.

Fig. 5. The cluster of upregulated genes with NCBI nr annotation in intestine. It shows the cluster b in Fig. 3. Heat map columns represent samples and rows correspond to genes. Expression values (FPKM) are log_2-transformed and then median-centered by gene. Relative levels of gene expression are represented by colors. Pale color is low expression and darker blue is high expression.

Fig. 6. The cluster of upregulated genes with NCBI nr annotation in heart. It shows the cluster a in Fig. 3. Heat map columns represent samples and rows correspond to genes.

Expression values (FPKM) are $\log_2$-transformed and then median-centered by gene. Relative levels of gene expression are represented by colors. Pale color is low expression and darker blue is high expression.

Fig. 7. The cluster of upregulated genes with NCBI nr annotation in liver. It shows the cluster c in Fig. 3. Heat map columns represent samples and rows correspond to genes. Expression values (FPKM) are $\log_2$-transformed and then median-centered by gene. Relative levels of gene expression are represented by colors. Pale color is low expression and darker blue is high expression.

Fig. 8. The cluster of upregulated genes with NCBI nr annotation in pancreas. It shows the cluster d in Fig. 3. Heat map columns represent samples and rows correspond to genes. Expression values (FPKM) are $\log_2$-transformed and then median-centered by gene. Relative levels of gene expression are represented by colors. Pale color is low expression and darker blue is high expression.

Fig. 9. The enriched GO terms of target genes in stomach. Using all 16992 transcripts annotated with GO terms as reference background, we set (a) 481 upregulated genes and (b) 182 highly expressed genes respectively as test set. The GO terms found over/under represented by a two-tailed Fisher Exact test with multiple testing correction of FDR (Benjamini and Hochberg) below 0.001. The GO terms are categorized and colored as three ontology categories: cellular component in green, molecular function in orange and biological process in black. The pie charts in the left corner display proportion of three categories.

Fig. 10. The enriched GO terms of target genes in intestine. Using all 16992 transcripts annotated with GO terms as reference background, we set (a) 689 upregulated genes and (b) 188 highly expressed genes respectively as test set. The GO terms found over/under represented by a two-tailed Fisher Exact test with multiple testing correction of FDR (Benjamini and Hochberg) below 0.001. The GO terms are categorized and colored as three ontology categories: cellular component in green, molecular function in orange and biological process in black. The pie charts in the left corner display proportion of three categories.

Fig. 11. The enriched GO terms of target genes in pancreas. Using all 16992 transcripts annotated with GO terms as reference background, we set (a) 376 upregulated genes and (b) 205 highly expressed genes respectively as test set. The GO terms found over/under represented by a two-tailed Fisher Exact test with multiple testing correction of FDR (Benjamini and Hochberg) below 0.001. The GO terms are categorized and colored as three ontology categories: cellular component in green, molecular function in orange and biological process in black. The pie charts in the left corner display proportion of three categories.

Fig. 12. The enriched GO terms of target genes in liver. Using all 16992 transcripts annotated with GO terms as reference background, we set (a) 606 upregulated genes and (b) 308 highly expressed genes respectively as test set. The GO terms found over/under represented by a two-tailed Fisher Exact test with multiple testing correction of FDR (Benjamini and Hochberg) below 0.001. The GO terms are categorized and colored as three ontology categories: cellular component in green, molecular function in orange and biological process in black. The pie charts in the left corner display proportion of three categories.

Fig. 13. The enriched GO terms of target genes in heart. Using all 16992 transcripts annotated with GO terms as reference background, we set (a) 107 upregulated genes and (b) 213 highly expressed genes respectively as test set. The GO terms found over/under represented by a two-tailed Fisher Exact test with multiple testing correction of FDR (Benjamini and Hochberg) below 0.001. The GO terms are categorized and colored as three ontology categories: cellular component in green, molecular function in orange and biological process in black. The pie charts in the left corner display proportion of three categories.

Fig. 14. The workflow used to identify the python's stomach secretome during digestion. 1) Initially pythons were feed with mice, or a peptide mixture, and later the gastric juice samples were obtained and mice debris were removed. **2)** The proteins were precipitated, denatured and digested with trypsin. **3)** The resulting tryptic peptides were analyzed by LC-MS/MS analyses and the data merged into a single file. **4)** The file was used to interrogate the in-house generated python protein sequence database (based on the transcriptomic data) and python proteins were identified. **5)** The data was filtered to remove

mice proteins and plasma proteins. Subsequently, the annotation of the remaining proteins were reassessed and the secretome identified.

Fig. 15. Protein sequence alignment of python progastricsin with progastricsin sequences from Anolis Carolinensis and from human. The two longest of the five python protein sequences identified in the gastric juice and annotated as progastricsin-like, were aligned with the two sequences from anole used for the annotation and with the human homolog. The degree of conservation of the individual residues are indicated below the alignment, the active site residues are highlighted in yellow (Asp91, Tyr134, and Asp277 in the human variant – based on MEROPS *the peptidase database*), the cysteine residues are shown in red, and the N-terminal of the activated human gastricsin is highlighted in green. The alignment illustrates that the python's most abundant proteolytic digestive enzymes, the gastricsins, are similar to gastricsins from other species.

Fig. 16. Cartoon depiction of colored KEGG pathway of gastric acid secretion in stomach. Entry in red represents upregulated during digestion; Entry in purple for highly expreseed. H/K is H+/K+-exchanging ATPase alpha polypeptide. CA is carbonic anhydrase. AE is solute carrier family 26 (anion exchange transporter).

Table 1. Summary of transcriptome assembly of Burmese Python.

Table 2. Color coding of genes in KEGG pathway maps. Three criteria are used to classify and color genes. First, i) whether the maximum FPKM of the gene among fasting, 24h and 48h is over 10, then ii) whether the gene is differential expressed in at least one of the pairwise comparison among fasting, 24h and 48h with FC over 4. Finally, iii) for those genes expressed, but not differential expressed, whether it is highly expressed with maximum FPKM among three time points over 200. The term expression trend indicates the trend of gene expression across fasting, 24h and 48h. e.g. The trend up means the gene is upregulated from either fasting to 24h, fasting to 48h or 24h to 48h. The trend up-then-down means the gene is firstly upregulated from fasting to 24h, then downregulated from 24h to 48h.

Table 3. The number of DEGs across fasting, 24h and 48h in each tissue. The expression trend is consistent with definition in Table 2.

Table 4. Upregulated genes and highly expressed genes respectively involved in three KEGG pathways for each tissue. Three pathways are glycolysis/gluconeogenesis, citrate cycle (TCA cycle), and oxidative phosphorylation. The number within bracket after gene description is the corresponding gene ID in NCBI database.

Table 5. Upregulated genes and highly expressed genes respectively involved in two categories of KEGG pathways for each tissue. The two categories are 1.3 lipid metabolism and 1.5 amino acid metabolism in KEGG pathway database. The number within bracket after gene description is the corresponding gene ID in NCBI database.

Reference

1. Secor SM, Diamond J: **A vertebrate model of extreme physiological regulation.** *Nature* 1998, **395:**659-662.
2. Cox CL, Secor SM: **Matched regulation of gastrointestinal performance in the Burmese python, Python molurus.** *J Exp Biol* 2008, **211:**1131-1140.
3. Secor SM, Diamond J: **Adaptive responses to feeding in Burmese pythons: pay before pumping.** *J Exp Biol* 1995, **198:**1313-1325.
4. Secor SM, Diamond JM: **Evolution of regulatory responses to feeding in snakes.** *Physiological and Biochemical Zoology* 2000, **73:**123-141.
5. Aubret F, Shine R, Bonnet X: **Evolutionary biology: adaptive developmental plasticity in snakes.** *Nature* 2004, **431:**261-262.
6. Cohn MJ, Tickle C: **Developmental basis of limblessness and axial patterning in snakes.** *Nature* 1999, **399:**474-479.
7. Ott BD, Secor SM: **Adaptive regulation of digestive performance in the genus Python.** *J Exp Biol* 2007, **210:**340-356.
8. Secor SM: **Digestive physiology of the Burmese python: broad regulation of integrated performance.** *J Exp Biol* 2008, **211:**3767-3774.
9. Zaar M, Overgaard J, Gesser H, Wang T: **Contractile properties of the functionally divided python heart: two sides of the same matter.** *Comp Biochem Physiol A Mol Integr Physiol* 2007, **146:**163-173.
10. Andersen JB, Rourke BC, Caiozzo VJ, Bennett AF, Hicks JW: **Physiology: postprandial cardiac hypertrophy in pythons.** *Nature* 2005, **434:**37-38.
11. Vidal N, Hedges SB: **Molecular evidence for a terrestrial origin of snakes.** *Proc Biol Sci* 2004, **271 Suppl 4:**S226-229.
12. Castoe TA, de Koning AP, Hall KT, Card DC, Schield DR, Fujita MK, Ruggiero RP, Degner JF, Daza JM, Gu W, et al: **The Burmese python genome reveals the molecular basis for extreme adaptation in snakes.** *Proc Natl Acad Sci U S A* 2013, **110:**20645-20650.
13. Andrew AL, Card DC, Ruggiero RP, Schield DR, Adams RH, Pollock DD, Secor SM, Castoe TA: **Rapid changes in gene expression direct rapid shifts in intestinal form and function in the Burmese python after feeding.** *Physiol Genomics* 2015, **47:**147-157.
14. Castoe TA, Fox SE, Jason de Koning A, Poole AW, Daza JM, Smith EN, Mockler TC, Secor SM, Pollock DD: **A multi-organ transcriptome resource for the Burmese Python (Python molurus bivittatus).** *BMC Res Notes* 2011, **4:**310.
15. Wang Z, Gerstein M, Snyder M: **RNA-Seq: a revolutionary tool for transcriptomics.** *Nat Rev Genet* 2009, **10:**57-63.
16. Mortazavi A, Williams BA, McCue K, Schaeffer L, Wold B: **Mapping and quantifying mammalian transcriptomes by RNA-Seq.** *Nat Methods* 2008, **5:**621-628.
17. Zerbino DR, Birney E: **Velvet: algorithms for de novo short read assembly using de Bruijn graphs.** *Genome Res* 2008, **18:**821-829.
18. Simão FA, Waterhouse RM, Ioannidis P, Kriventseva EV, Zdobnov EM: **BUSCO: assessing genome assembly and annotation completeness with single-copy orthologs.** *Bioinformatics* 2015:btv351.
19. Pruitt KD, Tatusova T, Maglott DR: **NCBI reference sequences (RefSeq): a curated non-redundant sequence database of genomes, transcripts and**

proteins. *Nucleic Acids Res* 2007, **35:**D61-65.

20. Benton MJ: **Phylogeny of the major tetrapod groups: morphological data and divergence dates.** *J Mol Evol* 1990, **30:**409-424.
21. Conesa A, Gotz S, Garcia-Gomez JM, Terol J, Talon M, Robles M: **Blast2GO: a universal tool for annotation, visualization and analysis in functional genomics research.** *Bioinformatics* 2005, **21:**3674-3676.
22. Hunter S, Apweiler R, Attwood TK, Bairoch A, Bateman A, Binns D, Bork P, Das U, Daugherty L, Duquenne L, et al: **InterPro: the integrative protein signature database.** *Nucleic Acids Res* 2009, **37:**D211-215.
23. Trapnell C, Williams BA, Pertea G, Mortazavi A, Kwan G, van Baren MJ, Salzberg SL, Wold BJ, Pachter L: **Transcript assembly and quantification by RNA-Seq reveals unannotated transcripts and isoform switching during cell differentiation.** *Nat Biotechnol* 2010, **28:**511-515.
24. Fan HP, Di Liao C, Fu BY, Lam LC, Tang NL: **Interindividual and interethnic variation in genomewide gene expression: insights into the biological variation of gene expression and clinical implications.** *Clin Chem* 2009, **55:**774-785.
25. Kanehisa M, Goto S, Sato Y, Furumichi M, Tanabe M: **KEGG for integration and interpretation of large-scale molecular data sets.** *Nucleic Acids Res* 2012, **40:**D109-114.
26. Kanehisa M, Goto S: **KEGG: kyoto encyclopedia of genes and genomes.** *Nucleic Acids Res* 2000, **28:**27-30.
27. Kageyama T: **Pepsinogens, progastricsins, and prochymosins: structure, function, evolution, and development.** *Cell Mol Life Sci* 2002, **59:**288-306.
28. Hayashi K, Agata K, Mochii M, Yasugi S, Eguchi G, Mizuno T: **Molecular cloning and the nucleotide sequence of cDNA for embryonic chicken pepsinogen: phylogenetic relationship with prochymosin.** *J Biochem* 1988, **103:**290-296.
29. Watanuki K, Yasugi S: **Analysis of transcription regulatory regions of embryonic chicken pepsinogen (ECPg) gene.** *Dev Dyn* 2003, **228:**51-58.
30. Aoki N, Deshimaru M, Terada S: **Active fragments of the antihemorrhagic protein HSF from serum of habu (Trimeresurus flavoviridis).** *Toxicon* 2007, **49:**653-662.
31. Perales J, Neves-Ferreira AG, Valente RH, Domont GB: **Natural inhibitors of snake venom hemorrhagic metalloproteinases.** *Toxicon* 2005, **45:**1013-1020.
32. Tomihara Y, Yonaha K, Nozaki M, Yamakawa M, Kawamura Y, Kamura T, Toyama S: **Purification of an antihemorrhagic factor from the serum of the non-venomous snake Dinodon semicarinatus.** *Toxicon* 1988, **26:**420-423.
33. Lee HJ, Sorimachi H, Jeong SY, Ishiura S, Suzuki K: **Molecular cloning and characterization of a novel tissue-specific calpain predominantly expressed in the digestive tract.** *Biol Chem* 1998, **379:**175-183.
34. Secor SM: **Gastric function and its contribution to the postprandial metabolic response of the Burmese python Python molurus.** *Journal of Experimental Biology* 2003, **206:**1621-1630.
35. Bessler SM, Secor SM: **Effects of feeding on luminal pH and morphology of the gastroesophageal junction of snakes.** *Zoology (Jena)* 2012, **115:**319-329.
36. Helmstetter C, Pope RK, T'Flachebba M, Secor SM, Lignot J-H: **The effects of feeding on cell morphology and proliferation of the gastrointestinal tract of juvenile Burmese pythons (Python molurus).** *Canadian Journal of Zoology* 2009, **87:**1255-1267.

37. Enok S, Simonsen LS, Wang T: **The contribution of gastric digestion and ingestion of amino acids on the postprandial rise in oxygen consumption, heart rate and growth of visceral organs in pythons.** *Comp Biochem Physiol A Mol Integr Physiol* 2013, **165:**46-53.
38. Nørgaard S, Andreassen K, Malte CL, Enok S, Wang T: **Low cost of gastric acid secretion during digestion in ball pythons.** *Comparative Biochemistry and Physiology Part A: Molecular & Integrative Physiology* 2016, **194:**62-66.
39. Secor SM, Taylor JR, Grosell M: **Selected regulation of gastrointestinal acid-base secretion and tissue metabolism for the diamondback water snake and Burmese python.** *J Exp Biol* 2012, **215:**185-196.
40. Andrade DV, De Toledo LF, Abe AS, Wang T: **Ventilatory compensation of the alkaline tide during digestion in the snake Boa constrictor.** *J Exp Biol* 2004, **207:**1379-1385.
41. Koelz HR: **Gastric acid in vertebrates.** *Scand J Gastroenterol Suppl* 1992, **193:**2-6.
42. Starck JM, Beese K: **Structural flexibility of the intestine of Burmese python in response to feeding.** *Journal of Experimental Biology* 2001, **204:**325-335.
43. Holmberg A, Kaim J, Persson A, Jensen J, Wang T, Holmgren S: **Effects of digestive status on the reptilian gut.** *Comparative Biochemistry and Physiology a-Molecular and Integrative Physiology* 2002, **133:**499-518.
44. Lignot JH, Helmstetter C, Secor SM: **Postprandial morphological response of the intestinal epithelium of the Burmese python (Python molurus).** *Comp Biochem Physiol A Mol Integr Physiol* 2005, **141:**280-291.
45. Secor SM, Whang EE, Lane JS, Ashley SW, Diamond J: **Luminal and systemic signals trigger intestinal adaptation in the juvenile python.** *American Journal of Physiology-Gastrointestinal and Liver Physiology* 2000, **279:**G1177-G1187.
46. Overgaard J, Andersen JB, Wang T: **The effects of fasting duration on the metabolic response to feeding in Python molurus: an evaluation of the energetic costs associated with gastrointestinal growth and upregulation.** *Physiol Biochem Zool* 2002, **75:**360-368.
47. Wang T, Hung CC, Randall DJ: **The comparative physiology of food deprivation: from feast to famine.** *Annu Rev Physiol* 2006, **68:**223-251.
48. Secor SM, White SE: **Prioritizing blood flow: cardiovascular performance in response to the competing demands of locomotion and digestion for the Burmese python, Python molurus.** *J Exp Biol* 2010, **213:**78-88.
49. Secor SM, Hicks JW, Bennett AF: **Ventilatory and cardiovascular responses of a python (Python molurus) to exercise and digestion.** *J Exp Biol* 2000, **203:**2447-2454.
50. Skovgaard N, Møller K, Gesser H, Wang T: **Histamine induces postprandial tachycardia through a direct effect on cardiac H2-receptors in pythons.** *American Journal of Physiology - Regulatory, Integrative and Comparative Physiology* 2009, **296:**R774-R785.
51. Enok S, Simonsen LS, Pedersen SV, Wang T, Skovgaard N: **Humoral regulation of heart rate during digestion in pythons (Python molurus and Python regius).** *Am J Physiol Regul Integr Comp Physiol* 2012, **302:**R1176-1183.
52. Riquelme CA, Magida JA, Harrison BC, Wall CE, Marr TG, Secor SM, Leinwand LA: **Fatty acids identified in the Burmese python promote beneficial cardiac growth.** *Science* 2011, **334:**528-531.
53. Enok S, Leite G, Leite C, Gesser H, Hedrick MS, Wang T: **Improved cardiac filling facilitates the postprandial elevation of stroke volume in Python regius.** *J*

Exp Biol 2016.

54. Slay CE, Enok S, Hicks JW, Wang T: **Reduction of blood oxygen levels enhances postprandial cardiac hypertrophy in Burmese python (Python bivittatus).** *J Exp Biol* 2014, **217:**1784-1789.
55. Jensen B, Larsen CK, Nielsen JM, Simonsen LS, Wang T: **Change of cardiac function, but not form, in postprandial pythons.** *Comp Biochem Physiol A Mol Integr Physiol* 2011, **160:**35-42.
56. Butler MW, Lutz TJ, Fokidis HB, Stahlschmidt ZR: **Eating increases oxidative damage in a reptile.** *J Exp Biol* 2016, **219:**1969-1973.
57. Schulz MH, Zerbino DR, Vingron M, Birney E: **Oases: robust de novo RNA-seq assembly across the dynamic range of expression levels.** *Bioinformatics* 2012, **28:**1086-1092.
58. Altschul SF, Gish W, Miller W, Myers EW, Lipman DJ: **Basic local alignment search tool.** *J Mol Biol* 1990, **215:**403-410.
59. Wu TD, Watanabe CK: **GMAP: a genomic mapping and alignment program for mRNA and EST sequences.** *Bioinformatics* 2005, **21:**1859-1875.
60. Camacho C, Coulouris G, Avagyan V, Ma N, Papadopoulos J, Bealer K, Madden TL: **BLAST+: architecture and applications.** *BMC Bioinformatics* 2009, **10:**421.
61. Eddy SR: **A probabilistic model of local sequence alignment that simplifies statistical significance estimation.** *PLoS Comput Biol* 2008, **4:**e1000069.
62. Notredame C, Higgins DG, Heringa J: **T-Coffee: A novel method for fast and accurate multiple sequence alignment.** *Journal of molecular biology* 2000, **302:**205-217.
63. Langmead B, Salzberg SL: **Fast gapped-read alignment with Bowtie 2.** *Nat Methods* 2012, **9:**357-359.
64. Li B, Dewey CN: **RSEM: accurate transcript quantification from RNA-Seq data with or without a reference genome.** *BMC Bioinformatics* 2011, **12:**323.
65. Xie C, Mao X, Huang J, Ding Y, Wu J, Dong S, Kong L, Gao G, Li CY, Wei L: **KOBAS 2.0: a web server for annotation and identification of enriched pathways and diseases.** *Nucleic Acids Res* 2011, **39:**W316-322.
66. Perkins DN, Pappin DJC, Creasy DM, Cottrell JS: **Probability-based protein identification by searching sequence databases using mass spectrometry data.** *Electrophoresis* 1999, **20:**3551-3567.
67. Dyrlund TF, Poulsen ET, Scavenius C, Sanggaard KW, Enghild JJ: **MS Data Miner: A web-based software tool to analyze, compare, and share mass spectrometry protein identifications.** *Proteomics* 2012, **12:**2792-2796.

 Click here to download Table Table1.xlsx

**Parameter**	***De novo* assembly**
Total transcripts	34,423
Annotated transcripts with nr NCBI	19,713
Annotated transcripts with GO term	16,992
Minimum transcript size (nt)	100
Medium transcrpt size (nt)	605
Mean transcript size (nt)	1,034
Largest transcript (nt)	26,010
N50	6,240
N50 size (nt)	1,673
Total assembled bases (Mb)	35.6

Table 2

Click here to download Table Table2.xlsx

Expression level	Fold change level	Expression trend (fasting -> 24h -> 48h)	Color code
max FPKM over 10	FC over 4	Up-regulated	Red
		Down-regulated	Blue
		Up-then-down regulated	Yellow
		Down-then-up regulated	Brown
	FC below 4	Highly expressed (max FPKM over 200)	Purple
		Moderately expressed (max FPKM below 200)	Pink
max FPKM below 10	-	Lowly expressed	Darkgrey

Click here to download Table Table3.xlsx

Expression trend (fasting -> 24h -> 48h)	Stomach	Intestine	Pancreas	Liver	Heart
Up-regulated	932 (2.9%)	1,131 (3.5%)	859 (2.6%)	1,047 (3.2%)	184 (0.6%)
Up-then-down regulated	28 (0.1%)	31 (0.1%)	150 (0.5%)	61 (0.2%)	6 (0.0%)
Down-regulated	869 (2.7%)	625 (1.9%)	567 (1.7%)	618 (1.9%)	168 (0.5%)
Down-then-up regulated	36 (0.1%)	45 (0.1%)	127 (0.4%)	90 (0.3%)	16 (0.1%)
Highly expressed	199 (0.6%)	211 (0.7%)	225 (0.7%)	354 (1.1%)	232 (0.7%)
Moderately expressed	5,541 (17.0%)	5,582 (17.2%)	4,933 (15.2%)	5,385 (16.5%)	6,044 (18.6%)
Lowly expressed	24,926 (76.6%)	24,906 (76.5%)	25,670 (78.9%)	24,976 (76.8%)	25,881 (79.5%)
**Total**	32,531 (100%)	32,531 (100%)	32,531 (100%)	32,531 (100%)	32,531 (100%)

Upregulated Genes			
**Organ**	**Glycolysis / Gluconeogenesis**	**Citrate cycle (TCA cycle)**	**Oxidative phosphorylation**
Stomach	phosphofructokinase, liver (5211)<br>pyruvate dehydrogenase (lipoamide) beta (5162)<br>alcohol dehydrogenase 7 (class IV), mu or sigma polypeptide (131)<br>hexokinase 2 (3099)<br>aldehyde dehydrogenase 9 family member A1 (223)<br>alcohol dehydrogenase 5 (class III), chi polypeptide (128)	ATP citrate lyase (47)<br>isocitrate dehydrogenase 1 (NADP+) (3417)<br>succinate dehydrogenase complex subunit A, flavoprotein (Fp) (6389)<br>pyruvate dehydrogenase (lipoamide) beta (5162)	ubiquinol-cytochrome c reductase, Rieske iron-sulfur polypeptide 1 (7386)<br>ubiquinol-cytochrome c reductase core protein I (7384)<br>succinate dehydrogenase complex subunit A, flavoprotein (Fp) (6389)<br>NADH:ubiquinone oxidoreductase subunit S6 (4726)<br>NADH:ubiquinone oxidoreductase subunit B9 (4715)<br>NADH:ubiquinone oxidoreductase subunit A5 (4698)<br>NADH:ubiquinone oxidoreductase subunit A10 (4705)<br>NADH:ubiquinone oxidoreductase core subunit V2 (4729)<br>cytochrome c oxidase assembly homolog 15 (yeast) (1355)<br>COX11 cytochrome c oxidase copper chaperone (1353)<br>ATPase, H+ transporting, lysosomal 34kDa, V1 subunit D (51382)<br>ATP synthase, H+ transporting, mitochondrial Fo complex subunit B1 (515)<br>ATP synthase, H+ transporting, mitochondrial F1 complex, O subunit (539)<br>ATP synthase, H+ transporting, mitochondrial F1 complex, gamma polypeptide 1 (509)<br>ATP synthase, H+ transporting, mitochondrial F1 complex, beta polypeptide (506)<br>ATP synthase, H+ transporting, mitochondrial F1 complex, alpha subunit 1, cardiac muscle (498)
Intestine	alcohol dehydrogenase 7 (class IV), mu or sigma polypeptide (131)<br>acyl-CoA synthetase short-chain family member 2 (55902)<br>ADP-dependent glucokinase (83440)<br>aldehyde dehydrogenase 9 family member A1 (223)<br>phosphoenolpyruvate carboxykinase 1 (5105)<br>hexokinase 2 (3099)<br>pyruvate dehydrogenase (lipoamide) beta (5162)	pyruvate dehydrogenase (lipoamide) beta (5162)<br>oxoglutarate dehydrogenase-like (55753)<br>phosphoenolpyruvate carboxykinase 1 (5105)<br>ATP citrate lyase (47)<br>isocitrate dehydrogenase 1 (NADP+) (3417)	ATPase, H+ transporting, lysosomal 70kDa, V1 subunit A (523)<br>ATPase, H+ transporting, lysosomal 34kDa, V1 subunit D (51382)<br>ATP synthase, H+ transporting, mitochondrial F1 complex, O subunit (539)<br>NADH:ubiquinone oxidoreductase subunit B9 (4715)
	aldehyde dehydrogenase 9 family member A1 (223)	isocitrate dehydrogenase 1 (NADP+) (3417)	ubiquinol-cytochrome c reductase, Rieske iron-sulfur polypeptide 1 (7386)<br>ATPase, H+ transporting, lysosomal accessory protein 1 (537)

Pancreas			ATPase, H+ transporting, lysosomal 70kDa, V1 subunit A (523)
Liver	alcohol dehydrogenase 1C (class I), gamma polypeptide (126)<br>alcohol dehydrogenase 5 (class III), chi polypeptide (128)<br>acyl-CoA synthetase short-chain family member 2 (55902)<br>phosphoglycerate mutase 1 (5223)<br>enolase 1, (alpha) (2023)<br>aldehyde dehydrogenase 2 family (mitochondrial) (217)<br>aldehyde dehydrogenase 9 family member A1 (223)<br>aldolase, fructose-bisphosphate B (229)<br>pyruvate dehydrogenase (lipoamide) beta (5162)<br>alcohol dehydrogenase 7 (class IV), mu or sigma polypeptide (131)	isocitrate dehydrogenase 1 (NADP+) (3417)<br>pyruvate dehydrogenase (lipoamide) beta (5162)<br>aconitase 1 (48)	ATPase, H+ transporting, lysosomal 70kDa, V1 subunit A (523)<br>ATPase, H+ transporting, lysosomal 56/58kDa, V1 subunit B2 (526)
Heart	aldolase, fructose-bisphosphate B (229)	isocitrate dehydrogenase 1 (NADP+) (3417)	

Highly expressed genes			
**Organ**	**Glycolysis / Gluconeogenesis**	**Citrate cycle (TCA cycle)**	**Oxidative phosphorylation**
Stomach	glyceraldehyde-3-phosphate dehydrogenase (2597)<br>pyruvate kinase, muscle (5315)	isocitrate dehydrogenase 2 (NADP+), mitochondrial (3418)<br>malate dehydrogenase 1 (4190)	ATP synthase, H+ transporting, mitochondrial Fo complex subunit C3 (subunit 9) (518)<br>ATPase, H+/K+ exchanging, alpha polypeptide (495)<br>ATPase, H+/K+ exchanging, beta polypeptide (496)<br>cytochrome c oxidase subunit I (4512)<br>cytochrome c oxidase subunit IV isoform 1 (1327)<br>cytochrome c1 (1537)<br>NADH dehydrogenase, subunit 1 (complex I) (4535)
Intestine	aldolase, fructose-bisphosphate B (229)<br>enolase 1, (alpha) (2023)<br>aldehyde dehydrogenase 2 family (mitochondrial) (217)<br>glyceraldehyde-3-phosphate dehydrogenase (2597)	malate dehydrogenase 1 (4190)	ATP synthase, H+ transporting, mitochondrial F1 complex, alpha subunit 1, cardiac muscle (498)<br>ATP synthase, H+ transporting, mitochondrial F1 complex, beta polypeptide (506)<br>NADH dehydrogenase, subunit 1 (complex I) (4535)<br>cytochrome c oxidase subunit I (4512)<br>ATP synthase, H+ transporting, mitochondrial Fo complex subunit C3 (subunit 9) (518)<br>cytochrome c oxidase subunit IV isoform 1 (1327)
Pancreas	glucose-6-phosphate isomerase (2821)<br>glyceraldehyde-3-phosphate dehydrogenase (2597)		ATP synthase, H+ transporting, mitochondrial F1 complex, beta polypeptide (506)<br>NADH:ubiquinone oxidoreductase subunit A5 (4698)<br>ATP synthase, H+ transporting, mitochondrial F1 complex, alpha subunit 1, cardiac muscle (498)<br>ATP synthase, H+ transporting, mitochondrial Fo complex subunit C3 (subunit 9) (518)<br>cytochrome c oxidase subunit I (4512)<br>NADH dehydrogenase, subunit 1 (complex I) (4535)<br>cytochrome c oxidase subunit IV isoform 1 (1327)
	fructose-bisphosphatase 1 (2203)<br>phosphoglycerate kinase 1 (5230)<br>glyceraldehyde-3-phosphate dehydrogenase (2597)<br>lactate dehydrogenase B (3945)<br>triosephosphate isomerase 1 (7167)<br>phosphoglucomutase 2 (55276)	malate dehydrogenase 1 (4190)<br>isocitrate dehydrogenase 2 (NADP+), mitochondrial (3418)<br>malate dehydrogenase 2 (4191)	ATP synthase, H+ transporting, mitochondrial Fo complex subunit C3 (subunit 9) (518)<br>ATP synthase, H+ transporting, mitochondrial F1 complex, gamma polypeptide 1 (509)<br>NADH dehydrogenase, subunit 1 (complex I) (4535)<br>cytochrome c oxidase subunit I (4512)<br>cytochrome c oxidase subunit IV isoform 1 (1327)<br>ATP synthase, H+ transporting, mitochondrial F1 complex, beta polypeptide (506)

Liver	glucose-6-phosphate isomerase (2821)		ATP synthase, H+ transporting, mitochondrial F1 complex, alpha subunit 1, cardiac muscle (498)<br>ubiquinol-cytochrome c reductase, Rieske iron-sulfur polypeptide 1 (7386)<br>cytochrome c1 (1537)
Heart	pyruvate kinase, muscle (5315)<br>glyceraldehyde-3-phosphate dehydrogenase (2597)<br>triosephosphate isomerase 1 (7167)<br>enolase 2 (gamma, neuronal) (2026)<br>glucose-6-phosphate isomerase (2821)<br>phosphoglycerate mutase 2 (5224)<br>aldolase, fructose-bisphosphate C (230)<br>phosphoglycerate kinase 1 (5230)<br>aldolase, fructose-bisphosphate A (226)	succinate dehydrogenase complex subunit A, flavoprotein (Fp) (6389)<br>succinate dehydrogenase complex subunit B, iron sulfur (Ip) (6390)<br>isocitrate dehydrogenase 2 (NADP+), mitochondrial (3418)<br>malate dehydrogenase 2 (4191)<br>aconitase 2 (50)<br>malate dehydrogenase 1 (4190)	NADH:ubiquinone oxidoreductase subunit A5 (4698)<br>succinate dehydrogenase complex subunit B, iron sulfur (Ip) (6390)<br>cytochrome c oxidase subunit IV isoform 1 (1327)<br>succinate dehydrogenase complex subunit A, flavoprotein (Fp) (6389)<br>NADH:ubiquinone oxidoreductase subunit A8 (4702)<br>NADH:ubiquinone oxidoreductase subunit A10 (4705)<br>ATP synthase, H+ transporting, mitochondrial F1 complex, alpha subunit 1, cardiac muscle (498)<br>ATP synthase, H+ transporting, mitochondrial F1 complex, O subunit (539)<br>ATP synthase, H+ transporting, mitochondrial F1 complex, beta polypeptide (506)<br>NADH dehydrogenase, subunit 1 (complex I) (4535)<br>cytochrome c oxidase subunit I (4512)<br>NADH:ubiquinone oxidoreductase subunit B10 (4716)<br>NADH:ubiquinone oxidoreductase subunit B9 (4715)<br>ATP synthase, H+ transporting, mitochondrial Fo complex subunit B1 (515)<br>ATP synthase, H+ transporting, mitochondrial F1 complex, gamma polypeptide 1 (509)<br>cytochrome c1 (1537)<br>NADH:ubiquinone oxidoreductase core subunit S7 (374291)<br>ubiquinol-cytochrome c reductase, Rieske iron-sulfur polypeptide 1 (7386)<br>ubiquinol-cytochrome c reductase core protein I (7384)<br>ATP synthase, H+ transporting, mitochondrial Fo complex subunit C3 (subunit 9) (518)<br>NADH:ubiquinone oxidoreductase subunit S6 (4726)

1.3 Lipid metabolism	Stomach	Intestine	Pancreas	Liver	Heart
	emopamil binding protein (sterol isomerase) (10682)	hydroxyacyl-CoA dehydrogenase/3-ketoacyl-CoA thiolase/enoyl-CoA hydratase (trifunctional protein), beta subunit (3032)	lysophosphatidylcholine acyltransferase 3 (10162)	ectonucleotide pyrophosphatase/phosphodiesterase 6 (133121)	carboxyl ester lipase (1056)
	NAD(P) dependent steroid dehydrogenase-like (50814)	sterol-C5-desaturase (6309)	methylsterol monooxygenase 1 (6307)	steroid-5-alpha-reductase, alpha polypeptide 1 (3-oxo-5 alpha-steroid delta 4-dehydrogenase alpha 1) (6715)	
	ELOVL fatty acid elongase 6 (79071)	ceramide synthase 1 (10715)	cytochrome P450 family 51 subfamily A member 1 (1595)	hydroxysteroid (17-beta) dehydrogenase 4 (3295)	
	sterol-C5-desaturase (6309)	bile acid-CoA:amino acid N-acyltransferase (570)	emopamil binding protein (sterol isomerase) (10682)	cytochrome P450 family 2 subfamily C member 19 (1557)	
	cytochrome P450 family 51 subfamily A member 1 (1595)	3-hydroxy-3-methylglutaryl-CoA synthase 1 (3157)	aldehyde dehydrogenase 9 family member A1 (223)	hydroxysteroid (17-beta) dehydrogenase 12 (51144)	
	lysophosphatidylcholine acyltransferase 3 (10162)	glycerol-3-phosphate acyltransferase 3 (84803)	NAD(P) dependent steroid dehydrogenase-like (50814)	acyl-CoA oxidase 2, branched chain (8309)	
	CDP-diacylglycerol synthase 1 (1040)	CDP-diacylglycerol synthase 1 (1040)	7-dehydrocholesterol reductase (1717)	24-dehydrocholesterol reductase (1718)	
	hydroxysteroid (17-beta) dehydrogenase 7 (51478)	sphingosine kinase 1 (8877)	acetyl-CoA acyltransferase 2 (10449)	3-hydroxyacyl-CoA dehydratase 2 (201562)	
	aldehyde dehydrogenase 9 family member A1 (223)	hydroxy-delta-5-steroid dehydrogenase, 3 beta- and steroid delta-isomerase 7 (80270)		3-hydroxymethyl-3-methylglutaryl-CoA lyase (3155)	
	lanosterol synthase (2,3-oxidosqualene-lanosterol cyclase) (4047)	lysophosphatidylcholine acyltransferase 3 (10162)		sphingomyelin phosphodiesterase 1 (6609)	
	acetyl-CoA acetyltransferase 1 (38)	diacylglycerol O-acyltransferase 1 (8694)		aldehyde dehydrogenase 9 family member A1 (223)	
	methylsterol monooxygenase 1 (6307)	aldehyde dehydrogenase 9 family member A1 (223)		acetyl-CoA acyltransferase 2 (10449)	
	hydroxysteroid (17-beta) dehydrogenase 12 (51144)	acetyl-CoA acyltransferase 2 (10449)		phosphate cytidylyltransferase 2, ethanolamine (5833)	
	7-dehydrocholesterol reductase (1717)	choline/ethanolamine phosphotransferase 1 (10390)		ceramide synthase 1 (10715)	
	24-dehydrocholesterol reductase (1718)	carbonyl reductase 1 (873)		aldehyde dehydrogenase 2 family (mitochondrial) (217)	
	ELOVL fatty acid elongase 1 (64834)	glycerol-3-phosphate dehydrogenase 1-like (23171)		cytochrome P450 family 8 subfamily B member 1 (1582)	
		ELOVL fatty acid elongase 6 (79071)		glyceronephosphate O-acyltransferase (8443)	
		thromboxane A synthase 1 (6916)		serine palmitoyltransferase long chain base subunit 2 (9517)	
		hydroxysteroid (17-beta) dehydrogenase 2 (3294)		sulfotransferase family 2B member 1 (6820)	
		hydroxysteroid (17-beta) dehydrogenase 7 (51478)		cytochrome P450 family 1 subfamily A member 1 (1543)	
		N-acylsphingosine amidohydrolase (non-lysosomal ceramidase) 2 (56624)		fatty acid synthase (2194)	
		glycerol kinase (2710)		lysophosphatidylcholine acyltransferase 3 (10162)	
		emopamil binding protein (sterol isomerase) (10682)		glutathione peroxidase 7 (2882)	

Up-regulated gene		ectonucleotide pyrophosphatase/phosphodiesterase 7 (339221)<br>arylsulfatase A (410)<br>transmembrane 7 superfamily member 2 (7108)<br>hydroxysteroid (17-beta) dehydrogenase 12 (51144)<br>cytochrome P450 family 3 subfamily A member 4 (1576)<br>diacylglycerol O-acyltransferase 2 (84649)<br>ELOVL fatty acid elongase 1 (64834)<br>7-dehydrocholesterol reductase (1717)<br>phosphate cytidylyltransferase 1, choline, beta (9468)<br>stearoyl-CoA desaturase (delta-9-desaturase) (6319)<br>cytochrome P450 family 8 subfamily B member 1 (1582)		sterol carrier protein 2 (6342)<br>fatty acid desaturase 1 (3992)<br>3-hydroxybutyrate dehydrogenase, type 2 (56898)<br>bile acid-CoA:amino acid N-acyltransferase (570)<br>1-acylglycerol-3-phosphate O-acyltransferase 3 (56894)<br>lysophosphatidylglycerol acyltransferase 1 (9926)<br>methylsterol monooxygenase 1 (6307)<br>peroxisomal trans-2-enoyl-CoA reductase (55825)<br>diacylglycerol O-acyltransferase 1 (8694)	
Highly expressed gene	hydroxyacyl-CoA dehydrogenase (3033)<br>glutathione peroxidase 1 (2876)	hydroxyacyl-CoA dehydrogenase (3033)<br>leukotriene A4 hydrolase (4048)<br>catechol-O-methyltransferase (1312)<br>aldehyde dehydrogenase 2 family (mitochondrial) (217)	carboxyl ester lipase (1056)<br>pancreatic lipase-related protein 1 (5407)	glycerol-3-phosphate dehydrogenase 1 (2819)<br>glutathione peroxidase 3 (2878)<br>sphingosine-1-phosphate lyase 1 (8879)<br>prostaglandin E synthase 3 (10728)<br>catechol-O-methyltransferase (1312)<br>hydroxyacyl-CoA dehydrogenase (3033)<br>cytochrome P450 family 1 subfamily A member 2 (1544)	3-oxoacid CoA-transferase 1 (5019)

**1.5 Amino acid metabolism**	Stomach	Intestine	Pancreas	Liver	Heart
	branched chain amino-acid transaminase 2, mitochondrial (587)	acyl-CoA dehydrogenase, short/branched chain (36)	aldehyde dehydrogenase 9 family member A1 (223)	alanine-glyoxylate aminotransferase (189)	creatine kinase, brain (1152)
	3-hydroxyisobutyrate dehydrogenase (11112)	creatine kinase, brain (1152)	asparagine synthetase (glutamine-hydrolyzing) (440)	acyl-CoA dehydrogenase, short/branched chain (36)	
	cysteine dioxygenase type 1 (1036)	nitrilase family member 2 (56954)	alanine-glyoxylate aminotransferase (189)	phosphoserine aminotransferase 1 (29968)	
	aldehyde dehydrogenase 9 family member A1 (223)	acetyl-CoA acyltransferase 2 (10449)	glutaminase (2744)	monoamine oxidase B (4129)	
	glutaryl-CoA dehydrogenase (2639)	amine oxidase, copper containing 1 (26)	aminomethyltransferase (275)	glutamine--fructose-6-phosphate transaminase 1 (2673)	
	mercaptopyruvate sulfurtransferase (4357)	4-aminobutyrate aminotransferase (18)	glutaryl-CoA dehydrogenase (2639)	aminomethyltransferase (275)	
	cystathionine gamma-lyase (1491)	aldehyde dehydrogenase 9 family member A1 (223)	4-aminobutyrate aminotransferase (18)	5'-aminolevulinate synthase 1 (211)	
	creatine kinase, brain (1152)	aldehyde dehydrogenase 5 family member A1 (7915)	creatine kinase, brain (1152)	argininosuccinate lyase (435)	
	aldehyde dehydrogenase 4 family member A1 (8659)	adenosylhomocysteinase like 2 (23382)	thiosulfate sulfurtransferase (7263)	alcohol dehydrogenase 1C (class I), gamma polypeptide (126)	
	phosphoserine phosphatase (5723)	CNDP dipeptidase 2 (metallopeptidase M20 family) (55748)	cystathionine gamma-lyase (1491)	phosphoglycerate mutase 1 (5223)	
	alcohol dehydrogenase 5 (class III), chi polypeptide (128)	thiosulfate sulfurtransferase (7263)	acetyl-CoA acyltransferase 2 (10449)	carnosine dipeptidase 1 (metallopeptidase M20 family) (84735)	
	alcohol dehydrogenase 7 (class IV), mu or sigma polypeptide (131)	leucine aminopeptidase 3 (51056)	nitric oxide synthase 1 (4842)	glutamic-pyruvate transaminase (alanine aminotransferase) (2875)	
	propionyl-CoA carboxylase alpha subunit (5095)	hydroxyacyl-CoA dehydrogenase/3-ketoacyl-CoA thiolase/enoyl-CoA hydratase (trifunctional protein), beta subunit (3032)	serine hydroxymethyltransferase 1 (soluble) (6470)	3-hydroxymethyl-3-methylglutaryl-CoA lyase (3155)	
	pyruvate dehydrogenase (lipoamide) beta (5162)	phosphoserine aminotransferase 1 (29968)		enoyl-CoA, hydratase/3-hydroxyacyl CoA dehydrogenase (1962)	
	dihydrolipoamide branched chain transacylase E2 (1629)	alcohol dehydrogenase 7 (class IV), mu or sigma polypeptide (131)		betaine--homocysteine S-methyltransferase (635)	
	asparagine synthetase (glutamine-hydrolyzing) (440)	phosphoserine phosphatase (5723)		cysteine dioxygenase type 1 (1036)	
	acetyl-CoA acetyltransferase 1 (38)	3-hydroxyisobutyryl-CoA hydrolase (26275)		argininosuccinate synthase 1 (445)	
		monoamine oxidase B (4129)		guanidinoacetate N-methyltransferase (2593)	
		3-hydroxy-3-methylglutaryl-CoA synthase 1 (3157)		amidohydrolase domain containing 1 (144193)	
		acyl-CoA dehydrogenase, C-4 to C-12 straight chain (34)		aldehyde dehydrogenase 9 family member A1 (223)	
		pyruvate dehydrogenase (lipoamide) beta (5162)		acetyl-CoA acyltransferase 2 (10449)	
		glutamic-oxaloacetic transaminase 1, soluble (2805)		3-hydroxyanthranilate 3,4-dioxygenase (23498)	
		glutaryl-CoA dehydrogenase (2639)		glutathione S-transferase zeta 1 (2954)	
		oxoglutarate dehydrogenase-like (55753)		pyruvate dehydrogenase (lipoamide) beta (5162)	

Up-regulated gene		carnosine dipeptidase 1 (metallopeptidase M20 family) (84735)<br>cystathionine gamma-lyase (1491)		alcohol dehydrogenase 5 (class III), chi polypeptide (128)<br>nitrilase family member 2 (56954)<br>thiosulfate sulfurtransferase (7263)<br>aldehyde dehydrogenase 2 family (mitochondrial) (217)<br>asparagine synthetase (glutamine-hydrolyzing) (440)<br>kynureninase (8942)<br>agmatinase (79814)<br>glutamate dehydrogenase 1 (2746)<br>aminoacylase 3 (91703)<br>aminocarboxymuconate semialdehyde decarboxylase (130013)<br>alcohol dehydrogenase 7 (class IV), mu or sigma polypeptide (131)<br>glutaryl-CoA dehydrogenase (2639)<br>adenosylmethionine decarboxylase 1 (262)<br>cytochrome P450 family 1 subfamily A member 1 (1543)	
Highly expressed gene	phosphoglycerate dehydrogenase (26227)<br>glutamic-pyruvate transaminase (alanine aminotransferase) (2875)<br>hydroxyacyl-CoA dehydrogenase (3033)	catechol-O-methyltransferase (1312)<br>aldehyde dehydrogenase 2 family (mitochondrial) (217)<br>D-amino-acid oxidase (1610)<br>phosphoglycerate dehydrogenase (26227)<br>glutamic-pyruvate transaminase (alanine aminotransferase) (2875)<br>adenylosuccinate lyase (158)<br>glutamic-oxaloacetic transaminase 2 (2806)<br>hydroxyacyl-CoA dehydrogenase (3033)	fumarylacetoacetate hydrolase (fumarylacetoacetase) (2184)<br>aldehyde dehydrogenase 4 family member A1 (8659)<br>proline dehydrogenase (oxidase) 2 (58510)	homogentisate 1,2-dioxygenase (3081)<br>cytochrome P450 family 1 subfamily A member 2 (1544)<br>fumarylacetoacetate hydrolase (fumarylacetoacetase) (2184)<br>serine hydroxymethyltransferase 2 (mitochondrial) (6472)<br>serine hydroxymethyltransferase 1 (soluble) (6470)<br>cystathionine gamma-lyase (1491)<br>catechol-O-methyltransferase (1312)<br>adenosylhomocysteinase (191)<br>lactate dehydrogenase B (3945)<br>phosphoserine phosphatase (5723)<br>leucine aminopeptidase 3 (51056)<br>catalase (847)<br>glutamic-oxaloacetic transaminase 2 (2806)<br>hydroxyacyl-CoA dehydrogenase (3033)	creatine kinase, muscle (1158)<br>glutamic-oxaloacetic transaminase 2 (2806)<br>3-oxoacid CoA-transferase 1 (5019)<br>glutamic-oxaloacetic transaminase 1, soluble (2805)<br>phosphoglycerate mutase 2 (5224)

Figure 1

Click here to download Figure Figure1.pdf

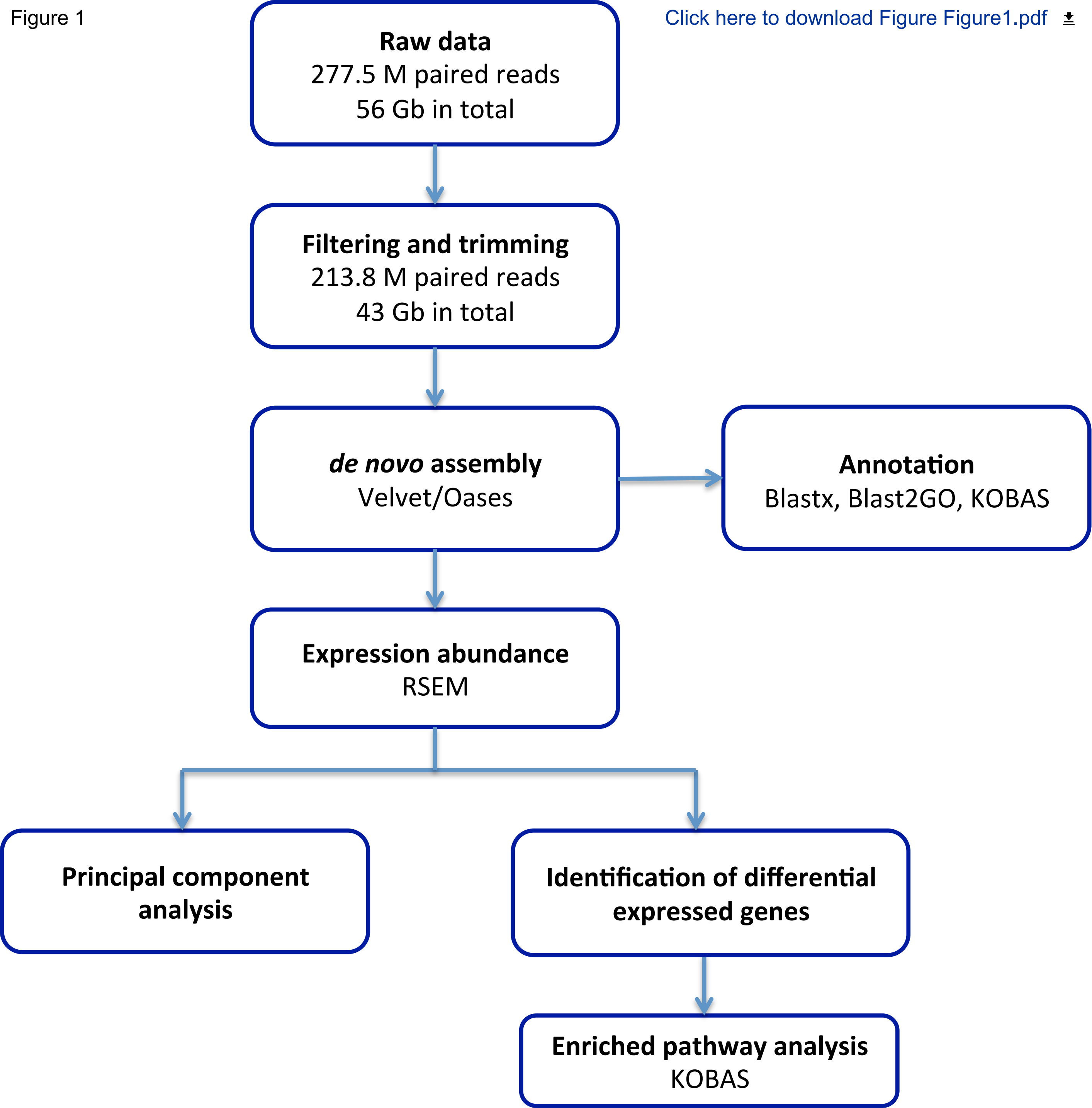

Figure 2

Click here to download Figure Figure2.pdf

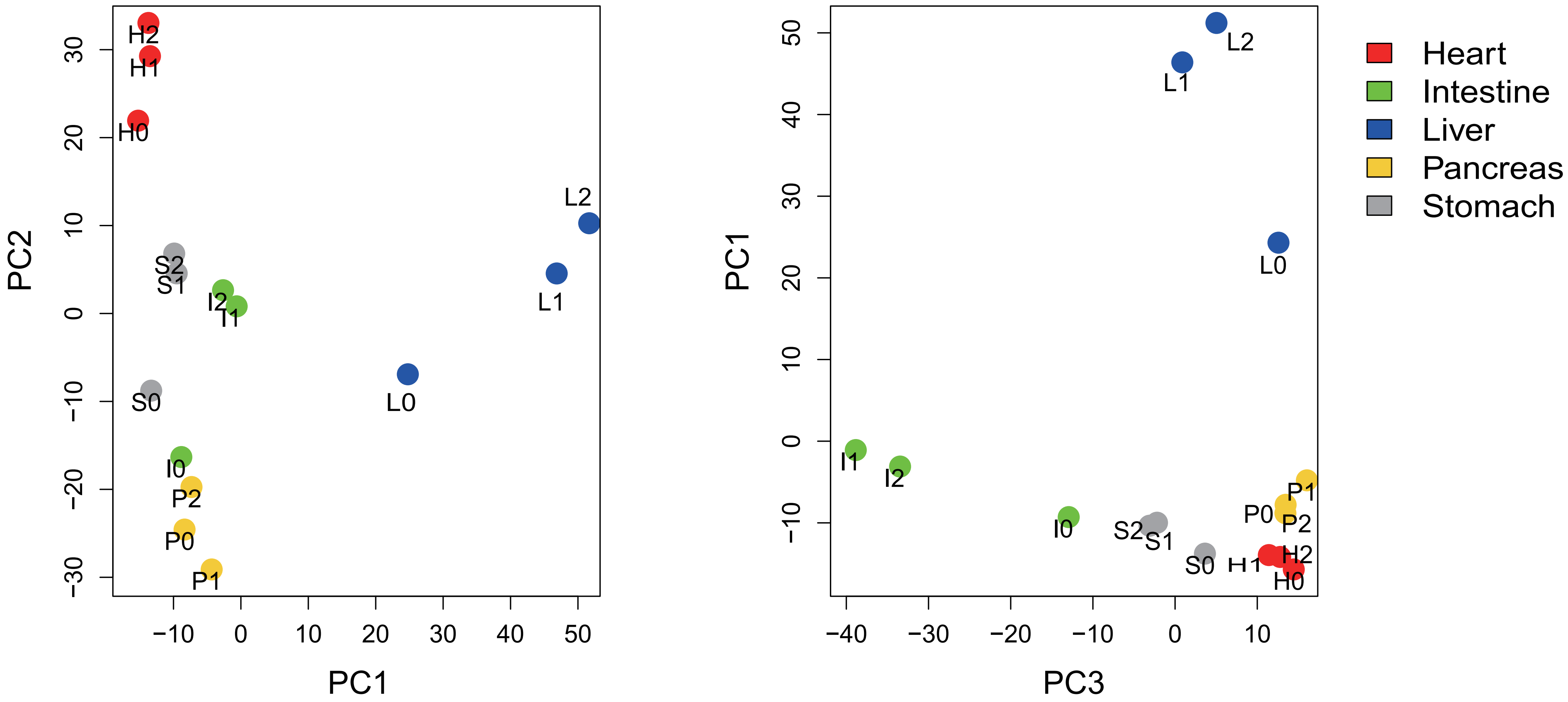

Figure 3

Click here to download Figure Figure3.pdf

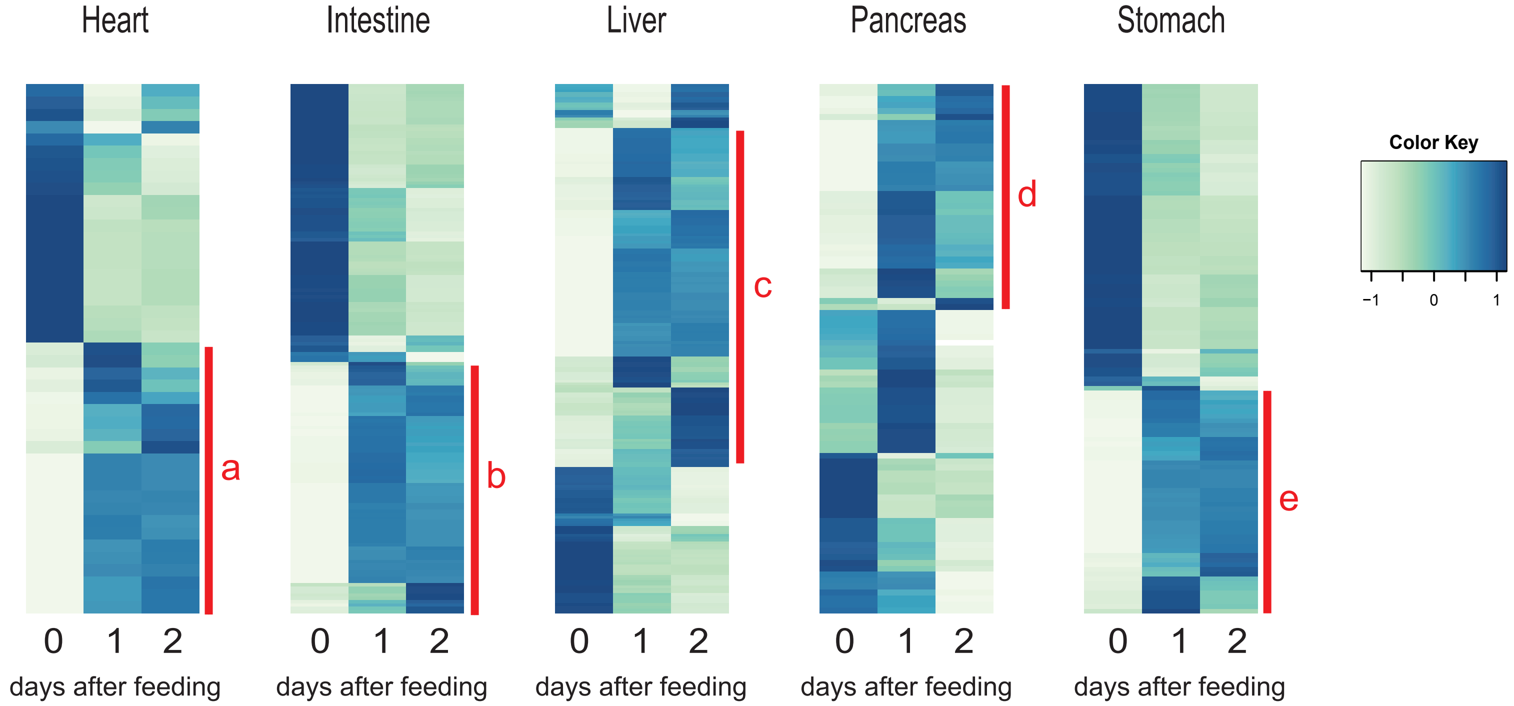

Figure 4

Click here to download Figure Figure4.pdf

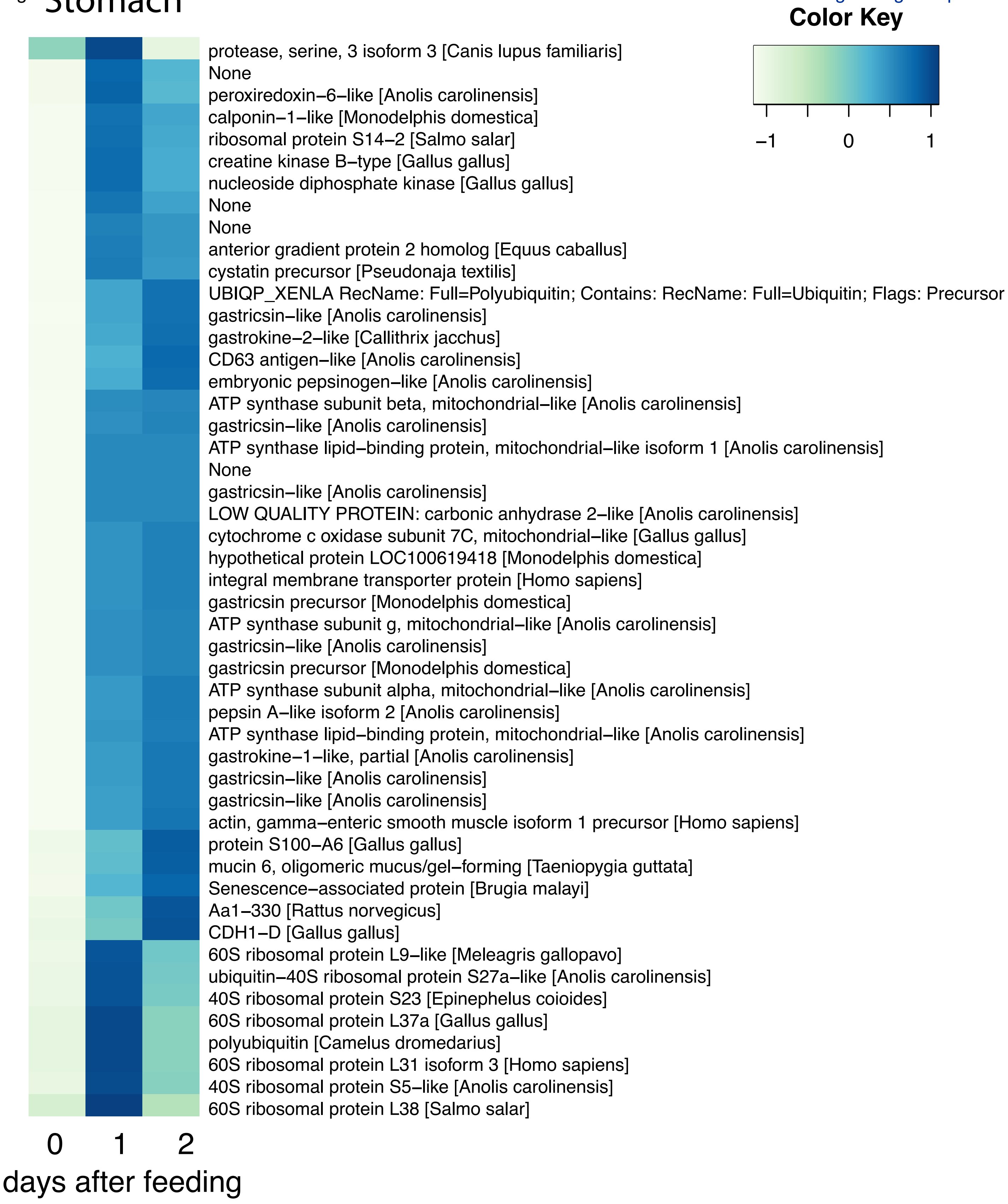

Figure 5

Click here to download Figure Figure5.pdf

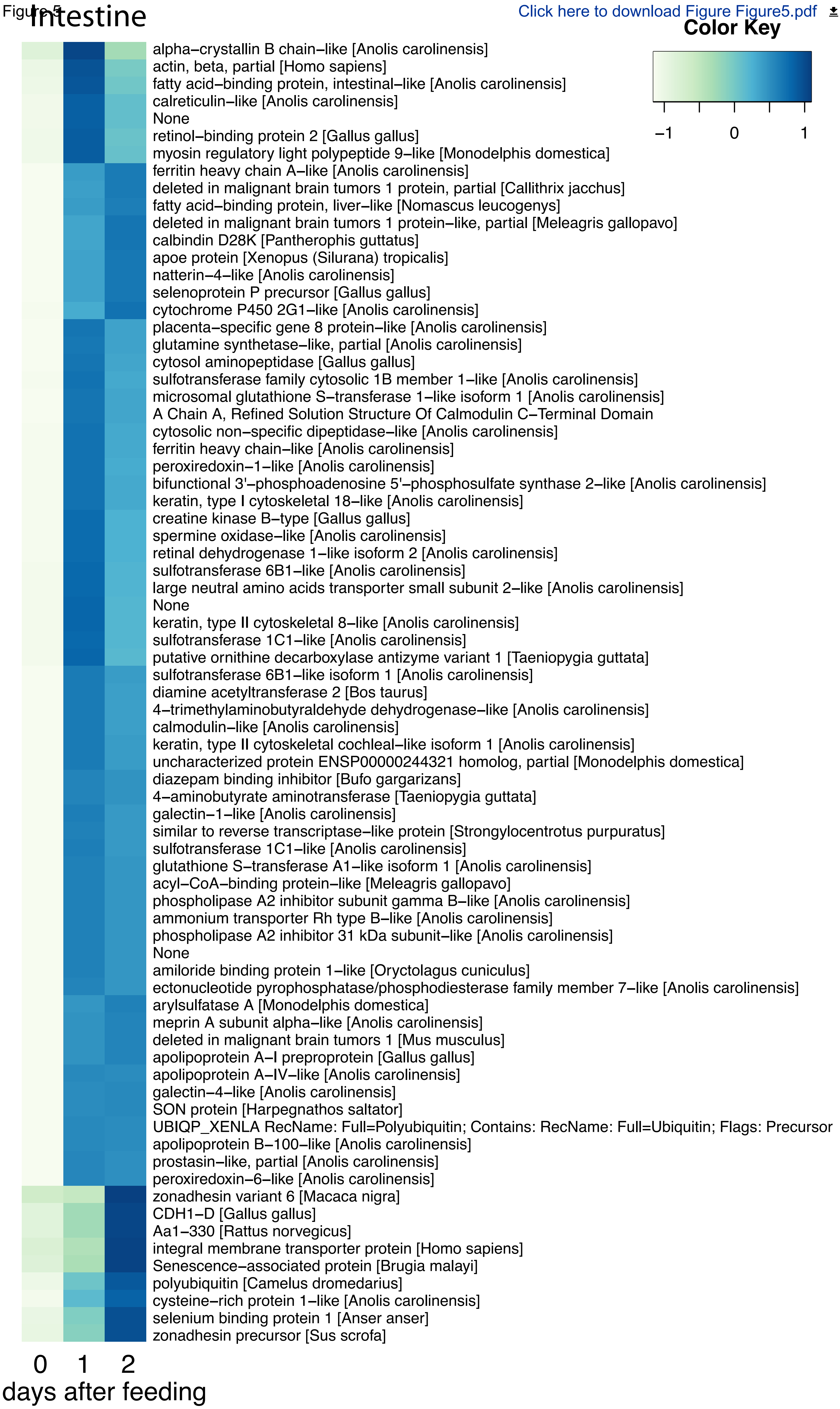

Figure 6

Click here to download Figure Figure6.pdf

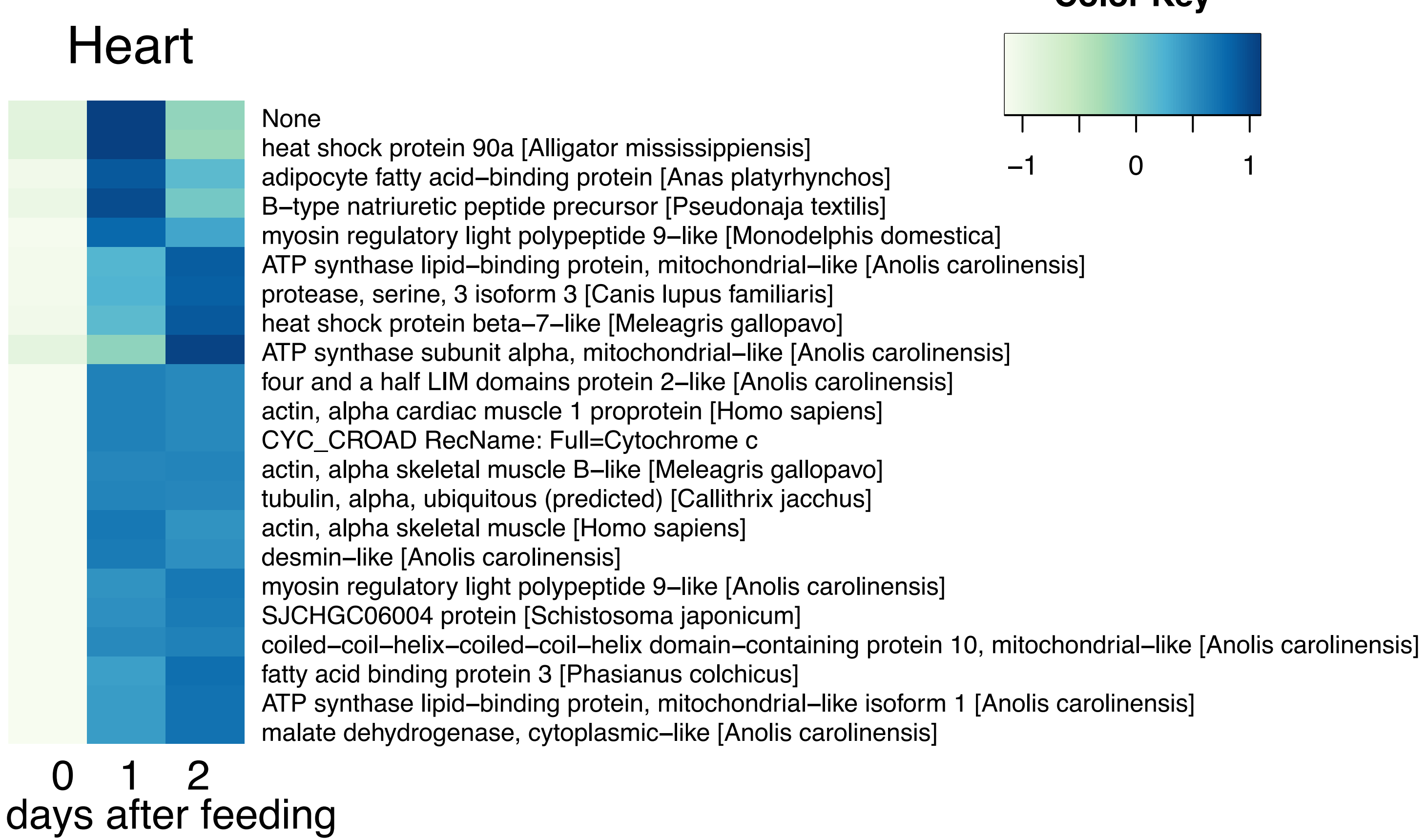

Figure 7

Click here to download Figure Figure7.pdf

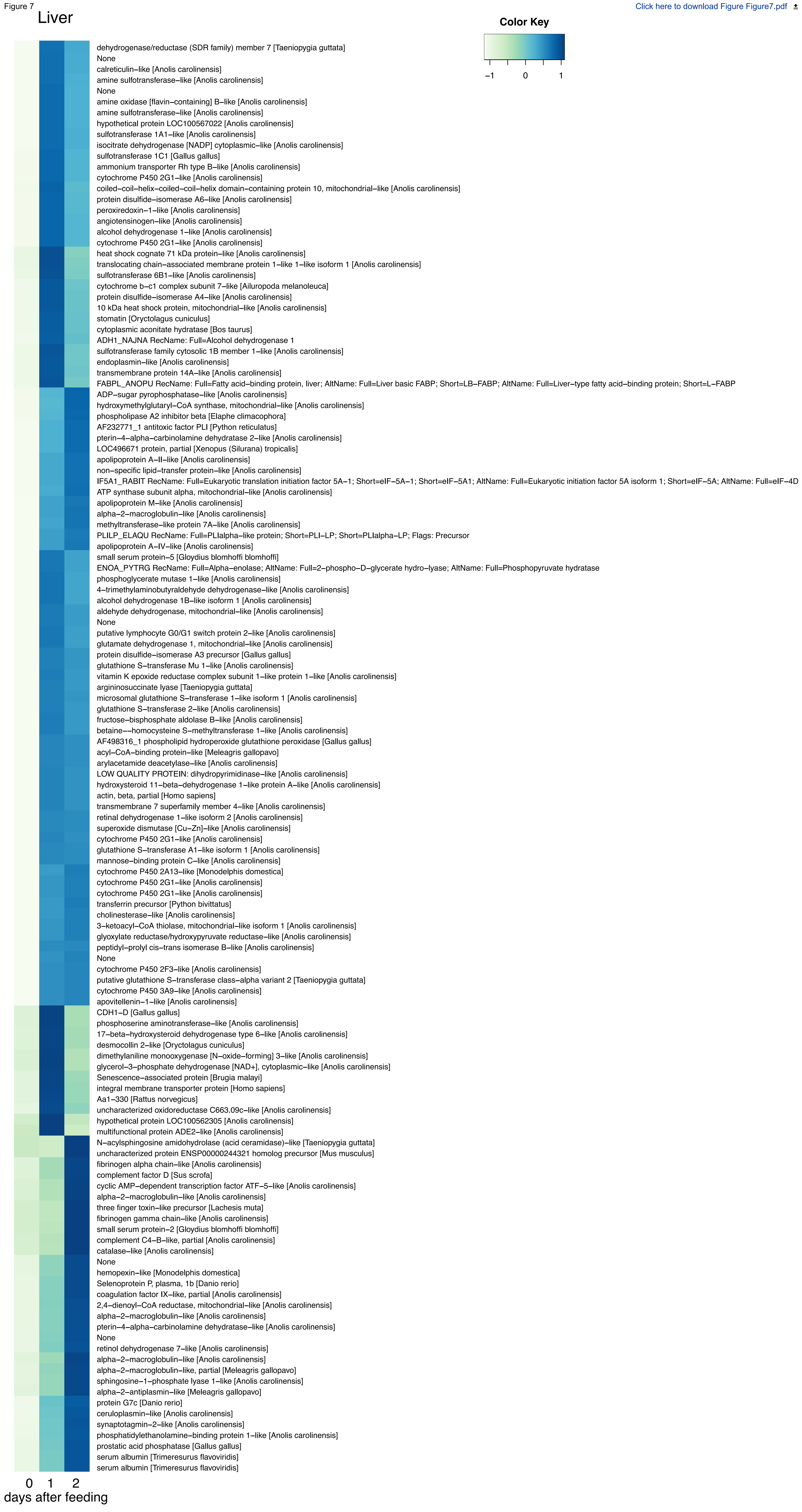

Figure 8

Click here to download Figure Figure8.pdf

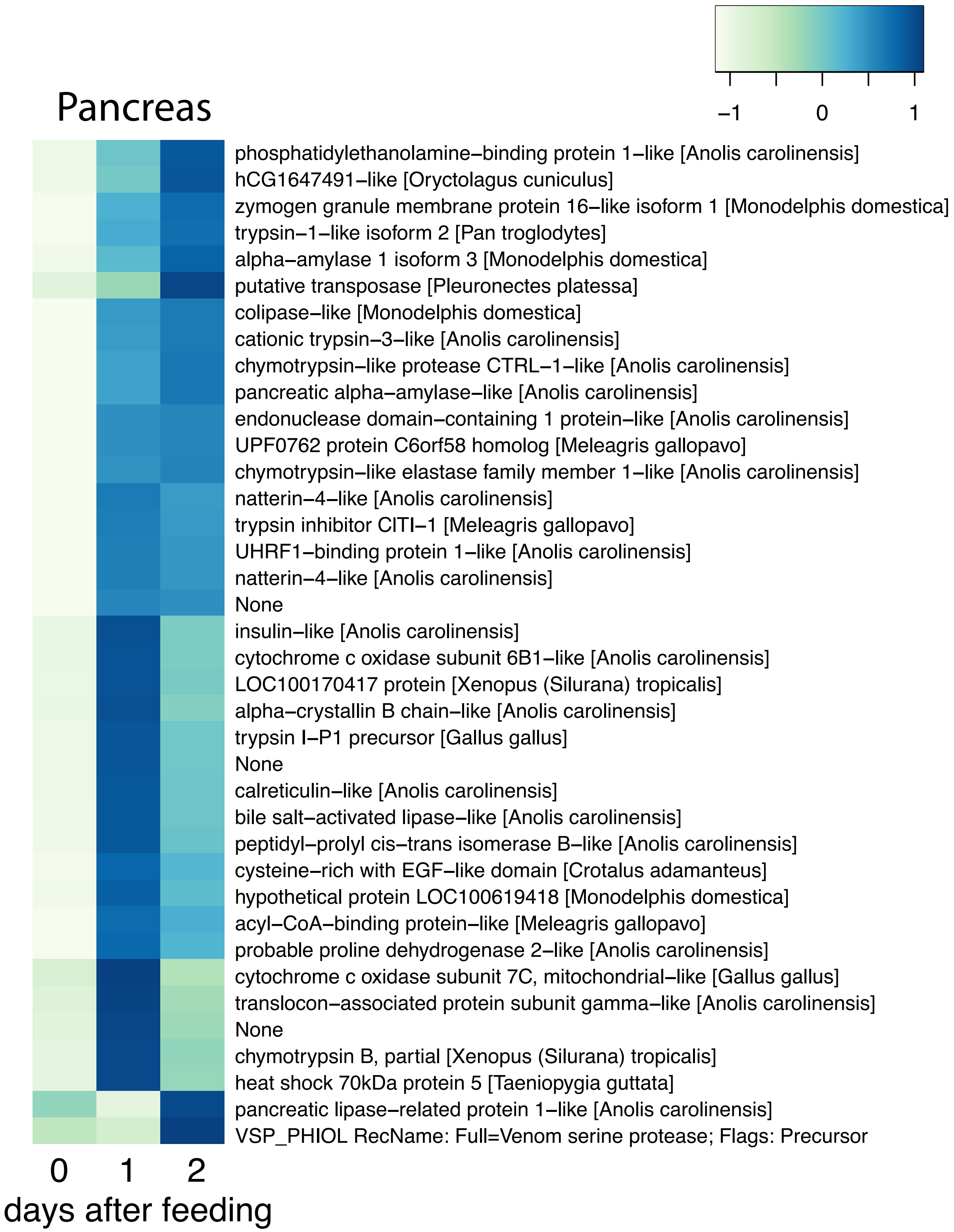

Figure 9

Click here to download Figure Figure9.pdf

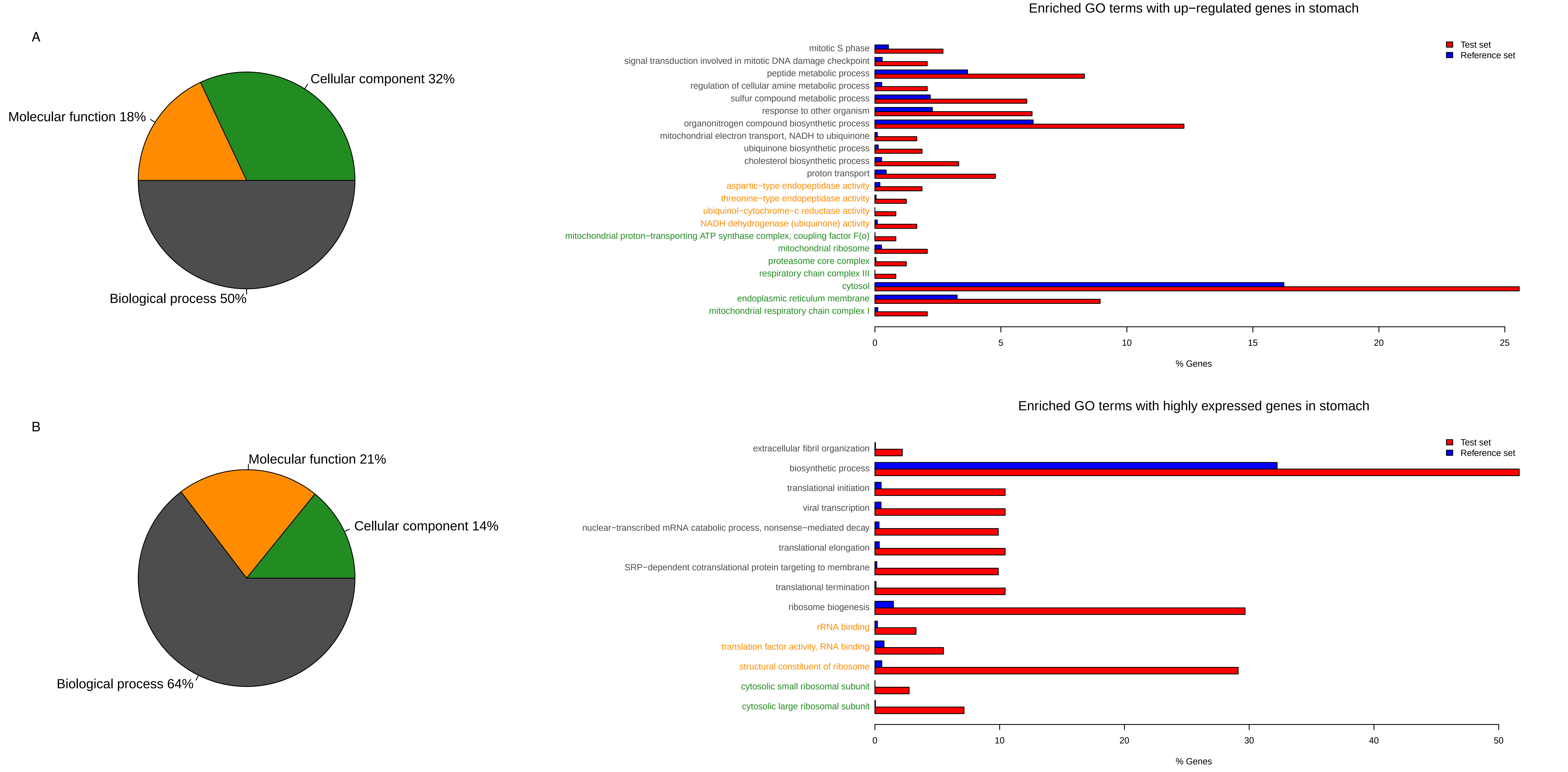

Figure 10

Click here to download Figure Figure10.pdf

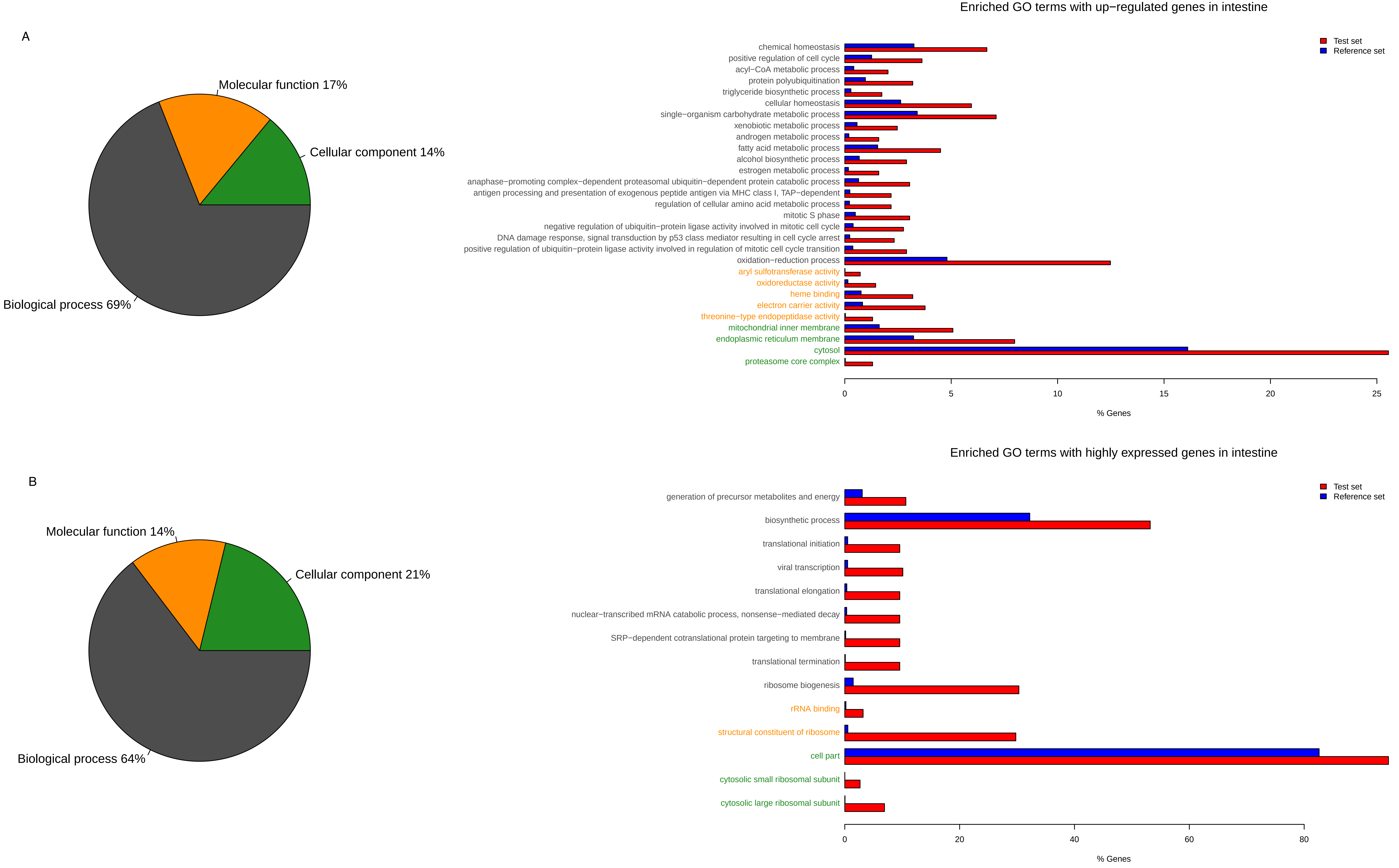

Figure 11

Click here to download Figure Figure11.pdf

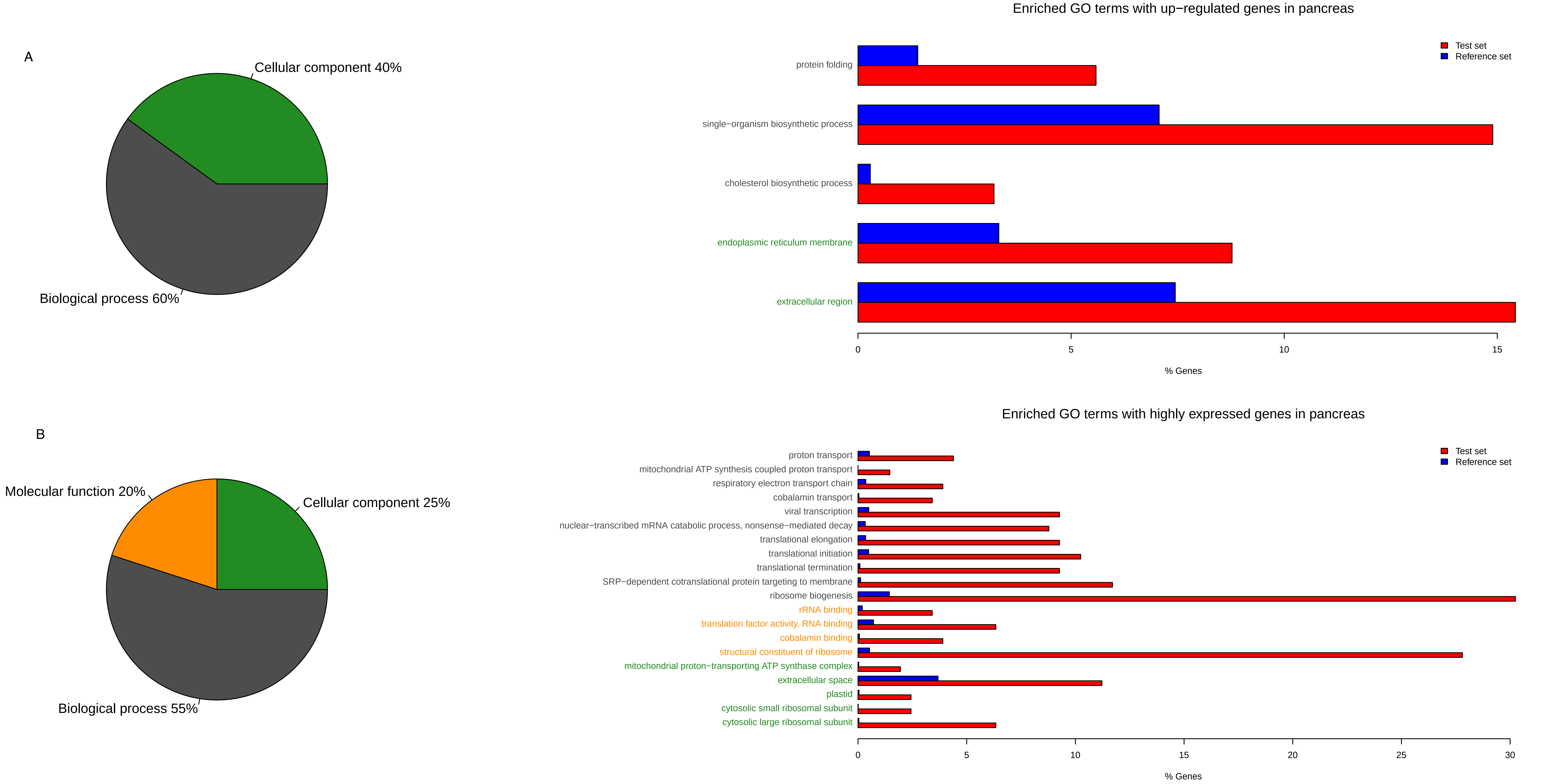

Figure 12

Click here to download Figure Figure12.pdf

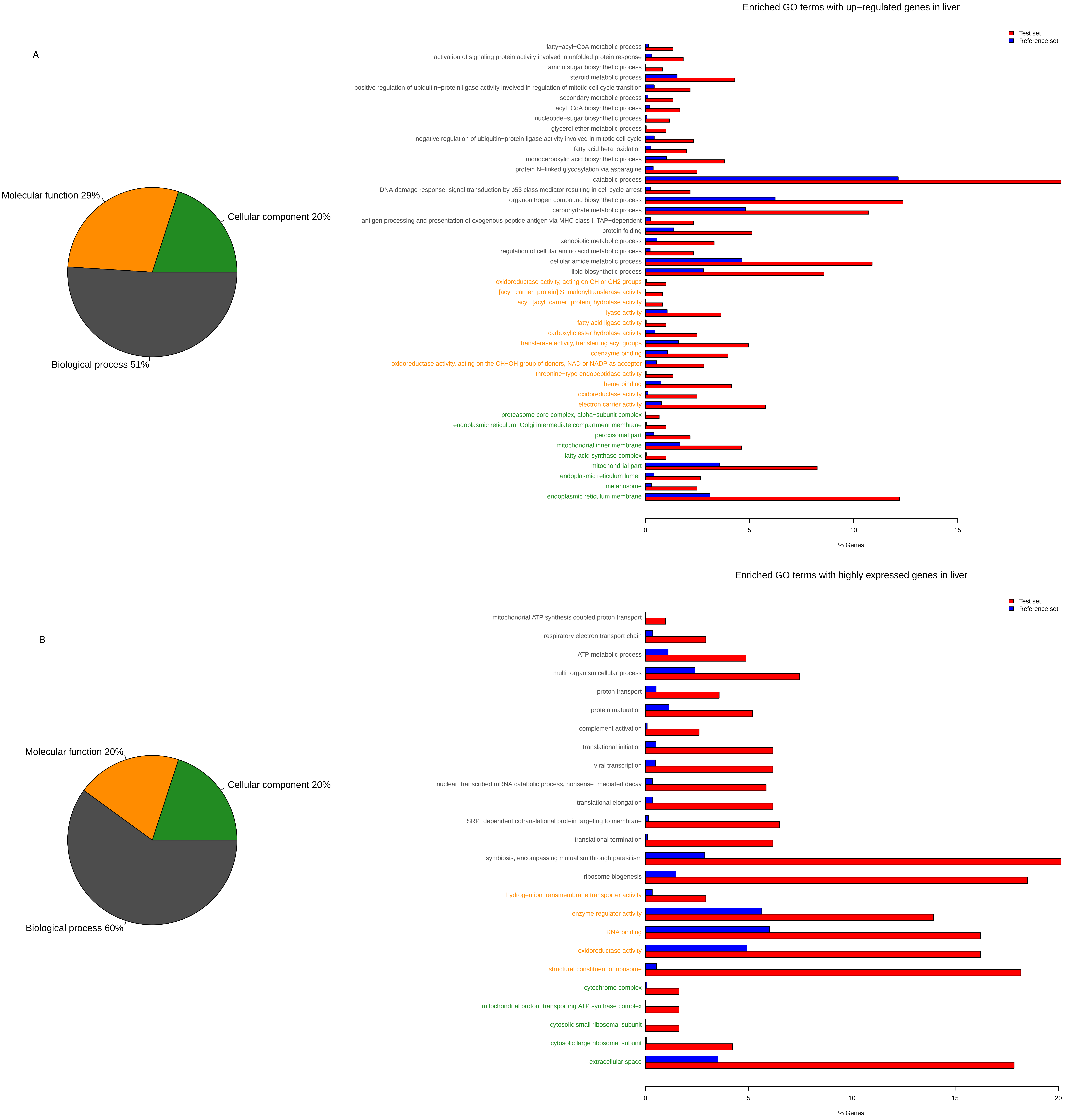

Figure 13

Click here to download Figure Figure13.pdf

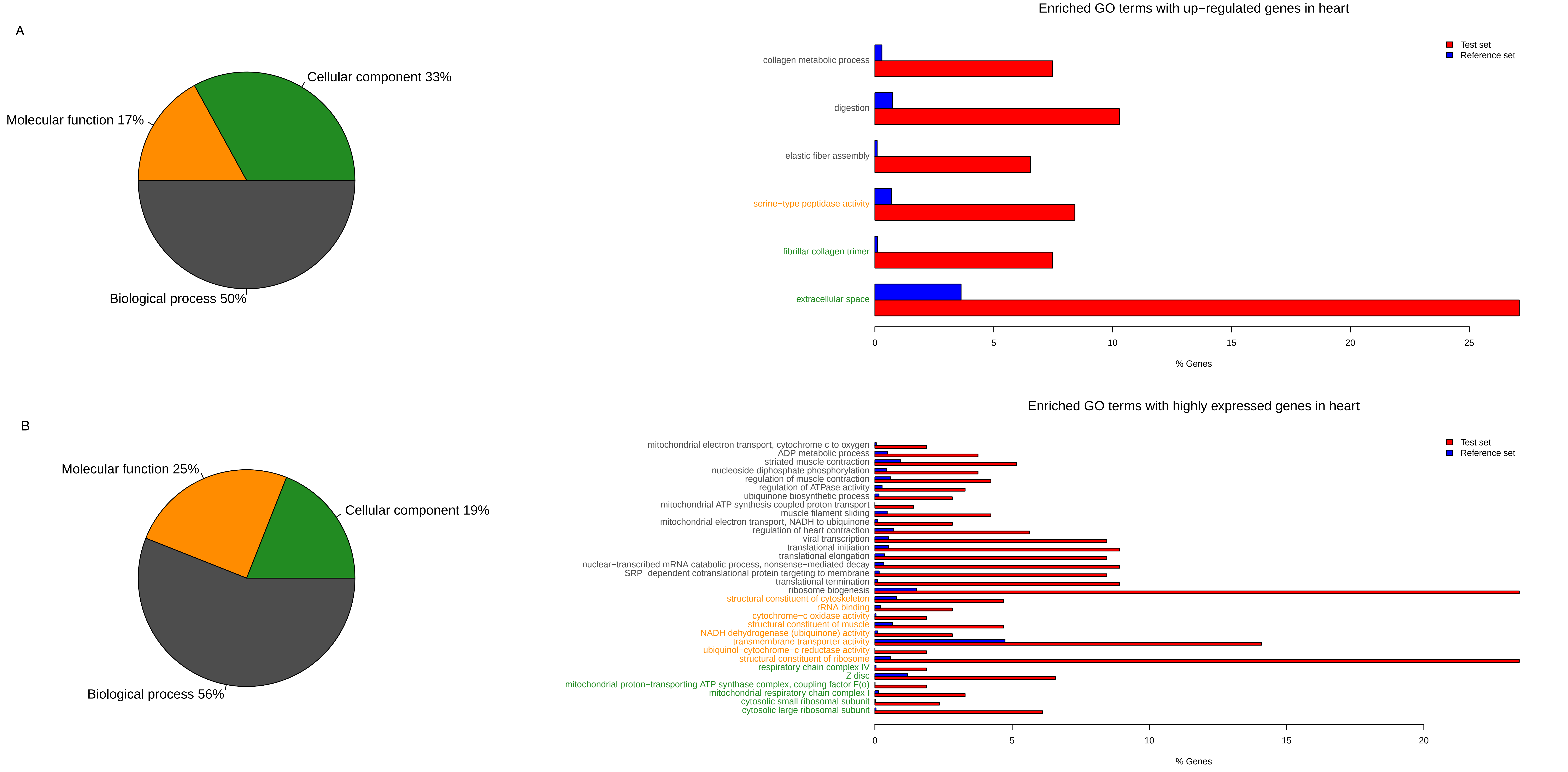

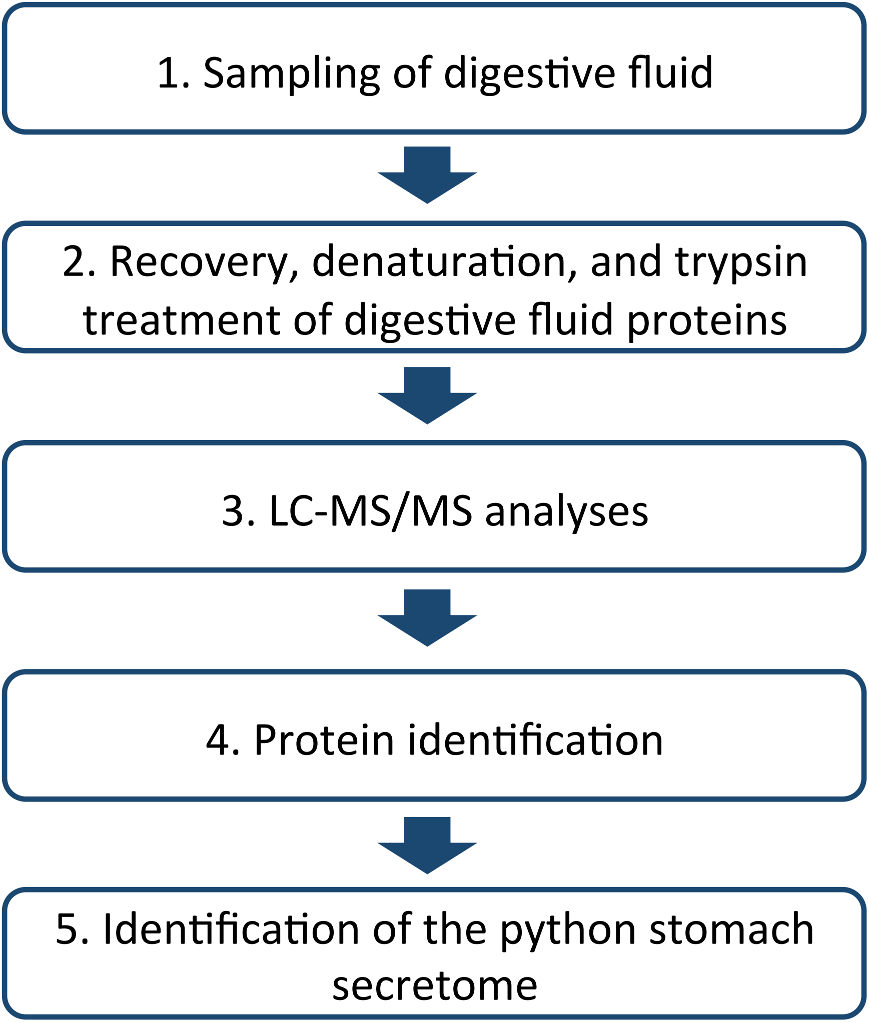
1. Sampling of digestive fluid
2. Recovery, denaturation, and trypsin treatment of digestive fluid proteins
3. LC-MS/MS analyses
4. Protein identification
5. Identification of the python stomach secretome

Figure 15

Click here to download Figure Figure15.pdf

```
m.29547_Py95 Python gastricsin        --------------EE-VERIVLKKGKSLREILKEKGMLEEFMKKYHVDPGLKYRPNEFH 45
m.30181_Py95 Python gastricsin        ----------------------------MREVMKEKGVHEQLL-HRYYDPASKYMN-KFA 30
XP_003220378.1 Anole gastricsin       MKWLIIVLVYLHLSEG-LERVILKRGKSIRENMKEKGVLEEFLKKNHVDPALKYHFNEYN 59
XP_003220377.1 Anole gastricsin       MKWLILAMVCLHLCEGAVIKVPLKKFKSMREVMKEKGVLDEFLKNNKFDPASKYFN-EFA 59
SP|P20142 Human gastricsin            MKWMVVVLVCLQLLEAAVVKVPLKKFKSIRETMKEKGLLGEFLRTHKYDPAWKYRFGDLS 60
                                                                  :** :****:  :::     **. **   .

m.29547_Py95 Python gastricsin        VVYESITSYMSNFYIGEISIGTPPQDFLVVMDTGSSNLWVPSVYCNTAACGNHRRFNPSA 105
m.30181_Py95 Python gastricsin        TGIEPLANYMDMSYYGEISIGTPPQDFLVLFDTGSSNLWVPSVYCQSEPCTNHPLFNPNQ 90
XP_003220378.1 Anole gastricsin       VAYEPITNYLNSYYFGEISIGTPPQNFLVVMDSGSSNLWVPSVYCDTAACAKHNRFSPSA 119
XP_003220377.1 Anole gastricsin       SGYEPLSNYMDMSYYGEISIGTPPQNFLVLFDTGSSNLWVASVYCQSEACTNHPLYNPNQ 119
SP|P20142 Human gastricsin            VTYEP-MAYMDAAYFGEISIGTPPQNFLVLFDTGSSNLWVPSVYCQSQACTSHSRFNPSE 119
                                         *    *:.  * **********:***::*:******* ****::  * .*  :.*.

m.29547_Py95 Python gastricsin        SSTYKNEGQSCTLYYGSGDLTVRMGYDTVRVQNIVVKNQEFGLSEDEPYDPFYYAYFDGI 165
m.30181_Py95 Python gastricsin        SSTYYSNGQTFSIQYGSGSLTGVFGYDTVTIQGISITNQEFGLSETEPGSAFVYSKFDGI 150
XP_003220378.1 Anole gastricsin       SSTYSNSGQTYTLYYGAGDLTVMLGYDTVMVQNIVVTNQEFGLSENEPMTPFYYASFDGI 179
XP_003220377.1 Anole gastricsin       SSTYSTNDQSFSLQYGTGSLTGIFGYDTVTIQGISITSQEFGLSETEPGTNFVYAQFDGI 179
SP|P20142 Human gastricsin            SSTYSTNGQTFSLQYGSGSLTGFFGYDTLTVQSIQVPNQEFGLSENEPGTNFVYAQFDGI 179
                                      **** .. *: :: **:*.**  :****: :* * : .******* **   * *: ****

m.29547_Py95 Python gastricsin        LGMAYPPLAFWGSQTIMQQMVNQGQLSEPIFSFYFSREPTYQYGGELILGGVLDRLFIGE 225
m.30181_Py95 Python gastricsin        LGLAYPSISAGGATTVMQGMMKENLLESAVFSFYLSQQPSSQNGGALVFGGTDPNLYTGQ 210
XP_003220378.1 Anole gastricsin       MGMAYPSLAVGGTATVMQQMLNQGQLSEPIFSFYFSRQPTVQYGGELILGGVDTQLFSGD 239
XP_003220377.1 Anole gastricsin       LGMAYPAISSGGATTVLQGLQQENLLDAPVFGFYLSGNEGS-QGGELALGGVDTNLYSGQ 238
SP|P20142 Human gastricsin            MGLAYPALSVDEATTAMQGMVQEGALTSPVFSVYLSNQQGS-SGGAVVFGGVDSSLYTGQ 238
                                      :*:*** ::   : * :* : ::  *   :*..*:* :     ** : :**.   *: *:

m.29547_Py95 Python gastricsin        ISWAPVTEQMYWQIAIDEFAIGNQATGWCSGGCQAIVDTGTFLLAVPQQYIGTFLNTLGA 285
m.30181_Py95 Python gastricsin        INWAPVTQEMYWQIAIDSFSVNNQTTNWCSQGCQGIVDTGTSLLTVPQNVFGELMQYIGA 270
XP_003220378.1 Anole gastricsin       VSWAPVTREVYWQIGVEEFAIGNEATGWCSEGCQAIVDTGTCQLTIPRQYFDTFLQAVGA 299
XP_003220377.1 Anole gastricsin       IVWTPVTQEAYWQIGIQSFYIGNQITDWCSQGCQAIVDTGTSLLTAPQQVFGELMQYVGA 298
SP|P20142 Human gastricsin            IYWAPVTQELYWQIGIEEFLIGGQASGWCSEGCQAIVDTGTSLLTVPQQYMSALLQATGA 298
                                      : *:***.: ****.::.* :  : : *** ***.******  *: *:: :  :::  **

m.29547_Py95 Python gastricsin        TE-SNYEFVVPCDKVPSMPTITFVINGSKFPLLPSAYIAQ-DGGSCVVAIETTYVPSRNG 343
m.30181_Py95 Python gastricsin        QENSYGEYVVNCDNIQSMPTITFVINGVSFPLSPSGYVLQYSGGSCQVGMMPTYLPSQNG 330
XP_003220378.1 Anole gastricsin       EE-YNGELLVNCNNVQNMPTITFVINGAQFPLPPSAYVAN-NDGYCTVVVEPTYLASQSG 357
XP_003220377.1 Anole gastricsin       EQDGNGQYVVSCSNIQSMPTITFVIGGSSFPLSPSAYVLENNNGYCTVGIMPTYLPSQNG 358
SP|P20142 Human gastricsin            QEDEYGQFLVNCNSIQNLPSLTFIINGVEFPLPPSSYILS-NNGYCTVGVEPTYLSSQNG 357
                                       :    : :* *..: .:*::**:* * .*** **.*: . . * * * :  **:  *:.*

m.29547_Py95 Python gastricsin        QPLWILGDVFLKEYYSVFDLANNRVGFAKSA----------------------- 374
m.30181_Py95 Python gastricsin        QPLWILGDVFLRSYYSVYDLGNNQVGMMPTYLPSQNGQPLWILGDVFLRSYYSV 384
XP_003220378.1 Anole gastricsin       EPLWILGDVFLKEYYSVFDMANNRVGFALSA----------------------- 388
XP_003220377.1 Anole gastricsin       QPLWILGDVFLRSYYSVYDFGNNQVGFAAAA----------------------- 389
SP|P20142 Human gastricsin            QPLWILGDVFLRSYYSVYDLGNNRVGFATAA----------------------- 388
                                      :**********:.****:*:.**:**:  :
```

 Click here to download Figure Figure16.pdf

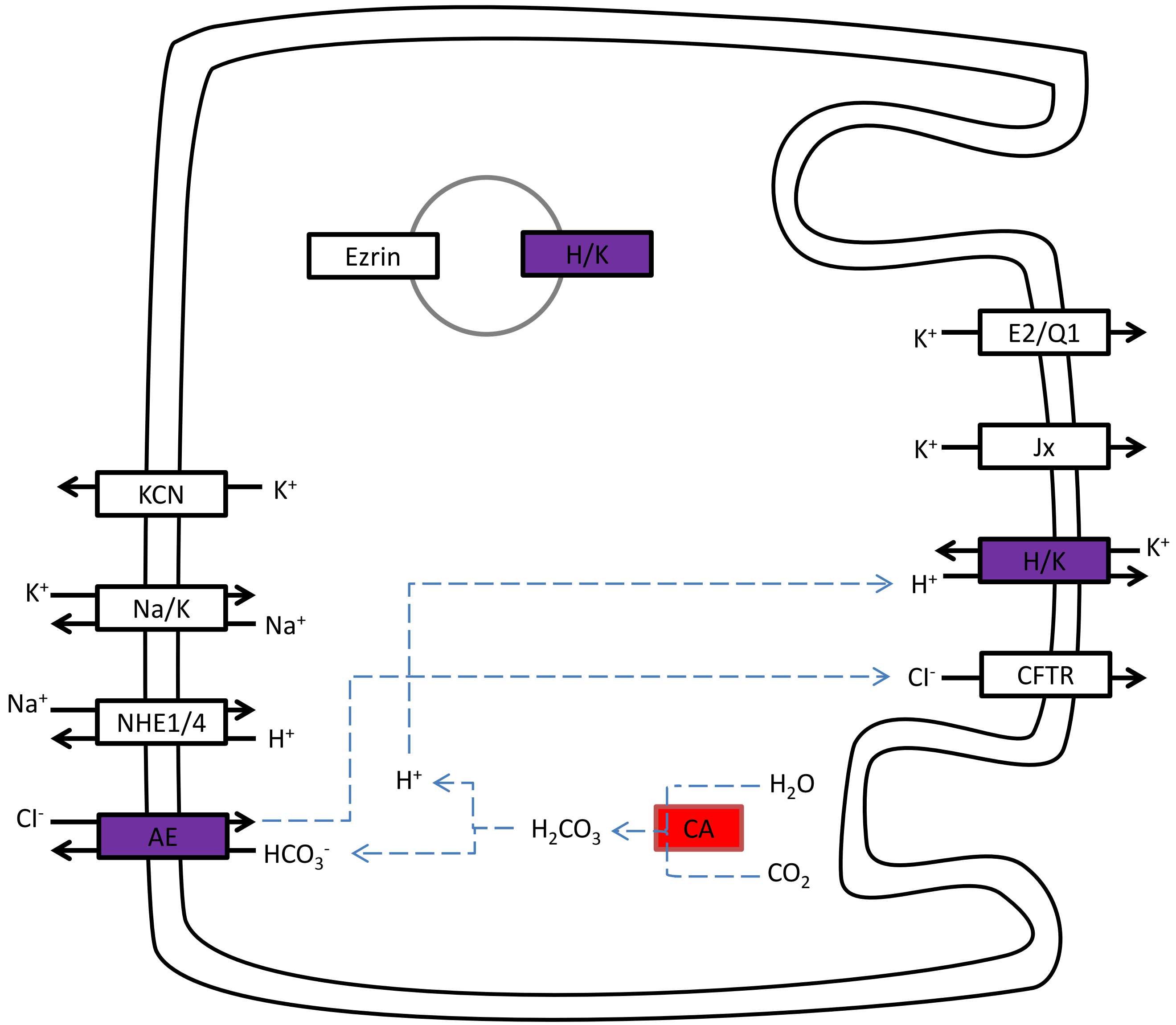

Click here to access/download
Supplementary Material
supplementary document.docx

Supplementary table 4

Click here to access/download
Supplementary Material
TableS4.xlsx

Supplementary table 5

Click here to access/download

Supplementary Material

TableS5.xlsx

Supplementary table 6

Click here to access/download
Supplementary Material
TableS6.xlsx

Supplementary table 7

Click here to access/download
Supplementary Material
TableS7.xlsx

Supplementary table 8

Click here to access/download
Supplementary Material
TableS8.xlsx

Supplementary table 9

Click here to access/download
Supplementary Material
TableS9.xlsx

Supplementary table 10

Click here to access/download
Supplementary Material
TableS10.xlsx

Supplementary table 11

Click here to access/download
Supplementary Material
TableS11.xlsx

Supplementary figure 3

Click here to access/download
Supplementary Material
FigureS3.pdf
