# Supplementary material for: Transcriptome analysis of the response of Burmese python to digestion
Source: Gigascience. 2017 Jul 13;6(8):1–18. doi: 10.1093/gigascience/gix057 (PMC5597892; doi:10.1093/gigascience/gix057)
Supplement: GIGA-D-16-00152_Revision-2.pdf [file gix057_GIGA-D-16-00152_Revision-2.pdf]

Manuscript

Click here to download Manuscript Transcriptome analysis of Burmese python_revision2.docx

Transcriptome Analysis of the Response of Burmese Python to Digestion

Jinjie Duan[1], Kristian Wejse Sanggaard[2,3], Leif Schauser[5], Sanne Enok Lauridsen[4], Jan J. Enghild[2,3], Mikkel Heide Schierup[1,4] and Tobias Wang[4]

1. Bioinformatics Research Center, Aarhus University, Denmark

2. Department of Molecular Biology and Genetics, Aarhus University, Denmark

3. Interdisciplinary Nanoscience Center (iNANO), Aarhus University, Denmark

4. Department of Bioscience, Aarhus University, Denmark

5. QIAGEN Aarhus, Silkeborgvej 2, 8000 Aarhus C, Denmark

Corresponding authors: Jinjie Duan, Mikkel Heide Schierup & Tobias Wang

Email address:

Jinjie Duan: jjduan@birc.au.dk

Kristian Wejse Sanggaard: kristian@wejsesanggaard.dk

Leif Schauser: Leif.Schauser@qiagen.com

Sanne Enok Lauridsen: sanne.lauridsen@inano.au.dk

Jan J. Enghild: jje@mbg.au.dk, ORCID: 0000-0001-9292-9172

Mikkel Heide Schierup: mheide@birc.au.dk, ORCID: 0000-0002-5028-1790

Tobias Wang: tobias.wang@bios.au.dk, ORCID: 0000-0002-4350-3682

Abstract

Background:

Exceptional and extreme feeding behaviour makes the Burmese python (*Python bivittatus*) an interesting model to study physiological remodelling and metabolic adaptation in response to refeeding after prolonged starvation. In this study, we used transcriptome sequencing of five visceral organs during fasting as well as 24h and 48h after ingestion of a large meal to unravel the postprandial changes in Burmese pythons. We first used the pooled data to perform a *de novo* assembly of the transcriptome and supplemented this with a proteomic survey of enzymes in the plasma and gastric fluid.

Results:

We constructed a high-quality transcriptome with 34,423 transcripts of which 19,713 (57%) were annotated. Among highly expressed genes (FPKM>100 in one tissue) we found the transition from fasting to digestion was associated with differential expression of 43 genes in the heart, 206 genes in the liver, 114 genes in the stomach, 89 genes in the pancreas and 158 genes in the intestine. We interrogated the function of these genes to test previous hypotheses on the response to feeding. We also used the transcriptome to identify 314 secreted proteins in the gastric fluid of the python.

Conclusions:

Digestion was associated with an upregulation of genes related to metabolic processes, and translational changes therefore appears to support the postprandial rise in metabolism. We identify stomach-related proteins from a digesting individual and demonstrate that the sensitivity of modern LC-MS/MS equipment allows the identification of gastric juice proteins

that are present during digestion.

Keywords:

Burmese Python, transcriptome, tissue expression, digestion, pathway, proteome

Background

All animals exhibit dynamic changes in the size and functional capacities of bodily organs and tissues to match energetic maintenance costs to prevailing physiological demands [1]. This phenotypic flexibility is particularly pronounced in the digestive organs in animals that naturally experience prolonged periods of fasting, but are capable of ingesting large prey items at irregular intervals. The Burmese python is an iconic example of this extreme phenotype [1]. Many species of pythons easily endure months of fasting, while remaining capable of subduing and ingesting very large meals. In Burmese pythons, digestion is attended by a large and rapid rise in mass and/or functional capacity of the intestine, stomach, liver, heart and kidneys [2-4] in combination with a stimulation of secretory processes and an activation of enzymes and transporter proteins. These physiological responses are associated with a many-fold rise in aerobic metabolism. Hence, the Burmese python is an excellent model to study the mechanisms underlying extreme metabolic transitions and physiological remodelling in response to altered demand [1, 3, 5-10].

The postprandial changes in the morphology and physiology of the intestine, heart and other organs have been described in some detail in pythons [1, 5, 8, 9, 11], but only a few studies [12-14] have addressed the underlying transcriptional changes of this interesting biological response. Transcriptome sequencing technology now allows comprehensive surveys [15, 16], prompting our use of transcriptome sequencing of heart, liver, stomach, pancreas and intestine in snakes that had fasted for one month and those at 24 and 48h into the postprandial period. These organs were chosen because a number of earlier studies have revealed their profound phenotypic changes during the postprandial period [1-4, 17], and they are therefore likely to exhibit large changes in gene expression. Differential gene expression in some of these organs has previously been reported [12-14], but we provide new data on 48h into the

digestive period and the first descriptions of gene expression in the stomach and the pancreas. As the Burmese python reference genome assembly [12] is relatively fragmented (contig size N50 ~10kb), we found it impractical to use re-sequencing approaches and opted instead to use our high coverage data to build a *de novo* transcriptome assembly to identify differentially expressed genes (DEGs). To identify the enzymes involved in the digestion process, we isolated the digestive fluid and characterized the protein composition using a proteomics-based approached. This also allowed us to identify the major hydrolytic enzymes used to digest the large and un-masticated meals.

Analyses

Data summary

277,485,924 raw paired reads (2*101 bp, insert size 180 bp) were obtained from Illumina Hi-Seq 2000 sequencing of 15 non-normalized cDNA libraries derived from five tissues (heart, liver, stomach, pancreas and intestine) at three time points (fasted for one month, 24h and 48h postprandial) and 10 DSN-normalized cDNA libraries (see methods and Supplementary Table S1). After removal of low-quality reads (See methods), 213,806,111 (77%), high-quality paired reads were retained. These reads contained a total of 43,146,073,200 bp nucleotides with a mean Phred quality higher than 37 (Q37). To develop a comprehensive transcriptomics resource for the Burmese python (Fig. 1), we pooled these high-quality reads from 25 libraries for subsequent *de novo* assembly.

De novo transcriptome assembly and evaluation

As short k-mers have a higher propensity to generate misassembled transcripts when using a de Bruijn graph-based *de novo* assembler, such as Velvet [18], we conservatively chose an assembly generated using long k-mers for subsequent analysis, at the cost of some sensitivity regarding assembled isoforms. Thus, balancing key metrics (Supplementary Table S2), we used an assembly based on the longest k-mer = 95 (Table 1), as it had the fewest scaffolds/transcripts (34,423), but represented a very large proportion (74%) of all reads. The scaffold N50 of this assembly was 1,673 bp.

To evaluate the accuracy of the transcriptome assembly, we compared it with the Burmese python reference genome (GenBank assembly accession: GCA_000186305.2) and corresponding gene set in NCBI database using rnaQUAST v1.4.0 [19]. The transcriptome assembly had 34,423 transcripts in total. 34,040 (98%) of these transcripts had at least one

significant alignment to the reference genome, and 31,102 (91%) out of 34,040 were uniquely aligned (Supplementary table S3). Average aligned fraction (i.e. total number of aligned bases in the transcript divided by the total transcript length) was 0.975 (Supplementary table S3). The high concordance between the de novo transcript assembly and the genome reference strengthened our confidence in using the de novo assembly as our reference, and showed that the individual fragments were accurate although the reference genome assembly is fragmented. By aligning assembled sequences back to the reference genome, we reviewed the chimeric assembled sequences which had discordant best-scored alignment (partial alignments that are either mapped to different strands/different chromosomes/in reverse order/too distant) and found 1,974 (5.7%) misassembled (chimeric) transcripts (Supplementary table S3). Considered that some of these sequences could be potentially correct, so we included all sequences in our subsequent analysis, but also provided chimeric sequences in a supplementary FASTA file. The comparison of assembled sequences and reference gene sequences (Supplementary table S3) showed that 26,320 (77.3%) assembled transcripts cover at least one isoform from the reference gene set and the mean fraction of transcript matched is 67.8%, suggesting there is a good concordance but also some differences which can be due to errors in either the reference genome assembly/annotation or our assembly. In addition, we assessed the completeness of our transcriptome assembly with the Benchmarking Universal Single-Copy Orthologs (BUSCO) strategy. Results comprised 55.2% (1,428 out of 2,586) complete BUSCOs, 19.8% (512) fragmented BUSCOs and 25% (646) missing BUSCOs. These results are consistent with a survey [20] of assessment completeness of 28 transcriptomes from 18 vertebrates. In this survey, most of transcriptomes from species with close phylogenetic relationship to snakes contain less than 50% complete BUSCOs and more than 40% missing BUSCOs. Therefore, we conclude the quality of our transcriptome assembly was acceptable.

Transcriptome annotation

19,713 transcripts (57% of 34,423) were annotated using transfer of blastx hit annotation against the non-redundant (nr) NCBI peptide database [21]. To assign proper annotation for each transcript, we chose the first best hit that was not represented in uninformative descriptions (Supplementary Table S4). The most closely related species with an annotated genome, *Anolis carolinensis* was able to annotate 10,704 transcripts (54% of all annotated transcripts). Burmese Python and *Anolis carolinensis* both belong to the reptilian order Squamata, and diverged from each other approximately 160 million years ago [22].

Blast2GO was used to [23] annotate these 19,713 transcripts, of which 16,992 could be assigned to one or more GO terms and their putative functional roles described. The distributions of the most frequently identified GO term categories for biological processes (BP), molecular function (MF) and cellular component (CC) are shown in Fig. S1. Moreover, we used the functionality of InterPro [24] annotations in Blast2GO to retrieve domain/motif information for our transcripts, and 21,023 transcripts were annotated by the InterPro database.

Gene expression analysis and principal component analysis

For comparisons between genes, expression profiles were obtained by mapping high quality reads to the reference transcriptome and the expression level was given by fragments per kilo base per million sequenced reads (FPKM) [25]. For the study of expression profiles, we chose to investigate 1862 highly expressed genes (FPKM $\geq$ 100 in at least one of 15 tissues), as it is known that for highly expressed genes, the biological variation among biological replicates in the same tissue at the same stage is lower than for genes showing low expression levels [26]. The majority (~64%) of these 1862 genes were expressed in all tissues, and only

~18% were expressed solely in one tissue (Supplementary Fig. S2). The liver had the highest number of uniquely expressed genes, which may reflect its particular role in metabolism and excretion of waste products.

We used principal component analysis (PCA) to reveal overall differences in gene expression patterns among tissues and time points within the digestive period. The first three principal components (PCs) accounted for ~58% of the variation (Supplementary Fig. S3). Despite the large overlap in expressed genes (Supplementary Fig. S2), the different tissues exhibited distinct transcriptional signatures shown by the PCA in Figure 2, showing a tendency for 24h to represent an intermediate position between fasting and 48h. Liver, intestine and stomach displayed greater shifts in the PCA plots compared to the heart and pancreas, and the largest changes occurred between fasting and 24h in the stomach and intestine. This fits well with the expectation that the stomach and intestine respond early in digestion [3]. The dramatic changes in gene expression in the liver are also consistent with previous observations in pythons [12].

Pattern of transcriptional responses to feeding

The postprandial response involves thousands of genes and large changes in gene expression. To restrict the analysis of these numerous genes, we used a conservative approach where we selected genes that are both highly and differentially expressed with two strict thresholds (see methods). Application of these two thresholds yielded 43 genes for heart, 206 genes for liver, 114 genes for stomach, 89 genes for pancreas and 158 genes for intestine, respectively, that were differentially expressed in response to digestion (Fig. 3). To illustrate this in greater detail, we enlarged the five sub-clusters with the most prominent increase in expression. These sub-clusters, labelled a - e in Figure 3, are shown with full annotation in Figures 4-8. To unravel the functional implications of these responses, we searched for genes encoding for

proteins involved in processes of tissue re-organization, cellular metabolism and digestion within these sub-clusters for each organ.

GO enrichment analysis and coloured KEGG pathway maps

To gain broader biological insight, in functional annotation analysis we applied a looser threshold set (Table 2) to define DEGs as both maximum FPKM (of three time points) over 10 and fold change (FC) over 4 (along with digestion) and highly expressed genes as both maximum FPKM over 200 and FC below 4. The summary of number of genes differentially expressed during digestion in each tissue is illustrated in Table 3. In each organ, most genes (> 76%) have low expression (max FPKM < 10). Around 1% of the genes are highly expressed (max FPKM ≥ 200). The number of upregulated genes is approximately 3% in each organ, except for the heart where only 0.57% of the genes were upregulated in response to feeding. This suggests that during digestion, the digestive organs, like liver, stomach, intestine and pancreas show more pronounced post feeding response than the heart. To dissect the functions of DEGs, we performed GO enrichment analysis with upregulated genes and highly expressed genes respectively for each organ (Supplementary Figs. S4-S8). As an example, the GO terms most significantly associated with upregulated genes in the stomach were “mitochondrial respiratory chain complex 1”, “endoplasmic reticulum membrane” and “cytosol” (Supplementary Fig. S4A).

To specifically identify the pathways associated with DEGs and highly expressed genes, we mapped genes to KEGG [27, 28] human pathway maps and coloured the mapped entries with trends of gene expression during digestion (Table 2). We identified upregulated genes and highly expressed genes, respectively, involved in three selected pathways (glycolysis/gluconeogenesis, citrate cycle (TCA cycle), and oxidative phosphorylation) for each tissue (Supplementary table S5), and we performed the same identification for two main

pathway categories in the KEGG pathway database (1.3 lipid metabolism and 1.5 amino acid metabolism; Supplementary table S6). The glycolysis/gluconeogenesis pathway, glyceraldehyde-3 phosphate dehydrogenase, showed high expression in all organs.

Identification of the python gastric juice proteome

We identified the secretome of the python stomach during digestion (Fig. 9). The resulting mass spectrometry data (containing 122538 MS/MS spectra) was used to interrogate our python transcriptome database, which included transcriptome from stimulated stomach tissue. In total, 549 python proteins were identified using this approach. Afterwards, all identifications based on a single tryptic peptide were removed, reducing the number of identified python proteins to 314 (Supplementary Table S7).

Five classical types of pepsinogens exist, namely pepsinogen A, B, and F, progastricsin (or pepsinogen C), and prochymosin [29]. Of these, our analyses (Supplementary table S8) show that pythons primarily rely on progastricsin for proteolytic digestion, as the five most abundant proteases identified in the gastric juice are annotated as progastricsin-like. We aligned the six gastricsin-like transcript sequences using webPRANK [30] on amino acid level and calculate the pairwise distance between sequences using Tajima-Nei model (Supplementary table S9) in MEGA7 [31]. The mean pairwise distance 1.16 suggests considerable differences in their sequences, which indicate the presence of numerous different proteins with similar functions. This annotation is based on accession XP_003220378.1 and XP_003220378.1 from *Anolis carolinensis.* Alignment of the python sequences with the two anole sequences, as well as with the well-characterized human gastricsin variant, shows that both the active site residues, as well as cysteine bridges, are conserved. It demonstrates the similarity between these enzymes and suggests that the identified python sequences indeed represent catalytically active proteolytic enzymes

(Supplementary Fig. S9). The last identified pepsinogen-like python sequence (m.31615_Py95) was annotated based on the predicted embryonic pepsinogen-like sequence (XP_003220239.1), also from *Anolis carolinensis.* Here, the annotation originates from an embryonic pepsinogen identified in chicken [32]. This protease was identified in the python's gastric juice with a lower emPAI value than the gastricsin sequences indicating a lower concentration of this enzyme (Supplementary table S8), although the transcript displays the highest concentration of the analysed pepsinogens in the post-prandial period (Supplementary table S9). As the name indicates, it is exclusively expressed during the embryonic period in chickens [32, 33], and phylogenetic analysis of the sequence suggests that its closest homolog, among the classical pepsinogens, is prochymosin [32]. Prochymosin also displays a temporal expression pattern and is, in mammals, mainly expressed in new-born species. However, the identified python embryonic-chicken-pepsinogen homolog does not display a similar development-related temporal expression pattern and is, as shown, produced among adult specimens during digestion. However, this does not exclude the protease also being expressed during the python's embryonic phase.

Identification of prey proteins and the python plasma proteome

Many of the obtained MS/MS spectra were expected to correspond to abundant mice proteins, such as collagen. To facilitate the downstream analyses of python proteins, we produced a list of background proteins related to the prey. Hence, cross examination of the mass spectrometry data with the 16693 mouse protein sequences in the Swiss-Prot database was performed, resulting in the identification of 212 mouse proteins, after removing hits based on single peptides (Supplementary table S10). To produce a list of identified python proteins, most likely present in the digestive fluid samples due to blood contamination during collection, we characterized the python plasma proteome. The most abundant plasma proteins

are produced by the liver. Consequently, our python transcriptome sequence database, which encompasses liver transcriptomes, is expected to contain the protein sequences of the python plasma proteins. Thus, our python plasma LC-MS/MS data was used to interrogate our python sequence database. It provided an overview of the most abundant python plasma proteins in Supplementary table S11. In total, 64 plasma proteins were identified with minimum two tryptic peptides. We observed a limited correlation R^2=0.13 fitted with a linear model (Supplementary table S11) between these abundant (based on emPAI) plasma protein expression and corresponding mRNA expression levels (based on FPKM value at 1 day post-feeding in liver). One protein that stands out is the anti-haemorrhagic factor cHLP-B (m.27_Py95), which appeared in high concentrations in the plasma of these snakes. This is a protease inhibitor of the haemorrhagic-causing metalloproteinases present in snake venom and these inhibitors have previously been purified from serum of venomous snakes [34, 35] and have been proposed to inhibit deleterious actions of venom enzymes in non-venomous snakes [36]. It is, however, also possible that it is an ancestral gene with a function not related to venom production.

Identification of the python stomach secretome

To identify the python stomach secretome, the list of python proteins, identified in the digestive fluid (Supplementary table S7) was analysed further. We assumed no overlap between abundant plasma proteins and proteins secreted by the stomach. Thus, plasma proteins, identified in the gastric juice, were assumed to be contaminations from blood and therefore the 64 identified plasma proteins were, when present, removed from the list. Subsequently, python proteins that most likely were identified based on prey proteins homology (*e.g.,* mouse collagens and keratins, as well as conserved intracellular household proteins) were removed. These two steps reduced the list of proteins identified in the stomach

samples from 314 to 114 proteins (Supplementary table S12). It cannot be excluded that a few proteins belonging to the python stomach secretome also were removed.

To identify the secretome, the 114 identified proteins were manually analysed as described in the method section (Supplementary table S12). In addition to household proteins, the identified intracellular proteins also included intracellular stomach-specific proteins (*e.g.* the stomach specific calpain 9 cysteine protease [37]), underlining the specificity of the proteomics analysis. In total, 37 proteins constituted the putative python stomach secretome (Supplementary table S8). These could be divided into 18 gastric mucosal-related proteins (e.g. mucin homologous and gastrokine), seven proteolytic enzymes (mainly pepsin homologous), four other hydrolytic enzymes (e.g. phospholipases), and eight other proteins (e.g. gastric intrinsic factor) (Supplementary table S8). Here, we identify stomach-related proteins from a digesting individual and thereby demonstrate that the sensitivity of modern LC-MS/MS equipment allows the identification of gastric juice proteins that are present during digestion.

Discussion

As a primary motivation, we wished to describe the temporal changes in gene expression in the visceral organs of Burmese pythons during the transition from fasting to digestion, and identify key regulatory genes and pathways responsible for the pronounced tissue restructuring, increased metabolism and the increased functional capacity during the postprandial period. We achieved these goals by identifying the biochemical and physiological roles of highly expressed genes with increased expression during digestion and by using KEGG analysis of the specific pathways underlying physiological responses known to be stimulated by digestion. We also present GO enrichment analyses of both up-regulated genes and highly expressed genes in all organs (Supplementary Figs. S4-S8), showing that "biological process" is the most common enriched category.

The influence of digestion on gene expression profiles in heart, liver, kidney and small intestine has been studied previously in pythons [12-14]. These earlier studies reported thousands of genes being either up- or downregulated within the first day of digestion [12-14], and we confirm these substantial changes in gene expression at 24h and 48h. However, we merely identified hundreds of genes, probably because we selected a more stringent threshold for defining differential expression. Given the differences in the selection thresholds and analysis strategies for differential expression and differences in the times of sampling, it is difficult to make a direct comparison between our study and that of Castoe et al (2013). Nevertheless, for heart, liver and small intestine, both studies have determined a number of upregulated genes at 24h, where we identified 15, 93 and 61 upregulated genes, respectively. Comparing upregulated genes between two studies (see supplementary material for detailed method and results), we found in liver more than half of the 93 upregulated genes identified in our study were also identified as upregulated genes by Castoe et al (2013).

However, there was less overlap for the heart and small intestine. These differences may be due to the use of different quantification methods for gene expression in the two studies, but may also be a result of the limited biological replicates in our study. Nevertheless, genes identified as being upregulated in both studies, can be referred to with high confidence.

Physiological interpretation of the upregulated genes in the stomach

The considerable changes in gene expression in the stomach were reflected in a pronounced rise in expression of ribosomal 40S and 60S proteins (Fig. 4) which is likely to have attended the rise in protein synthesis required for the marked transition from a quiescent fasting state to the activated digestive state. This is also supported by the presence of ribosomal functions in the enriched GO analysis of the highly-expressed genes in the stomach (Supplementary Fig. S4B). During fasting, gastric acid secretion and presumably also the secretion of digestive enzymes and lysozymes, is halted, such that the gastric fluid has a neutral pH, whilst ingestion of prey is followed by an immediate activation of gastric acid secretion [38, 39]. The stimulation of the secretory actions of the stomach is attended by an increased mass of the stomach, where particularly the mucosa expands within the first 24h [40].

The KEGG analysis, however, shows that the genes encoding for the gastric H,K ATPase, the active and ATP consuming ion-transporter responsible for gastric acid secretion, are highly expressed in fasting animals, and not additionally elevated in the postprandial period (Fig. 10). This strongly indicates that the enzymatic machinery for gastric acid secretion is maintained during fasting, a trait that may enable fast activation of acid secretion, at modest energetic expenditure, to kill bacteria and match gastric pH to the optimum value for pepsin. This interpretation is consistent with a number of recent studies indicating a rather modest contribution of gastric acid secretion to the specific dynamic action (SDA) response in pythons [41, 42], but we also did observe a high prevalence of ATP synthase subunits (Fig.

4) amongst the highly upregulated genes, which does indicate a rise in aerobic metabolism (see also supplementary Fig. S4). Furthermore, the upregulation of the gene encoding for creatine kinase (Fig. 4) indicate increased capacity for aerobic respiration required costs of acid secretion and the stimulation of the accompanying gastric functions. It has been proposed that gastric processes account for more than half of the rise in total metabolism during digestion [38], and aerobic metabolism of isolated gastric strips *in vitro* increased during digestion [43]. However, while metabolism of the stomach certainly must increase during the postprandial period, more recent studies indicate a considerably smaller contribution of gastric acid secretion to the total SDA response, meaning that gastric acid comprises considerably lower than 50% of the SDA [41, 42, 44].

Our KEGG analysis also showed a large rise in expression of the gene encoding for carbonic anhydrase (Fig. 10), the enzyme that hydrates CO_2 and provide protons for gastric acid secretion. Gastric acid secretion, therefore, does not appear to undergo transcriptional regulation, but is likely to involve translocation of existing H,K ATPases in vesicles from intracellular vacuoles to the apical membrane of the oxyntopeptic cells that are responsible for both gastric acid secretion as well as the release of pepsinogen in reptiles [45]. An activation of the processes involved in vesicle transport is further supported by increased transcription of the gene encoding for CD63 (Fig. 4), which belongs to the tetraspanin family and mediates signal transduction events.

In contrast to acid secretion, expression of several genes encoding digestive enzymes (embryonic pepsinogen-like, gastricsin precursor and gastricsin-like peptides) (Fig. 4) were upregulated, which is consistent with *de novo* synthesis of the enzymes responsible for gastric protein degradation. Also, there was good overlap between the upregulation of the relevant genes encoding the proteins identified in the stomach secretome, such as gastrokines, pepsin

homologs, phospholipases and gastric intrinsic factor (Supplementary table S8). In this context, it is also interesting that mucin 6 (Fig. 4), the gene coding for the large glycoprotein (gastric mucin) that protects the gastric mucosa from the acidic and proteolytically active chyme in the stomach lumen was upregulated. Thus, as gastric acid secretion is activated, probably in response to increased levels of the gastrin as well as luminal factors, there is an accompanying activation of the protective mucus layer that prevents auto-digestion of the gastric mucosa. It is also noteworthy that the genes for both gastrokine 1 and 2 were upregulated during digestion (Fig. 4). Gastrokines are constitutively produced proteins in the gastric mucosa in mammals and chickens, and while their physiological function remains somewhat elusive, they appear to be upregulated during mucosal remodelling in response to inflammation (*e.g.*, in connection with ulcers) and often downregulated in cancers. Thus, it is likely that the gastrokines are involved in regulating the restructuring of the mucosa during digestion in pythons.

In addition to analysing the gene expression profiles of the stomach, we also used a proteomics approach, assisted by our python transcriptome sequence database, to identify the hydrolytic enzymes in the gastric juice secreted during digestion. We identified python proteins on a complex background of highly abundant mice proteins. Thus, the digestive enzymes secreted by the pancreas are probably functionally similar to known hydrolytic enzymes from other species.

We hypothesized that relatively aggressive proteolytic digestive enzymes in the gastric juice facilitate digestion of large and un-masticated whole prey items [8]. In our analysis, six out of the seven identified proteolytic enzymes were pepsinogen homologs (Peptidase subfamily A1A), and these were also the most abundant hydrolytic enzymes in the gastric juice according to the emPAI values (Supplementary table S8). It is likely that other

pepsinogen isoforms exist in the gastric juice, as our approach predominantly targeted the most abundant proteolytic enzymes. The importance of the proteomics-identified pepsinogens was also substantiated by the transcriptomics data (Supplementary table S9). Here, we found that the six different pepsinogens were upregulated between 2.2 and 22.2 fold from the fasting animals to 48 hours after ingestion of mice. On average the pepsinogen transcripts were upregulated 10.7 fold. It supports that these proteases play a substantial role in the aggressive digestion process performed by the python.

Our proteomic analysis also suggested the identification of the pepsinogens as the major digestive proteolytic enzymes, similar to in all other vertebrate species. Thus, our results indicate that the pepsinogen is not unique (with respect to protease class) and hitherto uncharacterized proteases do not facilitate the aggressive digestion process. Instead, pepsins, homologous to pepsins among other species, digest the intact swallowed prey. As in other vertebrates, pythons have a low gastric pH during digestion [38, 42], and it is likely that these pepsins variants are among the most effective and aggressive pepsins identified so far and our sequence information facilitate future cloning, expression, and characterization of these potentially industrial relevant enzymes.

Physiological interpretation of the upregulated genes in the intestine

The small intestine of pythons undergoes a remarkable and fast expansion during digestion where both wet and dry mass more than double within the first 24 hours. The expansion stems primarily from increased mucosal mass, achieved by swelling of the individual enterocytes [46], while the smooth muscle in the gut wall is much less responsive [47]. Earlier studies on gene expression profiles during digestion in the python intestine revealed massive upregulation of more than one thousand genes, commencing within the first six hours after ingestion [12, 13]. Importantly, this previous study [13] identified a number of

genes that are likely to be involved in the restructuring of the microvilli, cell division and apoptosis, as well as brush-border transporter proteins. In line with these earlier findings, our GO enrichment analysis also highlights functions pertaining to mitotic cell division, which supports a contribution to growth by hyperplasia faster cell turnover (Supplementary Fig. S5). The expansion of the individual enterocytes is accompanied by pronounced elongation of the microvilli [48] and the resulting rise in surface area of the intestinal lining is accompanied by a ten-fold increase in intestinal transport capacity for amino acids and other nutrients [1, 4, 49].

Earlier studies provided strong evidence for an upregulation of genes coding for nutrient transporter proteins, such as D-glucose, L-proline and L-leucine [13]. In this context, it is noteworthy that there were no nutrient transporters amongst the highly expressed and upregulated genes in the intestine (Fig. 5), but our KEGG analysis nevertheless showed increased expression of the serosal L-type amino acid transporter. Clearly, it would be worthwhile to quantitatively analyse the extent to which *de novo* synthesis of the various nutrient transporters, particularly those for amino acids, is increased during digestion and how much such synthesis contributes to absorptive capacity. It would seem adaptive if many of the transporters merely have to be activated, either by insertion within the luminal membrane or exposed as the enterocytes expand: an energetically cheap manner of matching intestinal performance to the sudden appearance of nutrients in the intestine after a meal. The GO enrichment analysis also pointed to an enrichment of various metabolic processes during digestion, particularly for the upregulated genes (Supplementary Fig. S5). It is noteworthy that the expression of genes coding for glutathione S-transferase, peroxiredoxin and selenoprotein increased during digestion (Fig. 5). These three proteins are involved in cellular defence, particularly as antioxidants as a likely protection from the reactive oxygen species that result from increased aerobic metabolism.

There is consensus that the anatomical and structural responses underlying this phenotypic flexibility of intestinal function occur at modest energetic expenditure [17, 38, 50], but our expression profile does show increased expression of the gene coding for cytochrome P450, pointing to increased aerobic and mitochondrial metabolism. An increased expression of the genes involved in oxidative phosphorylation was also reported in earlier studies on pythons [12, 13]. This rise in metabolism may be driven primarily by the massive rise in secondary active transport to absorb the amino acids and smaller peptides rather than the structural changes [50]. Nevertheless, the structural changes may be reflected in increased expression of galectin 1 (Fig. 5), which mediates numerous functions including cell–cell interactions, cell–matrix adhesion and transmembrane signalling.

Fig. 5 reveals the importance of lipid absorption and its subsequent transport by the cardiovascular and lymph systems, and it is also possible that several of the expressed proteins play a role in the incorporation of lipid droplets within the enterocytes. Thus, the presence of numerous apolipoproteins, and their precursor apoe protein, amongst the list of highly expressed and highly expressed genes (Fig. 5) are probably required to transport the absorbed lipids in plasma and lymph, but the apolipoproteins could also act as enzyme cofactors, receptor ligands, and lipid transfer carriers in the regulation of lipoprotein metabolism and cellular uptake. The presence of diazepam-binding inhibitor (Fig. 5), a protein involved in lipid metabolism and under hormonal regulation mostly within nervous tissue, is also likely to reflect the increased lipid absorption and metabolism in the postprandial period, and there was also a rise in phospholipases (Fig. 5) that are likely to be involved in lipid degradation. Also, the capacity for protein metabolism clearly increased in the intestine during digestion (seen in *e.g.*, meprin A and endopeptidase that cleave peptides, as well as 4-aminobutyrate aminotransferase, 4-trimethylaminobutyraldehyde dehydrogenase and diamine acetyltransferase) and there was a rise in the ammonium transporter protein Rh

(Fig. 5). Finally, a number of proteins involved in calcium uptake and metabolism, such as calbindin and calmodulin (Fig. 5), could be important to handle the break-down of the bone in a normal rodent, and it was recently shown the enterocytes of pythons already contain small particles of bone at 24 hours after ingestion [48].

Physiological interpretation of the upregulated genes in the heart

The large metabolic response to digestion is accompanied by a doubling of heart rate and stroke volume of the heart such that cardiac output remains elevated for many days during digestion [51, 52]. This cardiovascular response plays a pivotal role in securing adequate oxygen delivery to the various organs and serves to ensure an appropriate convective transport of the nutrients taken up by the intestine. The tachycardia is mediated by a release of vagal tone and the presence of a non-adrenergic-non-cholinergic factor which stimulates the heart, which has been speculated to be released from the gastrointestinal organs during digestion [53, 54]. The increased heart rate, and the rise in the volume of blood pumped with each beat, must be supported by increased myocardial metabolism and we observed an upregulation of malate dehydrogenase, cytochromes and ATPase linked enzymes (Fig. 6) that are likely to be related to an increased oxidative phosphorylation within the individual myocytes (see also the prevalence of enriched GO terms associated with aerobic metabolism in Supplementary Fig. S8). Previous gene expression studies on the python heart also yielded evidence for its increased oxidative capacity in postprandial period [55] and cytochrome oxidase activity is almost doubled during digestion [56]. We confirm that transcription for heat shock proteins may be increased [55], possibly to protect against oxidative damage as result of the increased metabolism. As in earlier studies [55], our observation of increased ATP synthase lipid–binding protein and fatty acid binding protein 3 (Fig. 6) provide evidence for increased fatty acid metabolism, which may reflect the substantial rise in circulating fatty

acids in the plasma.

It was originally suggested that the postprandial rise in stroke volume could be ascribed to an impressive and swift growth of the heart [10], possibly triggered by lipid-signalling [55]. However, a number of recent studies, primarily from our laboratory, have shown that increased cardiac mass is not an obligatory postprandial response amongst pythons [56-58], and that stroke volume may be increased in response to increased venous return rather than cardiac hypertrophy [56]. It is nevertheless, noteworthy that our and the previous studies show a clear increase in the expression of contractile proteins (e.g. myosin and actin) as well as tubulin (Fig. 6), which may reflect increased protein-turnover in response to increased myocardial workload rather than cell proliferation or hypertrophy. The enriched GO analyses also point to major changes in the extracellular space as well as both elastin and collagen, which may indicate some level of cardiac reorganization at the cellular or subcellular level that may alter compliance of the myocardial wall and influence cardiac filling (Supplementary Fig. S8). It is noteworthy that the increased expression of BNP may serve a signalling function as described in response to the cardiac hypertrophy that attends hypertension.

Physiological interpretation of the genes in the liver

The liver exhibited a diverse expression profile in response to digestion that is likely to reflect its many metabolic functions in connection with metabolism, synthesis and detoxification during the postprandial period. This pattern is also evident from the many metabolic functions identified in the enriched GO analysis (Supplementary Fig. S7). There were marked upregulations of the P450 system (Fig. 7), which fits well with a rise in synthesis and breakdown of hormones and signalling molecules, cholesterol synthesis in response to lipid absorption and possibly also an increased metabolism of potentially toxic

compounds in the prey. A rise in cholesterol metabolism was supported by increased expression apolipoproteins (Fig. 7). The hepatic involvement in lipid metabolism was also supported by the increased expression of genes for Alpha-2-macroglobulin and serum albumin (Fig. 7). The increased expression of albumin obviously also corresponds nicely with the proteomic analysis of plasma proteins and it is likely that the postprandial rise in plasma albumin serves a functional role in the lipid transport between the intestine and the liver as well as other metabolically active organs.

It is also noteworthy that a number of genes associated with the protection of oxidative stress, such as catalase, heat shock protein and glutathionine transferase were markedly upregulated (Fig. 7). It was recently argued that snakes digesting large meals experience oxidative damage due to reactive oxygen metabolites requiring increased antioxidant responses to protect cellular functions [59].

Physiological interpretation of the genes in the pancreas

We sampled the entire pancreas for our analysis of gene expression and our data therefore reflect both endocrine and exocrine pancreatic functions. We found ample evidence for upregulated expression of genes associated with the digestive functions, such as lipases, trypsin, chymotrypsin and elastase and other enzymes for digestion of protein and lipid (Fig. 8). This general upregulation of secretory processes is likely to explain the prevalence of processes associated with protein synthesis in the enriched GO analysis (Supplementary Fig. S6). There was even an increased expression of amylase (Fig. 8) which breaks down polysaccharides. In connection with this latter function, the increased expression of insulin (Fig. 8) from the endocrine pancreas is likely to reflect increased cellular signalling for postprandial uptake of both glucose and amino acids. As in the other organs, we found increased expression of cytochrome oxidase (Fig. 8) indicative of increased metabolism

during digestion, and the rise in heat shock protein expression may reflect a response to formation of reactive oxygen-species as metabolism is stimulated by increased secretion of the pancreas.

Conclusions

Our study confirms that the extensive physiological and anatomical reorganization of the visceral organs of pythons during the postprandial period is driven by differential expression of hundreds or even thousands of genes. Many of the upregulated functions pertain to energy production to support the rise in aerobic metabolism associated with the digestion and absorption of large meals. In terms of the gastrointestinal organs, the gene expression profiles also support the view that many of the digestive functions, such as gastric acid secretion and nutrient absorption, can be stimulated with little change to gene expression, indicating that the proteins involved in these processes merely need to be activated during the postprandial period, and thus avoiding the energy and time-consuming processes associated with *de novo* synthesis. This digestive strategy may, at least in part, explain how intermittent feeders, such as snakes, retain the capacity for rapid and reliable upregulation of the digestive processes immediately after prey ingestion.

Methods

Stimulation of the postprandial response, collection of tissue biopsies and purification of RNA for mRNA-seq analyses

Six *Python bivittatus* (Tiger Python/Burmese Python) with a body mass ranging from 180 to 700 g (average 373 g) were obtained from a commercial supplier and housed in vivaria with a heating system providing temperatures of 25-32 °C. The animals were fed rodents once a week and fresh water was always available. The animals appeared healthy and all experiments were performed according to Danish Federal Regulations. All six individuals were fasted for one month and divided in three groups. Four animals were fed a rodent meal of 25 % of body weight and euthanized with an intra-peritoneal injection of pentobarbital (50 mg kg^{-1}; Mebumal) at 24h (N = 2) or 48h after feeding (N = 2). The remaining two snakes served as fasted controls. During deep anaesthesia, two biopsies were obtained from each snake from each of the following tissues: heart (ventricles), liver, stomach, intestine, and pancreas. In regard to the stomach tissue samples, one sample was obtained from the proximal part of the stomach and one sample was obtained from the distal part. In total, 60 biopsies were collected. The samples were taken from the same part of the different tissues in all individuals. After sampling, the biopsies were weighed and immediately snap frozen in liquid nitrogen; stomach and intestinal tissues were rinsed in sterile saline solution before weighting to avoid contamination with rodent tissue from the ingested meal. Subsequently, all 60 biopsies were homogenized in liquid nitrogen and the four biological replicates (two biopsies from each individual) were pooled in a 1:1 manner based on mass. This resulted in 15 samples (five tissues X three time points). From these samples, total RNA was purified using the Nucleospin RNA II kit (Machery-Nagel GmbH & Co.), as recommended by the manufacturer. The RNA concentration and quality were assessed by Nanodrop ND 1000

Spectrophotometer (Thermo Scientific) analyses, agarose gel-electrophoreses, and Agilent BioAnalyzer (Agilent) analyses.

Library production and sequencing

Poly-A transcripts were enriched and the transcripts broken in the presence of Zn^{2+}. Subsequently, double-stranded cDNA was synthesized using random primers and RNase H. After end repair and purification, the fragments were ligated with bar-coded paired-end adapters, and fragments with insert sizes of approximately 150-250 bp were isolated from an agarose gel. Each of the 15 samples derived from five tissues (heart, liver, stomach, pancreas and intestine) at the three time points (fasted for one month, 24h and 48h post-feeding) were amplified by PCR to generate DNA colonies template libraries and the libraries were then purified. In addition, to sample as broadly from each transcriptome as possible, we also produced normalized libraries for each tissue in order to capture the reads from lowly expressed, tissue-specific genes. Here, a part of the samples, which originating from the same tissue, were pooled before the PCR analyses, i.e. in total five pooled samples were generated. These five samples were split in two and after PCR amplification and library purification they were normalized using two different normalization protocols, i.e. in total 10 normalized libraries were prepared. Library quality of all 25 samples was then assessed by a titration-run (1 x 50 bp) on an Illumina HiSeq 2000 instrument. Finally, the sequencing was performed on the same instrument using paired-reads (2 × 101 bp). One channel was used for the 15 non-normalized libraries and one channel was used for the 10 normalized libraries.

Data pre-processing and *de novo* transcriptome assembly

To reduce the amount of erroneous data, the raw paired reads were processed by i) removing reads that contained the sequencing adaptor, ii) removing reads that contained ambiguous

characters (Ns), and iii) trimming bases that had the low average quality (Q<20) within a sliding window of length 10.

To develop a comprehensive transcriptomics resource for the Burmese python, all high-quality reads from 25 libraries were pooled together for *de novo* assembly. To determine the optimal assembly, *de novo* assembly was performed using Velvet（version 1.2.03)(Velvet, RRID:SCR_010755) [18] and Oases (Oases, RRID:SCR_011896) (version 0.2.06) [60] with different k-mer parameters. The performance of these assemblies was assessed according to number of transcripts, total length of transcripts, N50 length, mean length, proportion of mapped reads and number of transcripts which length is larger than N50 (Supplementary Table S2).

Assessment of the transcriptome assembly

The transcriptome assembly was evaluated by rnaQUAST 1.4.0 with default parameters supplying reference genome sequences and genome annotation of Burmese python (GenBank assembly accession: GCA_000186305.2).

BUSCO_v2 (BUSCO , RRID:SCR_015008) [20] was used to test the completeness of transcriptome assembly with dependencies NCBI BLAST+ 2.4.0 [61] and HMMER 3.1b2 (Hmmer, RRID:SCR_005305)[62]. The vertebrata lineage set was used and accessed on 28 Nov 2016.

Transcriptome annotation

To assess the identity of the most closely related gene in other organisms, the assembled transcripts were compared with the sequences in the National Center for Biotechnology Information (NCBI) non-redundant protein (nr) database using blastx (BLASTX , RRID:SCR_001653) [63] with an e-value cut-off of 0.01. The nr annotation term of each

transcript was assigned with the first best hit, which was not represented in uninformative description (e.g., 'hypothetical protein', 'novel protein', 'unnamed protein product', 'predicted protein' or 'Uncharacterized protein') (Supplementary Table S4). To assign functional annotations of transcripts, Blast2GO was used (e-value threshold = 0.01) to return GO annotation, Enzyme code annotation with KEGG maps and InterPro annotation.

Estimation of gene expression values

For each 15 non-normalized libraries, the paired-end reads were firstly mapped back to assembled transcriptome using Bowtie2 (Bowtie , RRID:SCR_005476) [64]with default parameters, the raw counts then were calculated based on the alignment results using RSEM (version 1.1.20) [65] for each transcript. To quantify the gene expression level, for genes with alternative splicing transcripts, the longest transcript was selected to represent the gene, and a gene’s abundance estimate was the sum of its transcripts’ abundance estimates. Finally, the raw expression counts were normalized into FPKM with custom Perl scripts.

PCA

To facilitate graphical interpretation of tissue relatedness, R function prcomp was used to perform PCA with genes which the maximum FPKM of 15 samples was greater than 100.

Identification of DEGs and clustering analysis

For each tissue, DEGs were selected with two thresholds, 1) FPKM is greater than or equal to 400 in at least one time point and 2) FC is greater than or equal to two in at least one pairwise comparison among three time points. FPKM values of DEGs were log2-transformed and median-centered, then hierarchical clustering was performed using R command hclust with method = ‘average’ and distance = ‘Spearman correlation’ and results were displayed using R command heatmap.2.

Coloured KEGG Pathway and GO enrichment analysis

For each tissue, all assembled genes were mapped to KEGG human pathway maps using KOBAS 2.0 [66] with e-value 1e-50. Then genes were coloured by representing FPKM value and trend of differential expression value (Table 2).

Blast2GO was used to implement GO enrichment analysis (Fisher's exact test) with threshold of FDR 0.001. The reference set is the whole transcripts with GO slim annotation. For each organ, the selected test set is either upregulated or highly expressed genes defined in Table 2. Finally, we performed Blast2GO to reduce to most specific GO terms.

Isolation of samples for proteomics analyses

Two Burmese pythons (weighing 400 and 800 g, respectively) were fed a rodent meal corresponding to approximately 25% of their body mass. Approximately 24 h into the postprandial period the animals were euthanized with an overdose of pentobarbital (100 mg kg^{-1}, i.m.). Immediately afterwards, an incision was made to expose the stomach, which was then ligated at the lower oesophagus and the pylorus, before the intact stomach was excised by a cleavage just below the two sutures resulting in the stomach being released from the rest of the animal. All undigested mouse remains were manually removed by forceps and 25 ml/kg tris-buffered saline (TBS) was injected into the stomach. The stomach was then ligated at the opened end, rinsed by gently shaking the tissue, and finally the digestive fluid-containing solution was collected and stored on ice. To ensure collection of all gastric fluid, the stomach was rinsed additional two-three times with 12 ml/kg TBS. Subsequently, the samples were filtered and centrifuged, and the supernatant stored at -80 °C. We also obtained two samples of gastric juice from a third individual (200 g) that had been fed 4 g peptone

(Sigma Aldrich), suspended in water. Peptone is a mixture of small peptides and amino acids and the solution was injected directly into the stomach and after three hours the snake was euthanized by an overdose of pentobarbital. The stomach was removed, rinsed with TBS, and a single sample collected and stored, as described above. We analysed two samples from each of the three individuals, resulting in a total of six digestive fluid samples being analysed by MS/MS. In addition, we obtained a single plasma sample from each snake by direct cardiac puncture followed by centrifugation and storage for later analysis.

Sample preparation for mass spectrometry analyses

The proteins in the six obtained python digestive fluid samples were recovered by trichloroacetic acid precipitation. The resulting pellets were resuspended in 8 M Urea, 5 mM DTT, 0.1 M ammonium bicarbonate pH 8.0 and incubated for 30 minutes at room temperature in order to denature and reduce the proteins. Subsequently, the proteins were alkylated by the addition of iodoacetamide to a final concentration of 25 mM. The samples were incubated for additional 20 minutes at room temperature and then diluted five times with a 50 mM ammonium bicarbonate, pH 8.0 buffer before the addition of approximately 2 μg sequencing grade modified trypsin (Promega) per 50 μg protein in the sample. Subsequently, the samples were incubated at 37 °C for approximately 16 h. The proteins in the plasma sample were denatured, reduced, alkylated, and digested with trypsin, as described for the digestive fluid samples. Finally, the resulting peptides in all samples were micropurified and stored at -20 C until the LC-MS/MS analyses.

Liquid chromatography-tandem mass spectrometry analyses

Nano-liquid chromatography-tandem mass spectrometry (LC–MS/MS) analyses were performed on a nanoflow HPLC system (Thermo Scientific, EASY-nLC II) connected to a

mass spectrometer (TripleTOF 5600, AB Sciex) equipped with an electrospray ionization source (NanoSpray III, AB Sciex) and operated under Analyst TF 1.6 control. The samples were dissolved in 0.1% formic acid, injected, trapped and desalted isocratically on a precolumn whereupon the peptides were eluted and separated on an analytical column (16 cm × 75 μm i.d.) packed in-house with ReproSil-Pur C18-AQ 3 μm resin (Dr. Marisch GmbH). The peptides were eluted at a flow rate of 250 nL/min using a 50 min gradient from 5 % to 35 % phase B (0.1 % formic acid and 90 % acetonitrile). An information dependent acquisition method was employed allowing up to 25 MS/MS spectra per cycle of 2.8 s.

Protein identification and filtering of data

The six collected MS files, related to digested fluid, were converted to Mascot generic format (MGF) using the AB SCIEX MS Data Converter beta 1.3 (AB SCIEX) and the “proteinpilot MGF” parameters. Subsequently, the files were merged to a single MGF-file using Mascot daemon. The resulting file (encompassing 122538 MS/MS queries) was used to interrogate the 16693 *Mus musculus* sequences in the Swiss-Prot database (version 2014_10) and the generated python database encompassing 21131 protein sequences using Mascot 2.5.0 (Matrix Science)[67]. Trypsin, with up to one missed cleavage allowed, was selected as enzyme; carbamidomethyl was employed as fixed modification, and oxidation of methionine and proline was selected as variable modifications. The instrument setting was specified as ESI-QUAD-TOF, the mass accuracy of the precursor and product ions was 15 ppm and 0.2 da respectively, and the significance threshold (p) was set to 0.01 and an expect cut-off at 0.005. The data obtained by the LC-MS/MS-analysis of the python plasma proteome was analysed as described for the digestive fluid samples, except that the *Mus musculus* sequences were not interrogated. This dataset contains 9224 MS/MS queries. All obtained results were subsequently parsed using MS Data Miner v. 1.3.0 [68], and protein hits were only accepted

if they were identified based on two unique peptides. Semi-quantitative proteomics data was obtained using the emPAI-values given by the Mascot 2.5.0 software after analysis of the MS/MS data [69].

To identify the proteins secreted into the python stomach, identified python plasma proteins, as well as the mouse protein homologs were removed from the list of identified python digestive fluid proteins. With regard to the removal of prey protein homologs, the overall mouse protein names were used to search the list of python proteins (e.g. “collagen” was used as search term, not “collagen alpha-1(I) chain”) and to identify python proteins that were identified based on homology with mouse. These proteins were removed from the list of stomach-secreted python proteins. For each identified protein remaining on the list, we reassessed the annotation of the python sequence, i.e. sequence comparisons were performed using blastp version 2.2.30, and in addition, UniProt and NCBI protein databases, as well as PubMed and SignalP 4.1, were interrogated to identify functional properties and cellular location of the identified proteins. Plasma proteins, remaining collagen homologous, intracellular proteins, and membrane proteins were discarded from the list of identified python stomach secretome proteins.

The mass spectrometry proteomics data have been deposited to the ProteomeXchange Consortium via the PRIDE [70] partner repository with the dataset identifier PXD006665.

List of abbreviations

DEG	differentially expressed genes
FC	fold change
FPKM	fragments per kilo base per million sequenced reads
PCA	principal component analysis

Declarations

Availability of data and materials

The raw RNA-Seq sequencing data that support the findings of this study have been deposited in the NCBI BioProject database (accession no. PRJNA343735). https://www.ncbi.nlm.nih.gov/bioproject/PRJNA343735

The mass spectrometry proteomics data have been deposited to the ProteomeXchange Consortium via the PRIDE [70] partner repository with the dataset identifier PXD006665.

Supporting data is also available from the *GigaScience* GigaDB repository[71].

Competing interests

The authors declare that they have no competing interests.

Funding

This work was supported by a grant (Novenia) from the Danish Research Council for Strategic Research (grant identification number: 09-067076).

Authors' contributions

JD, KWS, TW and MHS designed the study. JD performed the transcriptome data analysis with input from LS and was a major contributor in writing the manuscript. SEL performed RNA-Seq lab experiment. KWS and JE performed the proteomics experiment and data

analysis. WT interpreted the transcriptome data regarding digestion. All authors read and approved the final manuscript.

Acknowledgements

We thank Tania A. Nielsen (Aarhus, Denmark) for valuable assistance with RNA purification and Fasteris SA (Switzerland) for library preparation and Illumina sequencing.

Figure and table legends

Fig. 1. The workflow of Python RNA-Seq data analysis. The diagram shows the main steps and bioinformatics tools used in the study.

Fig. 2. PCA plots of FPKM of 1862 genes. PC, principal component. PC1 represents 25%, PC2 represents 18% and PC3 represents 16% of total variation in the data. The name of the label consists of two parts: one capital letter plus one number. Letter H, S, I, L, P represent heart, stomach, intestine, liver and pancreas respectively. Numbers 0, 1, 2 represent fasting for one month, 24h/1d after feeding and 48h/2d after feeding respectively.

Fig. 3. Heat maps from hierarchical clustering of DEGs in each tissue. Heat maps showing the hierarchically clustered Spearman correlation matrix resulting from comparing the normalized FPKM value for each pair of genes. Heat map columns represent samples and rows correspond to genes. Expression values (FPKM) are log_2-transformed and then median-centered by gene. Relative levels of gene expression are represented by colours. Pale colour is low expression and darker blue is high expression. Five sub-clusters labelled a to e are shown with full annotation in Fig. 4-8.

Fig. 4. The cluster of upregulated genes with NCBI nr annotation in stomach. Figure represents the cluster e in Fig. 3. Heat map columns represent samples and rows correspond to genes. Expression values (FPKM) are log_2-transformed and then median-centered by gene. Relative levels of gene expression are represented by colours. Pale colour is low expression and darker blue is high expression.

Fig. 5. The cluster of upregulated genes with NCBI nr annotation in intestine. Figure represents the cluster b in Fig. 3. Heat map columns represent samples and rows correspond to genes. Expression values (FPKM) are log_2-transformed and then median-centered by gene. Relative levels of gene expression are represented by colours. Pale colour is low expression and darker blue is high expression.

Fig. 6. The cluster of upregulated genes with NCBI nr annotation in heart. Figure represents the cluster a in Fig. 3. Heat map columns represent samples and rows

correspond to genes. Expression values (FPKM) are log_2-transformed and then median-centered by gene. Relative levels of gene expression are represented by colours. Pale colour is low expression and darker blue is high expression.

Fig. 7. The cluster of upregulated genes with NCBI nr annotation in liver. Figure represents the cluster c in Fig. 3. Heat map columns represent samples and rows correspond to genes. Expression values (FPKM) are log_2-transformed and then median-centered by gene. Relative levels of gene expression are represented by colours. Pale colour is low expression and darker blue is high expression.

Fig. 8. The cluster of upregulated genes with NCBI nr annotation in pancreas. Figure represents the cluster d in Fig. 3. Heat map columns represent samples and rows correspond to genes. Expression values (FPKM) are log_2-transformed and then median-centered by gene. Relative levels of gene expression are represented by colours. Pale colour is low expression and darker blue is high expression.

Fig. 9. The workflow used to identify the python's stomach secretome during digestion. 1) Initially pythons were fed with mice, or a peptide mixture, and later the gastric juice samples were obtained and mice debris was removed. **2)** The proteins were precipitated, denatured and digested with trypsin. **3)** The resulting tryptic peptides were analysed by LC-MS/MS analyses and the data merged into a single file. **4)** The file was used to interrogate the in-house generated python protein sequence database (based on the transcriptomic data) and python proteins were identified. **5)** The data was filtered to remove mice proteins and plasma proteins. Subsequently, the annotation of the remaining proteins was reassessed and the secretome identified.

Fig. 10. Cartoon depiction of coloured KEGG pathway of gastric acid secretion in stomach. Entry in red represents upregulated during digestion; Entry in purple for highly expressed. H/K is H+/K+-exchanging ATPase alpha polypeptide. CA is carbonic anhydrase. AE is solute carrier family 26 (anion exchange transporter).

Table 1. Summary of transcriptome assembly of Burmese Python.

Table 2. Colour coding of genes in KEGG pathway maps. Three criteria are used to classify and colour genes. First, i) whether the maximum FPKM of the gene among fasting, 24h and 48h is over 10, then ii) whether the gene is differential expressed in at least one of the pairwise comparison among fasting, 24h and 48h with FC over 4. Finally, iii) for those genes expressed, but not differential expressed, whether it is highly expressed with maximum FPKM among three time points over 200. The term expression trend indicates the trend of gene expression across fasting, 24h and 48h. e.g. The trend up means the gene is upregulated from either fasting to 24h, fasting to 48h or 24h to 48h. The trend up-then-down means the gene is firstly upregulated from fasting to 24h, then downregulated from 24h to 48h.

Table 3. The number of DEGs across fasting, 24h and 48h in each tissue. The expression trend is consistent with definition in Table 2.

Reference

1. Secor SM, Diamond J: **A vertebrate model of extreme physiological regulation.** *Nature* 1998, **395:**659-662.
2. Cox CL, Secor SM: **Matched regulation of gastrointestinal performance in the Burmese python, Python molurus.** *J Exp Biol* 2008, **211:**1131-1140.
3. Secor SM, Diamond J: **Adaptive responses to feeding in Burmese pythons: pay before pumping.** *J Exp Biol* 1995, **198:**1313-1325.
4. Secor SM, Diamond JM: **Evolution of regulatory responses to feeding in snakes.** *Physiological and Biochemical Zoology* 2000, **73:**123-141.
5. Aubret F, Shine R, Bonnet X: **Evolutionary biology: adaptive developmental plasticity in snakes.** *Nature* 2004, **431:**261-262.
6. Cohn MJ, Tickle C: **Developmental basis of limblessness and axial patterning in snakes.** *Nature* 1999, **399:**474-479.
7. Ott BD, Secor SM: **Adaptive regulation of digestive performance in the genus Python.** *J Exp Biol* 2007, **210:**340-356.
8. Secor SM: **Digestive physiology of the Burmese python: broad regulation of integrated performance.** *J Exp Biol* 2008, **211:**3767-3774.
9. Zaar M, Overgaard J, Gesser H, Wang T: **Contractile properties of the functionally divided python heart: two sides of the same matter.** *Comp Biochem Physiol A Mol Integr Physiol* 2007, **146:**163-173.
10. Andersen JB, Rourke BC, Caiozzo VJ, Bennett AF, Hicks JW: **Physiology: postprandial cardiac hypertrophy in pythons.** *Nature* 2005, **434:**37-38.
11. Vidal N, Hedges SB: **Molecular evidence for a terrestrial origin of snakes.** *Proc Biol Sci* 2004, **271 Suppl 4:**S226-229.
12. Castoe TA, de Koning AP, Hall KT, Card DC, Schield DR, Fujita MK, Ruggiero RP, Degner JF, Daza JM, Gu W, et al: **The Burmese python genome reveals the molecular basis for extreme adaptation in snakes.** *Proc Natl Acad Sci U S A* 2013, **110:**20645-20650.
13. Andrew AL, Card DC, Ruggiero RP, Schield DR, Adams RH, Pollock DD, Secor SM, Castoe TA: **Rapid changes in gene expression direct rapid shifts in intestinal form and function in the Burmese python after feeding.** *Physiol Genomics* 2015, **47:**147-157.
14. Castoe TA, Fox SE, Jason de Koning A, Poole AW, Daza JM, Smith EN, Mockler TC, Secor SM, Pollock DD: **A multi-organ transcriptome resource for the Burmese Python (Python molurus bivittatus).** *BMC Res Notes* 2011, **4:**310.
15. Wang Z, Gerstein M, Snyder M: **RNA-Seq: a revolutionary tool for transcriptomics.** *Nat Rev Genet* 2009, **10:**57-63.
16. Mortazavi A, Williams BA, McCue K, Schaeffer L, Wold B: **Mapping and quantifying mammalian transcriptomes by RNA-Seq.** *Nat Methods* 2008, **5:**621-628.
17. Wang T, Hung CC, Randall DJ: **The comparative physiology of food deprivation: from feast to famine.** *Annu Rev Physiol* 2006, **68:**223-251.
18. Zerbino DR, Birney E: **Velvet: algorithms for de novo short read assembly using de Bruijn graphs.** *Genome Res* 2008, **18:**821-829.

19. Bushmanova E, Antipov D, Lapidus A, Suvorov V, Prjibelski AD: **rnaQUAST: a quality assessment tool for de novo transcriptome assemblies.** *Bioinformatics* 2016, **32:**2210-2212.

20. Simão FA, Waterhouse RM, Ioannidis P, Kriventseva EV, Zdobnov EM: **BUSCO: assessing genome assembly and annotation completeness with single-copy orthologs.** *Bioinformatics* 2015:btv351.

21. Pruitt KD, Tatusova T, Maglott DR: **NCBI reference sequences (RefSeq): a curated non-redundant sequence database of genomes, transcripts and proteins.** *Nucleic Acids Res* 2007, **35:**D61-65.

22. Pyron RA: **Novel Approaches for Phylogenetic Inference from Morphological Data and Total-Evidence Dating in Squamate Reptiles (Lizards, Snakes, and Amphisbaenians).** *Syst Biol* 2017, **66:**38-56.

23. Conesa A, Gotz S, Garcia-Gomez JM, Terol J, Talon M, Robles M: **Blast2GO: a universal tool for annotation, visualization and analysis in functional genomics research.** *Bioinformatics* 2005, **21:**3674-3676.

24. Hunter S, Apweiler R, Attwood TK, Bairoch A, Bateman A, Binns D, Bork P, Das U, Daugherty L, Duquenne L, et al: **InterPro: the integrative protein signature database.** *Nucleic Acids Res* 2009, **37:**D211-215.

25. Trapnell C, Williams BA, Pertea G, Mortazavi A, Kwan G, van Baren MJ, Salzberg SL, Wold BJ, Pachter L: **Transcript assembly and quantification by RNA-Seq reveals unannotated transcripts and isoform switching during cell differentiation.** *Nat Biotechnol* 2010, **28:**511-515.

26. Fan HP, Di Liao C, Fu BY, Lam LC, Tang NL: **Interindividual and interethnic variation in genomewide gene expression: insights into the biological variation of gene expression and clinical implications.** *Clin Chem* 2009, **55:**774-785.

27. Kanehisa M, Goto S, Sato Y, Furumichi M, Tanabe M: **KEGG for integration and interpretation of large-scale molecular data sets.** *Nucleic Acids Res* 2012, **40:**D109-114.

28. Kanehisa M, Goto S: **KEGG: kyoto encyclopedia of genes and genomes.** *Nucleic Acids Res* 2000, **28:**27-30.

29. Kageyama T: **Pepsinogens, progastricsins, and prochymosins: structure, function, evolution, and development.** *Cell Mol Life Sci* 2002, **59:**288-306.

30. Loytynoja A, Goldman N: **webPRANK: a phylogeny-aware multiple sequence aligner with interactive alignment browser.** *BMC Bioinformatics* 2010, **11:**579.

31. Kumar S, Stecher G, Tamura K: **MEGA7: Molecular Evolutionary Genetics Analysis Version 7.0 for Bigger Datasets.** *Mol Biol Evol* 2016, **33:**1870-1874.

32. Hayashi K, Agata K, Mochii M, Yasugi S, Eguchi G, Mizuno T: **Molecular cloning and the nucleotide sequence of cDNA for embryonic chicken pepsinogen: phylogenetic relationship with prochymosin.** *J Biochem* 1988, **103:**290-296.

33. Watanuki K, Yasugi S: **Analysis of transcription regulatory regions of embryonic chicken pepsinogen (ECPg) gene.** *Dev Dyn* 2003, **228:**51-58.

34. Aoki N, Deshimaru M, Terada S: **Active fragments of the antihemorrhagic protein HSF from serum of habu (Trimeresurus flavoviridis).** *Toxicon* 2007, **49:**653-662.

35. Perales J, Neves-Ferreira AG, Valente RH, Domont GB: **Natural inhibitors of snake venom hemorrhagic metalloproteinases.** *Toxicon* 2005, **45:**1013-1020.

36. Tomihara Y, Yonaha K, Nozaki M, Yamakawa M, Kawamura Y, Kamura T, Toyama S: **Purification of an antihemorrhagic factor from the serum of the non-venomous snake Dinodon semicarinatus.** *Toxicon* 1988, **26:**420-423.
37. Lee HJ, Sorimachi H, Jeong SY, Ishiura S, Suzuki K: **Molecular cloning and characterization of a novel tissue-specific calpain predominantly expressed in the digestive tract.** *Biol Chem* 1998, **379:**175-183.
38. Secor SM: **Gastric function and its contribution to the postprandial metabolic response of the Burmese python Python molurus.** *Journal of Experimental Biology* 2003, **206:**1621-1630.
39. Bessler SM, Secor SM: **Effects of feeding on luminal pH and morphology of the gastroesophageal junction of snakes.** *Zoology (Jena)* 2012, **115:**319-329.
40. Helmstetter C, Pope RK, T'Flachebba M, Secor SM, Lignot J-H: **The effects of feeding on cell morphology and proliferation of the gastrointestinal tract of juvenile Burmese pythons (Python molurus).** *Canadian Journal of Zoology* 2009, **87:**1255-1267.
41. Enok S, Simonsen LS, Wang T: **The contribution of gastric digestion and ingestion of amino acids on the postprandial rise in oxygen consumption, heart rate and growth of visceral organs in pythons.** *Comp Biochem Physiol A Mol Integr Physiol* 2013, **165:**46-53.
42. Nørgaard S, Andreassen K, Malte CL, Enok S, Wang T: **Low cost of gastric acid secretion during digestion in ball pythons.** *Comparative Biochemistry and Physiology Part A: Molecular & Integrative Physiology* 2016, **194:**62-66.
43. Secor SM, Taylor JR, Grosell M: **Selected regulation of gastrointestinal acid-base secretion and tissue metabolism for the diamondback water snake and Burmese python.** *J Exp Biol* 2012, **215:**185-196.
44. Andrade DV, De Toledo LF, Abe AS, Wang T: **Ventilatory compensation of the alkaline tide during digestion in the snake Boa constrictor.** *J Exp Biol* 2004, **207:**1379-1385.
45. Koelz HR: **Gastric acid in vertebrates.** *Scand J Gastroenterol Suppl* 1992, **193:**2-6.
46. Starck JM, Beese K: **Structural flexibility of the intestine of Burmese python in response to feeding.** *Journal of Experimental Biology* 2001, **204:**325-335.
47. Holmberg A, Kaim J, Persson A, Jensen J, Wang T, Holmgren S: **Effects of digestive status on the reptilian gut.** *Comparative Biochemistry and Physiology a-Molecular and Integrative Physiology* 2002, **133:**499-518.
48. Lignot JH, Helmstetter C, Secor SM: **Postprandial morphological response of the intestinal epithelium of the Burmese python (Python molurus).** *Comp Biochem Physiol A Mol Integr Physiol* 2005, **141:**280-291.
49. Secor SM, Whang EE, Lane JS, Ashley SW, Diamond J: **Luminal and systemic signals trigger intestinal adaptation in the juvenile python.** *American Journal of Physiology-Gastrointestinal and Liver Physiology* 2000, **279:**G1177-G1187.
50. Overgaard J, Andersen JB, Wang T: **The effects of fasting duration on the metabolic response to feeding in Python molurus: an evaluation of the energetic costs associated with gastrointestinal growth and upregulation.** *Physiol Biochem Zool* 2002, **75:**360-368.
51. Secor SM, White SE: **Prioritizing blood flow: cardiovascular performance in response to the competing demands of locomotion and digestion for the Burmese python, Python molurus.** *J Exp Biol* 2010, **213:**78-88.

52. Secor SM, Hicks JW, Bennett AF: **Ventilatory and cardiovascular responses of a python (Python molurus) to exercise and digestion.** *J Exp Biol* 2000, **203:**2447-2454.

53. Skovgaard N, Møller K, Gesser H, Wang T: **Histamine induces postprandial tachycardia through a direct effect on cardiac H2-receptors in pythons.** *American Journal of Physiology - Regulatory, Integrative and Comparative Physiology* 2009, **296:**R774-R785.

54. Enok S, Simonsen LS, Pedersen SV, Wang T, Skovgaard N: **Humoral regulation of heart rate during digestion in pythons (Python molurus and Python regius).** *Am J Physiol Regul Integr Comp Physiol* 2012, **302:**R1176-1183.

55. Riquelme CA, Magida JA, Harrison BC, Wall CE, Marr TG, Secor SM, Leinwand LA: **Fatty acids identified in the Burmese python promote beneficial cardiac growth.** *Science* 2011, **334:**528-531.

56. Enok S, Leite G, Leite C, Gesser H, Hedrick MS, Wang T: **Improved cardiac filling facilitates the postprandial elevation of stroke volume in Python regius.** *J Exp Biol* 2016.

57. Slay CE, Enok S, Hicks JW, Wang T: **Reduction of blood oxygen levels enhances postprandial cardiac hypertrophy in Burmese python (Python bivittatus).** *J Exp Biol* 2014, **217:**1784-1789.

58. Jensen B, Larsen CK, Nielsen JM, Simonsen LS, Wang T: **Change of cardiac function, but not form, in postprandial pythons.** *Comp Biochem Physiol A Mol Integr Physiol* 2011, **160:**35-42.

59. Butler MW, Lutz TJ, Fokidis HB, Stahlschmidt ZR: **Eating increases oxidative damage in a reptile.** *J Exp Biol* 2016, **219:**1969-1973.

60. Schulz MH, Zerbino DR, Vingron M, Birney E: **Oases: robust de novo RNA-seq assembly across the dynamic range of expression levels.** *Bioinformatics* 2012, **28:**1086-1092.

61. Camacho C, Coulouris G, Avagyan V, Ma N, Papadopoulos J, Bealer K, Madden TL: **BLAST+: architecture and applications.** *BMC Bioinformatics* 2009, **10:**421.

62. Eddy SR: **A probabilistic model of local sequence alignment that simplifies statistical significance estimation.** *PLoS Comput Biol* 2008, **4:**e1000069.

63. Altschul SF, Gish W, Miller W, Myers EW, Lipman DJ: **Basic local alignment search tool.** *J Mol Biol* 1990, **215:**403-410.

64. Langmead B, Salzberg SL: **Fast gapped-read alignment with Bowtie 2.** *Nat Methods* 2012, **9:**357-359.

65. Li B, Dewey CN: **RSEM: accurate transcript quantification from RNA-Seq data with or without a reference genome.** *BMC Bioinformatics* 2011, **12:**323.

66. Xie C, Mao X, Huang J, Ding Y, Wu J, Dong S, Kong L, Gao G, Li CY, Wei L: **KOBAS 2.0: a web server for annotation and identification of enriched pathways and diseases.** *Nucleic Acids Res* 2011, **39:**W316-322.

67. Perkins DN, Pappin DJC, Creasy DM, Cottrell JS: **Probability-based protein identification by searching sequence databases using mass spectrometry data.** *Electrophoresis* 1999, **20:**3551-3567.

68. Dyrlund TF, Poulsen ET, Scavenius C, Sanggaard KW, Enghild JJ: **MS Data Miner: A web-based software tool to analyze, compare, and share mass spectrometry protein identifications.** *Proteomics* 2012, **12:**2792-2796.

69. Ishihama Y, Oda Y, Tabata T, Sato T, Nagasu T, Rappsilber J, Mann M: **Exponentially modified protein abundance index (emPAI) for estimation of**

absolute protein amount in proteomics by the number of sequenced peptides per protein. *Mol Cell Proteomics* 2005, **4:**1265-1272.

70. Vizcaino JA, Csordas A, del-Toro N, Dianes JA, Griss J, Lavidas I, Mayer G, Perez-Riverol Y, Reisinger F, Ternent T, et al: **2016 update of the PRIDE database and its related tools.** *Nucleic Acids Res* 2016, **44:**D447-456.

71. Duan, J; Sanggaard, K, W; Schauser, L; Lauridsen, S, E; Enghild, J, J; Schierup, M, H; Wang, T (2017): **Supporting data for "Transcriptome Analysis of the Response of Burmese Python to Digestion"** GigaScience Database. http://dx.doi.org/10.5524/100320

Table 1

Click here to download Table Table1.xlsx

**Parameter**	***De novo* assembly**
Total transcripts	34,423
Annotated transcripts with nr NCBI	19,713
Annotated transcripts with GO term	16,992
Minimum transcript size (nt)	100
Medium transcrpt size (nt)	605
Mean transcript size (nt)	1,034
Largest transcript (nt)	26,010
N50	6,240
N50 size (nt)	1,673
Total assembled bases (Mb)	35.6

Table 2

Click here to download Table Table2.xlsx

Expression level	Fold change level	Expression trend (fasting -> 24h -> 48h)	Color code
max FPKM over 10	FC over 4	Up-regulated	Red
		Down-regulated	Blue
		Up-then-down regulated	Yellow
		Down-then-up regulated	Brown
	FC below 4	Highly expressed (max FPKM over 200)	Purple
		Moderately expressed (max FPKM below 200)	Pink
max FPKM below 10	-	Lowly expressed	Darkgrey

Table 3

Click here to download Table Table3.xlsx

Expression trend (fasting -> 24h -> 48h)	Stomach	Intestine	Pancreas	Liver	Heart
Up-regulated	932 (2.9%)	1,131 (3.5%)	859 (2.6%)	1,047 (3.2%)	184 (0.6%)
Up-then-down regulated	28 (0.1%)	31 (0.1%)	150 (0.5%)	61 (0.2%)	6 (0.0%)
Down-regulated	869 (2.7%)	625 (1.9%)	567 (1.7%)	618 (1.9%)	168 (0.5%)
Down-then-up regulated	36 (0.1%)	45 (0.1%)	127 (0.4%)	90 (0.3%)	16 (0.1%)
Highly expressed	199 (0.6%)	211 (0.7%)	225 (0.7%)	354 (1.1%)	232 (0.7%)
Moderately expressed	5,541 (17.0%)	5,582 (17.2%)	4,933 (15.2%)	5,385 (16.5%)	6,044 (18.6%)
Lowly expressed	24,926 (76.6%)	24,906 (76.5%)	25,670 (78.9%)	24,976 (76.8%)	25,881 (79.5%)
**Total**	32,531 (100%)	32,531 (100%)	32,531 (100%)	32,531 (100%)	32,531 (100%)

Click here to download Figure Figure1.pdf

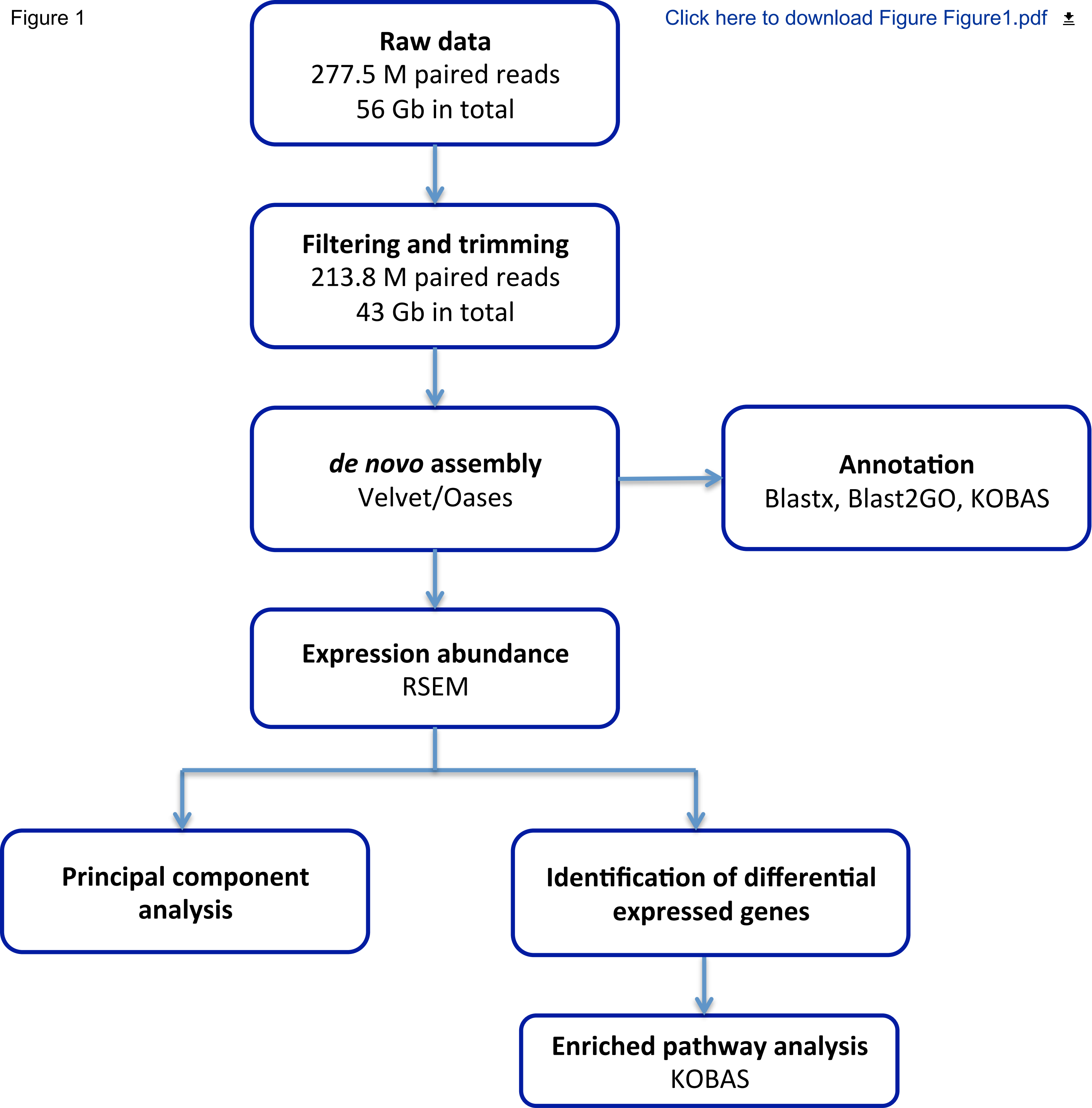

Figure 2

Click here to download Figure Figure2.pdf

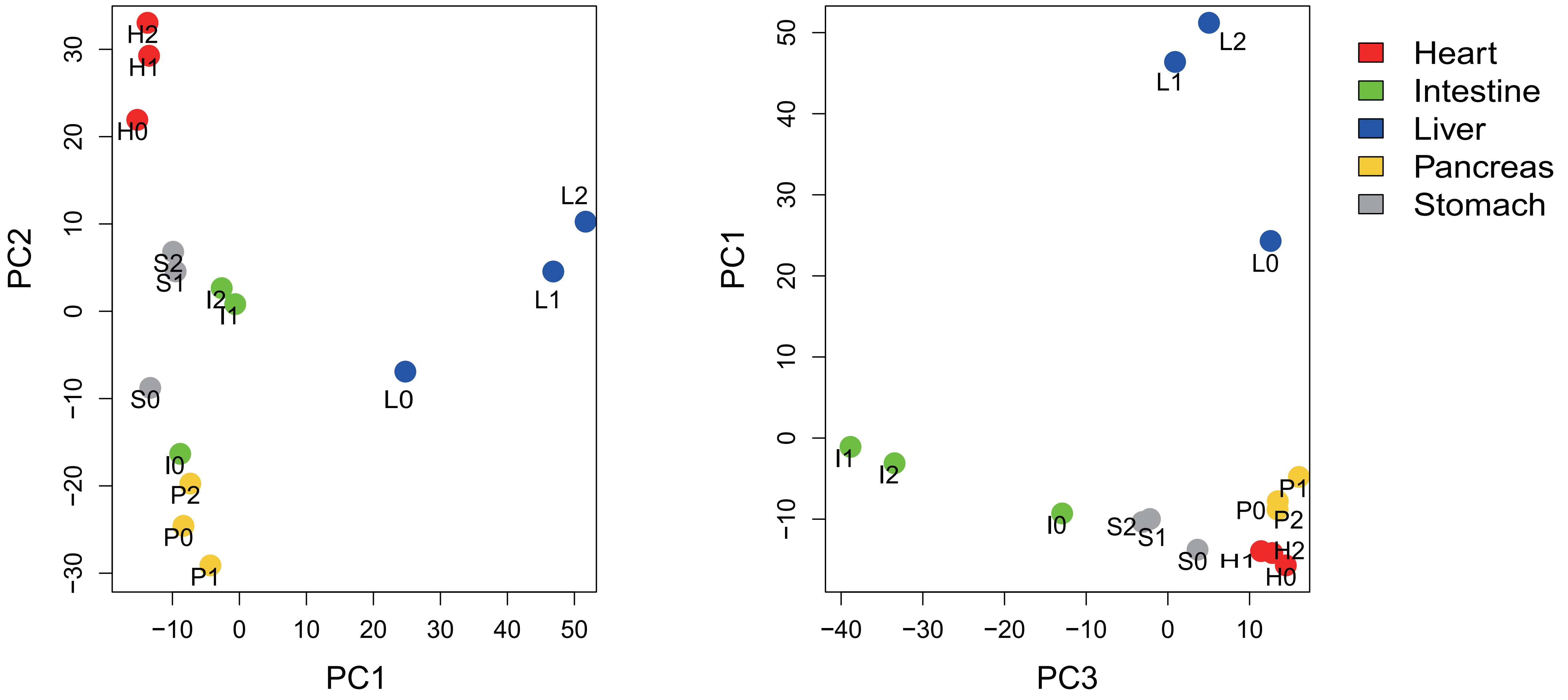

Figure 3 Click here to download Figure Figure3.pdf

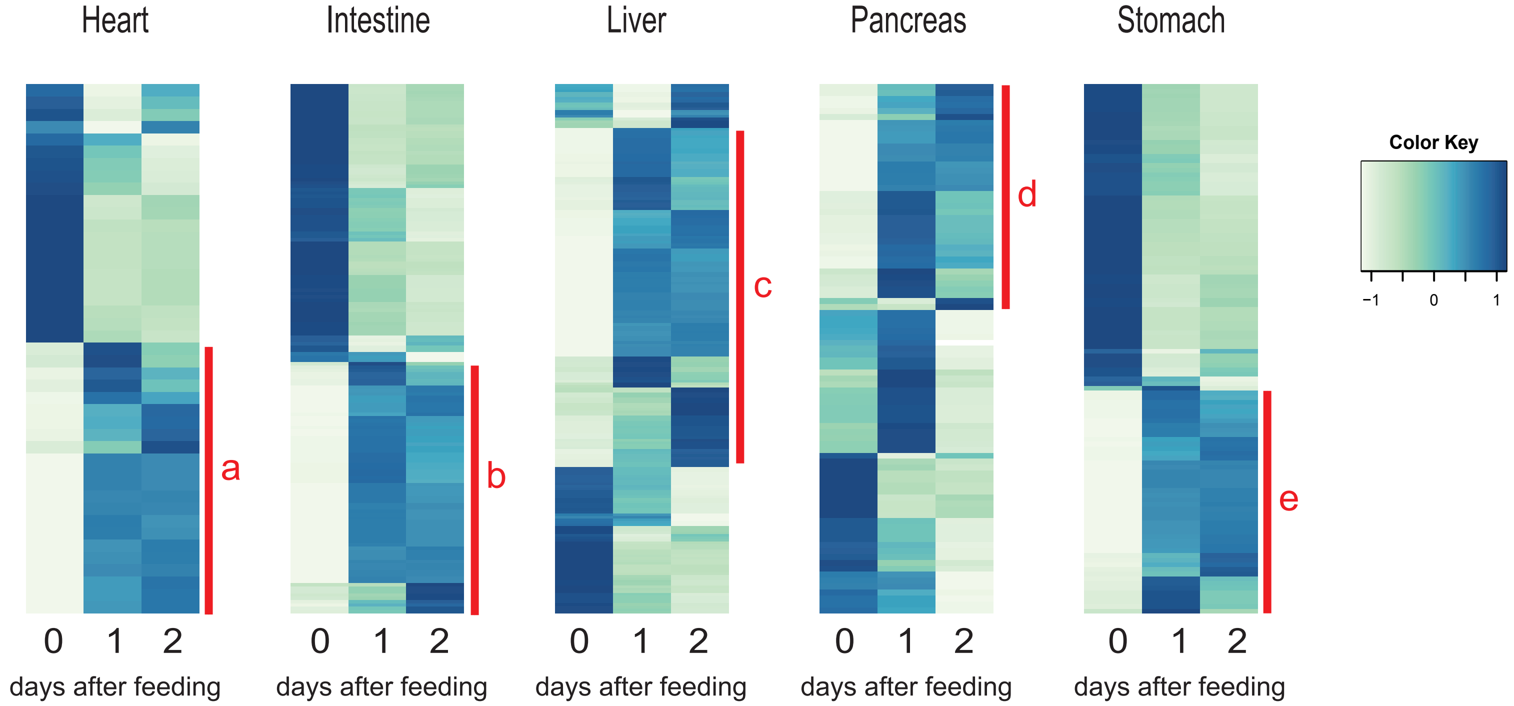

Figure 4

Click here to download Figure Figure4.pdf

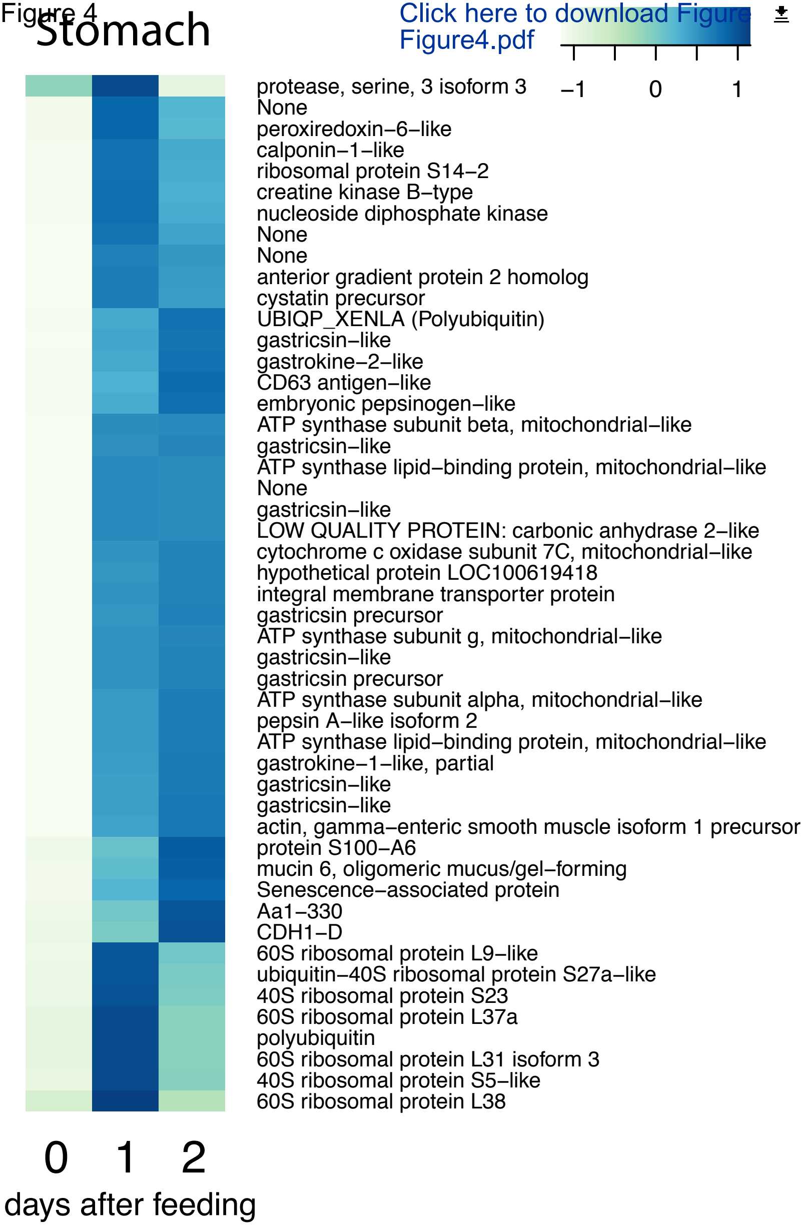

Figure 5

Click here to download Figure Figure5.pdf

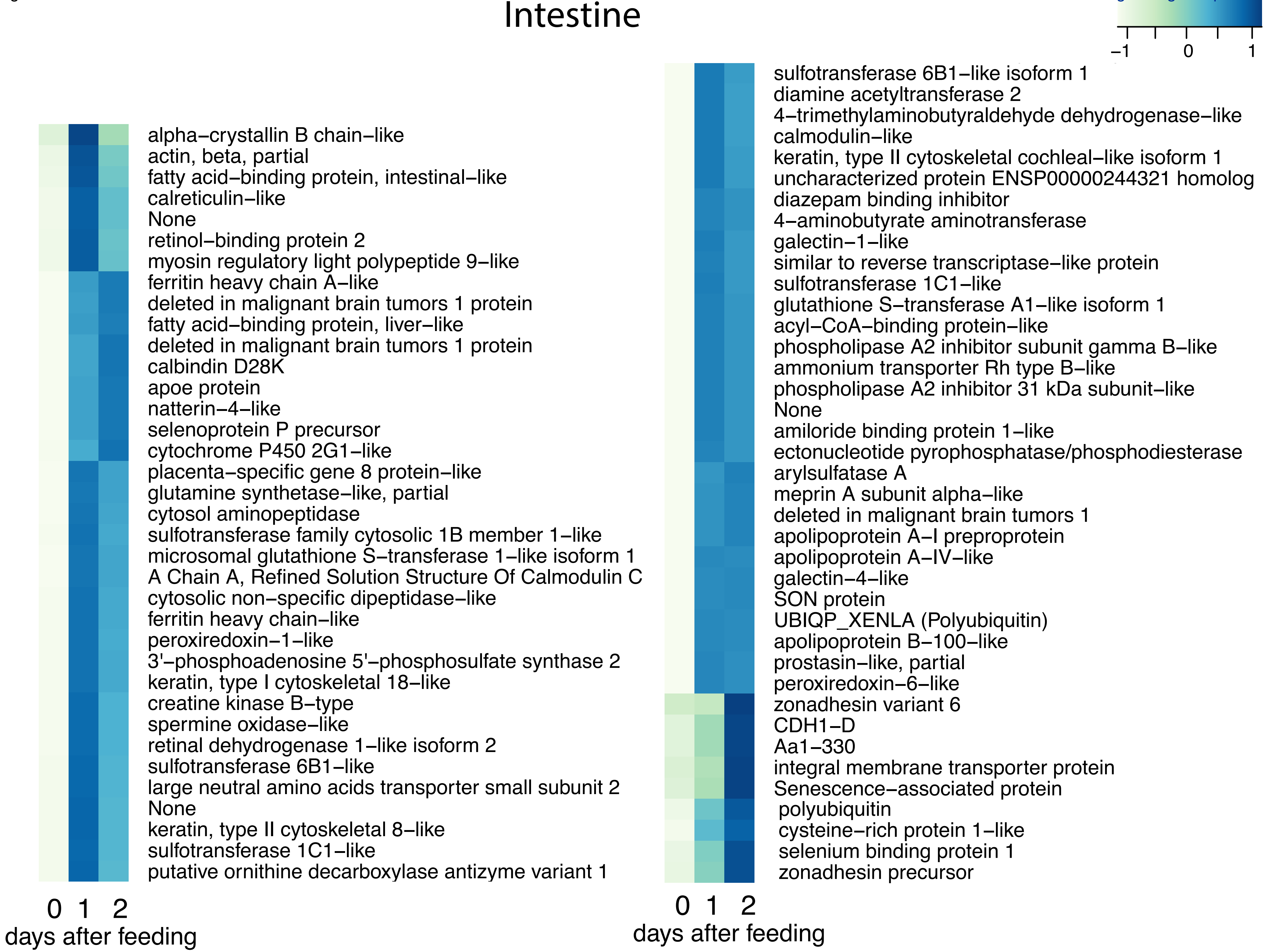

Figure 6 Click here to download Figure Figure6.pdf

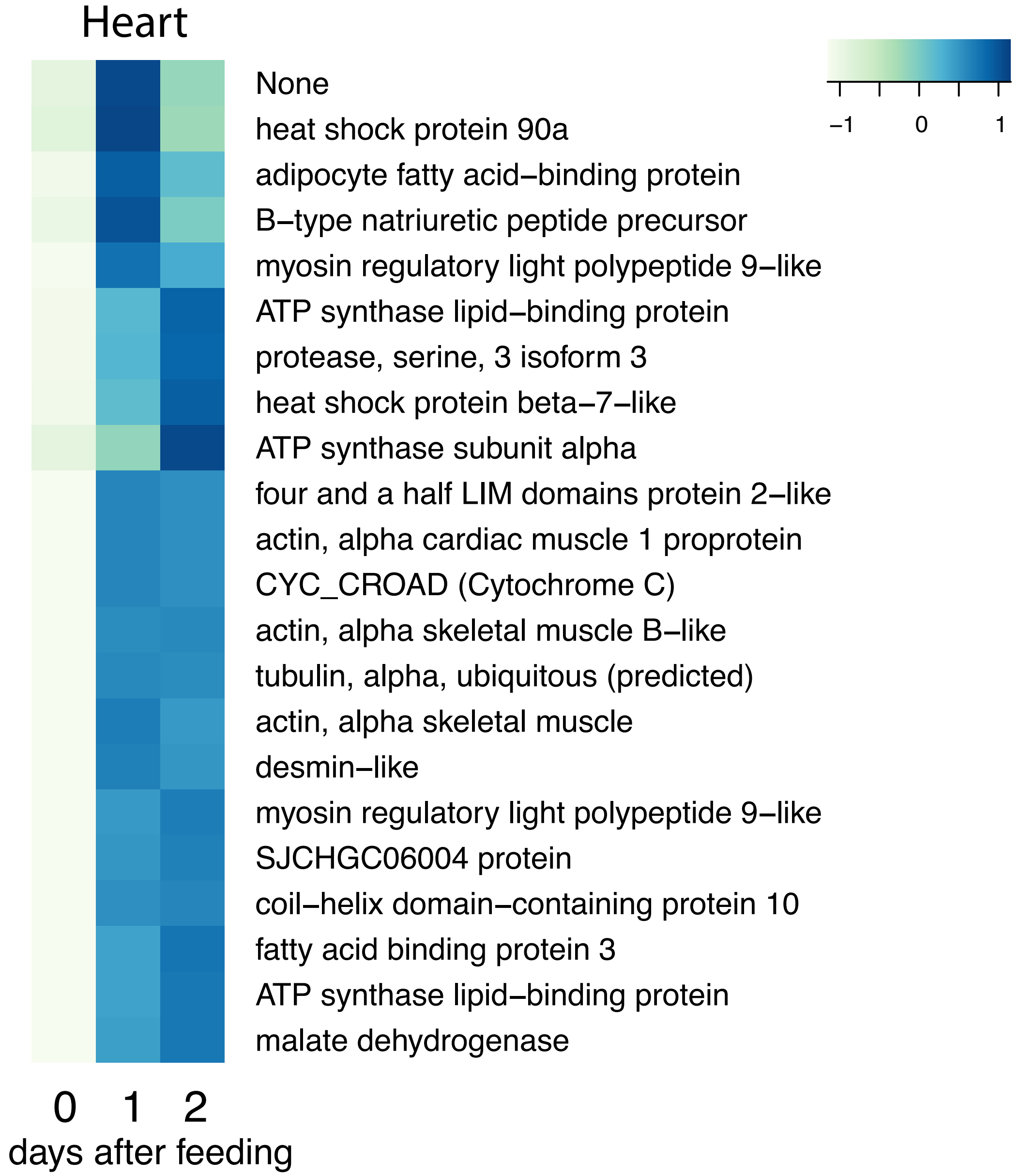

Figure 7

Click here to download Figure Figure7.pdf

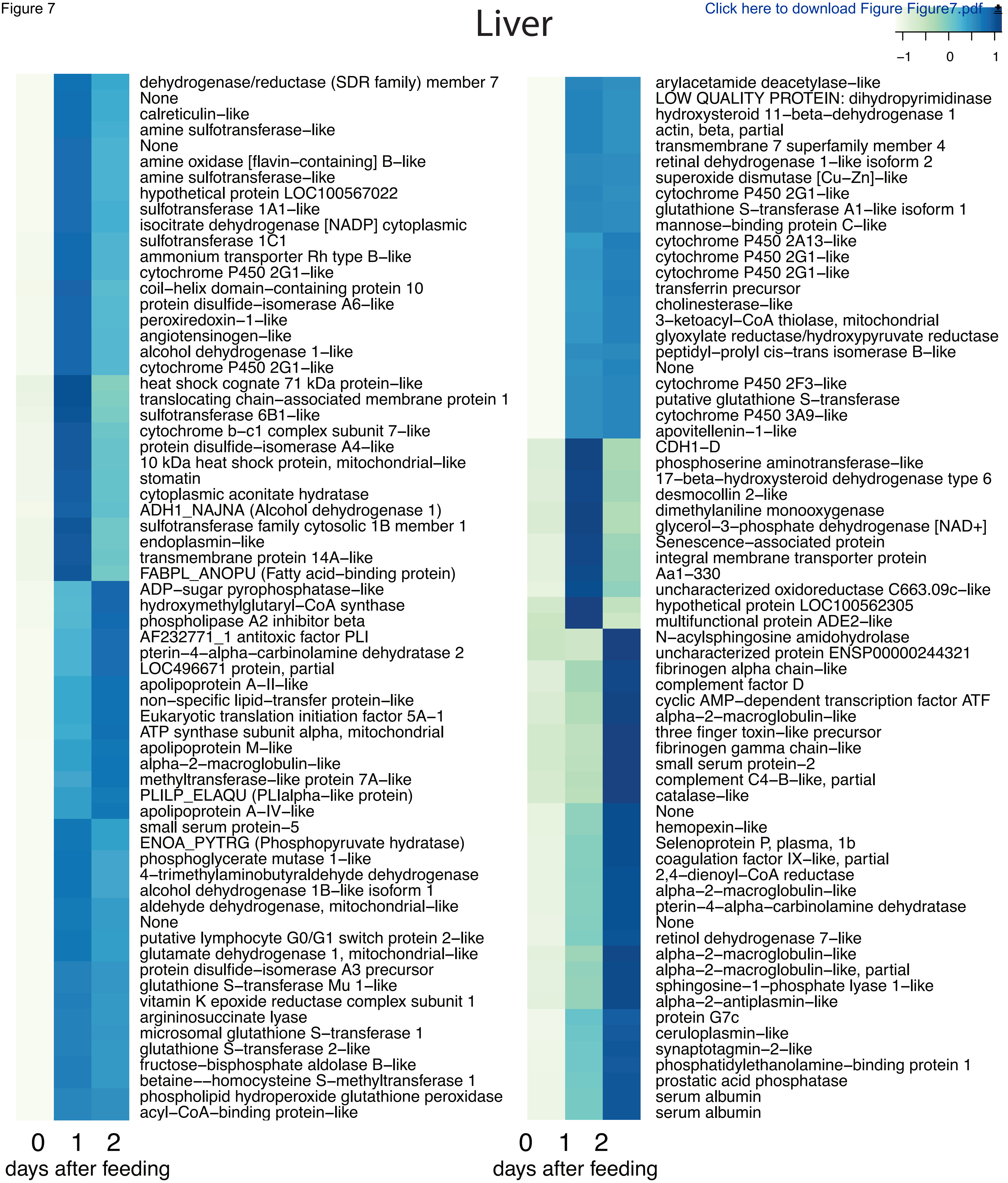

Figure 8

Click here to download Figure Figure8.pdf

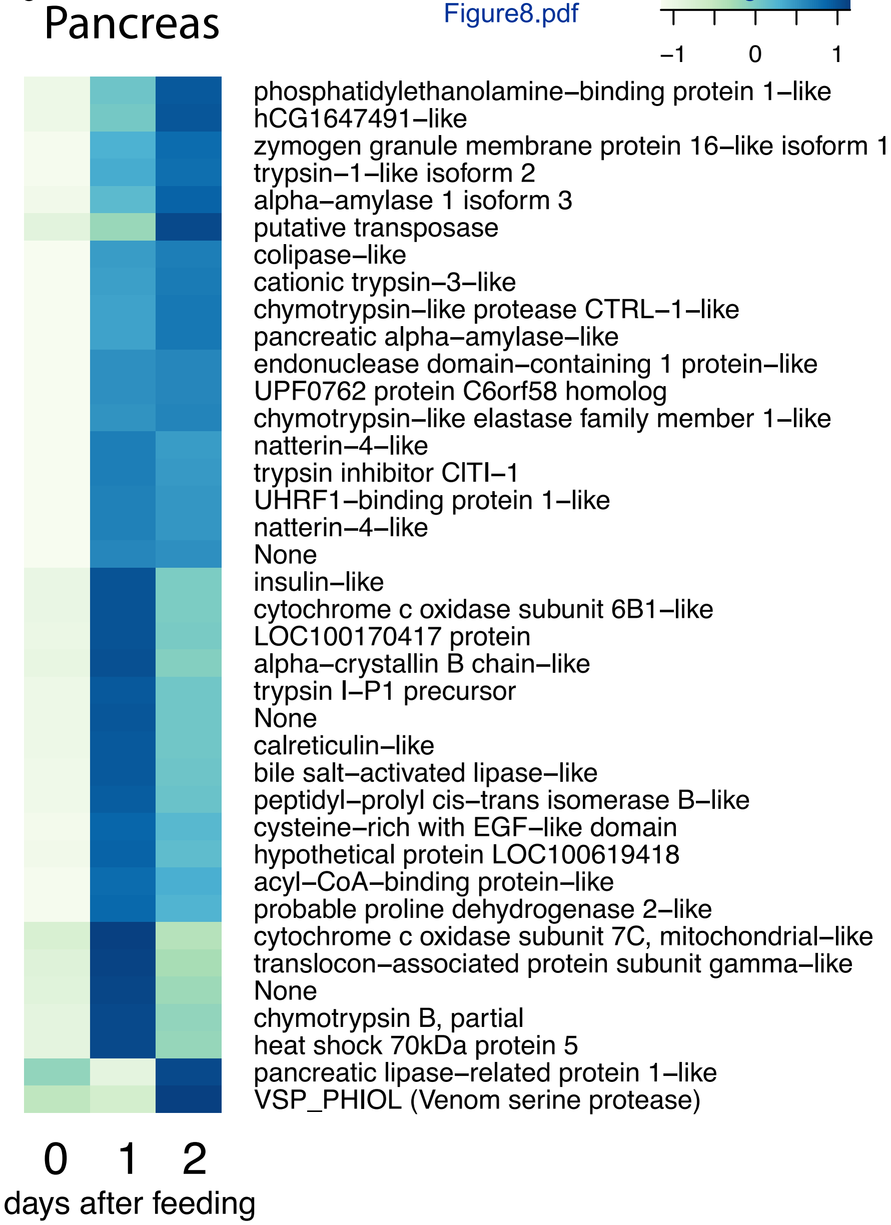

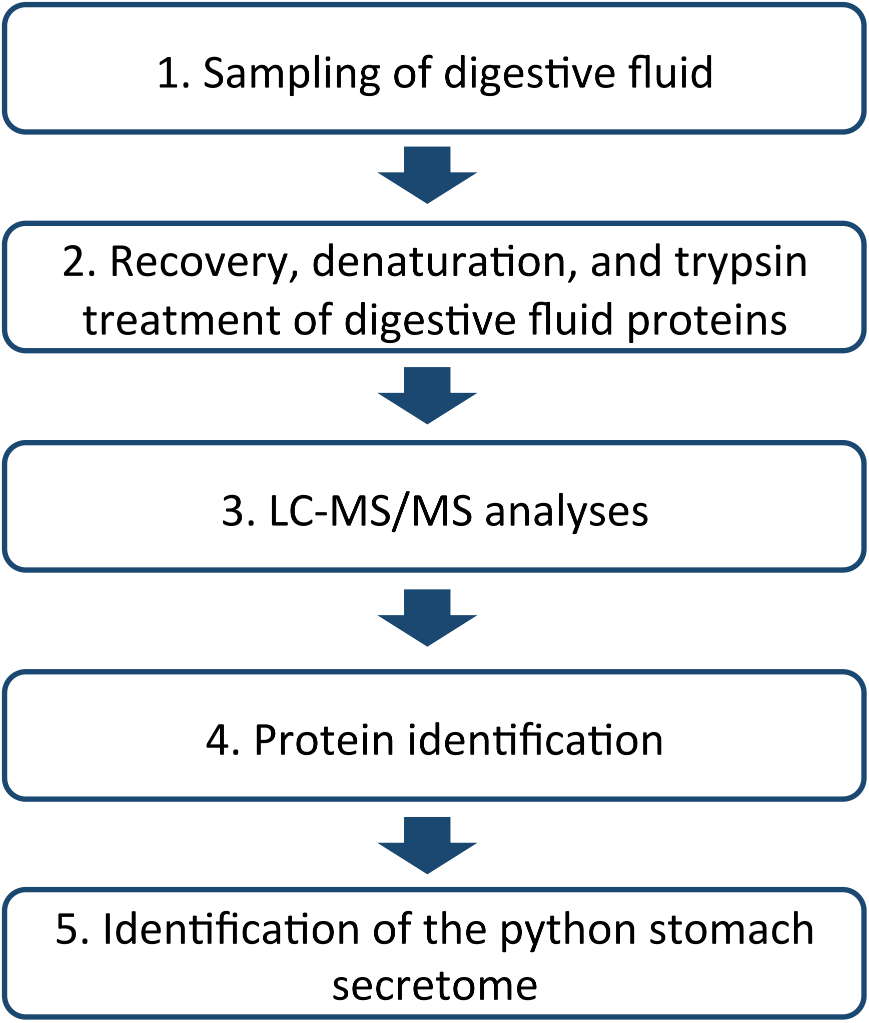
1. Sampling of digestive fluid
2. Recovery, denaturation, and trypsin treatment of digestive fluid proteins
3. LC-MS/MS analyses
4. Protein identification
5. Identification of the python stomach secretome

Figure 10

Click here to download Figure Figure10.pdf

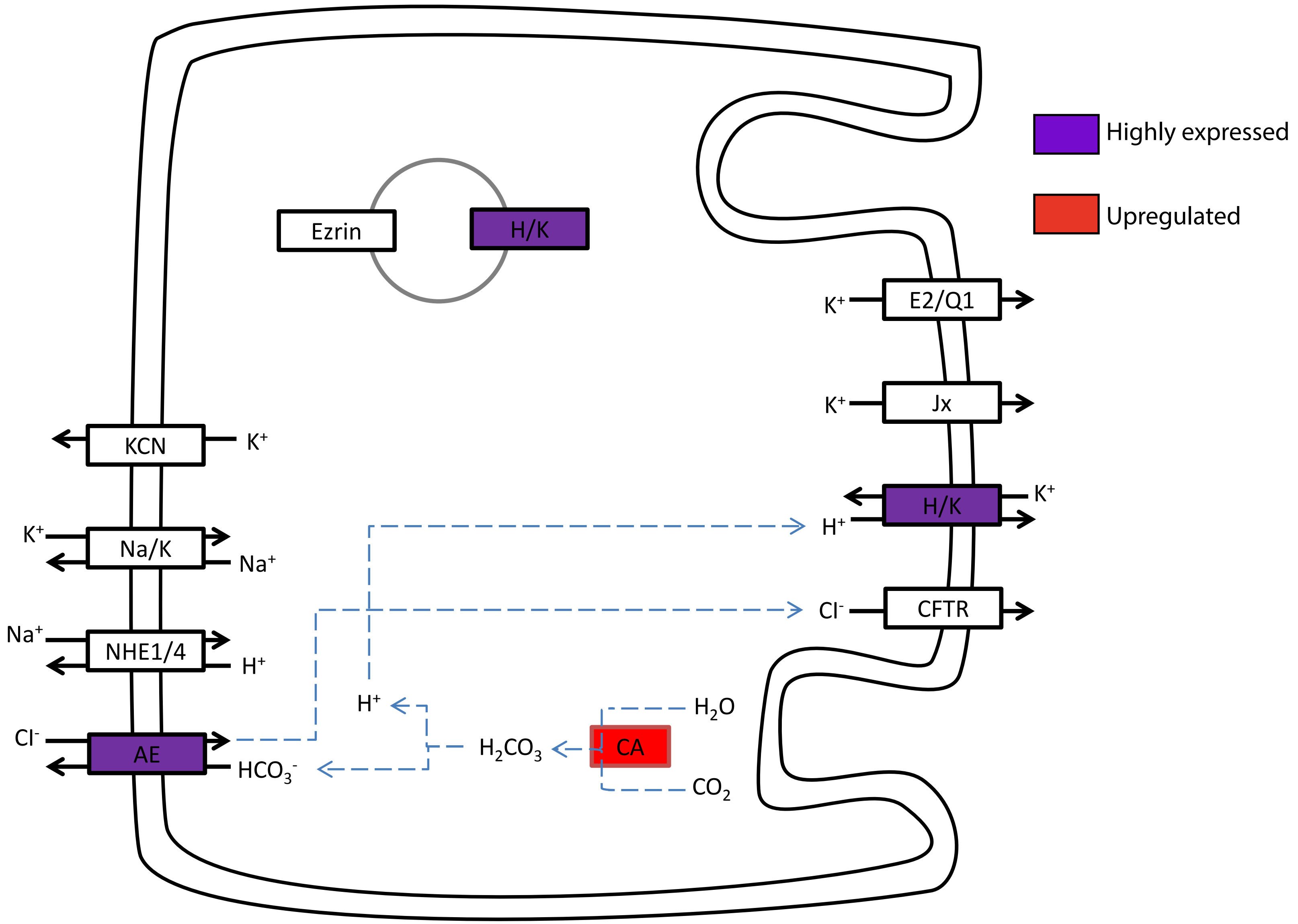

Click here to access/download
Supplementary Material
supplementary material.docx

FASTA sequences of misassembled transcripts

Click here to access/download
Supplementary Material
AU_assembly.misassembled.fasta

Figure S4

Click here to access/download
Supplementary Material
FigureS4.pdf

Figure S5

Click here to access/download
Supplementary Material
FigureS5.pdf

Figure S6

Click here to access/download
Supplementary Material
FigureS6.pdf

Figure S7

Click here to access/download
Supplementary Material
FigureS7.pdf

Figure S8

Click here to access/download
Supplementary Material
FigureS8.pdf

Figure S9

Click here to access/download
Supplementary Material
FigureS9.pdf

Table S3

Click here to access/download
Supplementary Material
TableS3.xlsx

Table S5

Click here to access/download
Supplementary Material
TableS5.xlsx

Table S6

Click here to access/download
Supplementary Material
TableS6.xlsx

Table S7

Click here to access/download
Supplementary Material
TableS7.xlsx

Table S8

Click here to access/download
Supplementary Material
TableS8.xlsx

Table S10

Click here to access/download
Supplementary Material
TableS10.xlsx

Table S12

Click here to access/download
Supplementary Material
TableS12.xlsx

Table S13

Click here to access/download
Supplementary Material
TableS13.xlsx

Table S14

Click here to access/download
Supplementary Material
TableS14.xlsx

Table S9

Click here to access/download
Supplementary Material
TableS9.xlsx

Table S11

Click here to access/download
Supplementary Material
TableS11.xlsx

 Click here to download Personal Cover GIGA-D-16-00152_R1_with_answers.pdf

GigaScience

Transcriptome Analysis of the Response of Burmese Python to Digestion

--Manuscript Draft--

**Manuscript Number:**	GIGA-D-16-00152R1
**Full Title:**	Transcriptome Analysis of the Response of Burmese Python to Digestion
**Article Type:**	Research
**Abstract:**	Background:<br>The exceptional and extreme feeding behaviour makes the Burmese python a unique and interesting model to study physiological remodelling and metabolic adaptation in response to feeding after prolonged starvation. With outset in specific hypotheses based on in vivo physiological responses, we use transcriptome sequencing of five visceral organs and three digestive stages to unravel the patterns of changes in the gene expression of Burmese python upon ingestion of a large meal. We first used the combined data to perform a de novo assembly of the transcriptome. We supplemented with a proteomic survey of enzymes in the gastric juice, stomach secretome and plasma during digestion assisted by our transcriptome sequence database.<br>Results:<br>We constructed a high-quality transcriptome with 34,423 transcripts of which 19,713 (57%) were annotated. Among highly expressed genes (FPKM>100 in one tissue) we found differential expression for 43 genes in heart, 206 genes in liver, 114 genes in stomach, 89 genes in pancreas and 158 genes in intestine. We interrogated the function of these genes in order to test previous hypothesis on the response to feeding. We also used the transcriptome to identify 314 secreted proteins in the python gastric juice.<br>Conclusions:<br>We provide comprehensive transcriptome data of multiple organs and various digestive time of Burmese python and address specific hypothesis on certain pathways known to related digestion process. We also identify, for the first time, stomach-related proteins from a digesting individual and thereby demonstrate that the sensitivity of modern LC-MS/MS equipment allows the identification of gastric juice proteins that are present during digestion thereby providing novel insight into the digestion mechanism.
**Response to Reviewers:**	Dear Editor,<br><br>Thank you for returning the constructive and useful comments from the two reviewers that kindly evaluated our manuscript entitled “Transcriptome Analysis of the Response of Burmese Python to Digestion” that we submitted for publication in GigaScience. Both reviewers provided positive overall assessments, but also raised a number of specific queries to be addressed in the revision. We are pleased to return a revised manuscript where we have followed all the advice given by the two reviewers. The responses to each query is listed in a separate PDF file entitled "Answer sheet to reviewers comments" attached as personal cover where you can see our responses describing the changes we have made to the manuscript. We greatly appreciate these comments and feel the manuscript has been improved in this review-process. We hope you will find the revised manuscript acceptable for publication in GigaScience and we are looking forward to hearing from you in due course. Please do not hesitate to contact me in case you need additional information.<br>Sincerely yours<br>Jinjie Duan (on behalf of all the authors)
**Additional Information:**	
**Question**	**Response**
Are you submitting this manuscript to a special series or article collection?	No
**Experimental design and statistics**<br><br><br>Full details of the experimental design and statistical methods used should be given	Yes

Powered by Editorial Manager® and ProduXion Manager® from Aries Systems Corporation

in the Methods section, as detailed in our Minimum Standards Reporting Checklist. Information essential to interpreting the data presented should be made available in the figure legends.<br><br>Have you included all the information requested in your manuscript?	
**Resources**<br><br>A description of all resources used, including antibodies, cell lines, animals and software tools, with enough information to allow them to be uniquely identified, should be included in the Methods section. Authors are strongly encouraged to cite Research Resource Identifiers (RRIDs) for antibodies, model organisms and tools, where possible.<br><br>Have you included the information requested as detailed in our Minimum Standards Reporting Checklist?	Yes
**Availability of data and materials**<br><br>All datasets and code on which the conclusions of the paper rely must be either included in your submission or deposited in publicly available repositories (where available and ethically appropriate), referencing such data using a unique identifier in the references and in the “Availability of Data and Materials” section of your manuscript.<br><br>Have you have met the above requirement as detailed in our Minimum Standards Reporting Checklist?	Yes

Manuscript

Click here to download Manuscript Transcriptome analysis of Burmese python.docx

Transcriptome Analysis of the Response of Burmese Python to Digestion

Jinjie Duan[1], Kristian Wejse Sanggaard[2,3], Leif Schauser[5], Sanne Enok Lauridsen[4], Jan J. Enghild[2,3], Mikkel Heide Schierup[1,4] and Tobias Wang[4]

1. Bioinformatics Research Center, Aarhus University, Denmark

2. Department of Molecular Biology and Genetics, Aarhus University, Denmark

3. Interdisciplinary Nanoscience Center (iNANO), Aarhus University, Denmark

4. Department of Bioscience, Aarhus University, Denmark

5. QIAGEN Aarhus, Silkeborgvej 2, 8000 Aarhus C, Denmark

Corresponding authors: Jinjie Duan, Mikkel Heide Schierup & Tobias Wang

Email address:

Jinjie Duan: jjduan@birc.au.dk

Kristian Wejse Sanggaard: kristian@wejsesanggaard.dk

Leif Schauser: Leif.Schauser@qiagen.com

Sanne Enok Lauridsen: sanne.lauridsen@inano.au.dk

Jan J. Enghild: jje@mbg.au.dk

Mikkel Heide Schierup: mheide@birc.au.dk

Tobias Wang: tobias.wang@bios.au.dk

Abstract

Background:

The exceptional and extreme feeding behaviour makes the Burmese python a unique and interesting model to study physiological remodelling and metabolic adaptation in response to feeding after prolonged starvation. With outset in specific hypotheses based on in vivo physiological responses, we use transcriptome sequencing of five visceral organs and three digestive stages to unravel the patterns of changes in the gene expression of Burmese python upon ingestion of a large meal. We first used the combined data to perform a *de novo* assembly of the transcriptome. We supplemented with a proteomic survey of enzymes in the gastric juice, stomach secretome and plasma during digestion assisted by our transcriptome sequence database.

Results:

We constructed a high-quality transcriptome with 34,423 transcripts of which 19,713 (57%) were annotated. Among highly expressed genes (FPKM>100 in one tissue) we found differential expression for 43 genes in heart, 206 genes in liver, 114 genes in stomach, 89 genes in pancreas and 158 genes in intestine. We interrogated the function of these genes in order to test previous hypothesis on the response to feeding. We also used the transcriptome to identify 314 secreted proteins in the python gastric juice.

Conclusions:

We provide comprehensive transcriptome data of multiple organs and various digestive time of Burmese python and address specific hypothesis on certain pathways known to related digestion process. We also identify, for the first time, stomach-related proteins from a

digesting individual and thereby demonstrate that the sensitivity of modern LC-MS/MS equipment allows the identification of gastric juice proteins that are present during digestion thereby providing novel insight into the digestion mechanism.

Keywords:

Burmese Python, transcriptome, tissue expression, digestion, pathway, proteome

Background

All animals exhibit dynamic changes in the size and functional capacities of bodily organs and tissues to match energetic maintenance costs to the prevailing physiological demands [1]. This phenotypic flexibility is particularly pronounced for the digestive organs in animals that naturally experience prolonged periods of fasting, but capable of ingesting large prey items at irregular intervals. The Burmese python is an iconic example of this extreme phenotype [1]. Many species of pythons easily endure months of fasting, but remain capable of subduing and ingesting very large meals. In Burmese pythons, digestion is attended by a large and rapid rise in mass and/or functional capacities of the intestine, stomach, liver, heart and kidneys [2-4] in combination with a stimulation of secretory processes and an activation of enzymes and transporter proteins. These physiological responses are associated with a many-fold rise in aerobic metabolism. Hence, the Burmese python is an excellent model to study the mechanisms underlying extreme metabolic transitions and physiological remodelling in response to altered demand [1, 3, 5-10].

The postprandial changes in the morphology and physiology of the intestine, heart and other organs have been described in some detail in pythons [1, 5, 8, 9, 11], but only a few studies [12-14] have addressed the underlying transcriptional changes of this interesting biological response. Transcriptome sequencing technology now allows comprehensive surveys [15, 16], and we therefore decided to use transcriptome sequencing of heart, liver, stomach, pancreas and intestine in snakes that had fasted for one month and at 24 and 48h into the postprandial period. These organs were chosen because a number of earlier studies reveal profound phenotypic changes during the postprandial period [1-4, 17], and are therefore likely to exhibit large changes in gene expression. Differential gene expression in some of these organs have previously been reported [12-14], but we provide new data on 48h into the

digestive period and the first descriptions of gene expression in the stomach and the pancreas. As the Burmese python reference genome assembly [12] ~~currently~~ is relatively fragmented (contig size N50 ~10kb), we found it impractical to use re-sequencing approaches and opted instead to use our high coverage data to build a *de novo* transcriptome assembly to identify differentially expressed genes (DEGs). To identify the enzymes involved in the digestion process, we initiated digestion, then isolated the digestive fluid and characterized the protein composition using a proteomics-based approached. This also allowed us to identify the major hydrolytic enzymes used to digest the large and un-masticated meals.

Analyses

Data summary

277,485,924 raw paired reads (2*101 bp, insert size 180 bp) were obtained from Illumina Hi-Seq 2000 sequencing of 15 non-normalized cDNA libraries derived from five tissues (heart, liver, stomach, pancreas and intestine) at three time points (fasted for one month, 24h and 48h post-feeding) and 10 DSN-normalized cDNA libraries (see methods) (Supplementary Table S1). After removal of low-quality reads (See methods), 213,806,111 (77%), high-quality paired reads were retained. These reads contained a total 43,146,073,200 bp nucleotides with a mean Phred quality higher than 37 (Q37). To develop a comprehensive transcriptomics resource for the Burmese python (Fig. 1), we pooled these high-quality reads from 25 libraries for subsequent *de novo* assembly.

de novo transcriptome assembly and evaluation

As short k-mers have a higher propensity to generate misassembled transcripts when using a de Bruijn graph-based *de novo* assembler, such as Velvet [18], we conservatively chose an assembly generated using long k-mers for subsequent analysis, at the cost of some sensitivity regarding assembled isoforms. Thus, balancing key metrics (Supplementary Table S2), we used an assembly based on the longest k-mer = 95 (Table 1), as it had the fewest scaffolds/transcripts (34,423), but represented a very large proportion (74%) of all reads. The scaffold N50 of this assembly was 1,673 bp.

To evaluate the accuracy of the transcriptome assembly, we compared it with the Burmese python reference genome (GenBank assembly accession: GCA_000186305.2) and corresponding gene set in NCBI database using rnaQUAST v1.4.0 [19]. The transcriptome assembly had 34,423 transcripts in total. 34,040 (98%) of these transcripts had at least one

significant alignment to the reference genome, and 31,102 (91%) out of 34,040 were uniquely aligned (Supplementary table S3). Average aligned fraction (i.e. total number of aligned bases in the transcript divided by the total transcript length) was 0.975 (Supplementary table S3). The high concordance between the *de novo* transcript assembly and genome reference strengthened our confidence in using *de novo* assembly as our reference, and shows that the individual fragments were accurate although the reference genome assembly is fragmented. By aligning assembled sequences back to reference genome, we checked the chimeric assembled sequences which have discordant best-scored alignment (partial alignments that are either mapped to different strands/different chromosomes/in reverse order/too far away) and found 1,974 (5.7%) misassembled (chimeric) transcripts (Supplementary table S3) which sequences were stored in a supplementary FASTA file. The comparison of assembled sequences and reference gene sequences (Supplementary table S3) showed that 26,320 (77.3%) assembled transcripts cover at least one isoform from the reference gene set and the mean fraction of transcript matched is 67.8%, suggesting there is a good concordance but also some differences which can be due to errors in either the reference genome assembly/annotation or our assembly. In addition, we assessed the completeness of our transcriptome assembly with the Benchmarking Universal Single-Copy Orthologs (BUSCO) strategy. Results showed 55.2% (1,428 out of 2,586) complete BUSCOs, 19.8% (512) fragmented BUSCOs and 25% (646) missing BUSCOs. These results are consistent with the survey [20] of assessment completeness of 28 transcriptomes from 18 vertebrates. In this survey, most of transcriptomes from species with close phylogenetic relationship to snake contain less than 50% complete BUSCOs and more than 40% missing BUSCOs. Therefore, we conclude the quality of our transcriptome assembly was ~~well~~ acceptable.

Transcriptome annotation

19,713 transcripts (57% of 34,423) were annotated using transfer of blastx hit annotation

against the non-redundant (nr) NCBI peptide database [21]. To assign proper annotation for each transcript, we chose the first best hit that was not represented in uninformative descriptions (Supplementary Table S4). The most closely related species with an annotated genome, *Anolis carolinensis* was able to annotate 10,704 transcripts (54% of all annotated transcripts). Burmese Python and *Anolis carolinensis* both belong to the reptilian Squamata order, and are separated by approximately 120 million years of evolution [22].

Blast2GO [23] then annotated these 19,713 transcripts, and 16,992 of them could be assigned by one or more GO terms and putative functional roles were described. The distributions of the most frequently identified GO terms categories for biological process (BP), molecular function (MF) and cellular component (CC) are shown in Fig. S1. Moreover, we used the functionality of InterPro [24] annotations in Blast2GO to retrieve domain/motif information for our transcripts, and 21,023 transcripts were annotated by the InterPro database.

Gene expression analysis and principal component analysis

For comparisons between genes, expression profiles were obtained by mapping high quality reads to the reference transcriptome and the expression level was given by fragments per kilo base per million sequenced reads (FPKM) [25]. For the study of expression profiles, we chose to investigate 1862 highly expressed genes (FPKM >= 100 in at least one tissue of 15), as it is known that for highly expressed genes, the biological variation among biological replicates in the same tissue at the same stage is lower than for genes showing low expression levels [26]. The majority (~64%) of these 1862 genes were expressed in all tissues, and only ~18% were expressed solely in one tissue (Supplementary Fig. S2). The liver had the highest number of uniquely expressed genes, which may reflect its particular role in metabolism and excretion of waste products.

We used principal component analysis (PCA) to reveal overall differences in gene expression patterns among tissues and time points within the digestive period. The first three principal components (PCs) accounted for ~58% of the variation (Supplementary Fig. S3). Despite the large overlap in expressed genes (Supplementary Fig. S2), the different tissues exhibited distinct transcriptional signatures shown by the PCA in Figure 2, showing a tendency for 24h to represent an intermediate position between fasting and 48h. Liver, intestine and stomach displayed greater shifts in the PCA plots compared to heart and pancreas, and the largest changes occurred between fasting and 24h in the stomach and intestine. This fits well with the expectation that the stomach and intestine respond early in digestion [3]. The dramatic changes in gene expression in the liver are also consistent with previous observations on pythons [12].

Pattern of transcriptional responses to feeding

The postprandial response involves thousands of genes and large changes in gene expression. To restrict the analysis of these many genes, we used a conservative approach where we selected genes that are both highly and differentially expressed with two strict thresholds (see methods). Application of these two thresholds yielded 43 genes for heart, 206 genes for liver, 114 genes for stomach, 89 genes for pancreas and 158 genes for intestine, respectively, that were differentially expressed in response to digestion (Fig. 3). To illustrate in greater detail, we enlarged the five sub-clusters with the most prominent increase in expression. These sub-clusters, labelled a - e in Figure 3, are shown with full annotation in Figures 4-8. To unravel the functional implications of these responses, we searched for genes encoding for proteins involved in processes of tissue re-organization, cellular metabolism and digestion within these sub-clusters for each organ.

GO enrichment analysis and colored KEGG pathway maps

To get a broader biological insight, compared to the strict threshold set used in the above clustering analysis, we applied a looser threshold set (Table 2) of defining DEG and highly expressed genes for functional annotation analysis. The summary of number of DEGs during digestion in each tissue is illustrated in Table 3. In each organ, most ~~of~~ genes (> 76%) have low expression (max FPKM < 10). Around 1% of the genes are highly expressed (max FPKM >= 200). The number of upregulated genes is approximately 3% in each organ, except for the heart where only 0.57% of the genes were upregulated in response to feeding. This suggests that during digestion, the digestive organs, like liver, stomach, intestine and pancreas show more pronounced post feeding response than the heart. To dissect the functions of DEGs, we performed GO enrichment analysis with upregulated genes and highly expressed genes respectively for each organ (Supplementary Figs. S4-S8). As an example, the most significantly associated GO term to upregulated genes in stomach was “mitochondrial respiratory chain complex 1”, “endoplasmic reticulum membrane” and “cytosol” (Supplementary Fig. S4A).

To specifically identify the pathways associated to DEGs and highly expressed genes, we mapped genes to KEGG [27, 28] human pathway maps and colored the mapped entries with trend of gene expression during digestion (Table 2). We identified upregulated genes and highly expressed genes, respectively, involved in three selected pathways (glycolysis/gluconeogenesis, citrate cycle (TCA cycle), and oxidative phosphorylation) for each tissue (Supplementary table S5), and we performed the same identification for two main pathway categories in the KEGG pathway database (1.3 lipid metabolism and 1.5 amino acid metabolism; Supplementary table S6). The glycolysis/gluconeogenesis pathway, glyceraldehyde-3 phosphate dehydrogenase showed high expression in all organs.

Identification of the python gastric juice proteome

We identified the secretome of the python stomach during digestion (Fig. 9). The resulting mass spectrometry data (containing 122538 MS/MS spectra) was used to interrogate our python transcriptome database, which includes transcriptome from stimulated stomach tissue. In total, 549 python proteins were identified using this approach. Afterwards, all identifications based on a single tryptic peptide were removed reducing the number of identified python proteins to 314 (Supplementary Table S7).

Five classical types of pepsinogens exist, namely pepsinogen A, B, and F, progastricsin (or pepsinogen C), and prochymosin [29]. Of these, our analyses (Supplementary table S8 and S9) show that pythons primarily rely on progastricsin for proteolytic digestion, as the five most abundant proteases identified in the gastric juice are annotated as progastricsin-like. Alignment of the sequences of the various transcripts for gastricsin-like proteins shows considerable differences in sequence, which indicate the presence of numerous different proteins with similar functions. This annotation is based on accession XP_003220378.1 and XP_003220378.1 from *Anolis carolinensis.* Alignment of the python sequences with the two anole sequences, as well as with the well-characterized human gastricsin variant, shows that both the active site residues, as well as cysteine bridges, are conserved. It demonstrates the similarity between these enzymes and suggests that the identified python sequences indeed represent catalytically active proteolytic enzymes (Supplementary Fig. S9). The last identified pepsinogen-like python sequence (m.31615_Py95) was annotated based on the predicted embryonic pepsinogen-like sequence (XP_003220239.1), also from *Anolis carolinensis.* Here, the annotation originates from an embryonic pepsinogen identified in chicken [30]. This protease was identified in the python's gastric juice with a lower emPAI value than the gastricsin sequences indicating a lower concentration of this enzyme (Supplementary table S8), although the transcript displays the highest concentration of the analysed pepsinogens in the post-prandial period (Supplementary

table S9). As the name indicate it is exclusively expressed during the embryonic period [30, 31], and phylogenic analysis of the sequence suggest that its closest homolog, among the classical pepsinogens, is prochymosin [30]. Also, prochymosin displays a temporal expression pattern and is, in mammals, mainly expressed in new-born species. However, the identified python snake embryonic chicken pepsinogen homolog does not display a similar development-related temporal expression pattern and is, as shown, used among adult species for digestion. However, it does not exclude that the protease is expressed during the python's embryonic phase.

Identification of prey proteins and the python plasma proteome

Many of the obtained MS/MS spectra were expected to correspond to abundant mice proteins, such as collagen. To facilitate the downstream analyses of the python proteins, we produced a list of background proteins related to the prey. Hence, interrogation of the mass spectrometry data against the 16693 mouse protein sequences in the Swiss-Prot database resulted in the identification of 212 mouse proteins, after removing hits based on single peptides (Supplementary table S10). To produce a list of identified python proteins, most likely present in the digestive fluid samples due to blood contaminations during collection, we characterized the python plasma proteome. The most abundant plasma proteins are produced by the liver. Consequently, our python transcriptome sequence database, which encompasses liver transcriptomes, is expected to contain the protein sequences of the python plasma proteins. Thus, our python plasma LC-MS/MS data was used to interrogate our python sequence database. It provided an overview of the most abundant python plasma proteins (Supplementary table S11). In total, 64 plasma proteins were identified with minimum two tryptic peptides. The result supports the liver transcriptome data, since the abundant (based on emPAI) plasma proteins correlate with the transcripts that are detected at

high concentration in the liver tissue. The overall protein composition is similar to the composition in humans with albumin, fibrinogen, alpha-2-macroglobulin, immunoglobulins, complement factors and apolipoproteins being the dominating proteins. One protein that stands out is the anti-haemorrhagic factor cHLP-B (m.27_Py95), which appeared in high concentration in the plasma of these snakes. This is a protease inhibitor of the haemorrhagic-causing metalloproteinases present in snake venom and these inhibitors have previously been purified from serum of venomous snakes and thoroughly characterized [32, 33]. The role of such a protease inhibitor in non-venomous pythons is not obvious, but it has been proposed that they inhibit the deleterious action of venom enzymes in non-venomous snakes [34].

Identification of the python stomach secretome

To identify the python stomach secretome, the list of python proteins, identified in the digestive fluid (Supplementary table S7) was analysed further. We assumed no overlap between abundant plasma proteins and proteins secreted by the stomach. Thus, plasma proteins, identified in the gastric juice, were assumed to be contaminations from blood and therefore the 64 identified plasma proteins were, when present, removed from the list. Subsequently, python proteins that most likely were identified based on prey proteins homology (*e.g.* python collagens and keratins, as well as conserved intracellular household proteins) were removed. These two steps reduced the list of proteins identified in the stomach samples from 314 to 114 proteins (Supplementary table S12). It cannot be excluded that a few proteins belonging to the python stomach secretome also were removed.

To identify the secretome, the 114 identified proteins were manually analysed as described in the method section (Supplementary table S12). In addition to household proteins, the identified intracellular proteins also included intracellular stomach-specific proteins (*e.g.* the stomach specific calpain 9 cysteine protease [35]), underlining the specificity of the

proteomics analysis. In total, 37 proteins constituted the putative python stomach secretome (Supplementary table S8). These could be divided into 18 gastric mucosal-related proteins (e.g. mucin homologous and gastrokine), seven proteolytic enzymes (mainly pepsin homologous), four other hydrolytic enzymes (e.g. phospholipases), and eight other proteins (e.g. gastric intrinsic factor) (Supplementary table S8). Previous gastric juice proteomics analyses were performed on samples obtained from fasting humans, most likely to avoid the complex prey-protein background. In our study, we identify, for the first time, stomach-related proteins from a digesting individual and thereby demonstrate that the sensitivity of modern LC-MS/MS equipment allows the identification of gastric juice proteins that are present during digestion.

Discussion

A primary motivation for our description of the temporal changes in gene expression profiles as the visceral organs of Burmese pythons made the transition from fasting to digestion was to identify key regulatory genes and pathways responsible for the pronounced tissue restructuring and the increased functional capacity during the postprandial period. An equally important motivation was to address specific hypothesis on the upregulation of certain pathways known to be involved in the secretion of digestive juices and enzymes as well as the absorption of the nutrients as digestion proceed. We achieved these goals by identifying the biochemical and physiological roles of the highly expressed genes with increased expression during digestion and by using KEGG analysis of specific pathways underlying physiological responses known to be stimulated by digestion. We also present GO enrichment analyses of both up-regulated genes and highly expressed genes in all organs (Supplementary Figs. S4-S8), showing that "biological process" is the most common enriched category.

The influence of digestion on gene expression profiles in heart, liver, kidney and small intestine has been studied previously in pythons [12-14]. These earlier studies reported thousands of genes being either up- or downregulated within the first day of digestion [12-14], and we confirm these substantial changes in gene expression at 24h and 48h. However, we merely identified hundreds of genes, probably because we selected a more stringent threshold for calling the differential expression. Given the differences in the selection of thresholds and analysis strategy for differential expression and differences in times of sampling, it is difficult to make a direct comparison between our study and that of Castoe et al (2013). Nevertheless, for heart, liver and small intestine, both studies have determined a number of upregulated genes at 24h where we identified 15, 93 and 61 upregulated genes,

respectively. Comparing upregulated genes between two studies (see supplementary material for detailed method and result), we found there was good overlap in identifying upregulated genes in the liver where more than half of the 93 genes identified in our study were identified as upregulated genes by Castoe et al (2013). However, there was less overlap for heart and the small intestine. These differences may be due to the use of different quantification methods for gene expression in the various studies, but may also be a result of the limited biological replicates in our study. Nevertheless, genes identified as being upregulated in both studies, are probably of high confidence.

Physiological interpretation of the upregulated genes in the stomach

The considerable changes in gene expression in the stomach were reflected in a pronounced rise in expression of ribosomal 40S and 60S proteins (Fig. 4) that is likely to have attended a rise in protein synthesis required for the marked transition from a quiescent fasting state to the activated digestive state. This is also supported by the presence of ribosomal functions in the enriched GO analysis of the stomach of the highly-expressed genes (Supplementary Fig. S4B). During fasting, gastric acid secretion and presumably also the secretion of digestive enzymes and lysozymes, is halted, such that the gastric juice has a neutral pH, whilst ingestion of prey is followed by an immediate activation of gastric acid secretion [36, 37]. The stimulation of the secretory actions of the stomach is attended by an increased mass of the stomach, where particularly the mucosa expands already within the first 24h [38].

The KEGG analysis, however, shows that the genes encoding for the gastric H,K ATPase, the active and ATP consuming ion-transporter responsible for gastric acid secretion, are highly expressed in fasting animals, and not additionally elevated in the postprandial period (Fig. 10). This strongly indicates that the enzymatic machinery for gastric acid secretion is maintained during fasting, a trait that may enable fast activation of acid secretion,

at modest energetic expenditure, to kill bacteria and match gastric pH to the optimum value for pepsin. This interpretation is consistent with a number of recent studies indicating a rather modest contribution of gastric acid secretion to the specific dynamic action (SDA) response in pythons [39, 40], but we also did observe a high prevalence of ATP synthase subunits (Fig. 4) amongst the highly upregulated genes, which does indicate a rise in aerobic metabolism (see also supplementary Fig. S4). Furthermore, the upregulation of the gene encoding for creatine kinase (Fig. 4) indicate increased capacity for aerobic respiration required costs of acid secretion and the stimulation of the accompanying gastric functions. It has been proposed that gastric processes account for more than half of the rise in total metabolism during digestion [36], and aerobic metabolism of isolated gastric strips *in vitro* increased during digestion [41]. However, while metabolism of the stomach certainly must increase during the postprandial period, more recent studies indicate a considerably smaller contribution of gastric acid secretion to the total SDA response is considerable lower than 50% [39, 40, 42].

Our KEGG analysis also showed a large rise in expression of the gene encoding for carbonic anhydrase (Fig. 10), the enzyme that hydrates CO_2 and provide protons for gastric acid secretion. Gastric acid secretion, therefore, does not appear to under transcriptional regulation, but is likely to involve translocation of existing H,K ATPases in vesicles from intracellular vacuoles to the apical membrane of the oxyntopeptic cells that are responsible for both gastric acid secreting as well as the release of pepsinogen in reptiles [43]. An activation of the processes involved in vesicle transport is further supported by increased transcription of the gene encoding for CD63 (Fig. 4), which belongs to the tetraspanin family and mediate signal transduction events.

In contrast to acid secretion, expression of several genes encoding for digestive

enzymes (embryonic pepsinogen-like, gastricsin precursor and gastricsin-like) (Fig. 4) were upregulated, which is consistent with *de novo* synthesis of the enzymes responsible gastric protein degradation. Also, there was good overlap between the upregulation of the relevant genes encoding for the proteins identified in the stomach secretome, such as gastrokines, pepsin homologous, phospholipases and gastric intrinsic factor (Supplementary table S8). In this context, it is also interesting that mucin 6 (Fig. 4), the gene coding for the large glycoprotein (gastric mucin) that protects the gastric mucosa from the acidic and proteolytically active chyme in the stomach lumen was upregulated. Thus, as gastric acid secretion is activated, probably in response to increased levels of the gastrin as well as luminal factors, there is an accompanying activation of the protective mucus layer that prevents auto-digestion of the gastric mucosa. It is also noteworthy that the genes for both gastrokine 1 and 2 were upregulated during digestion (Fig. 4). Gastrokines are constitutively produced proteins in the gastric mucosa in mammals and chickens, and while the physiological function remains somewhat elusive, they appear to upregulated during mucosal remodelling in response to inflammation (*e.g.* in connection with ulcers) and often downregulated in cancers. Thus, it is likely that the gastrokines are involved in regulating the restructuring of the mucosa during digestion in pythons.

In addition to analysing the gene expression profiles of the stomach, we also used a proteomics approach, assisted by our python transcriptome sequence database, to identify the hydrolytic enzymes in the gastric juice secreted during digestion. We identified python proteins on a complex background of highly abundant mice proteins. Python's digested food is, when it enters the duodenum, overall similar to digested food in e.g. humans. Thus, the digestive enzymes secreted by the pancreas are probably functional similar to known hydrolytic enzymes from other species. Consequently, the enzymes that facilitate the extreme digestion process and allow for have to be present in the stomach's digestive fluid.

We hypothesized that relative aggressive proteolytic digestive enzymes in the gastric juice facilitate digestion of large and un-masticated whole prey items [8]. In our analysis, six out of the seven identified proteolytic enzymes were pepsinogens homologous (Peptidase subfamily A1A), and these were also the most abundant hydrolytic enzymes in the gastric juice according to the emPAI values (Supplementary table S8). Most likely other pepsinogen isoforms exist in the gastric juice, as our approach predominantly target the most abundant proteolytic enzymes. The importance of the proteomics-identified pepsinogens was also substantiated by the transcriptomics data (Supplementary table S9). Here, we found that the six different pepsinogens were upregulated between 2.2 and 22.2 fold from the fasting animals to 48 hours after ingestion of mice. In average the pepsinogen transcripts were upregulated 10.7 fold. It supports that these proteases play a substantial role in the aggressive digestion process performed by the python.

Our proteomic analysis also suggested the identification of the pepsinogens as the major digestive proteolytic enzymes is similar to all other vertebrate species. Thus, our results indicate that it is not unique (with respect to protease class) and hitherto uncharacterized proteases that facilitate the aggressive digestion process. Instead, pepsins, homologous to pepsins among other species, digest the intact swallowed prey. The general condition in the stomach during digestion (e.g. pH) is also similar to other species. Thus, it is likely that these pepsins variants are among the most effective and aggressive pepsins identified so far and the provided sequence information facilitate future cloning, expression, and characterization of these potential industrial relevant enzymes.

Physiological interpretation of the upregulated genes in the intestine

The small intestine of pythons undergoes a remarkable and fast expansion during digestion where both wet and dry mass more than doubles within the first 24 hours. The expansion

stems primarily from increased mucosal mass, achieved by swelling of the individual enterocytes [44], while the smooth muscle in the gut wall is much less responsive [45]. Earlier studies on gene expression profiles during digestion in the python intestine revealed massive upregulation of more than one thousand genes, commencing within the first six hours after ingestion [12, 13]. Importantly, this previous study [13] identified a number of genes that are likely to be involved in the restructuring of the microvilli, cell division and apoptosis, as well as brush-border transporter proteins. In line with these earlier findings, our GO enrichment analysis also highlights functions pertaining to mitotic cell division, which supports a contribution to growth by hyperplasia faster cell turnover (Supplementary Fig. S5). The expansion of the individual enterocytes is accompanied by pronounced elongation of the microvilli [46] and the resulting rise in surface area of the intestinal lining is accompanied by an ten-fold increase in intestinal transport capacity for amino acids and other nutrients [1, 4, 47].

Earlier studies provided strong evidence for an upregulation of genes coding for nutrient transporter proteins, such as D-glucose, L-proline and L-leucine [13]. In this context, it is noteworthy that there were no nutrient transporters amongst the highly expressed and upregulated genes in the intestine (Fig. 5), but our KEGG analysis nevertheless showed increased expression of the serosal L-type amino acid transporter. Clearly, it would be worthwhile to quantitatively analyse the extent to which *de novo* synthesis of the various nutrient transporters, particularly those for amino acids, is increased during digestion and how much such synthesis contribute to absorptive capacity. It would seem adaptive if many of the transporters merely have to be activated, either by insertion within the luminal membrane or exposed as the enterocytes expand, to allow for an energetically cheap manner of matching intestinal performance to the sudden appearance of nutrients in the intestine after a meal. The GO enrichment analysis also pointed to an enrichment of various metabolic

processes during digestion, particularly for the upregulated genes (Supplementary Fig. S5). It is noteworthy that the expression of genes for glutathione S-transferase, peroxiredoxin and selenoprotein increased during digestion (Fig. 5). These three proteins are involved in cellular defence, particularly as antioxidants as a likely protection of reactive oxygen species resulting from increased aerobic metabolism.

There is consensus that the anatomical and structural responses underlying this phenotypic flexibility of intestinal function occur at modest energetic expenditure [17, 36, 48], but our expression profile does show increased expression of the gene coding for Cytochrome P450 pointing to increased aerobic and mitochondrial metabolism. An increased expression of genes involved in oxidative phosphorylation was also reported in earlier studies on pythons [12, 13]. This rise in metabolism may be driven primarily by the massive rise in secondary active transport to absorb the amino acids and smaller peptides rather than the structural changes [48]. Nevertheless, the structural changes may be reflected in increased expression of galectin 1 (Fig. 5), which mediate numerous function including cell–cell interactions, cell–matrix adhesion and transmembrane signalling.

Fig. 5 reveals the importance of lipid absorption and the subsequent transport by the cardiovascular and lymph systems, and it is also possible that several of the expressed proteins play a role in the incorporation of lipid droplets within the enterocytes. Thus, the presence of numerous apolipoproteins, and their precursor apoe protein, amongst the list of highly expressed and highly expressed genes (Fig. 5) are probably needed to transport the absorbed lipids in plasma and lymph, but the apolipoproteins could also act enzyme cofactors, receptor ligands, and lipid transfer carriers in the regulation of lipoprotein metabolism and cellular uptake. Diazepam-binding inhibitor (Fig. 5), a protein involved in lipid metabolism and under hormonal regulation mostly within nervous tissue, is also likely

to reflect the increased lipid absorption and metabolism in the postprandial period, and there was also a rise phospholipases (Fig. 5) that are likely to be involved in lipid degradation. Also, the capacity for protein metabolism clearly increased in the intestine during digestion (meprin A and endopeptidase that cleaves peptides, as well as 4-aminobutyrate aminotransferase, 4-trimethylaminobutyraldehyde dehydrogenase and diamine acetyltransferase) and there was a rise in the ammonium transporter protein Rh (Fig. 5). Finally, a number of proteins involved in calcium uptake and metabolism, such as calbindin and calmodulin (Fig. 5), could be important to handle the break-down of the bone in a normal rodent, and it was recently shown the enterocytes of pythons contain small particles of bone already 24 hours after ingestion [46].

Physiological interpretation of the upregulated genes in the heart

The large metabolic response to digestion is tailored by a doubling of heart rate and stroke of the heart such that cardiac output remains elevated for many days during digestion [49, 50]. This cardiovascular response plays a pivotal role in securing adequate oxygen delivery to the various organs and serves to ensure an appropriate convective transport of the nutrients taken up by the intestine. The tachycardia is mediated by a release of vagal tone and the presence of a non-adrenergic-non-cholinergic stimulation of the heart, which has been speculated to be released from the gastrointestinal organs during digestion [51, 52]. The increased heart rate, and the rise in the amount of blood pumped with each beat, must be supported by increased metabolism of the myocardium and we observed an upregulation of malate dehydrogenase, cytochromes and ATPase linked enzymes (Fig. 6) that are likely to be related to an increased oxidative phosphorylation within the individual myocytes (see also the prevalence of enriched GO terms associated with aerobic metabolism in Supplementary Fig. S8). Previous gene expression studies on the python heart also yielded evidence for increased oxidative

capacity in postprandial period [53] and cytochrome oxidase activity is almost doubled during digestion [54], and we confirm that transcription for heat shock proteins may be increased [53], possibly to protect against oxidative damage as result of the increased metabolism. As in earlier studies [53], our observation of increased ATP synthase lipid−binding protein and fatty acid binding protein 3 (Fig. 6) provide evidence for increased fatty acid metabolism, which may reflect the substantial rise in circulating fatty acids in the plasma.

It was originally suggested that the postprandial rise in stroke volume could be ascribed to an impressive and swift growth of the heart [10], possibly triggered lipid-signalling [53]. However, a number of recent studies, primarily from our laboratory, have shown that increased cardiac mass is not an obligatory postprandial response amongst pythons [54-56], and that stroke volume may be increased in response to increased venous return rather than cardiac hypertrophy [54]. It is nevertheless, noteworthy that our and the previous studies show a clear increase in the expression of contractile proteins (e.g. myosin and actin) as well as tubulin (Fig. 6), which may reflect increased protein-turnover in response to increased myocardial workload rather than cell proliferation or hypertrophy. The enriched GO analyses also point to major changes in the extracellular space as well as both elastin and collagen, which may indicate some level of cardiac reorganization at the cellular or subcellular level that may alter compliance of the myocardial wall and influence cardiac filling (Supplementary Fig. S8). It is noteworthy that the increased expression of BNP may serve a signalling function as described in response to the cardiac hypertrophy that attends hypertension.

Physiological interpretation of the genes in the liver

The liver exhibited a diverse expression profile in response to digestion that is likely to

reflect its many metabolic functions in connection with metabolism, synthesis and detoxification during the postprandial period. This pattern is also evident from the many metabolic functions identified in the enriched GO analysis (Supplementary Fig. S7). There were marked upregulations of the P450 system (Fig. 7), which stems well with a rise in synthesis and breakdown of hormones and signalling molecules, cholesterol synthesis in response to lipid absorption and possibly also an increased metabolism of potentially toxic compounds in the prey. A rise in cholesterol metabolism was supported by increased expression apolipoproteins (Fig. 7). The hepatic involvement in lipid metabolism was also supported by the increased expression of genes for Alpha-2-macroglobulin and serum albumin (Fig. 7). The increased expression of albumin obviously also fits nicely with the proteomic analysis of plasma proteins and it is likely that the postprandial rise in plasma albumin serves a functional role in the lipid transport between the intestine and the liver as well as other metabolically active organs

It is also noteworthy that a number of genes associated with the protection of oxidative stress, such as catalase, heat shock protein and glutathionine transferase were markedly upregulated (Fig. 7). It was recently argued that snakes digesting large meals experience oxidative damage due to reactive oxygen metabolites requiring increased antioxidant responses to protect cellular functions [57].

Physiological interpretation of the genes in the pancreas

We sampled the entire pancreas for our analysis of gene expression and our data therefore reflect both endocrine and exocrine pancreatic functions. The vast majority of the upregulated genes concerned the exocrine pancreas, and we found ample evidence for upregulated expression of genes associated with the digestive functions, such as lipases, trypsin, chymotrypsin and elastase and other enzymes for digestion of protein and lipid (Fig. 8). This

general upregulation of secretory processes is likely to explain the prevalence of processes associated with protein synthesis in the enriched GO analysis (Supplementary Fig. S6). There was even an increased expression of amylase (Fig. 8) that breaks down polysaccharides. In connection with this latter function, the increased expression of insulin (Fig. 8) from the endocrine pancreas is likely to reflect increased cellular signalling for postprandial uptake of both glucose and amino acids. As in the other organs, we found increased expression of cytochrome oxidase (Fig. 8) indicative of increased metabolism during digestion, and the rise in heat shock protein expression may reflect a response to formation of reactive oxygen-species as metabolism is stimulated by increased secretion of the pancreas.

Conclusions

Our study confirms that the extensive physiological and anatomical reorganization of the visceral organs of pythons during the postprandial period is driven by differential expression of hundreds or even thousands of genes. Many of the upregulated functions pertain to energy production to support the rise in aerobic metabolism associated with digestion and absorption of the large meals. In terms of the gastrointestinal organs, the gene expression profiles also support the view that many of the digestive functions, such as gastric acid secretion and nutrient absorption, can be stimulated with little gene expression indicating that the proteins involved in these processes are merely need to be activated during the postprandial period, and thus avoiding the energy and time-consuming processes associated with *de novo* synthesis. This digestive strategy may, at least in part, explain how intermittent feeders, such as snakes, retain the capacity for fast and reliable upregulation of the digestive processes immediately after ingestion.

Methods

Stimulation of the postprandial response, collection of tissue biopsies and purification of RNA for mRNA-seq analyses

Six *Python molurus* (Tiger Python/Burmese Python) with a body mass ranging from 180 to 700 g (average 373 g) were obtained from a commercial supplier and housed in vivaria with a heating system providing temperatures of 25-32 °C. The animals were fed rodents once a week and fresh water was always available. The animals appeared healthy and all experiments were performed according to Danish Federal Regulations. All six individuals were fasted for one month and divided in three groups. Four animals were fed a rodent meal of 25 % of body weight and euthanized with an intra-peritoneal injection of pentobarbital (50 mg kg^{-1}; Mebumal) at 24h (N = 2) or 48h after feeding (N = 2). The remaining two snakes served as fasted controls. During deep anaesthesia, two biopsies were obtained from each snake from each of the following tissues: The heart (ventricles), liver, stomach, intestine, and pancreas. In regard to the stomach tissue samples, one sample was obtained from the proximal part of the stomach and one sample was obtained from the distal part. In total, 60 biopsies were collected. The samples were taken from the same part of the different tissues in all individuals. After sampling, the biopsies were weighted and immediately snap frozen in liquid nitrogen; stomach and intestinal tissues were rinsed in sterile saline solution before weighting to avoid contamination with rodent tissue from the ingested meal. Subsequently, all 60 biopsies were homogenized in liquid nitrogen and the four biological replicates (two biopsies from each individual) were pooled in a 1:1 manner based on mass. It resulted in 15 samples (five tissues X three time points). From these samples, total RNA was purified using the Nucleospin RNA II kit (Machery-Nagel GmbH & Co.), as recommended by the manufacturer. The RNA concentration and quality were assessed by Nanodrop ND 1000

Spectrophotometer (Thermo Scientific) analyses, agarose gel-electrophoreses, and Agilent BioAnalyzer (Agilent) analyses.

Library production and sequencing

Poly-A transcripts were enriched and the transcripts broken in the presence of Zn^{2+}. Subsequently, double-stranded cDNA was synthesized using random primers and RNase H. After end repair and purification, the fragments were ligated with bar-coded paired-end adapters, and fragments with insert sizes of approximately 150-250 bp were isolated from an agarose gel. Each of the 15 samples derived from five tissues (heart, liver, stomach, pancreas and intestine) at the three time points (fasted for one month, 24h and 48h post-feeding) were amplified by PCR to generate DNA colonies template libraries and the libraries were then purified. In addition, to sample as broadly from transcriptome as possible, we also produced normalized libraries for each tissue in order to capture the reads from lowly expressed, tissue-specific genes. Here, a part of the samples, which originating from the same tissue, were pooled before the PCR analyses, i.e. in total five pooled samples were generated. These five samples were split in two and after PCR amplification and library purification they were normalized using two different normalization protocols, i.e. in total 10 normalized libraries were prepared. Library quality of all 25 samples was then assessed by a titration-run (1 x 50 bp) on an Illumina HiSeq 2000 instrument. Finally, the sequencing was performed on the same instrument using paired-reads (2 × 101 bp). One channel was used for the 15 non-normalized libraries and one channel was used for the 10 normalized libraries.

Data pre-processing and *de novo* transcriptome assembly

To reduce the amount of erroneous data, the raw paired reads were processed by i) removing reads that contained the sequencing adaptor, ii) removing reads that contained ambiguous

characters (Ns), and iii) trimming bases that had low average quality (Q<20) within a sliding window of length 10.

To develop a comprehensive transcriptomics resource for the Burmese python, all high-quality reads from 25 libraries were pooled together for *de novo* assembly. To determine the optimal assembly, *de novo* assembly was performed using Velvet（version 1.2.03) [18] and Oases (version 0.2.06) [58] with different k-mer parameters. The performance of these assemblies was assessed according to number of transcripts, total length of transcripts, N50 length, mean length, proportion of mapped reads and number of transcripts which length is larger than N50 (Supplementary Table S2).

Assessment of the transcriptome assembly

The transcriptome assembly was evaluated by rnaQUAST 1.4.0 with default parameters supplying reference genome sequences and genome annotation of Burmese python (GenBank assembly accession: GCA_000186305.2).

BUSCO_v2 [20] was used to test the completeness of transcriptome assembly with dependencies NCBI BLAST+ 2.4.0 [59] and HMMER 3.1b2 [60]. The vertebrata lineage set was used and accessed on 28 Nov 2016.

Transcriptome annotation

To assess the identity of the most closely related gene in other organisms, the assembled transcripts were compared with the sequences in the National Center for Biotechnology Information (NCBI) non-redundant protein (nr) database using blastx [61] with an e-value cut-off of 0.01. The nr annotation term of each transcript was assigned with the first best hit, which was not represented in uninformative description (e.g., 'hypothetical protein', 'novel protein', 'unnamed protein product', 'predicted protein' or 'Uncharacterized protein')

(Supplementary Table S4). To assign functional annotations of transcripts, Blast2GO was used (e-value threshold = 0.01) to return GO annotation, Enzyme code annotation with KEGG maps and InterPro annotation.

Estimation of gene expression values

For each 15 non-normalized libraries, the paired-end reads were firstly mapped back to assembled transcriptome using Bowtie2 [62]with default parameters, the raw counts then were calculated based on the alignment results using RSEM (version 1.1.20) [63] for each transcript. To quantify the gene expression level, for genes with alternative splicing transcripts, the longest transcript was selected to represent the gene, and a gene's abundance estimate was the sum of its transcripts' abundance estimates. Finally, the raw expression counts were normalized into FPKM with custom Perl scripts.

PCA

To facilitate graphical interpretation of tissue relatedness, R function prcomp was used to perform PCA with genes which the maximum FPKM of 15 samples was greater than 100.

Identification of DEGs and clustering analysis

For each tissue, DEGs were selected with two thresholds, 1) FPKM is greater than or equal to 400 in at least one time point and 2) fold change (FC) is greater than or equal to two in at least one pairwise comparison among three time points. FPKM values of DEGs were log2-transformed and median-centered, then hierarchical clustering was performed using R command hclust with method = 'average' and distance = 'Spearman correlation' and results were displayed using R command heatmap.2.

Colored KEGG Pathway and GO enrichment analysis

For each tissue, all assembled genes were mapped to KEGG human pathway maps using KOBAS 2.0 [64] with e-value 1e-50. Then genes were colored by representing FPKM value and trend of differential expression value (Table 2).

Blast2GO was used to implement GO enrichment analysis (Fisher's exact test) with threshold of FDR 0.001. The reference set is the whole transcripts with GO slim annotation. For each organ, the selected test set is either upregulated or highly expressed genes defined in Table 2. Finally, we performed Blast2GO to reduce to most specific GO terms.

Isolation of samples for proteomics analyses

Two Burmese pythons (weighing 400 and 800 g, respectively) were fed a rodent meal corresponding to approximately 25% of their body mass. Approximately 24 h into the postprandial period the animals were euthanized with an overdose of pentobarbital (100 mg kg^{-1}, i.m.). Immediately afterwards, an incision was made to expose the stomach, which was then ligated at the lower oesophagus and the pylorus, before the intact stomach was excised by a cleavage just below the two sutures resulting in the stomach being released from the rest of the animal. All undigested mouse remains were manually removed by forceps and 25 ml/kg tris-buffered saline (TBS) was injected into the stomach. The stomach was then ligated at the opened end, rinsed by gently shaking the tissue, and finally the digestive fluid-containing solution was collected and stored on ice. To ensure collection of all gastric fluid, the stomach was rinsed additional two-three times with 12 ml/kg TBS. Subsequently, the samples were filtered and centrifuged, and the supernatant stored at -80 °C. We also obtained two samples of gastric juice from a third individual (200 g) that had been fed 4 g peptone (Sigma Aldrich), suspended in water. Peptone is a mixture of small peptides and amino acids

and the solution was injected directly into the stomach and after three hours the snake was euthanized by an overdose of pentobarbital. The stomach was removed, rinsed with TBS, and a single sample collected and stored, as described above. We analysed two samples from each of the three individuals, resulting in a total of six digestive fluid samples being analysed by MS/MS. In addition, we obtained a single plasma sample from each snake by direct cardiac puncture followed by centrifugation and storage for later analysis.

Sample preparation for mass spectrometry analyses

The proteins in the six obtained python digestive fluid samples were recovered by trichloroacetic acid precipitation. The resulting pellets were resuspended in 8 M Urea, 5 mM DTT, 0.1 M ammonium bicarbonate pH 8.0 and incubated for 30 minutes at room temperature in order to denature and reduce the proteins. Subsequently, the proteins were alkylated by the addition of iodoacetamide to a final concentration of 25 mM. The samples were incubated for additional 20 minutes at room temperature and then diluted five times with a 50 mM ammonium bicarbonate, pH 8.0 buffer before the addition of approximately 2 μg sequencing grade modified trypsin (Promega) per 50 μg protein in the sample. Subsequently, the samples were incubated at 37 °C for approximately 16 h. The proteins in the plasma sample were denatured, reduced, alkylated, and digested with trypsin, as described for the digestive fluid samples. Finally, the resulting peptides in all samples were micropurified and stored at -20 C until the LC-MS/MS analyses.

Liquid chromatography-tandem mass spectrometry analyses

Nano-liquid chromatography-tandem mass spectrometry (LC–MS/MS) analyses were performed on a nanoflow HPLC system (Thermo Scientific, EASY-nLC II) connected to a mass spectrometer (TripleTOF 5600, AB Sciex) equipped with an electrospray ionization

source (NanoSpray III, AB Sciex) and operated under Analyst TF 1.6 control. The samples were dissolved in 0.1% formic acid, injected, trapped and desalted isocratically on a precolumn whereupon the peptides were eluted and separated on an analytical column (16 cm × 75 μm i.d.) packed in-house with ReproSil-Pur C18-AQ 3 μm resin (Dr. Marisch GmbH). The peptides were eluted at a flow rate of 250 nL/min using a 50 min gradient from 5 % to 35 % phase B (0.1 % formic acid and 90 % acetonitrile). An information dependent acquisition method was employed allowing up to 25 MS/MS spectra per cycle of 2.8 s.

Protein identification and filtering of data

The six collected MS files, related to digested fluid, were converted to Mascot generic format (MGF) using the AB SCIEX MS Data Converter beta 1.3 (AB SCIEX) and the “proteinpilot MGF” parameters. Subsequently, the files were merged to a single MGF-file using Mascot daemon. The resulting file (encompassing 122538 MS/MS queries) was used to interrogate the 16693 *Mus musculus* sequences in the Swiss-Prot database (version 2014_10) and the generated python database encompassing 21131 protein sequences using Mascot 2.5.0 (Matrix Science)[65]. Trypsin, with up to one missed cleavage allowed, was selected as enzyme; carbamidomethyl was employed as fixed modification, and oxidation of methionine and proline was selected as variable modifications. The instrument setting was specified as ESI-QUAD-TOF, the mass accuracy of the precursor and product ions was 15 ppm and 0.2 da respectively, and the significance threshold (p) was set to 0.01 and an expect cut-off at 0.005. The data obtained by the LC-MS/MS-analysis of the python plasma proteome was analysed as described for the digestive fluid samples, except that the *Mus musculus* sequences were not interrogated. This dataset contains 9224 MS/MS queries. All obtained results were subsequently parsed using MS Data Miner v. 1.3.0 [66], and protein hits were only accepted if they were identified based on two unique peptides. Semi-quantitative proteomics data was

obtained using the emPAI-values given by the Mascot 2.5.0 software after analysis of the MS/MS data [67].

To identify the proteins secreted into the python stomach, identified python plasma proteins, as well as the mouse protein homologs were removed from the list of identified python digestive fluid proteins. With regard to the removal of prey protein homologs, the overall mouse protein names were used to search the list of python proteins (e.g. “collagen” was used as search term, not “collagen alpha-1(I) chain”) and to identify python proteins that were identified based on homology with mouse. These proteins were removed from the list of stomach-secreted python proteins. For each identified protein remaining on the list, we reassessed the annotation of the python sequence, i.e. sequence comparisons were performed using blastp version 2.2.30, and in addition, UniProt and NCBI protein databases, as well as PubMed and SignalP 4.1, were interrogated to identify functional properties and cellular location of the identified proteins. Plasma proteins, remaining collagen homologous, intracellular proteins, and membrane proteins were discarded from the list of identified python stomach secretome proteins.

List of abbreviations

DEG differentially expressed genes

FC fold change

FPKM fragments per kilo base per million sequenced reads

PCA principal component analysis

Declarations

Ethics approval and consent to participate

Not applicable

Consent for publication

Not applicable

Availability of data and material

The raw RNA-Seq sequencing data that support the findings of this study have been deposited in the NCBI BioProject database (accession no. PRJNA343735). https://www.ncbi.nlm.nih.gov/bioproject/PRJNA343735

Competing interests

The authors declare that they have no competing interests.

Funding

This work was supported by a grant (Novenia) from the Danish Research Council for Strategic Research (grant identification number: 09-067076).

Authors' contributions

JD, KWS, TW and MHS designed the study. JD performed the transcriptome data analysis with input from LS and was a major contributor in writing the manuscript. SEL performed RNA-Seq lab experiment. KWS and JE performed the proteomics experiment and data analysis. WT interpreted the transcriptome data regarding digestion. All authors read and approved the final manuscript.

Acknowledgements

We thank Tania A. Nielsen (Aarhus, Denmark) for valuable assistance with RNA purification and Fasteris SA (Switzerland) for library preparation and Illumina sequencing.

Figure and table legends

Fig. 1. The workflow of Python RNA-Seq data analysis. The diagram shows the main steps and bioinformatics tools used in the study.

Fig. 2. PCA plots of FPKM of 1862 genes. PC, principal component. PC1 represents 25%, PC2 represents 18% and PC3 represents 16% of total variation in the data. The name of the label consists two part, one capital letter plus one number. Letter H, S, I, L, P represent heart, stomach, intestine, liver and pancreas respectively. Number 0, 1, 2 represent fasting for one month, 24h/1d after feeding and 48h/2d after feeding respectively.

Fig. 3. Heat maps from hierarchical clustering of DEGs in each tissue. Heat maps showing the hierarchically clustered Spearman correlation matrix resulting from comparing the normalized FPKM value for each pair of genes. Heat map columns represent samples and rows correspond to genes. Expression values (FPKM) are log_2-transformed and then median-centered by gene. Relative levels of gene expression are represented by colors. Pale colour is low expression and darker blue is high expression. Five sub-clusters labelled a to e are shown with full annotation in Fig. 4-8.

Fig. 4. The cluster of upregulated genes with NCBI nr annotation in stomach. It shows the cluster e in Fig. 3. Heat map columns represent samples and rows correspond to genes. Expression values (FPKM) are log_2-transformed and then median-centered by gene. Relative levels of gene expression are represented by colors. Pale colour is low expression and darker blue is high expression.

Fig. 5. The cluster of upregulated genes with NCBI nr annotation in intestine. It shows the cluster b in Fig. 3. Heat map columns represent samples and rows correspond to genes. Expression values (FPKM) are log_2-transformed and then median-centered by gene. Relative levels of gene expression are represented by colors. Pale colour is low expression and darker blue is high expression.

Fig. 6. The cluster of upregulated genes with NCBI nr annotation in heart. It shows the cluster a in Fig. 3. Heat map columns represent samples and rows correspond to genes.

Expression values (FPKM) are log_2-transformed and then median-centered by gene. Relative levels of gene expression are represented by colors. Pale colour is low expression and darker blue is high expression.

Fig. 7. The cluster of upregulated genes with NCBI nr annotation in liver. It shows the cluster c in Fig. 3. Heat map columns represent samples and rows correspond to genes. Expression values (FPKM) are log_2-transformed and then median-centered by gene. Relative levels of gene expression are represented by colors. Pale colour is low expression and darker blue is high expression.

Fig. 8. The cluster of upregulated genes with NCBI nr annotation in pancreas. It shows the cluster d in Fig. 3. Heat map columns represent samples and rows correspond to genes. Expression values (FPKM) are log_2-transformed and then median-centered by gene. Relative levels of gene expression are represented by colors. Pale colour is low expression and darker blue is high expression.

Fig. 9. The workflow used to identify the python's stomach secretome during digestion. 1) Initially pythons were feed with mice, or a peptide mixture, and later the gastric juice samples were obtained and mice debris were removed. **2)** The proteins were precipitated, denatured and digested with trypsin. **3)** The resulting tryptic peptides were analysed by LC-MS/MS analyses and the data merged into a single file. **4)** The file was used to interrogate the in-house generated python protein sequence database (based on the transcriptomic data) and python proteins were identified. **5)** The data was filtered to remove mice proteins and plasma proteins. Subsequently, the annotation of the remaining proteins was reassessed and the secretome identified.

Fig. 10. Cartoon depiction of colored KEGG pathway of gastric acid secretion in stomach. Entry in red represents upregulated during digestion; Entry in purple for highly expressed. H/K is H+/K+-exchanging ATPase alpha polypeptide. CA is carbonic anhydrase. AE is solute carrier family 26 (anion exchange transporter).

Table 1. Summary of transcriptome assembly of Burmese Python.

Table 2. Colour coding of genes in KEGG pathway maps. Three criteria are used to classify and colour genes. First, i) whether the maximum FPKM of the gene among fasting, 24h and 48h is over 10, then ii) whether the gene is differential expressed in at least one of the pairwise comparison among fasting, 24h and 48h with FC over 4. Finally, iii) for those genes expressed, but not differential expressed, whether it is highly expressed with maximum FPKM among three time points over 200. The term expression trend indicates the trend of gene expression across fasting, 24h and 48h. e.g. The trend up means the gene is upregulated from either fasting to 24h, fasting to 48h or 24h to 48h. The trend up-then-down means the gene is firstly upregulated from fasting to 24h, then downregulated from 24h to 48h.

Table 3. The number of DEGs across fasting, 24h and 48h in each tissue. The expression trend is consistent with definition in Table 2.

Reference

1. Secor SM, Diamond J: **A vertebrate model of extreme physiological regulation.** *Nature* 1998, **395:**659-662.
2. Cox CL, Secor SM: **Matched regulation of gastrointestinal performance in the Burmese python, Python molurus.** *J Exp Biol* 2008, **211:**1131-1140.
3. Secor SM, Diamond J: **Adaptive responses to feeding in Burmese pythons: pay before pumping.** *J Exp Biol* 1995, **198:**1313-1325.
4. Secor SM, Diamond JM: **Evolution of regulatory responses to feeding in snakes.** *Physiological and Biochemical Zoology* 2000, **73:**123-141.
5. Aubret F, Shine R, Bonnet X: **Evolutionary biology: adaptive developmental plasticity in snakes.** *Nature* 2004, **431:**261-262.
6. Cohn MJ, Tickle C: **Developmental basis of limblessness and axial patterning in snakes.** *Nature* 1999, **399:**474-479.
7. Ott BD, Secor SM: **Adaptive regulation of digestive performance in the genus Python.** *J Exp Biol* 2007, **210:**340-356.
8. Secor SM: **Digestive physiology of the Burmese python: broad regulation of integrated performance.** *J Exp Biol* 2008, **211:**3767-3774.
9. Zaar M, Overgaard J, Gesser H, Wang T: **Contractile properties of the functionally divided python heart: two sides of the same matter.** *Comp Biochem Physiol A Mol Integr Physiol* 2007, **146:**163-173.
10. Andersen JB, Rourke BC, Caiozzo VJ, Bennett AF, Hicks JW: **Physiology: postprandial cardiac hypertrophy in pythons.** *Nature* 2005, **434:**37-38.
11. Vidal N, Hedges SB: **Molecular evidence for a terrestrial origin of snakes.** *Proc Biol Sci* 2004, **271 Suppl 4:**S226-229.
12. Castoe TA, de Koning AP, Hall KT, Card DC, Schield DR, Fujita MK, Ruggiero RP, Degner JF, Daza JM, Gu W, et al: **The Burmese python genome reveals the molecular basis for extreme adaptation in snakes.** *Proc Natl Acad Sci U S A* 2013, **110:**20645-20650.
13. Andrew AL, Card DC, Ruggiero RP, Schield DR, Adams RH, Pollock DD, Secor SM, Castoe TA: **Rapid changes in gene expression direct rapid shifts in intestinal form and function in the Burmese python after feeding.** *Physiol Genomics* 2015, **47:**147-157.
14. Castoe TA, Fox SE, Jason de Koning A, Poole AW, Daza JM, Smith EN, Mockler TC, Secor SM, Pollock DD: **A multi-organ transcriptome resource for the Burmese Python (Python molurus bivittatus).** *BMC Res Notes* 2011, **4:**310.
15. Wang Z, Gerstein M, Snyder M: **RNA-Seq: a revolutionary tool for transcriptomics.** *Nat Rev Genet* 2009, **10:**57-63.
16. Mortazavi A, Williams BA, McCue K, Schaeffer L, Wold B: **Mapping and quantifying mammalian transcriptomes by RNA-Seq.** *Nat Methods* 2008, **5:**621-628.
17. Wang T, Hung CC, Randall DJ: **The comparative physiology of food deprivation: from feast to famine.** *Annu Rev Physiol* 2006, **68:**223-251.
18. Zerbino DR, Birney E: **Velvet: algorithms for de novo short read assembly using de Bruijn graphs.** *Genome Res* 2008, **18:**821-829.
19. Bushmanova E, Antipov D, Lapidus A, Suvorov V, Prjibelski AD: **rnaQUAST: a quality assessment tool for de novo transcriptome assemblies.** *Bioinformatics* 2016, **32:**2210-2212.

20. Simão FA, Waterhouse RM, Ioannidis P, Kriventseva EV, Zdobnov EM: **BUSCO: assessing genome assembly and annotation completeness with single-copy orthologs.** *Bioinformatics* 2015:btv351.
21. Pruitt KD, Tatusova T, Maglott DR: **NCBI reference sequences (RefSeq): a curated non-redundant sequence database of genomes, transcripts and proteins.** *Nucleic Acids Res* 2007, **35:**D61-65.
22. Benton MJ: **Phylogeny of the major tetrapod groups: morphological data and divergence dates.** *J Mol Evol* 1990, **30:**409-424.
23. Conesa A, Gotz S, Garcia-Gomez JM, Terol J, Talon M, Robles M: **Blast2GO: a universal tool for annotation, visualization and analysis in functional genomics research.** *Bioinformatics* 2005, **21:**3674-3676.
24. Hunter S, Apweiler R, Attwood TK, Bairoch A, Bateman A, Binns D, Bork P, Das U, Daugherty L, Duquenne L, et al: **InterPro: the integrative protein signature database.** *Nucleic Acids Res* 2009, **37:**D211-215.
25. Trapnell C, Williams BA, Pertea G, Mortazavi A, Kwan G, van Baren MJ, Salzberg SL, Wold BJ, Pachter L: **Transcript assembly and quantification by RNA-Seq reveals unannotated transcripts and isoform switching during cell differentiation.** *Nat Biotechnol* 2010, **28:**511-515.
26. Fan HP, Di Liao C, Fu BY, Lam LC, Tang NL: **Interindividual and interethnic variation in genomewide gene expression: insights into the biological variation of gene expression and clinical implications.** *Clin Chem* 2009, **55:**774-785.
27. Kanehisa M, Goto S, Sato Y, Furumichi M, Tanabe M: **KEGG for integration and interpretation of large-scale molecular data sets.** *Nucleic Acids Res* 2012, **40:**D109-114.
28. Kanehisa M, Goto S: **KEGG: kyoto encyclopedia of genes and genomes.** *Nucleic Acids Res* 2000, **28:**27-30.
29. Kageyama T: **Pepsinogens, progastricsins, and prochymosins: structure, function, evolution, and development.** *Cell Mol Life Sci* 2002, **59:**288-306.
30. Hayashi K, Agata K, Mochii M, Yasugi S, Eguchi G, Mizuno T: **Molecular cloning and the nucleotide sequence of cDNA for embryonic chicken pepsinogen: phylogenetic relationship with prochymosin.** *J Biochem* 1988, **103:**290-296.
31. Watanuki K, Yasugi S: **Analysis of transcription regulatory regions of embryonic chicken pepsinogen (ECPg) gene.** *Dev Dyn* 2003, **228:**51-58.
32. Aoki N, Deshimaru M, Terada S: **Active fragments of the antihemorrhagic protein HSF from serum of habu (Trimeresurus flavoviridis).** *Toxicon* 2007, **49:**653-662.
33. Perales J, Neves-Ferreira AG, Valente RH, Domont GB: **Natural inhibitors of snake venom hemorrhagic metalloproteinases.** *Toxicon* 2005, **45:**1013-1020.
34. Tomihara Y, Yonaha K, Nozaki M, Yamakawa M, Kawamura Y, Kamura T, Toyama S: **Purification of an antihemorrhagic factor from the serum of the non-venomous snake Dinodon semicarinatus.** *Toxicon* 1988, **26:**420-423.
35. Lee HJ, Sorimachi H, Jeong SY, Ishiura S, Suzuki K: **Molecular cloning and characterization of a novel tissue-specific calpain predominantly expressed in the digestive tract.** *Biol Chem* 1998, **379:**175-183.
36. Secor SM: **Gastric function and its contribution to the postprandial metabolic response of the Burmese python Python molurus.** *Journal of Experimental Biology* 2003, **206:**1621-1630.

37. Bessler SM, Secor SM: **Effects of feeding on luminal pH and morphology of the gastroesophageal junction of snakes.** *Zoology (Jena)* 2012, **115:**319-329.
38. Helmstetter C, Pope RK, T'Flachebba M, Secor SM, Lignot J-H: **The effects of feeding on cell morphology and proliferation of the gastrointestinal tract of juvenile Burmese pythons (Python molurus).** *Canadian Journal of Zoology* 2009, **87:**1255-1267.
39. Enok S, Simonsen LS, Wang T: **The contribution of gastric digestion and ingestion of amino acids on the postprandial rise in oxygen consumption, heart rate and growth of visceral organs in pythons.** *Comp Biochem Physiol A Mol Integr Physiol* 2013, **165:**46-53.
40. Nørgaard S, Andreassen K, Malte CL, Enok S, Wang T: **Low cost of gastric acid secretion during digestion in ball pythons.** *Comparative Biochemistry and Physiology Part A: Molecular & Integrative Physiology* 2016, **194:**62-66.
41. Secor SM, Taylor JR, Grosell M: **Selected regulation of gastrointestinal acid-base secretion and tissue metabolism for the diamondback water snake and Burmese python.** *J Exp Biol* 2012, **215:**185-196.
42. Andrade DV, De Toledo LF, Abe AS, Wang T: **Ventilatory compensation of the alkaline tide during digestion in the snake Boa constrictor.** *J Exp Biol* 2004, **207:**1379-1385.
43. Koelz HR: **Gastric acid in vertebrates.** *Scand J Gastroenterol Suppl* 1992, **193:**2-6.
44. Starck JM, Beese K: **Structural flexibility of the intestine of Burmese python in response to feeding.** *Journal of Experimental Biology* 2001, **204:**325-335.
45. Holmberg A, Kaim J, Persson A, Jensen J, Wang T, Holmgren S: **Effects of digestive status on the reptilian gut.** *Comparative Biochemistry and Physiology a-Molecular and Integrative Physiology* 2002, **133:**499-518.
46. Lignot JH, Helmstetter C, Secor SM: **Postprandial morphological response of the intestinal epithelium of the Burmese python (Python molurus).** *Comp Biochem Physiol A Mol Integr Physiol* 2005, **141:**280-291.
47. Secor SM, Whang EE, Lane JS, Ashley SW, Diamond J: **Luminal and systemic signals trigger intestinal adaptation in the juvenile python.** *American Journal of Physiology-Gastrointestinal and Liver Physiology* 2000, **279:**G1177-G1187.
48. Overgaard J, Andersen JB, Wang T: **The effects of fasting duration on the metabolic response to feeding in Python molurus: an evaluation of the energetic costs associated with gastrointestinal growth and upregulation.** *Physiol Biochem Zool* 2002, **75:**360-368.
49. Secor SM, White SE: **Prioritizing blood flow: cardiovascular performance in response to the competing demands of locomotion and digestion for the Burmese python, Python molurus.** *J Exp Biol* 2010, **213:**78-88.
50. Secor SM, Hicks JW, Bennett AF: **Ventilatory and cardiovascular responses of a python (Python molurus) to exercise and digestion.** *J Exp Biol* 2000, **203:**2447-2454.
51. Skovgaard N, Møller K, Gesser H, Wang T: **Histamine induces postprandial tachycardia through a direct effect on cardiac H2-receptors in pythons.** *American Journal of Physiology - Regulatory, Integrative and Comparative Physiology* 2009, **296:**R774-R785.
52. Enok S, Simonsen LS, Pedersen SV, Wang T, Skovgaard N: **Humoral regulation of heart rate during digestion in pythons (Python molurus and Python regius).** *Am J Physiol Regul Integr Comp Physiol* 2012, **302:**R1176-1183.

53. Riquelme CA, Magida JA, Harrison BC, Wall CE, Marr TG, Secor SM, Leinwand LA: **Fatty acids identified in the Burmese python promote beneficial cardiac growth.** *Science* 2011, **334:**528-531.

54. Enok S, Leite G, Leite C, Gesser H, Hedrick MS, Wang T: **Improved cardiac filling facilitates the postprandial elevation of stroke volume in Python regius.** *J Exp Biol* 2016.

55. Slay CE, Enok S, Hicks JW, Wang T: **Reduction of blood oxygen levels enhances postprandial cardiac hypertrophy in Burmese python (Python bivittatus).** *J Exp Biol* 2014, **217:**1784-1789.

56. Jensen B, Larsen CK, Nielsen JM, Simonsen LS, Wang T: **Change of cardiac function, but not form, in postprandial pythons.** *Comp Biochem Physiol A Mol Integr Physiol* 2011, **160:**35-42.

57. Butler MW, Lutz TJ, Fokidis HB, Stahlschmidt ZR: **Eating increases oxidative damage in a reptile.** *J Exp Biol* 2016, **219:**1969-1973.

58. Schulz MH, Zerbino DR, Vingron M, Birney E: **Oases: robust de novo RNA-seq assembly across the dynamic range of expression levels.** *Bioinformatics* 2012, **28:**1086-1092.

59. Camacho C, Coulouris G, Avagyan V, Ma N, Papadopoulos J, Bealer K, Madden TL: **BLAST+: architecture and applications.** *BMC Bioinformatics* 2009, **10:**421.

60. Eddy SR: **A probabilistic model of local sequence alignment that simplifies statistical significance estimation.** *PLoS Comput Biol* 2008, **4:**e1000069.

61. Altschul SF, Gish W, Miller W, Myers EW, Lipman DJ: **Basic local alignment search tool.** *J Mol Biol* 1990, **215:**403-410.

62. Langmead B, Salzberg SL: **Fast gapped-read alignment with Bowtie 2.** *Nat Methods* 2012, **9:**357-359.

63. Li B, Dewey CN: **RSEM: accurate transcript quantification from RNA-Seq data with or without a reference genome.** *BMC Bioinformatics* 2011, **12:**323.

64. Xie C, Mao X, Huang J, Ding Y, Wu J, Dong S, Kong L, Gao G, Li CY, Wei L: **KOBAS 2.0: a web server for annotation and identification of enriched pathways and diseases.** *Nucleic Acids Res* 2011, **39:**W316-322.

65. Perkins DN, Pappin DJC, Creasy DM, Cottrell JS: **Probability-based protein identification by searching sequence databases using mass spectrometry data.** *Electrophoresis* 1999, **20:**3551-3567.

66. Dyrlund TF, Poulsen ET, Scavenius C, Sanggaard KW, Enghild JJ: **MS Data Miner: A web-based software tool to analyze, compare, and share mass spectrometry protein identifications.** *Proteomics* 2012, **12:**2792-2796.

67. Ishihama Y, Oda Y, Tabata T, Sato T, Nagasu T, Rappsilber J, Mann M: **Exponentially modified protein abundance index (emPAI) for estimation of absolute protein amount in proteomics by the number of sequenced peptides per protein.** *Mol Cell Proteomics* 2005, **4:**1265-1272.

Table 1

Click here to download Table Table1.xlsx

**Parameter**	***De novo* assembly**
Total transcripts	34,423
Annotated transcripts with nr NCBI	19,713
Annotated transcripts with GO term	16,992
Minimum transcript size (nt)	100
Medium transcrpt size (nt)	605
Mean transcript size (nt)	1,034
Largest transcript (nt)	26,010
N50	6,240
N50 size (nt)	1,673
Total assembled bases (Mb)	35.6

Table 2

Click here to download Table Table2.xlsx

Expression level	Fold change level	Expression trend (fasting -> 24h -> 48h)	Color code
max FPKM over 10	FC over 4	Up-regulated	Red
		Down-regulated	Blue
		Up-then-down regulated	Yellow
		Down-then-up regulated	Brown
	FC below 4	Highly expressed (max FPKM over 200)	Purple
		Moderately expressed (max FPKM below 200)	Pink
max FPKM below 10	-	Lowly expressed	Darkgrey

Table 3 Click here to download Table Table3.xlsx

Expression trend (fasting -> 24h -> 48h)	Stomach	Intestine	Pancreas	Liver	Heart
Up-regulated	932 (2.9%)	1,131 (3.5%)	859 (2.6%)	1,047 (3.2%)	184 (0.6%)
Up-then-down regulated	28 (0.1%)	31 (0.1%)	150 (0.5%)	61 (0.2%)	6 (0.0%)
Down-regulated	869 (2.7%)	625 (1.9%)	567 (1.7%)	618 (1.9%)	168 (0.5%)
Down-then-up regulated	36 (0.1%)	45 (0.1%)	127 (0.4%)	90 (0.3%)	16 (0.1%)
Highly expressed	199 (0.6%)	211 (0.7%)	225 (0.7%)	354 (1.1%)	232 (0.7%)
Moderately expressed	5,541 (17.0%)	5,582 (17.2%)	4,933 (15.2%)	5,385 (16.5%)	6,044 (18.6%)
Lowly expressed	24,926 (76.6%)	24,906 (76.5%)	25,670 (78.9%)	24,976 (76.8%)	25,881 (79.5%)
**Total**	32,531 (100%)	32,531 (100%)	32,531 (100%)	32,531 (100%)	32,531 (100%)

Figure 1

Click here to download Figure Figure1.pdf

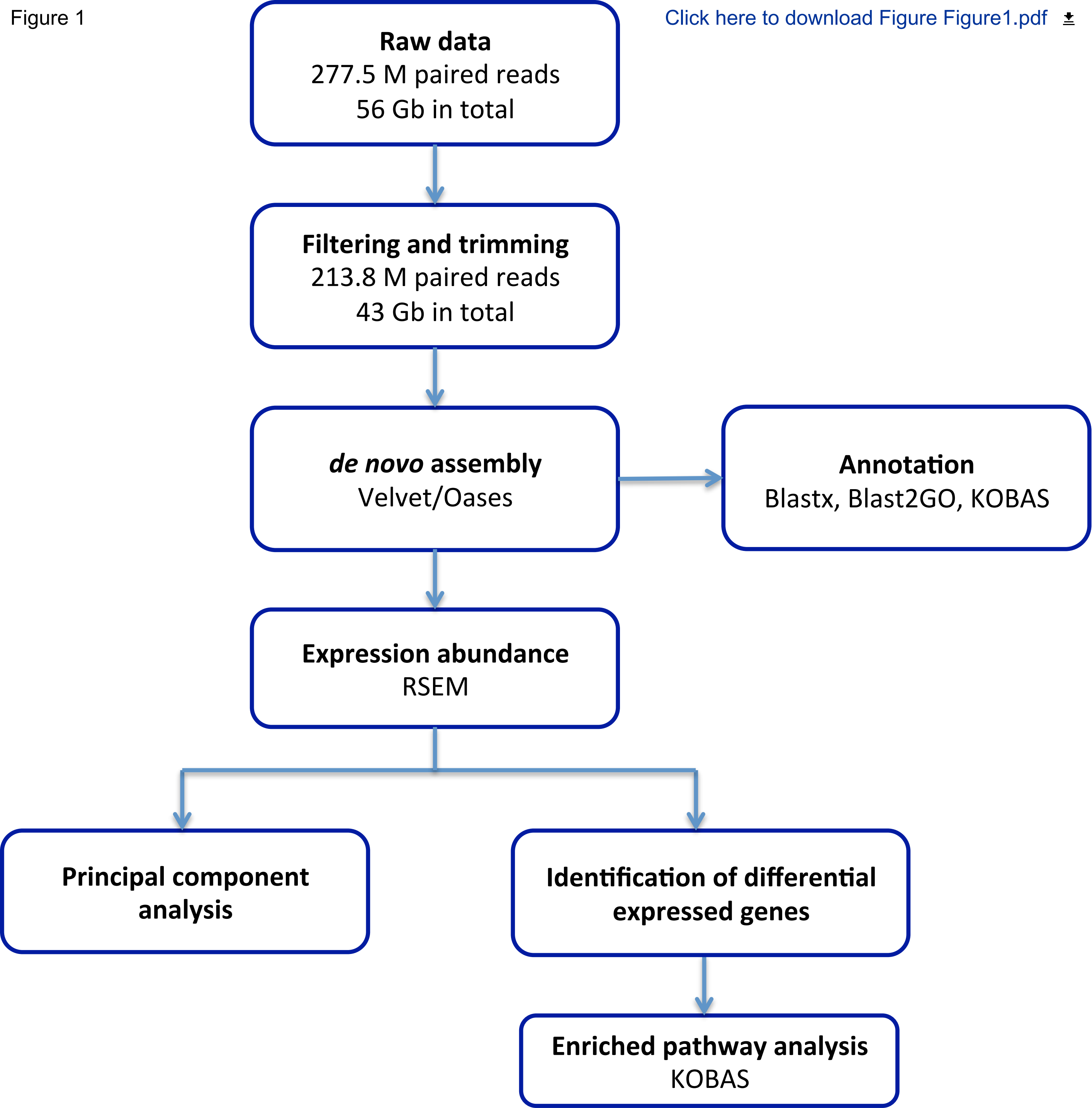

Figure 2

Click here to download Figure Figure2.pdf

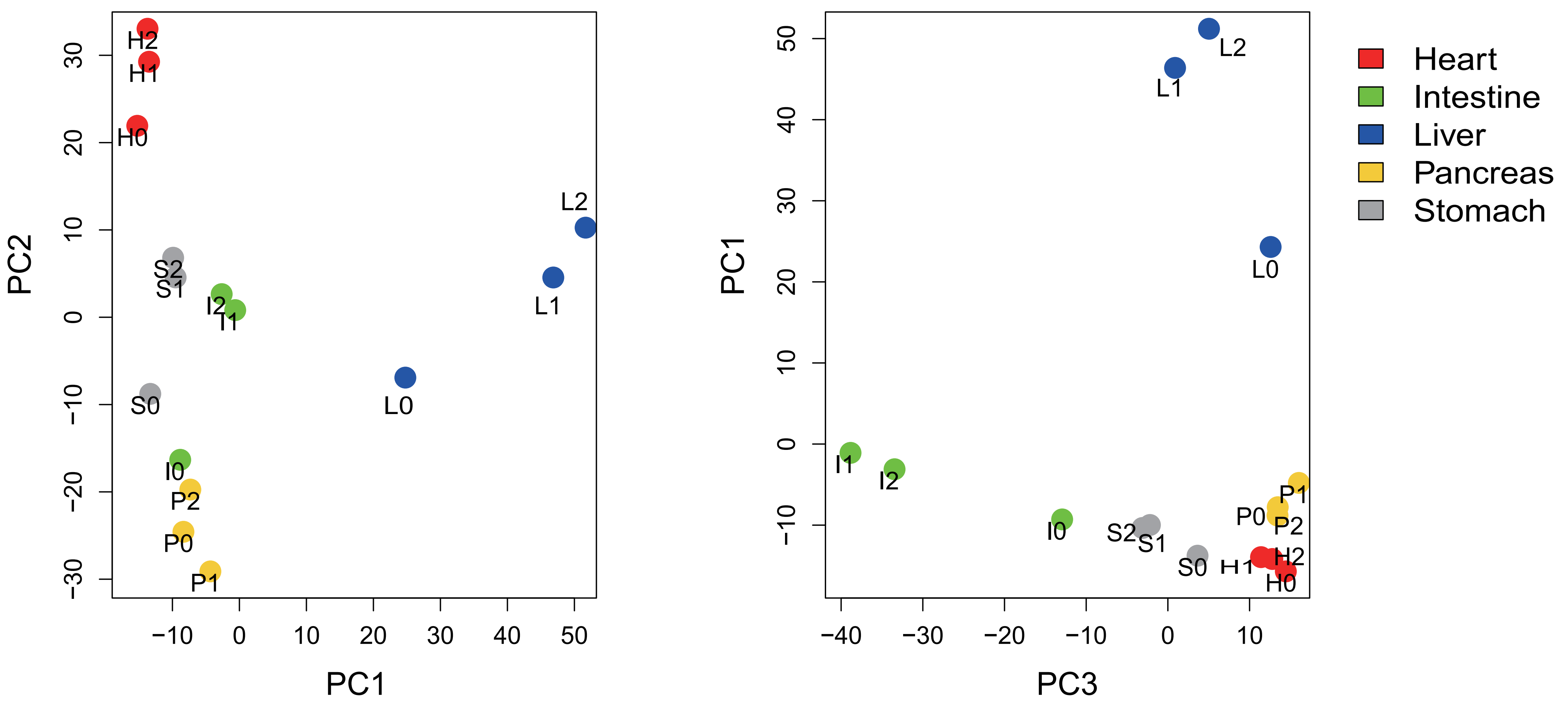

Figure 3

Click here to download Figure Figure3.pdf

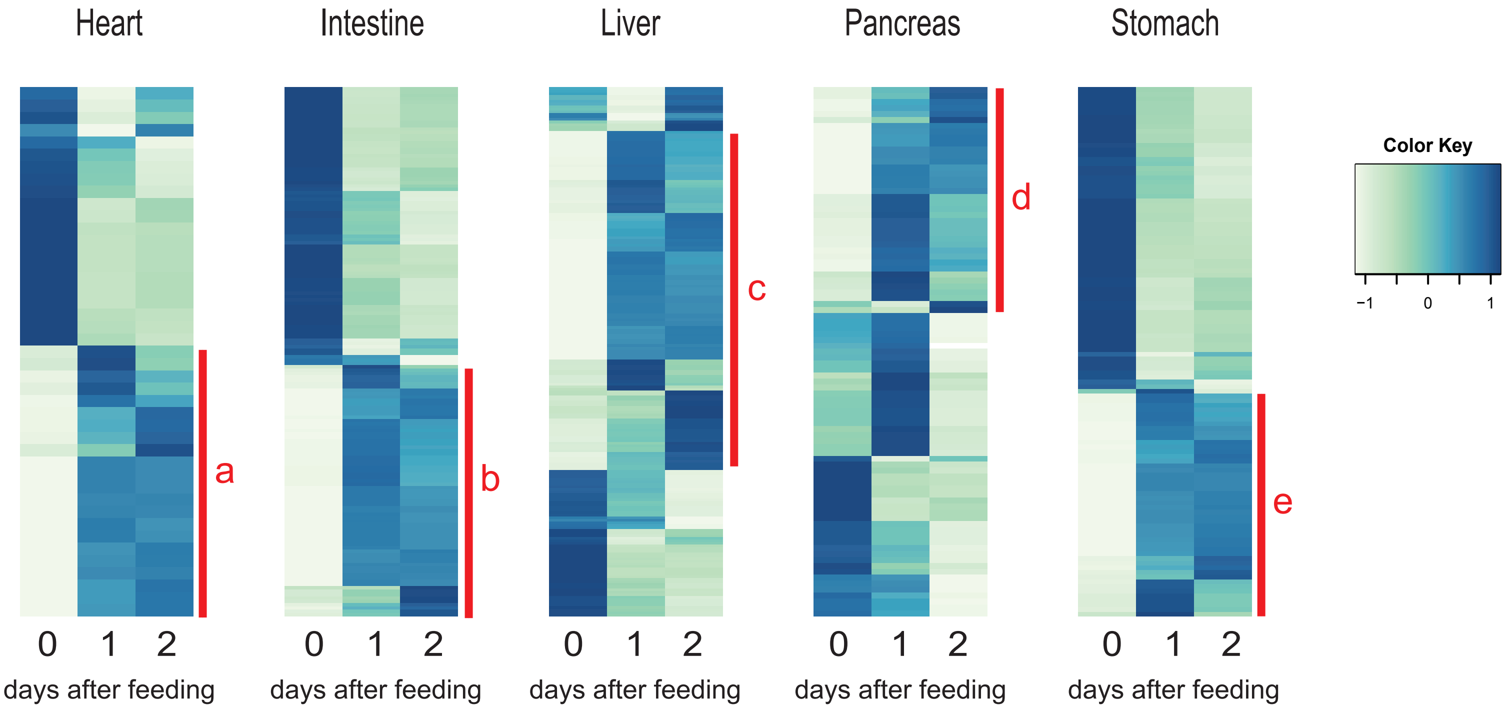

Figure 4

Click here to download Figure Figure4.pdf

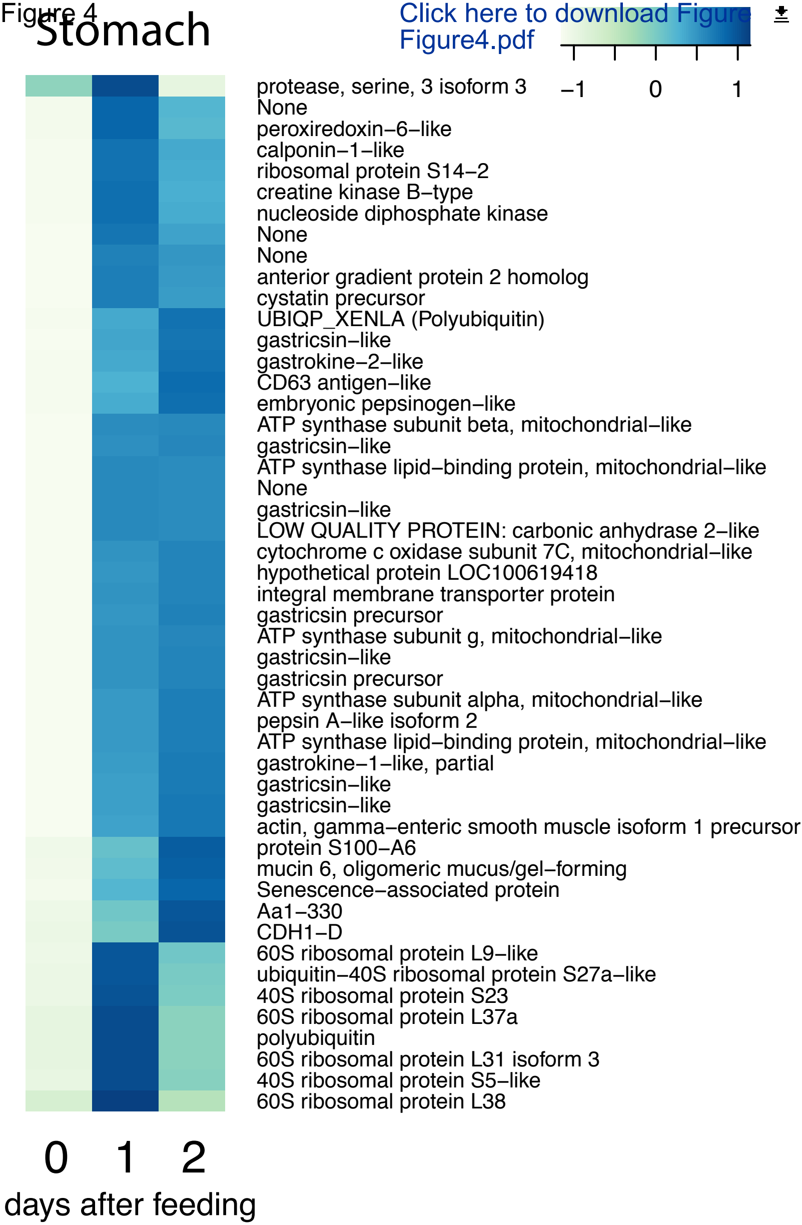

Figure 5

Click here to download Figure Figure5.pdf

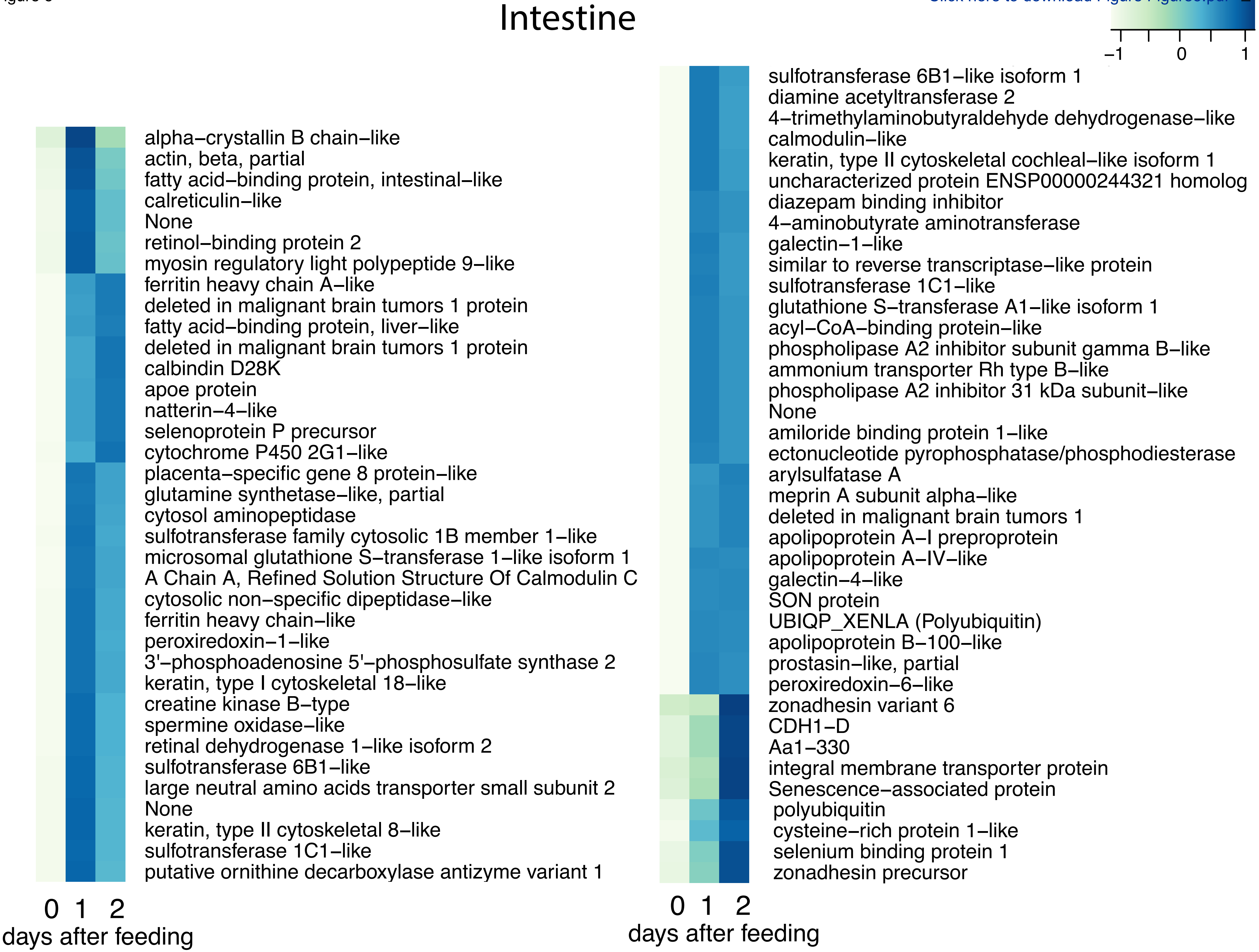

Figure 6

Click here to download Figure Figure6.pdf

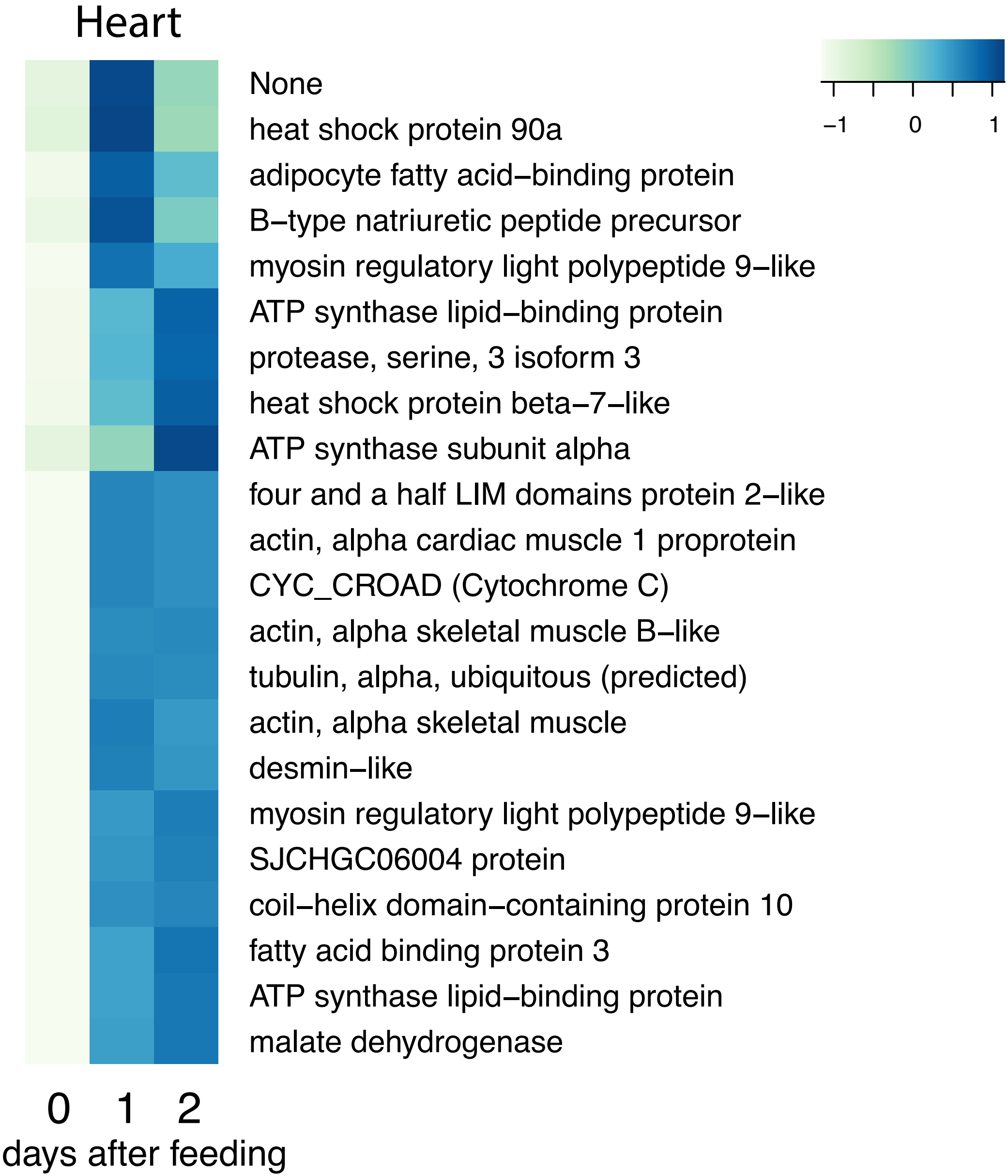

Figure 7

Click here to download Figure Figure7.pdf

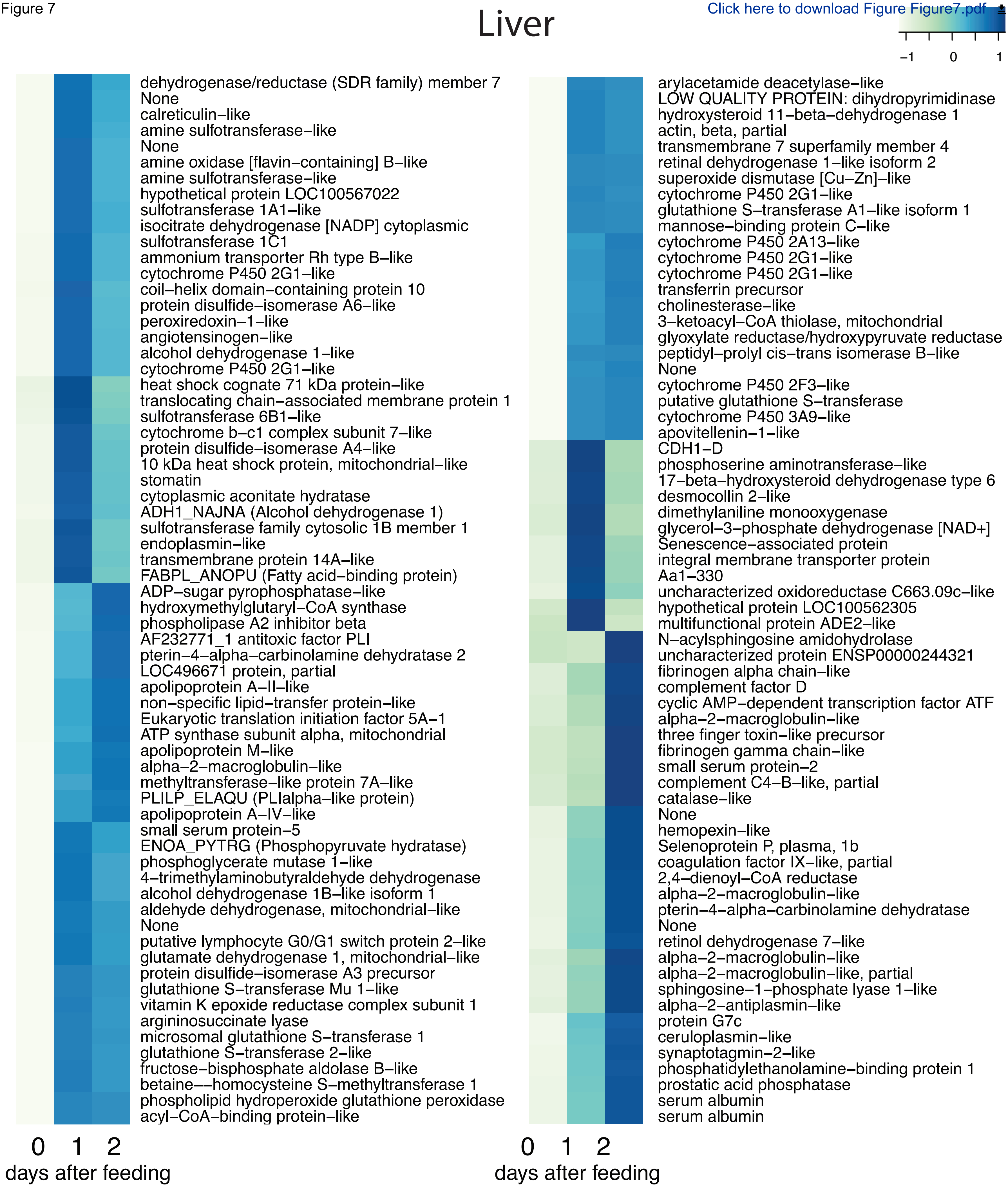

Figure 8

Click here to download Figure Figure8.pdf

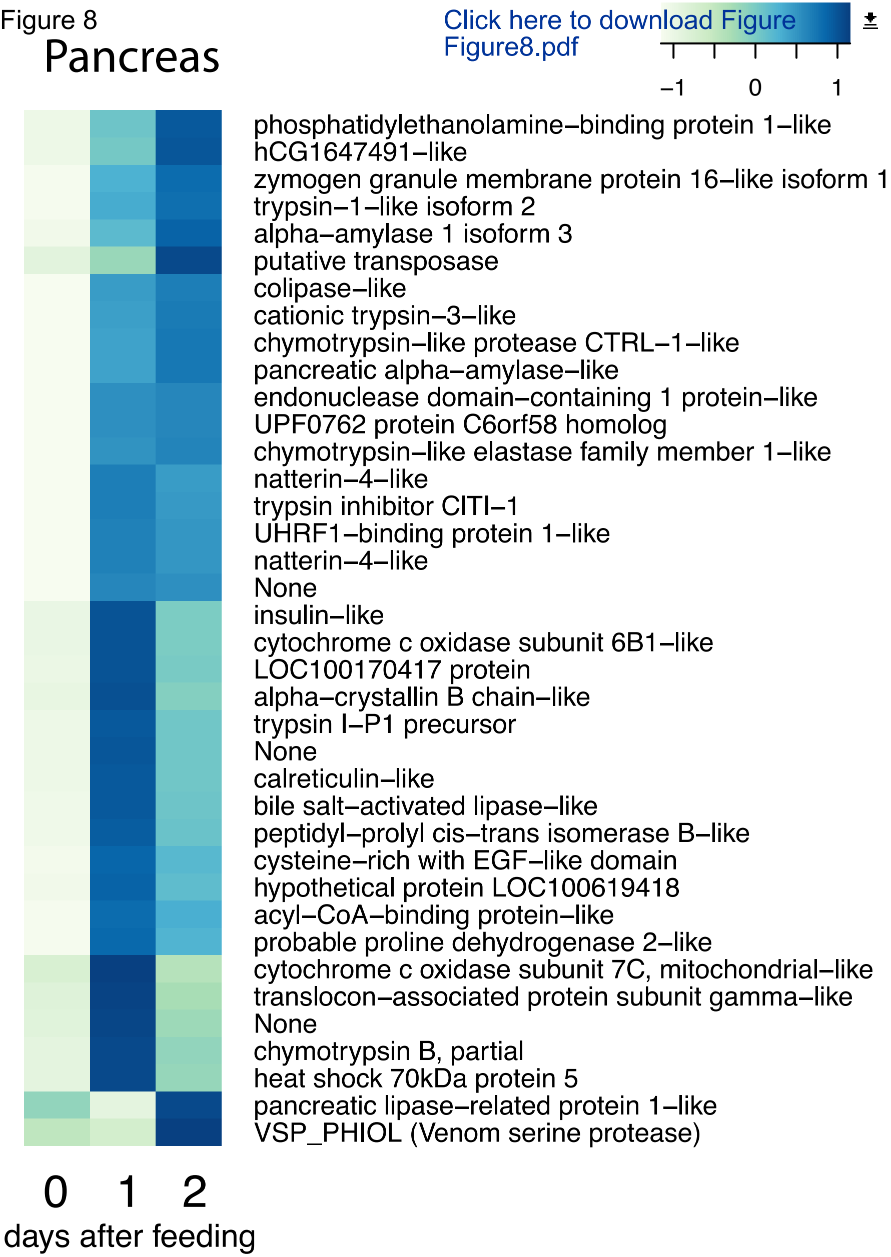

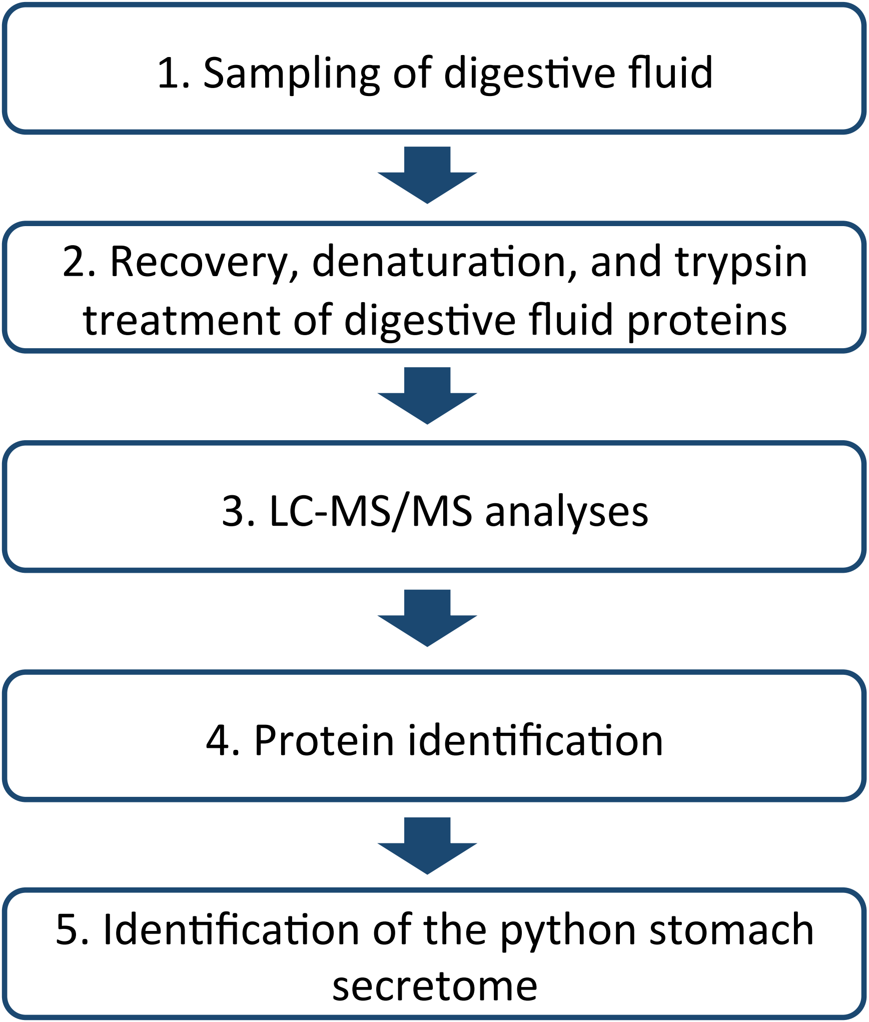
1. Sampling of digestive fluid
2. Recovery, denaturation, and trypsin treatment of digestive fluid proteins
3. LC-MS/MS analyses
4. Protein identification
5. Identification of the python stomach secretome

Figure 10

Click here to download Figure Figure10.pdf

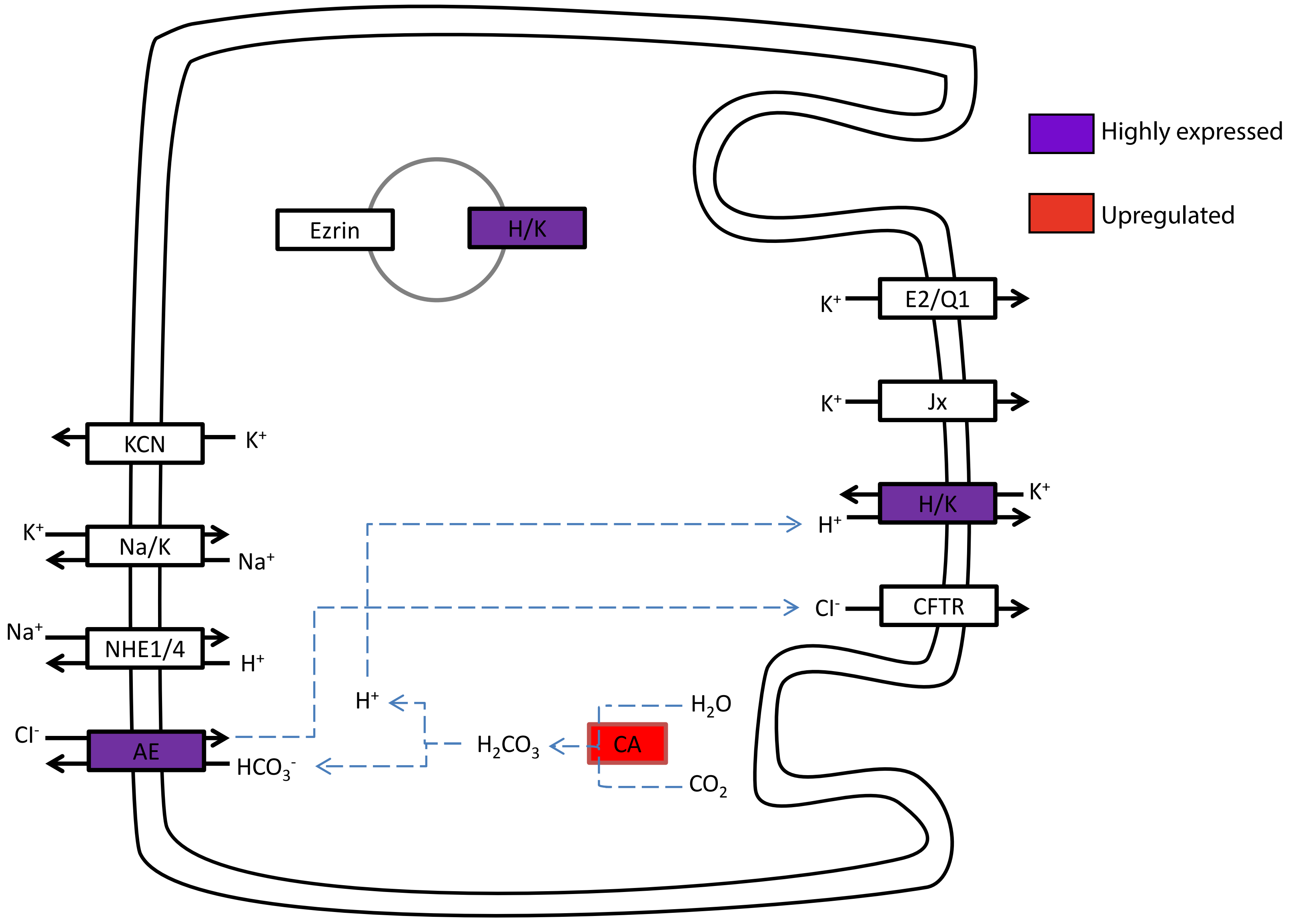

Figure S4

Click here to access/download
Supplementary Material
FigureS4.pdf

Figure S5

Click here to access/download
Supplementary Material
FigureS5.pdf

Figure S6

Click here to access/download

Supplementary Material

FigureS6.pdf

Figure S7

Click here to access/download
Supplementary Material
FigureS7.pdf

Figure S8

Click here to access/download
Supplementary Material
FigureS8.pdf

Figure S9

Click here to access/download
Supplementary Material
FigureS9.pdf

Table S3

Click here to access/download
Supplementary Material
TableS3.xlsx

Table S5

Click here to access/download
Supplementary Material
TableS5.xlsx

Table S6

Click here to access/download
Supplementary Material
TableS6.xlsx

Table S7

Click here to access/download

Supplementary Material

TableS7.xlsx

Table S8

Click here to access/download
Supplementary Material
TableS8.xlsx

Table S9

Click here to access/download
Supplementary Material
TableS9.xlsx

Table S10

Click here to access/download
Supplementary Material
TableS10.xlsx

Table S11

Click here to access/download
Supplementary Material
TableS11.xlsx

Table S12

Click here to access/download
Supplementary Material
TableS12.xlsx

Table S13

Click here to access/download
Supplementary Material
TableS13.xlsx

Table S14

Click here to access/download
Supplementary Material
TableS14.xlsx

Click here to access/download
Supplementary Material
supplementary material.docx

FASTA sequences of misassembled transcripts

Click here to access/download
Supplementary Material
AU_assembly.misassembled.fasta

 Click here to download Personal Cover Answer sheet to reviewers comments.pdf

Dear Editor,

Thank you for returning the constructive and useful comments from the two reviewers that kindly evaluated our manuscript entitled “Transcriptome Analysis of the Response of Burmese Python to Digestion” that we submitted for publication in GigaScience. Both reviewers provided positive overall assessments, but also raised a number of specific queries to be addressed in the revision. We are pleased to return a revised manuscript where we have followed all the advice given by the two reviewers. The responses to each query is listed in a separate PDF file where you can see our responses describing the changes we have made to the manuscript. We greatly appreciate these comments and feel the manuscript has been improved in this review-process. We hope you will find the revised manuscript acceptable for publication in GigaScience and we are looking forward to hearing from you in due course. Please do not hesitate to contact me in case you need additional information.

Sincerely yours

Jinjie Duan (on behalf of all the authors)

Reviewer #1:

The authors used a transcriptomic time-series of five different organs and complementary proteomic surveys to characterize changes in expression following a feeding event in the Burmese Python.

I recommend the acceptance of this manuscript pending revisions. The only major criticism I have for the current manuscript is the lack of comparison to previous studies on this system and believe a section should be added to the manuscript explicitly comparing the time-series transcriptome data of this paper with others (see below). Comments below.

Introduction

After reading the introduction, it was unclear what knowledge this paper would add considering time-series transcriptome sequencing of particular organs (e.g., heart, liver) has already been performed (e.g., Castoe et al., 2013 PNAS). There is novel work being done in the form of additional tissues and, particularly, the proteomics, and I think the authors should state this explicitly to make it clear to the reader what is novel.

This is a good point and we now emphasize that we have repeated measurements in some tissues, but bring new data regarding the pancreas and the stomach as well as a new time point (48h) into the digestive period. These changes have been made in the second paragraph of the section entitled “background”.

Methods

Page 26 lines 526-527: Why were the biopsies pooled? This reduced the n from two to one.

At the time (2011) it was for technical and economic reasons, two samples were pooled to average out some of the variability among biological replicates. Yet, we regret not having more biological replicates since this restricts our analysis to highly expressed genes. Nevertheless, by virtue of our three time points (fasting, 24h and 48h, respectively), we do present some biological replication.

Page 27 lines 541-542: Why were samples pooled by tissue? What was the justification?

We included some normalized libraries to sample as broadly from the transcriptome as possible. This also included sampling over the different tissues, such that we would obtain some reads also from lowly expressed, tissue-specific genes. We have added an explanation in line 583 “In addition, to sample as broadly from transcriptome as possible, we also produced normalized libraries for each tissue in order to capture the reads from lowly expressed, tissue-specific genes.”

Page 28 lines 554-560: Although the authors used relatively long k-mers for assembly, they should still perform a specific check for mis-assembled chimeric sequences, especially considering the reads from all libraries were pooled to assemble a reference (see Yang and Smith 2013 BMC Genomics), and this reference was the basis for all subsequent transcriptomic analyses.

To check the mis-assembled chimeric sequences, we have now compared our assembly with the reference genome (Castoe et al) and gene sequences using rnaQUAST, and amended the result (lines 101-115) and method (lines 606-608) sections, correspondingly. The result shows our transcriptome assembly had 34,423 transcripts in total. 34,040 (98%) transcripts had at least 1 significant alignment to the reference genome and 31,102 out of 34,040 were uniquely aligned. Average aligned fraction (i.e. total number of aligned bases in the transcript divided by the total transcript length) was 0.975. The total number of misassembled (chimeric) transcripts, which have discordant best-scored alignment (partial alignments that are either mapped to different strands/different chromosomes/in reverse order/too far away) was 1,974 (5.7%). The FASTA sequences of these misassembled transcripts are attached in Supplementary material.

Page 29 lines 578-579: Why was T-coffee used specifically for albumin-like genes? Justification should be added to the section.

We used T-coffee because it is well recommended for better accuracy of multiple sequences alignment (Thompson et al, 2011, Plos One; Pais et al,2014, Algorithms Mol Biol). Due to the improved analysis of the albumin-like sequences, we decided to move the “albumin-story” to the supplementary material (A more detailed explanation

is presented below under the responses to comments raised to our “analyses”). The justification of using T-coffee was added in the supplementary material in line 10: “We did multiple sequences alignment of these paralogues genes together with predicted ORF sequences of our five sequences using T-coffee (version 11.00) [2] with default parameters which is well recommended for better accuracy of multiple sequence alignment [3, 4]”.

Page 31 line 623: I failed to follow the text and come up with 6 samples. Were two snakes at 400 g and two snakes at 800 g fed a rodent? If so, these samples, along with the peptone control, equals five. If only one snake at 400 g and one snake at 800 g were fed a rodent, these samples, again along with the control, equal three. I do not see how the authors collected six samples. Was there also an n of two for each of these groups? Additionally, how was plasma collected?

We apologize for the confusing manner in which we originally described these procedures. We have altered to the text in line 650 and 661 to clarify that samples were obtained in duplicate from three individuals snakes (two upon digestion of a rodent meal, as well as one snake that had been fed peptone). We have also added a description in line 668 to explain that the blood (plasma) samples were obtained by cardiac puncture.

Analyses

Page 8 lines 124-129: How dissimilar were the transcripts (i.e.,sequence divergence)? Were the six albumin-like proteins identified in the MS analysis the six most highly-expressed albumin-like transcripts? In other words, was there a detection bias in your MS analyses against low-abundance transcripts? I have seen this in my work (e.g., Rokyta et al., 2015 G3). Also, can the authors be sure that these are different copies and not alternatively spliced transcripts?

We appreciate this concern. After performing additional analyses (phylogenetic analysis and alignment against reference genome), we conclude these albumin-like sequences are most likely alternatively spliced transcripts, rather than paralogues. Therefore, we feel that this part of our results do not longer present sufficient new advance to be discussed in the main text. We accordingly decided to move the albumin results to supplementary material in lines 2-26. In addition, we did observe a

discordance between transcriptome and proteome in our study, which may be due to delayed protein synthesis and degradation. However, the imbalance didn’t affect albumin-like transcripts because these six albumin-like transcripts were the six most highly expressed albumin-like transcripts in liver.

Line 197: "the five most abundant proteases identified in the gastric juice": How was protein quantitation performed? The methods do not mention protein quantitation. Are these simply based on spectral counts? If the authors are attempting to quantify the proteome, a more complete transcriptome-proteome comparison is warranted.

We apologize for not clarifying the method in where we originally described. We have added a description to explain the method used on protein quantitation (line 710): “Semi-quantitative proteomics data was obtained using the emPAI-values given by the Mascot 2.5.0 software after analysis of the MS/MS data [67].”

Lines 201, 208: carolinensis should not be capitalized

Thank you for spotting this mistake. It has now been corrected.

Perhaps the largest gap in the current study was the lack of a comparison to previous, extremely similar work on this system (e.g., Castoe et al., 2013 PNAS and Andrew et al.,2015 Physiol. Genomics). How do the authors' results compare to those of previous studies? Were they largely congruent? A section explicitly comparing the current study to previously published works should be added.

For the gene expressions in the intestine, heart and liver where previous data exist (Castoe et al., and Andrew et al), we have added a paragraph in the discussion (second paragraph of the new discussion) describing the overlap of upregulated genes in our and the previous studies. Information on the methods of comparison are now described in the supplementary material. It is noteworthy that the data from the liver was rather similar between studies, whereas the heart and small intestine revealed rather large differences between the studies.

Figures and Tables

Information in table 2 should be provided with the KEGG pathway figures.

We have added the missing information on the color-coding in the KEGG pathway that illustrates the gastric acid secretion (figure 10).

How were the sub-clusters in Figure 3 chosen? Do these represent all of the DEGs for that tissue?

The heat maps in Figure 3 show, for each tissue, all the genes that are both highly and differentially expressed with strict thresholds (defined in section "identification of DEGs and clustering analysis in Method section). We chose those sub-clusters because they represent a cluster of all upregulated genes, which are expected to be involved many functional changes during digestion.

Other

Small grammatical errors throughout, particularly in the discussion.

We have edited the manuscript carefully and hope we have corrected all grammatical mistakes.

Reviewer #2:

Duan et al. conduct a broad study using transcriptomic and proteomic methods to understand the molecular underpinnings of extreme physiological responses to feeding in Burmese pythons. Overall, the data collected are extensive and reasonably analyzed, and the manuscript is well written. The lack of replication and thorough analyses substantially limit the conclusions and novelty of the study, although generally I do believe that the manuscript is reasonable and valid in its current form. As such, given the aims of the journal, I do believe this manuscript does fit within its scope, as a sound descriptive study associated with a large amount of data that benefits from having these data directly linked to the paper. Below I note a handful of concerns and suggestions that would improve the ms.

I found it interesting that the authors chose to use de novo transcript assemblies rather than the annotated gene set available for the Burmese python genome. The authors make the case that the genome is somewhat fragmentary, which is true, and that this justified the use of a de novo assembly. While I don't completely agree, I do believe that their use of the de novo transcript assembly for mapping RNAseq data is reasonable, and what they find seems quite sensible. I am surprised, however, that they did not compare their annotations in any way to the annotated gene set on NCBI.

We appreciate these good comments. We have compared our assembly with annotated gene set in NCBI using rnaQUAST, and have updated the corresponding result and method section. It now reads in lines 115-120 "The comparison of assembled sequences and reference gene sequences (Supplementary table S3) showed that 26,320 (77.3%) assembled transcripts cover at least one isoform from the reference gene set and the mean fraction of transcript matched is 67.8%, suggesting there is a good concordance but also some differences which can be due to errors in either the reference genome assembly/annotation or our assembly".

Unfortunately, the authors did not have any replication in their RNAseq or proteomic data, and therefore any meaningful statistical comparisons are made difficult - for example, it is difficult to get decent estimates of how many genes are statistically differentially expressed across time points for organ-specific time course analyses. I

assume this is why the authors instead use arbitrary cutoffs: "1) FPKM is greater than or equal to 400 in at least one time point and 2) fold change is greater than or equal to 2 in at least one pairwise comparison among three time points." Without replication, I suppose the authors are somewhat limited in what they can do, and I do accept what they did as reasonable. However, they should avoid any instances of using the word "significant" throughout the text, which they use several times (e.g., LINE: 279: genes with significantly increased expression during digestion"). Honestly, they don't really have the power to detect significance with these data.

We agree that we should avoid the word significant when discussing the results since it is likely read as meaning statistically significant which we cannot know. Consequently, we have moved all "significant" throughout the manuscript.

I am concerned about what might be an over-interpretation of the findings from serum proteomics studies. The authors claim to have found a peptide that they identify in the serum as the protease inhibitor "anti-haemorrhagic factor cHLP-B (m.27_Py95)", and go on to conclude that "Our data supports older studies that identify these inhibitors of the deleterious action of venom enzymes in non-venomous snakes [32]."… My sense is that they should tone down their conclusion because 1) the python isn't venomous (and thus has no need for such proteins), and 2) the inference is simply based on blast homology with what is likely available online (venomous snake blood peptides). I think the finding is interesting and notable, but their inference of the function of this peptide being directly linked to resistance to venom is quite far fetched - more likely it may be indicative of a class of plasma peptides that could have been recruited in venomous snakes for self-defense against self-envenomation.

We agree with the reviewer's point and have changed the words accordingly in lines 255-259 such that it now reads "This is a protease inhibitor of the haemorrhagic-causing metalloproteinases present in snake venom and these inhibitors have previously been purified from serum of venomous snakes and thoroughly characterized [32, 33]. The role of such a protease inhibitor in non-venomous pythons is not obvious, but it has been proposed that they inhibit the deleterious action of venom enzymes in non-venomous snakes [34]."

Discussion section "Physiological interpretation of the upregulated genes in the intestine" - this section is noticeably lacking any citations or linking of results to a previously published in-depth transcriptional study of the python intestine (citation #13). There are also a number of incorrect claims made here (e.g., LINE 379: "It remains, however, unknown to what extent the increased capacity for nutrient uptake is also driven by increased synthesis of nutrient transporters".) that in fact have been clearly demonstrated in citation 13 - these links and statements made in this section need to be carefully re-written to more meaningfully incorporate this previous work.

We agree and appreciate this criticism. In the revised manuscript, we now give more credit to the previous studies in postprandial gene expression and we point more specifically to where there are differences between their findings and those reported by us. We hope you find the revised manuscript to be better balanced.

The figures should be improved for reading as a printed article. For example, there are multiple heat maps that are enormous, and are not printable in any reasonable way that would allow the labels to be read (e.g., Fig. 7). Simply spanning these over multiple columns would at least help with this. Also, while I realize that Gigascience is an online journal, the use of 15 in-text figures seems to be counter-productive for having there be clear points conveyed by the MS, and make the manuscript appear more like a massive data dump rather than a paper.

We have splitted the long heatmaps (Figures 5 and 7) into two columns to enable the reading of the labels.

We have moved the original Figures 9-13,15 and Tables 4-5 to supplementary material.

Copy Edits:

I suggest searching throughout the manuscript and writing out any numbers less than 10. For example: writing out four rather than 4.

Thanks for the comment. We have corrected them throughout the text.

Line 201 (and elsewhere) - change to: Anolis carolinensis (here and throughout the MS so that specific name is lower case)

Thanks for the comment. We have corrected them throughout the text.
