# Supplementary material for: Transcriptome analysis of the response of Burmese python to digestion
Source: Gigascience. 2017 Jul 13;6(8):1–18. doi: 10.1093/gigascience/gix057 (PMC5597892; doi:10.1093/gigascience/gix057)
Supplement: Supplement Materials [file gix057_Supp.zip › supplementary material.docx]

**Supplementary material**

Assembled sequences annotated with serum albumin

We observed 12 assembled sequences annotated with ‘serum albumin [Trimeresurus flavoviridis]’ (Supplementary Table S13). After filtering mis-assembled chimeric sequences (annotated by rnaQUAST [[1](#_ENREF_1)]), eight out of 12 sequences were kept. Then we filtered out sequences without predicted open reading frame (ORF) and obtained five sequences (accession ID: L32, L122, L445, L986, L3747). To check whether these five serum albumin-like sequences are paralogues or alternatively spliced variants, we collected amino acid sequences of paralogous genes of serum albumin in human, chicken, lizard, cobra and python from NCBI database. We did multiple sequences alignment of these paralogues genes together with predicted ORF sequences of our five sequences using T-coffee (version 11.00) [[2](#_ENREF_2)] with default parameters which is well recommended for better accuracy of multiple sequence alignment [[3](#_ENREF_3), [4](#_ENREF_4)] and constructed the phylogenetic tree using IQ-TREE [[5](#_ENREF_5)] version 1.5.3 with parameters “-m TEST -bb 1000 -alrt 1000”. The tree (Supplementary Fig. S10) indicates all five sequences are within one cluster together with two spliced variants of serum albumin from NCBI Burmese python reference gene set, suggesting our five sequences are spliced variants, rather than paralogues. Furthermore, we aligned these five sequences against reference genome using GMAP [[6](#_ENREF_6)] version 2014-09-29 with parameter ‘intronlength = 30000’ and found four of them were aligned to the same scaffold and clustered together with some degree overlap (Supplementary Fig. S11), supporting they are more likely to be spliced variants. In addition, we identified three (accession ID: L32, L122, L3747) (Supplementary Fig. S10) out of these five sequences at protein-level by a proteomics analysis (LC-MS/MS) of python plasma (Supplementary Table S14). It suggests that python has at least three alternatively splice variants, whereas python reference gene set in NCBI has two.

Comparisons between our upregulated genes and upregulated genes from Castoe et al (2013)

Both of two studies have sampled heart, liver and small intestine at fasting time point and 24h post-feeding time point. Therefore, we made a comparison of detected upregulated genes between two studies for each of these three tissues at 24h. We applied our stringent threshold (FC >= 2 and maxium FPKM (of fasting and 24h) >= 400) for indetifying upregulated genes and obtained 15, 93 and 61 upregulated genes for heart, liver and small intestine, respectively.

To determine orthologs between two studies, we performed reciprocal BLAST search and compiled a list of the reciprocal best hits (RBHs). We have downloaded supporting information dataset sd01.xlsx with gene expression values from Castoe et al (2013). By linking our indentified upregualted gene IDs to corresponding RBHs in dataset sd01, we found 51 out of 93 in liver were also upregulated (the corresponding FC ranged from 1.2 to 156) in Castoe’s study, and 10 out of 61 in intestine were upregulated (the corresponding FC ranged from 4.4 to 60.2) in Castoe’s study, whereas none of genes in heart were identified as upregulated genes in Castoe’s study which may due to limited biological replicates in our study.

**Supplementary Figures**


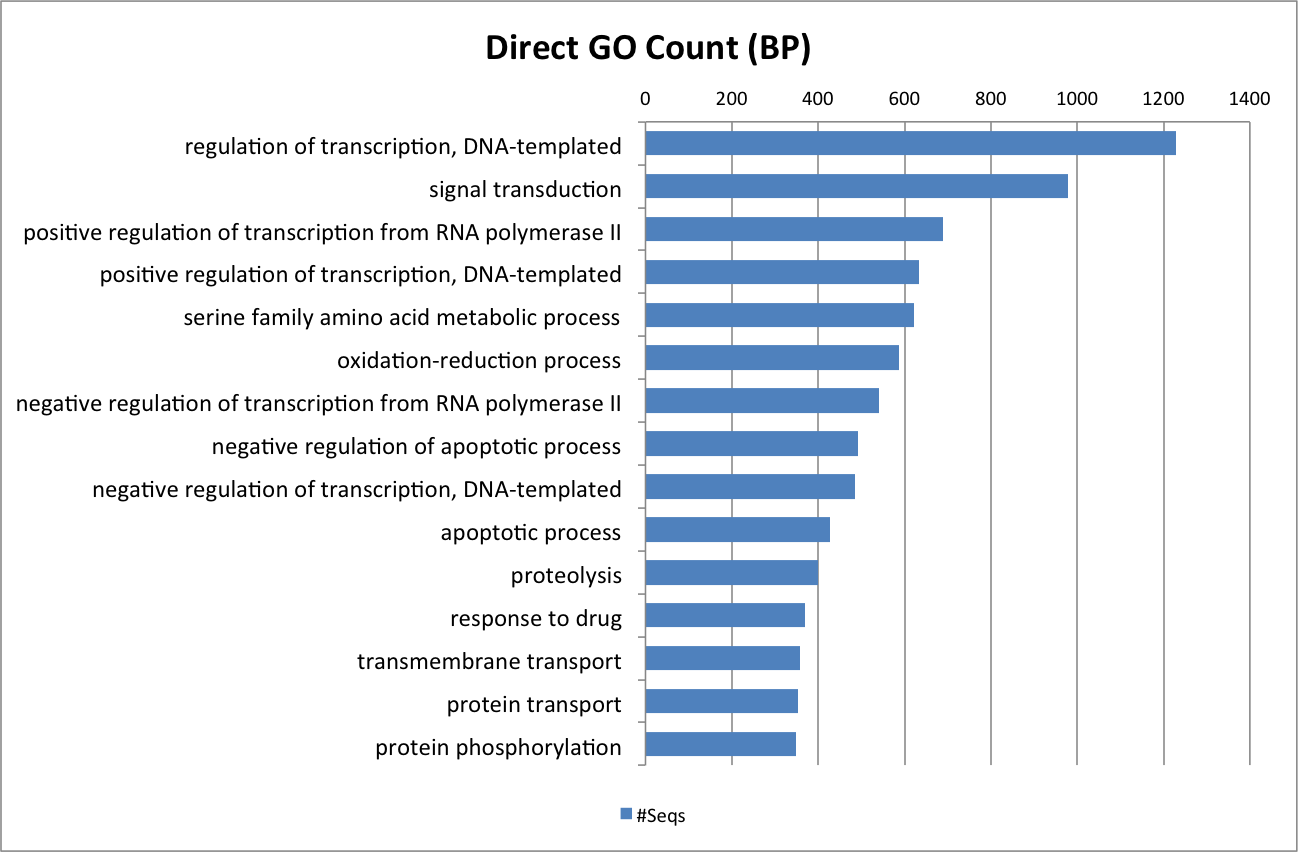

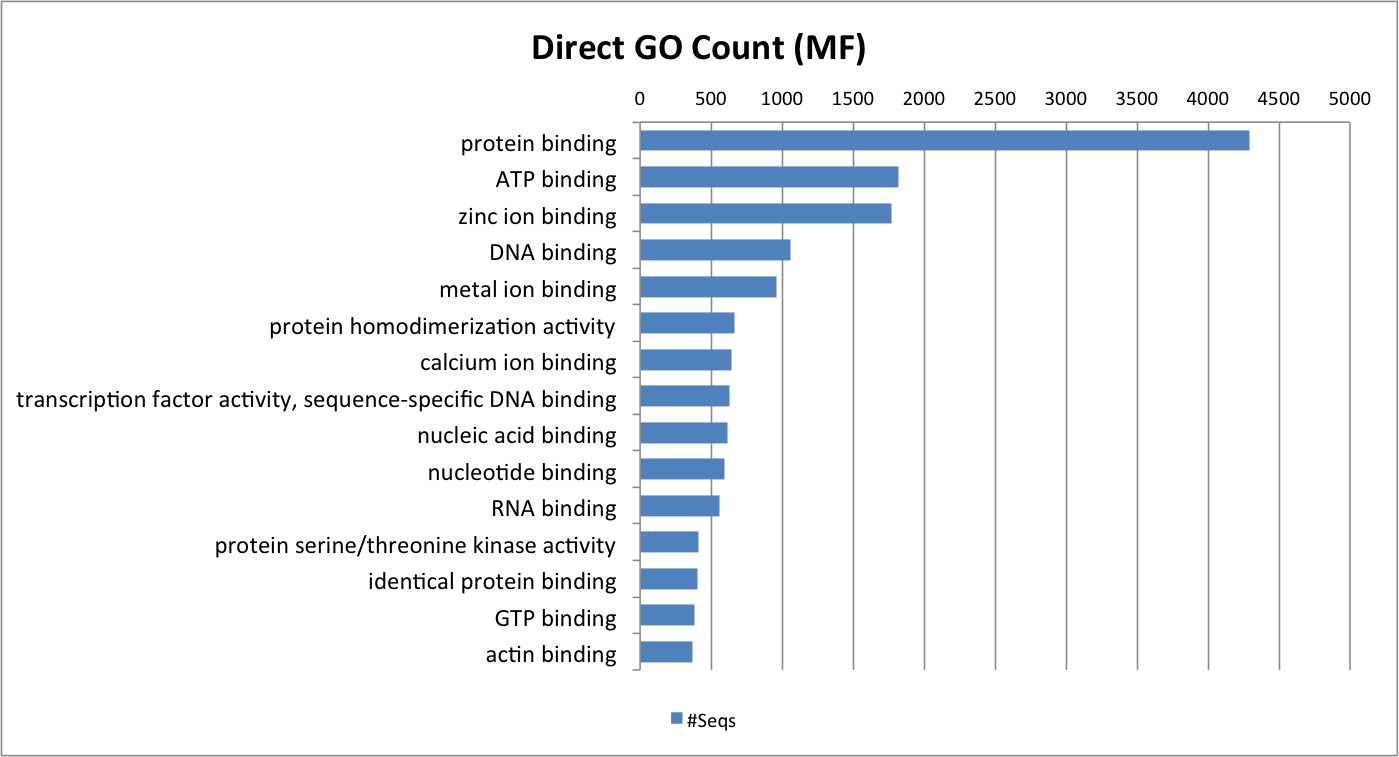

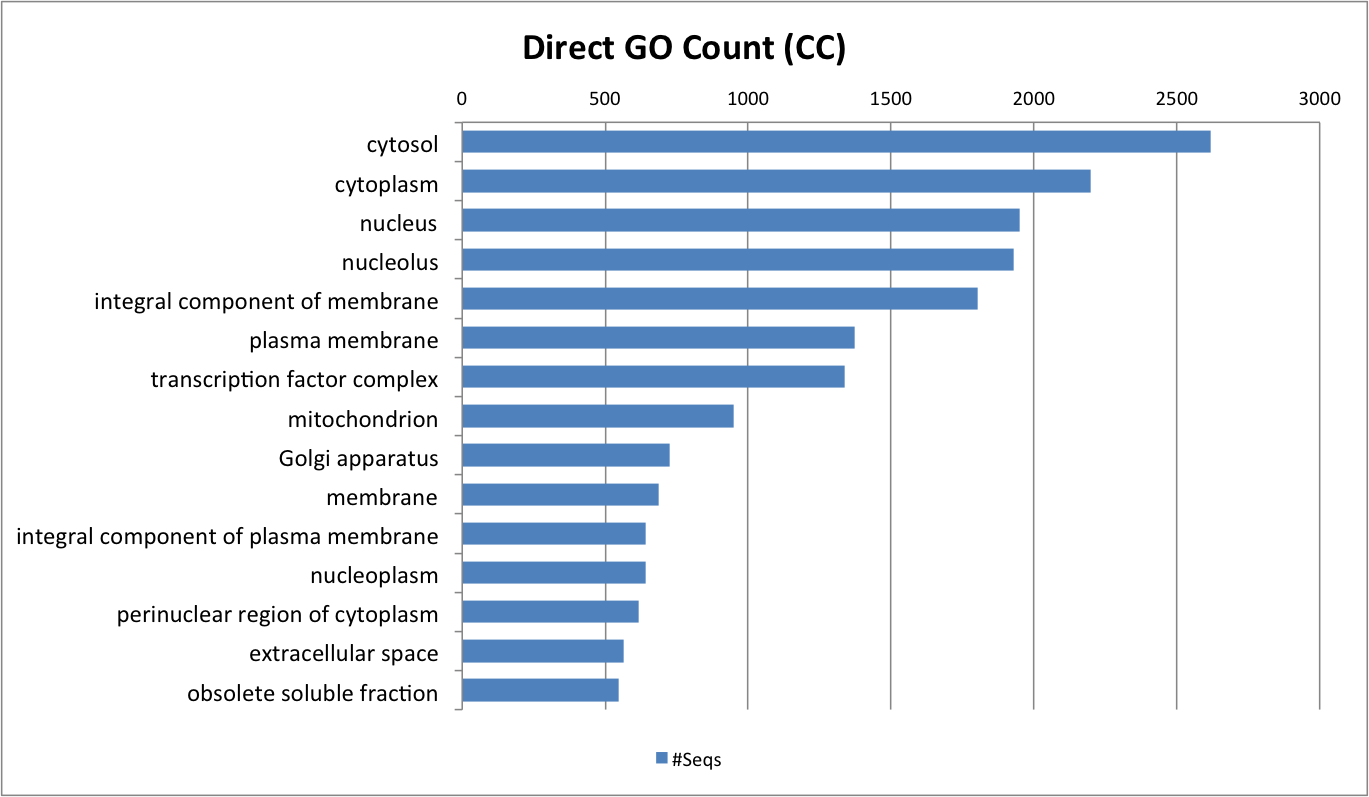


Figure S1. The distributions of the top 15 most frequently identified GO terms of assembled transcripts. It categories for biological process (BP), molecular function (MF) and cellular component (CC).

Figure S2. Venn diagrams showing overlapping expressed genes in different tissues under each digesting time point. Firstly, we kept genes which have FPKM >= 100 in at least one sample out of 15 samples. Then the number of expressed genes in each library was counted with FPKM >=1.

Figure S3. PCA applied to 1862 genes identifies 5 trends that explain ~58% of the total expression variance with the first three PCs together.

Figure S4. The enriched GO terms of target genes in stomach. Using all 16992 transcripts annotated with GO terms as reference background, we set (a) 481 upregulated genes and (b) 182 highly expressed genes respectively as test set. The GO terms found over/under represented by a two-tailed Fisher Exact test with multiple testing correction of FDR (Benjamini and Hochberg) below 0.001. The GO terms are categorized and colored as three ontology categories: cellular component in green, molecular function in orange and biological process in black. The pie charts in the left corner display proportion of three categories.

Figure S5. The enriched GO terms of target genes in intestine. Using all 16992 transcripts annotated with GO terms as reference background, we set (a) 689 upregulated genes and (b) 188 highly expressed genes respectively as test set. The GO terms found over/under represented by a two-tailed Fisher Exact test with multiple testing correction of FDR (Benjamini and Hochberg) below 0.001. The GO terms are categorized and colored as three ontology categories: cellular component in green, molecular function in orange and biological process in black. The pie charts in the left corner display proportion of three categories.

Figure S6. The enriched GO terms of target genes in pancreas. Using all 16992 transcripts annotated with GO terms as reference background, we set (a) 376 upregulated genes and (b) 205 highly expressed genes respectively as test set. The GO terms found over/under represented by a two-tailed Fisher Exact test with multiple testing correction of FDR (Benjamini and Hochberg) below 0.001. The GO terms are categorized and colored as three ontology categories: cellular component in green, molecular function in orange and biological process in black. The pie charts in the left corner display proportion of three categories.

Figure S7. The enriched GO terms of target genes in liver. Using all 16992 transcripts annotated with GO terms as reference background, we set (a) 606 upregulated genes and (b) 308 highly expressed genes respectively as test set. The GO terms found over/under represented by a two-tailed Fisher Exact test with multiple testing correction of FDR (Benjamini and Hochberg) below 0.001. The GO terms are categorized and colored as three ontology categories: cellular component in green, molecular function in orange and biological process in black. The pie charts in the left corner display proportion of three categories.

Figure S8. The enriched GO terms of target genes in heart. Using all 16992 transcripts annotated with GO terms as reference background, we set (a) 107 upregulated genes and (b) 213 highly expressed genes respectively as test set. The GO terms found over/under represented by a two-tailed Fisher Exact test with multiple testing correction of FDR (Benjamini and Hochberg) below 0.001. The GO terms are categorized and colored as three ontology categories: cellular component in green, molecular function in orange and biological process in black. The pie charts in the left corner display proportion of three categories.

Figure S9. Protein sequence alignment of python progastricsin with progastricsin sequences from Anolis carolinensis and from human. The two longest of the five python protein sequences identified in the gastric juice and annotated as progastricsin-like, were aligned with the two sequences from anole used for the annotation and with the human homolog. The degree of conservation of the individual residues are indicated below the alignment, the active site residues are highlighted in yellow (Asp91, Tyr134, and Asp277 in the human variant – based on MEROPS *the peptidase database*), the cysteine residues are shown in red, and the N-terminal of the activated human gastricsin is highlighted in green. The alignment illustrates that the python’s most abundant proteolytic digestive enzymes, the gastricsins, are similar to gastricsins from other species.


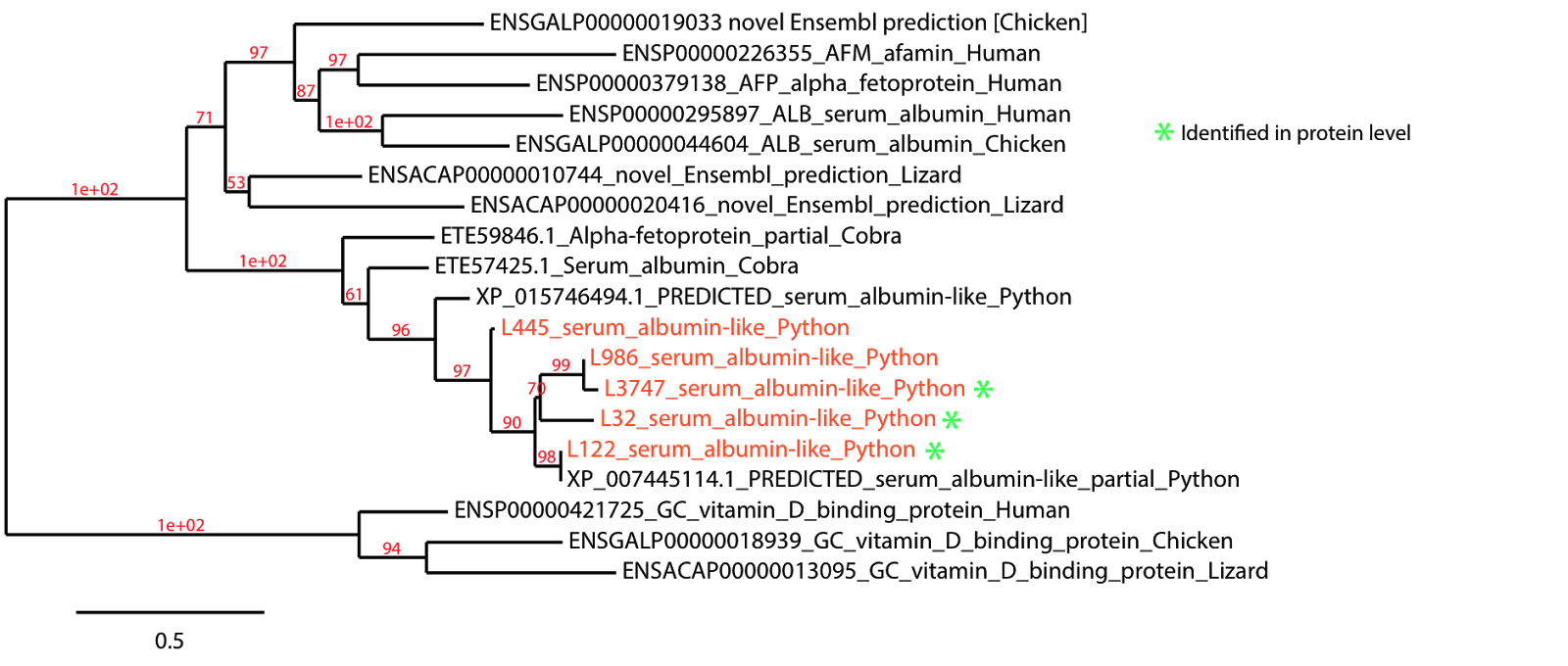


Figure S10. The phylogenetic tree of assembled albumin-like sequences and paralogous genes of serum albumin in human, chicken, lizard, cobra and python from NCBI database. The name ID in red is assembled sequences. Assembled sequences identified in protein level are labelled with a green star behind the name ID.


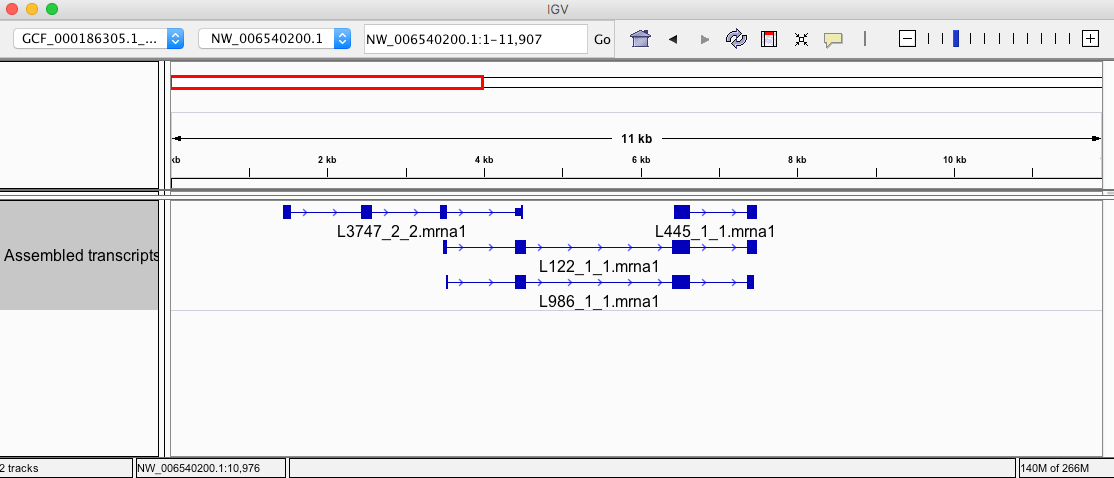


Figure S11. Alignment of four assembled albumin-like sequences against reference genome.

**Supplementary Tables**


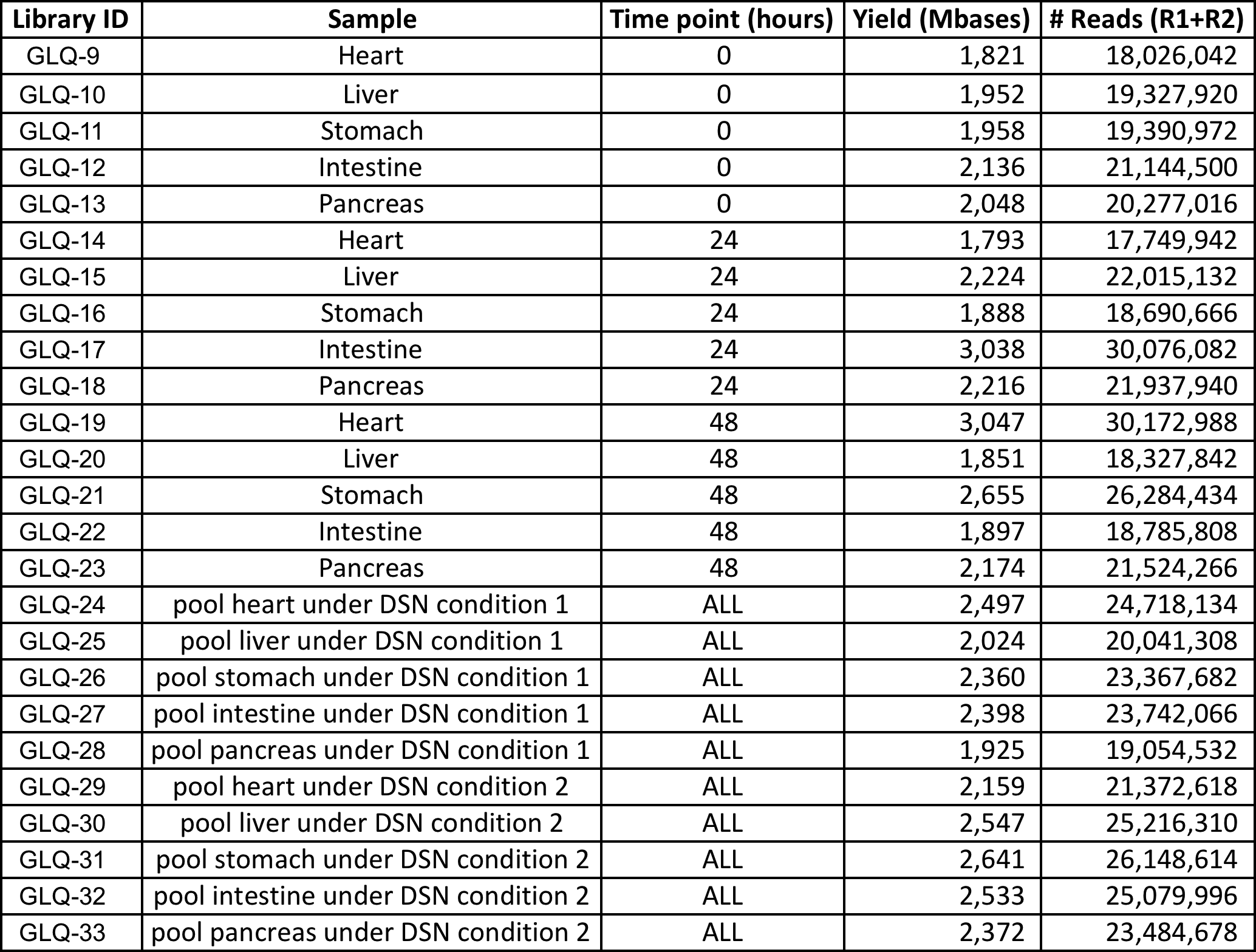


Table S1. Summary statistics of Transcriptome sequencing raw data. 25 paired-end (insert size ~150-250 bp) libraries were sequenced using Illumina HiSeq 2000 platform. The length of one-end read was 100bp.

Table S2. Performance of different k-mer transcriptome assembly using assembler Velvet and Oases. The scaffold N50 is the length of the smallest scaffold in the set that contains the fewest (largest) scaffolds whose combined length represents at least 50% of the assembly. Total mapped reads is the number of high quality paired reads that map back to the assembly produced using a given k-mer.

 Table S4. The criteria for assigning the best description of nr database annotation. The right column shows how the regular expression used in Perl language.

## Reference

1. Bushmanova E, Antipov D, Lapidus A, Suvorov V, Prjibelski AD: **rnaQUAST: a quality assessment tool for de novo transcriptome assemblies.** *Bioinformatics* 2016, **32:**2210-2212.

2. Notredame C, Higgins DG, Heringa J: **T-Coffee: A novel method for fast and accurate multiple sequence alignment.** *J Mol Biol* 2000, **302:**205-217.

3. Thompson JD, Linard B, Lecompte O, Poch O: **A Comprehensive Benchmark Study of Multiple Sequence Alignment Methods: Current Challenges and Future Perspectives.** *Plos One* 2011, **6**.

4. Pais FS, Ruy Pde C, Oliveira G, Coimbra RS: **Assessing the efficiency of multiple sequence alignment programs.** *Algorithms Mol Biol* 2014, **9:**4.

5. Nguyen LT, Schmidt HA, von Haeseler A, Minh BQ: **IQ-TREE: a fast and effective stochastic algorithm for estimating maximum-likelihood phylogenies.** *Mol Biol Evol* 2015, **32:**268-274.

6. Wu TD, Watanabe CK: **GMAP: a genomic mapping and alignment program for mRNA and EST sequences.** *Bioinformatics* 2005, **21:**1859-1875.
