# Supplementary material for: Transcriptome analysis of the response of Burmese python to digestion
Source: Gigascience. 2017 Jul 13;6(8):1–18. doi: 10.1093/gigascience/gix057 (PMC5597892; doi:10.1093/gigascience/gix057)
Supplement: Response-to-Reviewer-Comments_Original-Submission.pdf [file gix057_Response-to-Reviewer-Comments_Original-Submission.pdf]

Author's Response To Reviewer Comments

Dear Editor,

Thank you for returning the constructive and useful comments from the two reviewers that kindly evaluated our manuscript entitled “Transcriptome Analysis of the Response of Burmese Python to Digestion” that we submitted for publication in GigaScience. Both reviewers provided positive overall assessments, but also raised a number of specific queries to be addressed in the revision. We are pleased to return a revised manuscript where we have followed all the advice given by the two reviewers. The responses to each query is listed in a separate PDF file entitled "Answer sheet to reviewers comments" attached as personal cover where you can see our responses describing the changes we have made to the manuscript. We greatly appreciate these comments and feel the manuscript has been improved in this review-process. We hope you will find the revised manuscript acceptable for publication in GigaScience and we are looking forward to hearing from you in due course. Please do not hesitate to contact me in case you need additional information.

Sincerely yours

Jinjie Duan (on behalf of all the authors)
