# Supplementary material for: Transcriptome analysis of the response of Burmese python to digestion
Source: Gigascience. 2017 Jul 13;6(8):1–18. doi: 10.1093/gigascience/gix057 (PMC5597892; doi:10.1093/gigascience/gix057)
Supplement: Response-to-Reviewer-Comments_Revision-1.pdf [file gix057_Response-to-Reviewer-Comments_Revision-1.pdf]

Author's Response To Reviewer Comments

Dear editor and reviewer,

Thank you very much for providing another round of constructive criticism, which we have taken into account when revising the manuscript. Specifically, we have paid careful considerations to provide evidences when making quantitative comparisons within our own data (particularly when comparing mRNA and protein measures) and with other published data (particularly the data from the “Castoe study”). As a result, we also deleted several statements where we are unable to provide quantitative comparisons. We thank the reviewer for these corrections/suggestions and we have answered all specific queries in the annotated PDF.

The raw proteomics data have been uploaded into the PRIDE repository and the accession number are quoted in the “Method” section.

Submission details:
Project Name: A Proteomic Survey of Enzymes in the Plasma and Gastric Fluid of Burmese Python
Project accession: PXD006665
Project DOI: Not applicable
Reviewer account details: Username: reviewer95252@ebi.ac.uk

We hope you will find the revised manuscript to be acceptable for publication in GigaScience, and we are looking forward to hearing from you in due course.

best wishes,
Jinjie on behalf of the all other authors.

Answer sheet:

Reviewer report #1: I have attached an annotated draft of the manuscript with comments.

Major comments are as follows:

1. The quality of writing still needs to be improved. I have highlighted some (but by no means all) grammatical mistakes in the annotated draft.
A: We have addressed all the specific queries and we have edited the entire manuscript.

2. The authors repeatedly reference "specific" hypotheses but never state what these hypotheses are.

A: We have deleted these general statements concerning specific hypotheses, but we obviously address a number of hypotheses in the individual sections of the discussion.

3. Figures and tables are cited but rarely discussed. The authors should discuss in the text the major findings and not rely solely on figures.
A: The figures and tables are specifically discussed and interpreted in the “Discussion section” and form the basis of the discussion of the postprandial changes in each organ.

4. The comparison to previous work is crude and vague. I would like to see some sort of quantitative comparison to previous work. The largest flaw of the current manuscript is this lack of quantitative comparisons. Specific quantitative work, especially for the transcriptome-proteome and proteome-proteome comparisons, needs to be added.
A: We have added quantitative measures of the transcriptome-proteome comparison. We have softened or deleted the sections with direct proteome-proteome comparisons to avoid the impression that we provide quantitative comparisons to the previous studies.

5. The raw proteomics data are not made available.
A: The raw proteomics data have been uploaded into the PRIDE repository and the accession number are quoted in the revised MS.
