# Supplementary material for: Transcriptome analysis of the response of Burmese python to digestion
Source: Gigascience. 2017 Jul 13;6(8):1–18. doi: 10.1093/gigascience/gix057 (PMC5597892; doi:10.1093/gigascience/gix057)
Supplement: Reviewer-1-Report-(Original-Submission).pdf [file gix057_Reviewer-1-Report-(Original-Submission).pdf]

Reviewer Report

Title: Transcriptome Analysis of the Response of Burmese Python to Digestion

Version: Original Submission **Date:** 12/9/2016

Reviewer name: Mark Margres

Reviewer Comments to Author:

The authors used a transcriptomic time-series of five different organs and complementary proteomic surveys to characterize changes in expression following a feeding event in the Burmese Python.

I recommend the acceptance of this manuscript pending revisions. The only major criticism I have for the current manuscript is the lack of comparison to previous studies on this system and believe a section should be added to the manuscript explicitly comparing the time-series transcriptome data of this paper with others (see below). Comments below.

Introduction

After reading the introduction, it was unclear what knowledge this paper would add considering time-series transcriptome sequencing of particular organs (e.g., heart, liver) has already been performed (e.g., Castoe et al., 2013 PNAS). There is novel work being done in the form of additional tissues and, particularly, the proteomics, and I think the authors should state this explicitly to make it clear to the reader what is novel.

Methods

Page 26 lines 526-527: Why were the biopsies pooled? This reduced the n from two to one.

Page 27 lines 541-542: Why were samples pooled by tissue? What was the justification?

Page 28 lines 554-560: Although the authors used relatively long k-mers for assembly, they should still perform a specific check for mis-assembled chimeric sequences, especially considering the reads from all libraries were pooled to assemble a reference (see Yang and Smith 2013 BMC Genomics), and this reference was the basis for all subsequent transcriptomic analyses.

Page 29 lines 578-579: Why was T-coffee used specifically for albumin-like genes? Justification should be added to the section.

Page 31 line 623: I failed to follow the text and come up with 6 samples. Were two snakes at 400 g and two snakes at 800 g fed a rodent? If so, these samples, along with the peptone control, equals five. If only one snake at 400 g and one snake at 800 g were fed a rodent, these samples, again along with the

control, equal three. I do not see how the authors collected six samples. Was there also an n of two for each of these groups? Additionally, how was plasma collected?

Analyses

Page 8 lines 124-129: How dissimilar were the transcripts (i.e., sequence divergence)? Were the six albumin-like proteins identified in the MS analysis the six most highly-expressed albumin-like transcripts? In other words, was there a detection bias in your MS analyses against low-abundance transcripts? I have seen this in my work (e.g., Rokyta et al., 2015 G3). Also, can the authors be sure that these are different copies and not alternatively spliced transcripts?

Line 197: "the five most abundant proteases identified in the gastric juice": How was protein quantitation performed? The methods do not mention protein quantitation. Are these simply based on spectral counts? If the authors are attempting to quantify the proteome, a more complete transcriptome-proteome comparison is warranted.

Lines 201, 208: carolinensis should not be capitalized

Perhaps the largest gap in the current study was the lack of a comparison to previous, extremely similar work on this system (e.g., Castoe et al., 2013 PNAS and Andrew et al., 2015 Physiol. Genomics). How do the authors' results compare to those of previous studies? Were they largely congruent? A section explicitly comparing the current study to previously published works should be added.

Figures and Tables

Information in table 2 should be provided with the KEGG pathway figures.

How were the sub-clusters in Figure 3 chosen? Do these represent all of the DEGs for that tissue?

Other

Small grammatical errors throughout, particularly in the discussion.

Methods

Are the methods appropriate to the aims of the study, are they well described, and are necessary controls included? Yes

Conclusions

Are the conclusions adequately supported by the data shown? Yes

Reporting Standards

Does the manuscript adhere to the journal's guidelines on minimum standards of reporting? YesChoose an item.

Statistics

Are you able to assess all statistics in the manuscript, including the appropriateness of statistical tests used? Yes, and I have assessed the statistics in my report.

Quality of Written English

Please indicate the quality of language in the manuscript: Acceptable

Declaration of Competing Interests

Please complete a declaration of competing interests, considering the following questions:

- Have you in the past five years received reimbursements, fees, funding, or salary from an organisation that may in any way gain or lose financially from the publication of this manuscript, either now or in the future?
- Do you hold any stocks or shares in an organisation that may in any way gain or lose financially from the publication of this manuscript, either now or in the future?
- Do you hold or are you currently applying for any patents relating to the content of the manuscript?
- Have you received reimbursements, fees, funding, or salary from an organization that holds or has applied for patents relating to the content of the manuscript?
- Do you have any other financial competing interests?
- Do you have any non-financial competing interests in relation to this paper?

If you can answer no to all of the above, write 'I declare that I have no competing interests' below. If your reply is yes to any, please give details below.

I declare that I have no competing interests.

I agree to the open peer review policy of the journal. I understand that my name will be included on my report to the authors and, if the manuscript is accepted for publication, my named report including any attachments I upload will be posted on the website along with the authors' responses. I agree for my report to be made available under an Open Access Creative Commons CC-BY license (http://creativecommons.org/licenses/by/4.0/). I understand that any comments which I do not wish to be included in my named report can be included as confidential comments to the editors, which will not be published.

I agree to the open peer review policy of the journal

To further support our reviewers, we have joined with Publons, where you can gain additional credit to further highlight your hard work (see: https://publons.com/journal/530/gigascience). On publication of this paper, your review will be automatically added to Publons, you can then choose whether or not to claim your Publons credit. I understand this statement.

Yes
