# Supplementary material for: Transcriptome analysis of the response of Burmese python to digestion
Source: Gigascience. 2017 Jul 13;6(8):1–18. doi: 10.1093/gigascience/gix057 (PMC5597892; doi:10.1093/gigascience/gix057)
Supplement: Reviewer-2-Report-(Original-Submission).pdf [file gix057_Reviewer-2-Report-(Original-Submission).pdf]

Reviewer Report

Title: Transcriptome Analysis of the Response of Burmese Python to Digestion

Version: Original Submission **Date:** 12/12/2016

Reviewer name: Todd Castoe

Reviewer Comments to Author:

Duan et al. conduct a broad study using transcriptomic and proteomic methods to understand the molecular underpinnings of extreme physiological responses to feeding in Burmese pythons. Overall, the data collected are extensive and reasonably analyzed, and the manuscript is well written. The lack of replication and thorough analyses substantially limit the conclusions and novelty of the study, although generally I do believe that the manuscript is reasonable and valid in its current form. As such, given the aims of the journal, I do believe this manuscript does fit within its scope, as a sound descriptive study associated with a large amount of data that benefits from having these data directly linked to the paper. Below I note a handful of concerns and suggestions that would improve the ms.

I found it interesting that the authors chose to use de novo transcript assemblies rather than the annotated gene set available for the Burmese python genome. The authors make the case that the genome is somewhat fragmentary, which is true, and that this justified the use of a de novo assembly. While I don't completely agree, I do believe that their use of the de novo transcript assembly for mapping RNAseq data is reasonable, and what they find seems quite sensible. I am surprised, however, that they did not compare their annotations in any way to the annotated gene set on NCBI.

Unfortunately, the authors did not have any replication in their RNAseq or proteomic data, and therefore any meaningful statistical comparisons are made difficult - for example, it is difficult to get decent estimates of how many genes are statistically differentially expressed across time points for organ-specific time course analyses. I assume this is why the authors instead use arbitrary cutoffs: "1) FPKM is greater than or equal to 400 in at least one time point and 2) fold change is greater than or equal to 2 in at least one pairwise comparison among three time points." Without replication, I suppose the authors are somewhat limited in what they can do, and I do accept what they did as reasonable. However, they should avoid any instances of using the word "significant" throughout the text, which they use several times (e.g., LINE: 279: genes with significantly increased expression during digestion"). Honestly, they don't really have the power to detect significance with these data.

I am concerned about what might be an over-interpretation of the findings from serum proteomics studies. The authors claim to have found a peptide that they identify in the serum as the protease inhibitor "anti-haemorrhagic factor cHLP-B (m.27_Py95)", and go on to conclude that "Our data supports older studies that identify these inhibitors of the deleterious action of venom enzymes in non-venomous snakes [32]."… My sense is that they should tone down their conclusion because 1) the python isn't venomous (and thus has no need for such proteins), and 2) the inference is simply based on blast

homology with what is likely available online (venomous snake blood peptides). I think the finding is interesting and notable, but their inference of the function of this peptide being directly linked to resistance to venom is quite far fetched - more likely it may be indicative of a class of plasma peptides that could have been recruited in venomous snakes for self-defense against self-envenomation.

Discussion section "Physiological interpretation of the upregulated genes in the intestine" - this section is noticeably lacking any citations or linking of results to a previously published in-depth transcriptional study of the python intestine (citation #13). There are also a number of incorrect claims made here (e.g., LINE 379: "It remains, however, unknown to what extent the increased capacity for nutrient uptake is also driven by increased synthesis of nutrient transporters"..) that in fact have been clearly demonstrated in citation 13 - these links and statements made in this section need to be carefully re-written to more meaningfully incorporate this previous work.

The figures should be improved for reading as a printed article. For example, there are multiple heat maps that are enormous, and are not printable in any reasonable way that would allow the labels to be read (e.g., Fig. 7). Simply spanning these over multiple columns would at least help with this. Also, while I realize that Gigascience is an online journal, the use of 15 in-text figures seems to be counter-productive for having there be clear points conveyed by the MS, and make the manuscript appear more like a massive data dump rather than a paper.

Copy Edits:

I suggest searching throughout the manuscript and writing out any numbers less than 10. For example: writing out four rather than 4.

Line 201 (and elsewhere) - change to: Anolis carolinensis (here and throughout the MS so that specific name is lower case)

Methods

Are the methods appropriate to the aims of the study, are they well described, and are necessary controls included? Yes

Conclusions

Are the conclusions adequately supported by the data shown? Yes

Reporting Standards

Does the manuscript adhere to the journal's guidelines on minimum standards of reporting? YesChoose an item.

Statistics

Are you able to assess all statistics in the manuscript, including the appropriateness of statistical tests used? Yes, and I have assessed the statistics in my report.

Quality of Written English

Please indicate the quality of language in the manuscript: Acceptable

Declaration of Competing Interests

Please complete a declaration of competing interests, considering the following questions:

- Have you in the past five years received reimbursements, fees, funding, or salary from an organisation that may in any way gain or lose financially from the publication of this manuscript, either now or in the future?
- Do you hold any stocks or shares in an organisation that may in any way gain or lose financially from the publication of this manuscript, either now or in the future?
- Do you hold or are you currently applying for any patents relating to the content of the manuscript?
- Have you received reimbursements, fees, funding, or salary from an organization that holds or has applied for patents relating to the content of the manuscript?
- Do you have any other financial competing interests?
- Do you have any non-financial competing interests in relation to this paper?

If you can answer no to all of the above, write 'I declare that I have no competing interests' below. If your reply is yes to any, please give details below.

I declare that I have no competing interests.

I agree to the open peer review policy of the journal. I understand that my name will be included on my report to the authors and, if the manuscript is accepted for publication, my named report including any attachments I upload will be posted on the website along with the authors' responses. I agree for my report to be made available under an Open Access Creative Commons CC-BY license (http://creativecommons.org/licenses/by/4.0/). I understand that any comments which I do not wish to be included in my named report can be included as confidential comments to the editors, which will not be published.

I agree to the open peer review policy of the journal

To further support our reviewers, we have joined with Publons, where you can gain additional credit to further highlight your hard work (see: https://publons.com/journal/530/gigascience). On publication of this paper, your review will be automatically added to Publons, you can then choose whether or not to claim your Publons credit. I understand this statement.

Yes
